# Pd-Catalyzed Heteroannulation Using *N*-Arylureas as a Sterically Undemanding Ligand Platform

**DOI:** 10.1021/jacs.2c01019

**Published:** 2022-04-05

**Authors:** Jakub Vaith, Dasha Rodina, Gregory C. Spaulding, Shauna M. Paradine

**Affiliations:** Department of Chemistry, University of Rochester, Rochester, New York 14627, United States

## Abstract

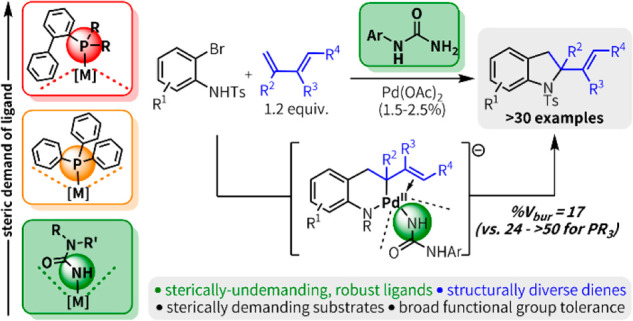

We report the development of ureas as sterically undemanding pro-ligands for Pd catalysis. *N*-Arylureas outperform phosphine ligands for the Pd-catalyzed heteroannulation of *N*-tosyl-*o*-bromoanilines and 1,3-dienes, engaging diverse coupling partners for the preparation of 2-subsituted indolines, including sterically demanding substrates that have not previously been tolerated. Experimental and computational studies on model Pd-urea and Pd-ureate complexes are consistent with monodentate binding through the nonsubstituted nitrogen, which is uncommon for metal-ureate complexes.

The development of ligand platforms is a key driver of innovation in homogeneous transition metal catalysis. This arises from the invaluable feature of transition metal-catalyzed reactions: the ability to effectively control the reactivity of a transition metal by modulating the properties of the ligand bound to the metal center. While ligand characteristics such as solubility or ligand rigidity are important, the two main influences controlling the reactivity of the metal center are the steric and electronic properties of the ligand. In palladium catalysis, significant reactivity breakthroughs have been achieved with sterically demanding, electron-rich ligands such as dialkylbiaryl phosphines and N-heterocyclic carbenes (NHCs).^1^

The privileged status of these ligands, however, has narrowed the focus of ligand discovery, with most modern development of ligands for Pd-catalyzed transformations falling within this space,^2^ leaving other areas along the steric-electronic ligand map largely neglected (Figure 1a). Sterically demanding, electron-deficient ligands have also seen substantial development,^3^ while the ligand space of small organic ligands is currently the most underdeveloped.^4^ We hypothesized that sterically undemanding ligands could be advantageous in reactions where the steric demands of key intermediates are high. However, such ligand space cannot be accessed with phosphines or NHCs. Since it has been shown that primary amine ligands are sterically undemanding,^5^ we decided to focus on ureas as pro-ligands for ureates to fill this steric-electronic ligand space gap (Figure 1b). In addition to their steric and electronic properties, ureas have practical advantages that make them attractive as an alternative ligand class; they are readily prepared from widely available amine precursors, are bench stable, and are robust to a variety of reaction conditions. Despite this, urea derivatives have remained virtually unexplored as ligands for late transition metals,^6,7^ even with a key precedent demonstrating their compatibility with Pd catalysis.^8,9^ Moreover, the few reports using amines or ureas as ligands for Pd catalysis make no reactivity comparison to traditional ligands,^4,8,9^ leaving it an open question whether complementary reactivity is possible by exploring this steric-electronic region of ligand space.

**Figure 1:**
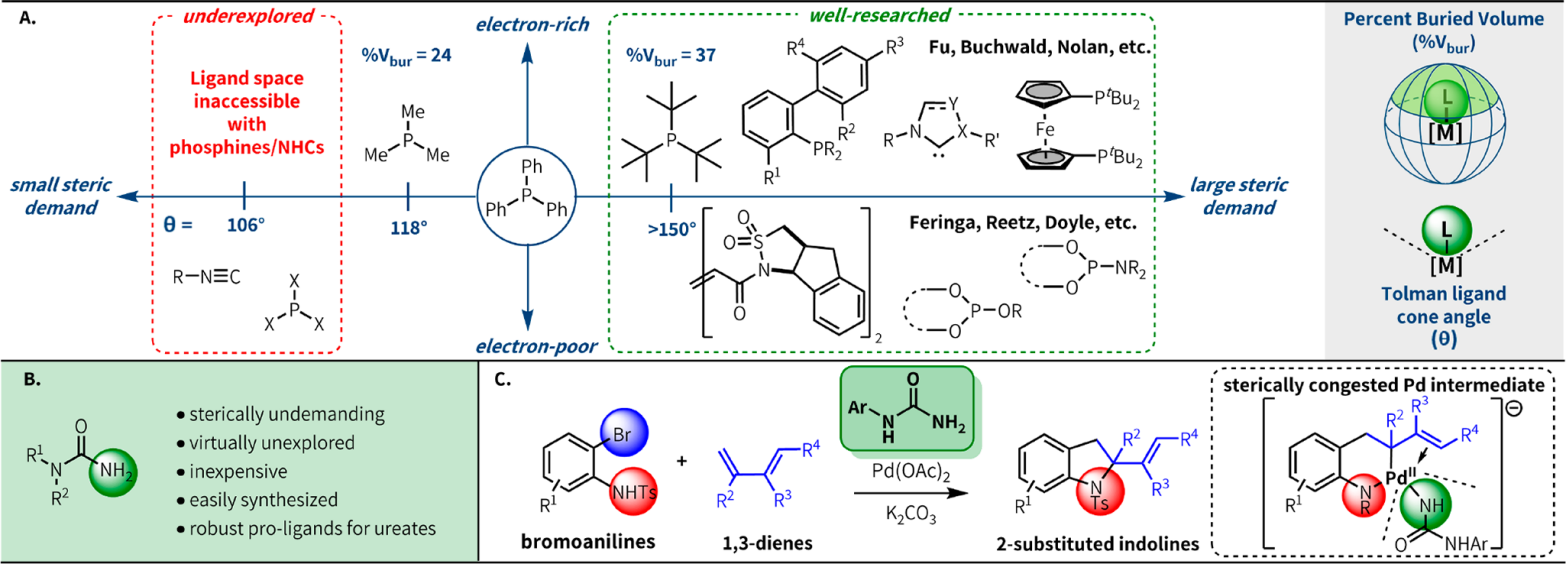
Accessing new regions of ligand space for Pd catalysis. (A) Organic ligands for late transition metal catalysis. (B) Ureas as a ligand platform for Pd catalysis. (C) This work: urea-enabled heteroannulation of bromoanilines and dienes.

We identified Pd-catalyzed heteroannulation of haloanilines and 1,3-dienes as an ideal transformation to test our ligand design hypothesis (Figure 1c). This convergent approach to indolic azaheterocycles, which are a privileged structural motif in drug discovery owing to their ubiquity in alkaloids,^10^ has received considerable interest. Despite important advances in both reactivity and enantioselectivity,^11−14^ two key limitations remain: (1) the only examples using bromoanilines (rather than iodoanilines) require strained olefins,^15^ and (2) existing methodologies demonstrate limited tolerance for steric bulk in either substrate. Additionally, when dienes are used as substrates, phosphine ligands are sometimes inhibitory in these reactions.^11b^ Although detailed mechanistic analysis is lacking, one possible reason for this inhibition is the increased coordination and steric requirements with dienes, which are thought to generate an η^3^-allyl complex upon migratory insertion,^11b^ relative to isolated olefins.

Herein, we present ureas as pro-ligands for ureates, sterically undemanding ligands for Pd-mediated reactions, while demonstrating their utility in the heteroannulation of structurally diverse *N*-tosylbromoanilines and 1,3-dienes. We also provide evidence that ureate binding to Pd(II) occurs preferentially through the nonsubstituted nitrogen, which is rare for transition metal–urea complexes;^16^ this provides preliminary insight into the function of these ligands.

Using *N*-tosyl *o*-bromoaniline (**1a**) and myrcene (**2a**) as model substrates, we investigated the competence of various ligands in the heteroannulation reaction (Table 1).^17,18^ Without exogenous ligand, product yield was modest (32% yield). Diverse phosphine ligands all inhibited the reaction (<20% yield), consistent with previous reports.^11b^ In contrast, we observed a significant improvement in product yield with urea (**4a**) and monosubstituted urea **4b**, affording indolines **3a/3a′** in ∼60% yield (**3a/3a′** = 90:10). While comparable yield could be achieved with further substitution as long as the urea bore a free −NH_2_ (e.g., **4c**), only a modest ligand effect was observed with *N,N′*-disubstituted urea **4d** and none with tri- and tetrasubstituted ureas (**4e**,**f**). We also systematically investigated related compounds bearing an −NH_2_ group. Amides (**5a**–**c**), thioureas (**5d**,**e**), and phenylguanidine (**5f**), although structurally similar to ureas, all inhibited the reaction, and no ligand effect was observed with carbamimidate **5g**, amines (**5h**–**j**), or pyridine,^19^ further highlighting the unique efficacy of ureas in these reactions. Only *O*-substituted carbamates bearing an −NH_2_ group showed a ligand effect (**5k**,**l**), though inferior to ureas **4a**–**c**. Similar to ureas, the ligand effect was lost with the introduction of *N*-substitution (**5m**).

**Table 1:**
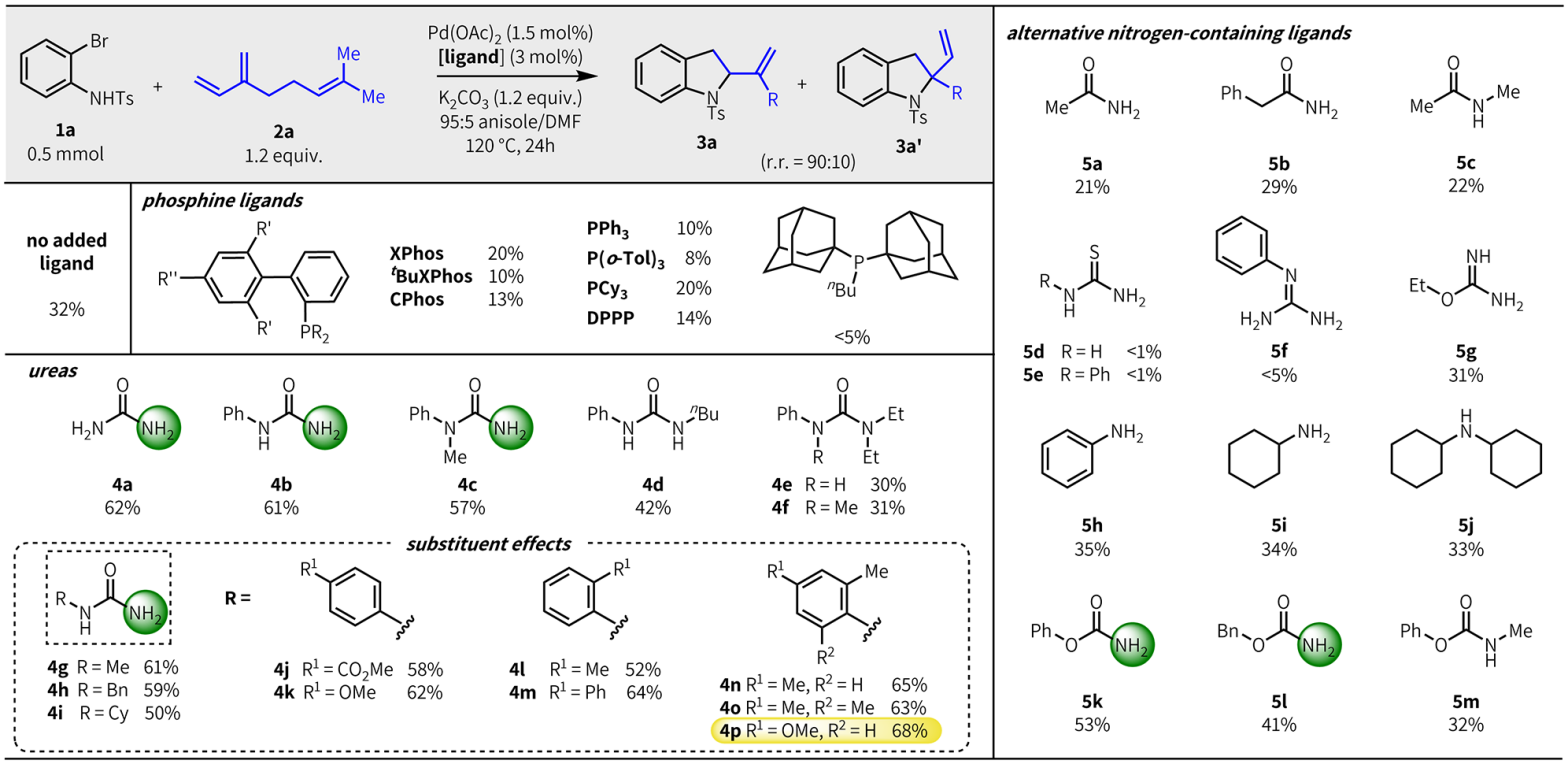
Ligand Structure–Reactivity Relationship Studies^18^

Among the substituted ureas, **4b** afforded the best product yields and so was selected for further structure–reactivity studies. The electronic properties of the aryl group did not affect product yield; ureas bearing unhindered alkyl groups (**4g**,**h**) and electronically modified phenyl ureas (**4j**,**k**) all afforded similar yields to **4b**. While added steric bulk was detrimental in some cases (**4i**, **4l**), product yield improved when the ortho substituent was phenyl (**4m**) or was combined with an electron-donating para substituent (**4n**–**p**), with urea ligand **4p** being optimal. The site selectivity was not affected by the urea ligand structure, but rather the countercation of the base, with potassium being the most site selective.^19^

Next, we explored the generality of our urea-enabled method with various *o*-bromoanilines and 1,3-dienes (Figure 2).^18^ The reaction is insensitive to the electronic properties of the *o-*bromoaniline; substrates bearing electron-withdrawing or electron-donating groups para to either the nitrogen (**3a**–**g**) or bromide (**3h**–**j**) are all effective in the reaction, with yields ranging from 49% to 76%. Remarkably, while prior related heteroannulation methods for the synthesis of indolines have generally not been compatible with substrates bearing substitution adjacent to the halide or nitrogen,^20^ both types of substrates are well-tolerated under our reaction conditions (∼50%, **3k**,**l**). Substitution in these positions has been shown to enhance the biological activity of several indolic therapeutics (e.g., antimalarials, antituberculars).^21^ Substrates bearing various carbonyl functionalities (**3m**–**o**), including tertiary amides, reacted smoothly to afford indolines that closely resemble bioactive alkaloids such as benzastatins.^22^

**Figure 2:**
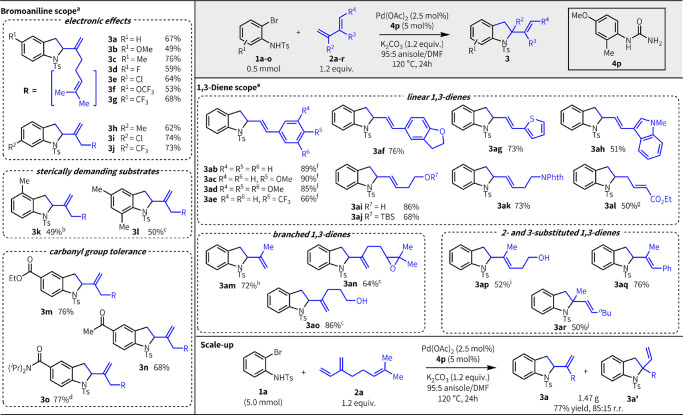
Reaction scope.^18^ Legend: (a) Product ratios of **3/3′** generally 88:12–93:7.^19^ (b) 82:18 r.r. (c) > 99:1 r.r. (d) 5 mol % Pd(OAc)_2_ and 10 mol % **4p** used. (e) Diene scope was run with 1.3 equiv of diene. (f) 1.5 mol % Pd(OAc)_2_ and 3 mol % **4p** used. (g) **2l** added in 2 portions. (h) 97:3 r.r. (i) 1.8:1 *E*/*Z*. (j) 1.0 equiv of *n*-Bu_4_NCl added .

Our urea-enabled heteroannulation methodology also shows a broad scope with respect to the diene. In contrast to prior reports,^11−14^ our method effectively engages structurally and functionally diverse π-coupling partners. Conjugated linear dienes bearing electron-rich aryl groups afford excellent yields of product (**3ab**–**ad**) with just 1.5 mol % Pd. While the yield is lower with electron-deficient groups, reactivity is still good (66%, **3ae**). Conjugated dienes bearing a variety of heterocycles are effective in the reaction (**3af**–**ah**), including potentially coordinating groups such as thiophene (**3ag**). Nonconjugated, heteroatom-containing linear dienes, including unprotected alcohols, also afford indoline products in good yields (**3ai**–**al**).^23^ Likewise, branched dienes, including those bearing sensitive groups such as epoxides, are good substrates under our reaction conditions (**3am**–**ao**). Particularly notable is our method’s tolerance for sterically demanding 2- and 3-substituted dienes (**3ap**–**ar**); such branching in the π-coupling partner has not previously been demonstrated in related transformations.^11,13^ The primary limitation of our method is with internal dienes, which are unreactive under our current conditions.^19^ The reaction scales readily; in fact, when performed at a 10-fold increase in scale, the product yield improved (77% at 5 mmol vs 68% at 0.5 mmol), allowing us to isolate ∼1.5 g of indoline products (**3a/3a′** = 85:15).

Having established the beneficial effect of urea pro-ligands in the heteroannulation reaction, we next focused our investigations on the nature of Pd–urea coordination, as experimental data on binding of urea ligands to Pd is limited.^24^ Specifically, to the best of our knowledge, Pd–urea binding under basic conditions or binding of monosubstituted ureas to Pd has not been investigated. It is essential to bridge this gap to better guide the future design of ureate ligands for late transition metal catalysis. With most known coordination complexes of urea/ureate with transition metals, urea coordinates through oxygen; examples of monodentate, *N*-bound urea complexes are rare.^16,24^ In the only examples of Pd–urea catalysis under basic conditions, it was hypothesized that upon urea deprotonation, the resulting ureate coordinates through both N and O, similar to ureate complexes with early transition metals,^7^ although no studies were undertaken to test this hypothesis.^8,9^ On the basis of our empirical ligand structure–reactivity studies, we proposed an alternative model where the ureate binds in a monodentate fashion through N. We undertook a series of experimental and computational studies to distinguish between these and other potential ureate binding modes.

First, we investigated the binding of monosubstituted ureas to Pd. We prepared stable coordination complex **6** from phenylurea **4b** and PdCl_2_, which was isolated as an analytically pure yellow solid in 77% yield (Figure 3a). Complex **6** is a competent precatalyst for the reaction, affording **3a/3a′** in 56% yield; likewise, PdCl_2_ in the presence of **4b** gave 45% yield. Since no crystals suitable for X-ray analysis could be obtained, we assigned the structure of **6** using infrared (IR) and Raman spectroscopy. The IR spectrum showed significant lowering of vibrational frequencies associated with the −NH_2_ relative to free **4b**, with negligible changes to the −NHPh frequencies, and an increase in the C=O stretching frequency (+50 cm^–1^).^19^ Through ^1^H NMR, we observed rapid deuterium exchange preferentially at the −NH_2_ group upon coordination to Pd, with complete loss of the peak corresponding to those protons (Figure 3a). These data are consistent with monodentate Pd–urea binding through the −NH_2_ group.

**Figure 3:**
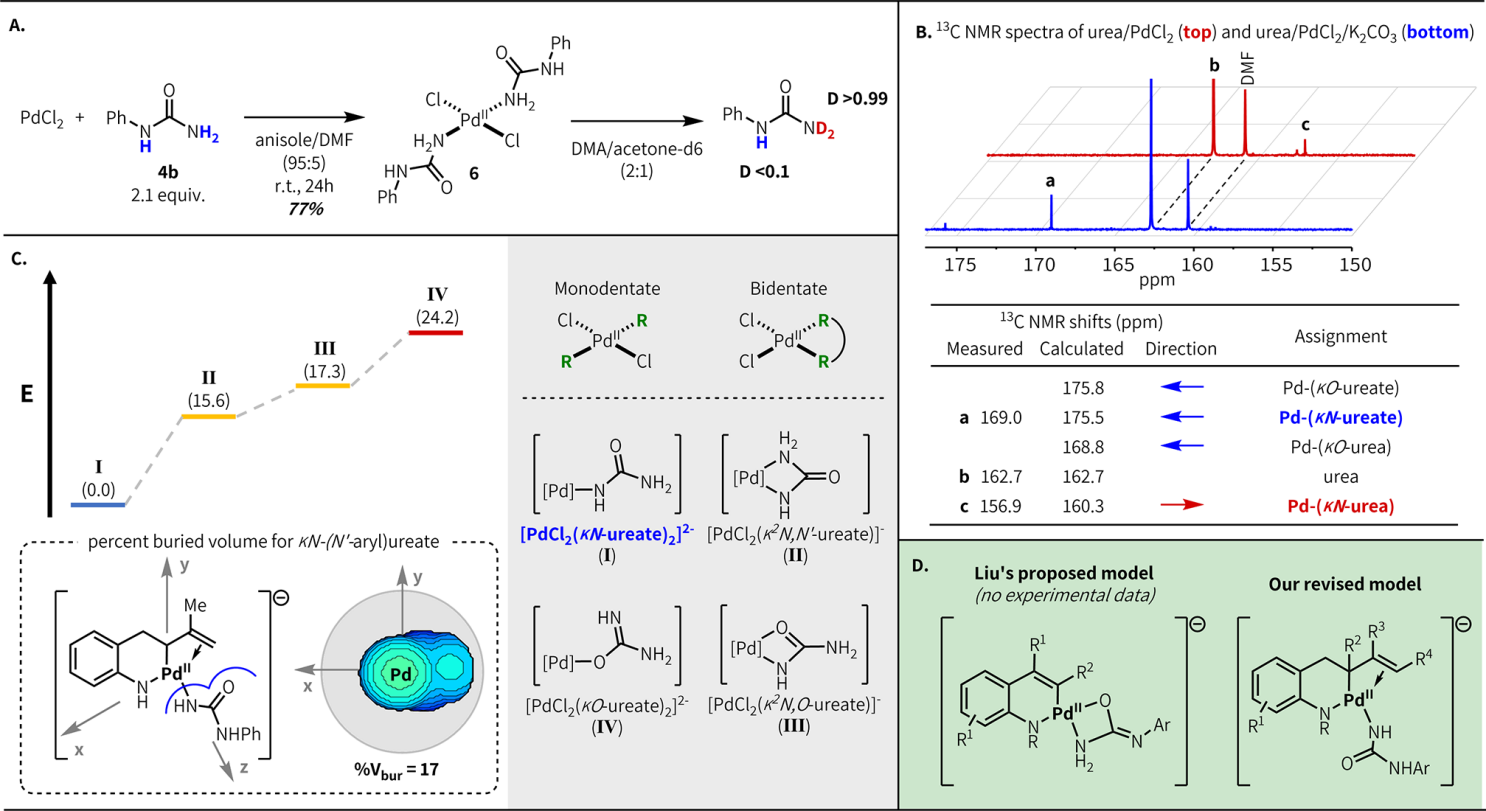
Pd–urea and Pd–ureate coordination studies. (A) Synthesis of model monosubstituted Pd–urea complex and deuteration study. (B) Solution-state NMR analysis of Pd–urea and Pd–ureate complexes; calculated values adjusted to experimental NMR shift of urea. (C) Computational studies; values in parentheses are Gibbs free energies in kcal/mol.^19,25,28^ (D) Original proposed Pd–ureate coordination model and revised model.

Next, we examined Pd–urea binding in solution to better reflect the potential coordination dynamics that may be operative under the reaction conditions. ^13^C NMR solution spectroscopy and computational studies were used to ascertain the nature of Pd–urea and Pd–ureate coordination (Figure 3b). A prior solution-state study showed binding of urea to Pd(en)(H_2_O)^2+^ cation through either O or N in acetone, with slight preference for the former (1.6 kcal/mol).^24b^ This experimentally measured ratio was used to identify an appropriate functional and basis set for our calculations (see below).^25^ While the ^13^C NMR spectrum of ^13^C-urea in a D_2_O solution of K_2_PdCl_4_ showed two new resonances corresponding to *O*-bound urea and *N*-bound urea in 1:2 ratio,^19^ no *O*-bound urea species were detected in an acetone-*d*_6_/dimethylformamide (DMF) (1:2) solution of PdCl_2_. When K_2_CO_3_ was added as a base, a new species was detected at 169.0 ppm (cf. 162.7 ppm for free urea). Under neutral conditions, the downfield NMR shift of the carbonyl peak has been assigned to the *O*-bound urea.^24b^ Equivalent data are not available for the deprotonated ureate ligand, so we used the gauge-independent atomic orbital method to calculate NMR shielding tensors for the plausible ureate-PdCl_2_ complexes.^26^ Since an equivalent downfield shift was predicted for both *N*- and *O*-bound ureate, we could not determine the binding mode of the downfield species observed under basic conditions using NMR. However, our calculations show that monodentate binding of two ureate ligands through N is strongly favored relative to both *O*-binding and bidentate *N,O*-binding (+24 and +17 kcal/mol, respectively) (Figure 3c).

These results, taken together with our empirical observations of the need for a free −NH_2_ in the urea pro-ligand, are consistent with our hypothesis that *N*-arylureas act as monodentate *N*-bound ureate ligands under our reaction conditions, coordinating through the nonsubstituted nitrogen (Figure 3d).^27^ Buried volume calculations on both the model Pd-ureate complex and the proposed catalytic intermediate indicate that the steric demand of ureates is considerably lower than that of phosphines (%*V*_bur_ = 17 vs 24 to >50).^28^ Future mechanistic studies will investigate potential changes in coordination during catalysis and further elucidate the origin of the observed ligand effect.

The development of ureas as sterically undemanding pro-ligands for Pd has enabled a general method for the heteroannulation of *N*-tosyl-*o*-bromoanilines and 1,3-dienes. Our method displays a broad substrate scope in both coupling partners, including sterically demanding substrates and those bearing sensitive functionality. Moreover, by using low loadings of reagents, only a slight excess of diene, and environmentally benign anisole as the predominant solvent, we reduce the environmental impact of this transformation.^29^ The general reactivity, combined with the ready scalability of the reaction and the attractive practical features of ureate ligands, makes this method amenable for the convergent synthesis of 2-substituted indolines. We anticipate that the reactivity enhancement achieved with ureate ligands will be applicable to a broader range of late transition metal-catalyzed reactions.

## Supporting Information Available

Additional experimental details, experimental procedures, computational studies, compound characterization, and NMR spectra for all new compounds (PDF)
