# Supplementary material for: Pd-Catalyzed Heteroannulation Using N-Arylureas as a Sterically Undemanding Ligand Platform
Source: J Am Chem Soc. 2022 Apr 5;144(15):6667–73. doi: 10.1021/jacs.2c01019 (PMC9026275; doi:10.1021/jacs.2c01019)

Pd-Catalyzed Heteroannulation using *N*-Arylureas as a Sterically-Undemanding Ligand Platform

Jakub Vaith, Dasha Rodina, Gregory C. Spaulding, Shauna M. Paradine*

Department of Chemistry, University of Rochester, 414 Hutchison Hall, 120 Trustee Road, Rochester, NY 14627 (USA).

*Corresponding Author: sparadin@ur.rochester.edu

Supporting Information

Contents

General Information 2

Methods 2

Materials and Reagents 3

Instrumentation 3

Abbreviations Used 3

Preparation of *N*-Tosyl *o*-Bromoanilines 4

Preparation of 1,3-Dienes 10

Preparation of Ureas 17

Ligand Structure-reactivity Relationship Studies 20

Reaction Condition Optimization 31

Table S1. Reaction optimization studies. 31

Reaction Time Course Experiment 36

Table S2. Kinetic comparison Studies. 36

Reaction Scope 37

Bromoaniline Scope 38

1,3-Diene Scope 45

Method Limitations 53

Table S3. Unsuccessful substrates. 53

Gram Scale Reaction 54
Model *N*-phenylurea-$PdCl_2$ complex 55
Table S4. Assignment of the relevant bands in the IR spectra (units = cm^{-1}).[33] 56
Figure S1. Infrared spectra of urea 4b (top) and complex 6 (bottom). 56
Figure S2. Raman spectrum of complex 6. 57
Figure S3. Close-up of ^{1}H NMR spectra of urea 4b (top) and complex 6 (bottom) in 2:1 DMA/acetone-*d*6. 58
Figure S4. ^{1}H NMR spectra of urea 4b (top) and complex 6 (bottom) in 2:1 DMA/acetone-*d*6. 59
Table S5. Comparison of competence of complex 6 as precatalyst. 60
^{13}C NMR urea-Pd binding studies 61
Figure S5. ^{13}C NMR spectra of samples A-D 62
Computational studies 63
Computational level benchmarking 63
Table S6. Results of benchmarking of the functional, basis set, and solvation model on a prior solution-state study of urea binding of urea to $Pd(en)(H2O)^{2+}$ 63
Comparison of urea-Pd binding modes 65
Figure S6. Comparison of urea-Pd binding modes 65
Comparison of ureate-Pd binding modes 67
Figure S7. Comparison of ureate-Pd binding modes 67
NMR shielding tensor calculations using Gauge-Independent Atomic Orbital (GIAO) method 71
Percent Buried Volume (%Vbur) calculations 71
Characterization of $Pd(OAc)_2$ precatalyst 74
Figure S8. ^{1}H NMR spectrum of $Pd(OAc)_2$ in $CDCl_3$. 75
References Cited 75
NMR Spectra of New Compounds 79

General Information

Methods

All reactions were carried out under a nitrogen atmosphere in flame–dried glassware with magnetic stir bar unless otherwise specified. Stainless steel gas-tight syringes were used to

transfer air- and moisture-sensitive liquids. Reactions were monitored by thin–layer chromatography (TLC) on pre–coated silica gel 60 F254 glass–supported plates from EMD, and visualized under UV light (254 nm) or with p–anisaldehyde followed by heating. Flash chromatography was performed using SiliaFlash P60 (230–400 mesh, SiliCycle). Reported product yields were determined based on material isolated after column purification.

Materials and Reagents

Reagents were used as obtained from commercial suppliers without further purification unless otherwise noted. $Pd(OAc)_2$ was purchased from Strem; see S74-75 for certificate of analysis and characterization of impurities. Reaction solvents – Tetrahydrofuran (THF), diethyl ether (Et_2O), dichloromethane (DCM) – were purchased from Fisher and dried by passing through columns of activated alumina (Pure Process Technology SPS). Pyridine (Fisher), acetonitrile (Fisher), *N,N*-dimethylacetamide (DMA), toluene (Fisher) and anisole (Alfa Aesar) were used without further purification. *N,N*-Dimethylformamide (Fisher) was stored over 3A molecular sieves. Deuterated solvents $CDCl_3$, DMSO-d_6, acetone-d_6 (Cambridge Isotope Laboratories), and HPLC solvents (Fisher) were used without further purification. ^{13}C-Urea (99% ^{13}C) was purchased from Aldrich. Synthesized dienes were stored at –30 °C; some decomposed over time and were used soon after isolation (see Preparation of 1,3-Dienes section for storage conditions of specific substrates).

Instrumentation

Proton nuclear magnetic resonance (^{1}H NMR) and proton-decoupled carbon nuclear magnetic resonance (^{13}C{^{1}H} NMR) spectra were recorded on a Bruker DPX–400 instrument (operating at 400 MHz for ^{1}H, 100 MHz for ^{13}C) or a Bruker DPX–500 instrument (operating at 500 MHz for ^{1}H, 125 MHz for ^{13}C) at ambient temperature. Proton resonances are referenced to residual protium in the NMR solvent. Carbon resonances are referenced to the carbon resonances of the NMR solvent. Data are represented as follows: chemical shift, multiplicity (br = broad, s = singlet, d = doublet, t = triplet, q = quartet, m = multiplet, app = apparent), coupling constants (J) in Hertz (Hz), integration. Infrared (IR) spectra were obtained using a Shimadzu IRAffinity-1 FTIR spectrometer equipped with an attenuated total reflectance (ATR) single reflection unit. Mass spectral (MS) data were obtained on a Thermo Fisher Q Exactive Plus spectrometer (University of Rochester Medical Center Mass Spectrometry Resource Laboratory). Microanalysis samples were weighed with a PerkinElmer Model AD6000 Autobalance and their compositions were determined with a PerkinElmer 2400 Series II Analyzer. High-performance liquid chromatography (HPLC) analysis was performed using a Shimadzu Prominence–I LC–2030 Plus system with commercially available Restek Pinnacle DB Cyano column (5 µm, 150x4.6 mm). All samples were eluted with 2% ^{i}PrOH/hexanes at an elution rate of 1 mL/min and detected at 254 nm. Total run time was 25 min.

Abbreviations Used

aq. = aqueous, cm^{-1} = wavenumber, DCM = dichloromethane, DMA = *N,N*-dimethylacetamide, DMF = *N,N*-dimethylformamide, DMSO = dimethylsulfoxide, equiv. = equivalents, h = hours, min = minutes, m/z = mass to charge ratio, r.r. = regioisomeric ratio, rt = room temperature, sat = saturated, TBACl = tetrabutylammonium chloride, THF = tetrahydrofuran, TBS = *tert*-butyldimethylsilyl, Ts = toluenesulfonyl.

Preparation of *N*-Tosyl *o*-Bromoanilines

General procedure for synthesis of *N*-tosyl *o*-bromoanilines: Prepared according to a modified literature procedure.[1] To a solution of corresponding *o*-bromoaniline (1.0 equiv.) in pyridine (ca 1.0M) was added *p*-toluenesulfonyl chloride (1.0 equiv.) and the reaction mixture was stirred overnight at rt. Water (5-10 mL per mL of pyridine) was then added to the reaction mixture, resulting in precipitation of crude product. This mixture was filtered, then the solid residue was washed with water and recrystallized in refluxing EtOH/hexanes to afford the pure product.

_N_-(2-Bromophenyl)-4-methylbenzenesulfonamide (1a)

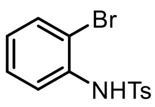

Prepared according to general procedure. 2-Bromoaniline (5.55 g, 32.3 mmol, 1.0 equiv.), pyridine (25 mL, 1.29M), and *p*-toluenesulfonyl chloride (6.16 g, 32.3 mmol, 1.0 equiv.) were used. The recrystallized product was obtained as a white crystalline solid (8.63 g, 26.5 mmol, 82%).

^{1}H NMR (400 MHz, $CDCl_3$) δ 7.69–7.65 (m, 3H), 7.42 (d, *J* = 8.0 Hz, 1H), 7.28 (dd, *J* = 8.0, 7.6 Hz, 1H), 7.22 (d, *J* = 8.4 Hz, 2H), 7.00–6.96 (m, 2H), 2.38 (s, 3H). Spectral data agree with that reported in the literature.[2]

_N_-(2-Bromophenyl-4-methoxyphenyl)-4-methylbenzenesulfonamide (1b)

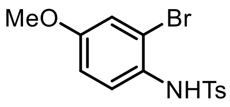

Prepared according to general procedure. 2-Bromo-4-methoxyaniline (1.00 g, 4.95 mmol, 1.0 equiv.), pyridine (5.0 mL, 0.99M), and *p*-toluenesulfonyl chloride (0.943 g, 4.95 mmol, 1.0 equiv.) were used. The recrystallized product was obtained as a white crystalline solid (1.24 g, 3.48 mmol, 70%).

^{1}H NMR (400 MHz, $CDCl_3$) δ 7.59 (d, *J* = 9.2 Hz, 1H), 7.56 (d, *J* = 8.0 Hz, 2H), 7.19 (d, *J* = 8.0 Hz, 2H), 6.93 (d, *J* = 2.8 Hz, 1H), 6.84 (dd, *J* = 9.2, 2.8 Hz, 1H), 6.62 (s, 1H), 3.75 (s, 3H), 2.38 (s, 3H). Spectral data agree with that reported in the literature.[2]

_N_-(2-Bromophenyl-4-methylphenyl)-4-methylbenzenesulfonamide (1c)

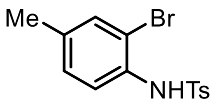

Prepared according to general procedure. 2-Bromo-4-methylaniline (1.50 g, 8.06 mmol, 1.0 equiv.), pyridine (10 mL, 0.81M), and *p*-toluenesulfonyl chloride (1.54 g, 8.06 mmol, 1.0 equiv.) were used. The recrystallized product was obtained as a white crystalline solid (1.55 g, 4.56 mmol, 57%).

^{1}H NMR (400 MHz, $CDCl_3$) δ 7.62 (d, *J* = 8.4 Hz, 2H), 7.55 (d, *J* = 8.0 Hz, 1H), 7.21–7.19 (m, 3H), 7.07 (d, *J* = 8.0 Hz, 1H), 6.82 (s, 1H), 2.37 (s, 3H), 2.25 (s, 3H). Spectral data agree with that reported in the literature.[3]

_N_-(2-Bromophenyl-4-fluorophenyl)-4-methylbenzenesulfonamide (1d)

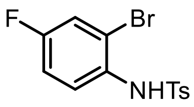

Prepared according to general procedure. 2-Bromo-4-fluoroaniline (3.00 g, 15.8 mmol, 1.0 equiv.), pyridine (15 mL, 1.05M), and *p*-toluenesulfonyl chloride (3.01 g, 15.8 mmol, 1.0 equiv.) were used. The recrystallized product was obtained as a white crystalline solid (3.96 g, 11.5 mmol, 73%).

^{1}H NMR (400 MHz, $CDCl_3$) δ 7.67 (dd, *J* = 9.2, 5.2 Hz, 1H), 7.59 (d, *J* = 8.0 Hz, 2H), 7.21 (d, *J* = 8.0 Hz, 2H), 7.15 (dd, *J* = 7.6, 2.8 Hz, 1H), 7.02 (ddd, *J* = 9.2, 8.0, 2.8 Hz, 1H), 6.79 (s, 1H), 2.39 (s, 3H). Spectral data agree with that reported in the literature.[2]

N-(2-Bromophenyl-4-chlorophenyl)-4-methylbenzenesulfonamide (1e)

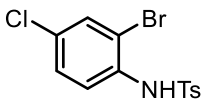

Prepared according to general procedure. 2-Bromo-4-chloroaniline (3.52 g, 9.76 mmol, 1.0 equiv.), pyridine (10 mL, 0.98M), and *p*-toluenesulfonyl chloride (1.86 g, 9.76 mmol, 1.0 equiv.) were used. The recrystallized product was obtained as a white crystalline solid (1.92 g, 5.58 mmol, 53%).

^{1}H NMR (400 MHz, $CDCl_3$) δ 7.62–7.58 (m, 3H), 7.39 (d, *J* = 2.2 Hz, 1H), 7.25–7.21 (m, 3H), 6.87 (s, 1H), 2.37 (s, 3H). Spectral data agree with that reported in the literature.[3]

N-(2-Bromophenyl-4-(trifluoromethoxy)phenyl)-4-methylbenzenesulfonamide (1f)

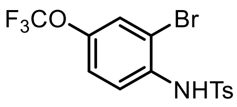

Prepared according to general procedure. 2-Bromo-4-(trifluoromethoxy)aniline (1.50 g, 5.86 mmol, 1.0 equiv.), pyridine (5.0 mL, 1.17M), and *p*-toluenesulfonyl chloride (1.17 g, 5.86 mmol, 1.0 equiv.) were used. The recrystallized product was obtained as a white crystalline solid (1.59 g, 3.88 mmol, 66%).

^{1}H NMR (400 MHz, $CDCl_3$) δ 7.70 (d, *J* = 8.8 Hz, 1H), 7.65 (d, *J* = 8.4 Hz, 2H), 7.31 (d, *J* = 0.6 Hz, 1H), 7.24 (d, *J* = 8.4 Hz, 2H), 7.16 (dd, *J* = 8.8, 0.6 Hz, 1H), 6.94 (s, 1H), 2.40 (s, 3H).

^{13}C NMR (125 MHz, $CDCl_3$) δ 146.0, 144.7, 135.8, 133.8, 129.9, 127.4, 125.4, 123.4, 121.3, 120.4 (q, $J_{C\text{-}F}$ = 258 Hz), 115.9, 21.7.

HRMS (ESI) m/z calculated for $C_{14}H_{12}BrF_3NO_3S$ $[M+H]^+$: 409.9673, found 409.9662.

N-(2-Bromophenyl-4-(trifluoromethyl)phenyl)-4-methylbenzenesulfonamide (1g)

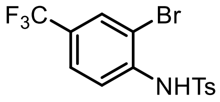

Prepared according to general procedure. 2-Bromo-4-(trifluoromethyl)aniline (2.51 g, 10.5 mmol, 1.0 equiv.), pyridine (10 mL, 1.05M), and *p*-toluenesulfonyl chloride (2.00 g, 10.5 mmol, 1.0 equiv.) were used. The recrystallized product was obtained as a white crystalline solid (2.65 g, 6.73 mmol, 64%).

^{1}H NMR (400 MHz, $CDCl_3$) δ 7.76–7.68 (m, 4H), 7.51 (d, *J* = 8.8 Hz, 1H), 7.27 (d, *J* = 6.8 Hz, 2H), 7.22 (s, 1H), 2.40 (s, 3H). Spectral data agree with that reported in the literature.[2]

N-(2-Bromophenyl-5-methylphenyl)-4-methylbenzenesulfonamide (1h)

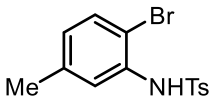

Prepared according to general procedure. 2-Bromo-5-methylaniline (1.50 g, 8.06 mmol, 1.0 equiv.), pyridine (10 mL, 0.81M), and *p*-toluenesulfonyl chloride (1.54 g, 8.06 mmol, 1.0 equiv.) were used. The recrystallized product was obtained as an orange crystalline solid (1.85 g, 5.44 mmol, 67%).

^{1}H NMR (400 MHz, $CDCl_3$) δ 7.63 (d, *J* = 8.4 Hz, 2H), 7.48 (d, *J* = 1.2 Hz, 1H), 7.24 (d, *J* = 8.2 Hz, 1H), 7.21–7.16 (m, 2H), 6.93 (s, 1H), 6.75 (dd, *J* = 8.2, 1.2 Hz, 1H), 2.35 (s, 3H), 2.28 (s, 3H).

^{13}C NMR (125 MHz, $CDCl_3$) δ 144.2, 136.9, 136.0, 132.9, 132.1, 129.7, 129.4, 127.5, 123.2, 116.1, 21.7, 20.7.

HRMS (ESI) m/z calculated for $C_{14}H_{15}BrNO_2S$ $[M+H]^+$: 340.0007, found 399.9998.

N-(2-Bromophenyl-5-chlorophenyl)-4-methylbenzenesulfonamide (1i)

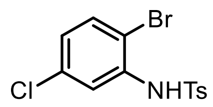

Prepared according to general procedure. 2-Bromo-5-chloroaniline (1.50 g, 7.26 mmol, 1.0 equiv.), pyridine (8.0 mL, 0.91M), and *p*-toluenesulfonyl chloride (1.38 g, 7.26 mmol, 1.0 equiv.) were used. The recrystallized product was obtained as a white crystalline solid (1.41 g, 3.91 mmol, 54%).

^{1}H NMR (400 MHz, $CDCl_3$) δ 7.70-7.66 (m, 3H), 7.33 (d, *J* = 8.6 Hz, 1H), 7.25 (d, *J* = 8.4 Hz, 2H), 6.99 (s, 1H), 6.94 (dd, *J* = 8.6, 2.3 Hz, 1H), 2.39 (s, 3H).

^{13}C NMR (100 MHz, $CDCl_3$) δ 144.7, 135.9, 135.7, 134.6, 133.4, 130.0, 127.5, 126.3, 122.0, 113.1, 21.7.

HRMS (ESI) m/z calculated for $C_{13}H_{12}BrClNO_2S$ $[M+H]^+$: 359.9461, found 359.9452.

N-(2-Bromophenyl-5-(trifluoromethyl)phenyl)-4-methylbenzenesulfonamide (1j)

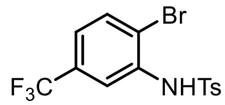

Prepared according to general procedure. 2-Bromo-5-(trifluoromethyl)aniline (2.51 g, 10.5 mmol, 1.0 equiv.), pyridine (10 mL, 1.05M), and *p*-toluenesulfonyl chloride (2.06 g, 10.5 mmol, 1.0 equiv.) were used. The recrystallized product was obtained as a white crystalline solid (2.71 g, 6.88 mmol, 66%).

^{1}H NMR (500 MHz, $CDCl_3$) δ 7.92 (d, *J* = 1.2 Hz, 1H), 7.68 (d, *J* = 8.2 Hz, 2H), 7.55 (d, *J* = 8.2 Hz, 1H), 7.25 (d, *J* = 8.2 Hz, 2H), 7.20 (dd, *J* = 8.2, 1.2 Hz, 1H), 7.14 (br, 1H), 2.39 (s, 3H).

^{13}C NMR (125 MHz, $CDCl_3$) δ 144.9, 135.7, 135.6, 133.4, 131.3 (q, $J_{C\text{-}F}$ = 33.5 Hz), 130.0, 127.5, 123.4 (q, $J_{C\text{-}F}$ = 273.6 Hz), 122.5 (q, $J_{C\text{-}F}$ = 3.5 Hz), 118.7 (q, $J_{C\text{-}F}$ = 3.8 Hz), 21.7.

HRMS (ESI) m/z calculated for $C_{14}H_{12}BrF_3NO_2S$ $[M+H]^+$: 393.9724, found 393.9719.

N-(2-Bromophenyl-4,6-dimethylphenyl)-4-methylbenzenesulfonamide (1k)

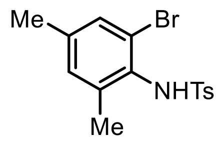

Prepared according to general procedure. 2-Bromo-4,6-dimethylaniline (1.50 g, 7.50 mmol, 1.0 equiv.), pyridine (8.0 mL, 0.94M), and *p*-toluenesulfonyl chloride (1.43 g, 7.50 mmol, 1.0 equiv.) were used. The recrystallized product was obtained as a white crystalline solid (1.48 g, 4.18 mmol, 56%).

^{1}H NMR (500 MHz, $CDCl_3$) δ 7.56 (d, *J* = 8.1 Hz, 2H), 7.21 (d, *J* = 8.1 Hz, 2H), 7.08 (s, 1H), 7.02 (s, 1H), 6.23 (s, 1H), 2.47 (s, 3H), 2.41 (s, 3H), 2.25 (s, 3H).

^{13}C NMR (125 MHz, $CDCl_3$) δ 143.9, 140.3, 139.0, 137.1, 131.8, 130.9, 130.3, 129.6, 127.8, 123.3, 21.7, 20.7, 20.3.

HRMS (ESI) m/z calculated for $C_{15}H_{17}BrNO_2S$ $[M+H]^+$: 354.0163, found 354.0151.

N-(2-Bromophenyl-3-methylphenyl)-4-methylbenzenesulfonamide (1l)

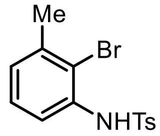

Prepared according to general procedure. 2-Bromo-3-methylaniline (1.00 g, 5.56 mmol, 1.0 equiv.), pyridine (5.0 mL, 1.11M), and *p*-toluenesulfonyl chloride (1.06 g, 5.56 mmol, 1.0 equiv.) were used. The recrystallized product was obtained as a white crystalline solid (0.483 g, 1.42 mmol, 25%).

^{1}H NMR (400 MHz, $CDCl_3$) δ 7.66 (d, *J* = 8.4 Hz, 2H), 7.49 (d, *J* = 8.0 Hz, 1H), 7.21 (d, *J* = 8.4 Hz, 2H), 7.14 (dd, *J* = 8.0, 7.4 Hz, 1H), 7.10 (s, 1H), 6.96 (d, *J* = 7.4 Hz, 1H), 2.36 (s, 3H), 2.31 (s, 3H).

^{13}C NMR (125 MHz, $CDCl_3$) δ 144.2, 139.1, 136.0, 134.9, 129.7, 127.8, 127.5, 127.1, 119.4, 118.2, 23.9, 21.7.

HRMS (ESI) m/z calculated for $C_{14}H_{15}BrNO_2S$ $[M+H]^+$: 340.0007, found 399.9996.

Preparation of ethyl 3-bromo-4-((4-methylphenyl)sulfonamido)benzoate (1m)

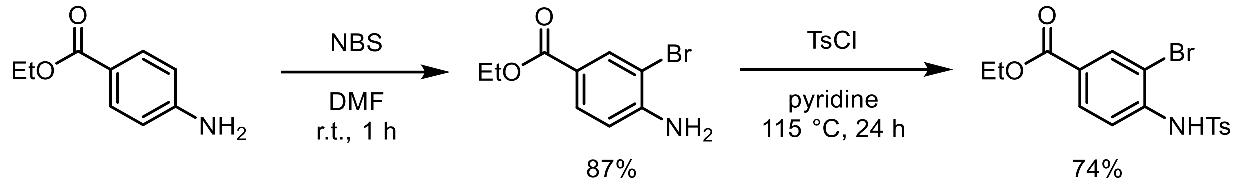

Ethyl 4-amino-3-bromobenzoate

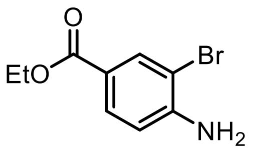

Prepared according to a modified literature procedure.[4] To a solution of benzocaine (4.95 g, 30.0 mmol, 1.0 equiv.) in DMF (60 mL, 0.5M) at 0 °C was added *N*-bromosuccinimide (5.34, 30.0 mmol, 1.0 equiv.). The reaction mixture was warmed up to rt and stirred for 1 h. Upon completion, water (400 mL) was added resulting in immediate formation of a white precipitate. Filtration of the suspension and wash of the precipitate with water afforded the product as a white solid (6.27 g, 26.1 mmol, 87%).

^{1}H NMR (400 MHz, $CDCl_3$) δ 8.12 (d, *J* = 1.7 Hz, 1H), 7.79 (dd, *J* = 8.4, 1.7 Hz, 1H), 6.73 (d, *J* = 8.4 Hz, 1H), 4.32 (q, *J* = 7.1 Hz, 2H), 1.36 (t, *J* = 7.1 Hz, 3H). Spectral data agree with that reported in the literature.[4]

Ethyl 3-bromo-4-((4-methylphenyl)sulfonamido)benzoate (1m)

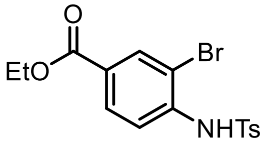

Prepared according to modified general procedure. To a solution of ethyl 4-amino-3-bromobenzoate (6.15 g, 25.2 mmol, 1.0 equiv.) in pyridine (20 mL, 1.26M) at rt was added *p*-toluenesulfonyl chloride (4.80 g, 25.2 mmol, 1.0 equiv.). The reaction mixture was heated to 115 ºC for 24 h. Upon completion, the reaction mixture was cooled down to rt, water (100 mL) was added, and the reaction mixture was stirred for 1 h resulting in precipitation of the crude product. This mixture was filtered, then the solid residue was washed with water and recrystallized in refluxing EtOH/hexanes to afford the pure product as a white crystalline solid (7.43 g, 18.65 mmol, 74%).

^{1}H NMR (400 MHz, $CDCl_3$) δ 8.11 (d, *J*=1.5 Hz, 1H), 7.91 (dd, *J* = 8.5, 1.5 Hz, 1H), 7.73–7.66 (m, 3H), 7.24 (d, *J* = 7.6 Hz, 2H), 4.33 (q, *J* = 7.1 Hz, 2H), 2.38 (s, 3H), 1.36 (t, *J* = 7.1 Hz, 3H).

^{13}C NMR (125 MHz, DMSO-d6) δ 163.9, 143.6, 139.6, 137.2, 133.6, 129.8, 129.1, 128.1, 126.8, 125.2, 117.7, 61.1, 21.0, 14.0.

HRMS (ESI) m/z calculated for $C_{16}H_{17}BrNO_4S$ $[M+H]^+$: 398.0062, found 398.0061.

Preparation of *N*-(4-acetyl-2-bromophenyl)-4-methylbenzenesulfonamide (1n)

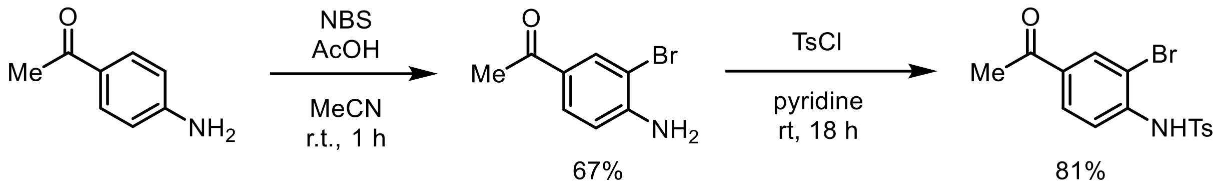

1-(4-Amino-3-bromophenyl)ethan-1-one

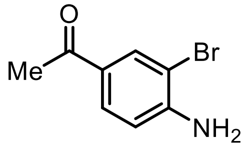

Prepared according to a modified literature procedure.[4] To a solution of 4-aminoacetophenone (4.00 g, 29.6 mmol, 1.0 equiv.) in MeCN/AcOH (40 mL, 3:1, 0.74M) at 0 °C was added dropwise (over 30 minutes) a solution of *N*-bromosuccinimide (5.00, 28.1 mmol, 0.95 equiv.) in MeCN (20 mL, 1.41M). The reaction mixture was warmed up to rt and stirred for 18 h. Upon completion, brine (100 mL) and ethyl acetate (100 mL) were added to the reaction mixture. The organic layer was separated, washed with sat. $NaHCO_3$ solution, and then washed with brine. Removal of volatiles under reduced pressure afforded the crude product as an off-white solid. The crude product was purified by flash column chromatography (silica, hexanes/ethyl acetate 2:1) to obtain the product as a white solid (4.25 g, 19.9 mmol, 67%).

^{1}H NMR (400 MHz, $CDCl_3$) δ 8.02 (s, 1H), 7.69 (d, *J* = 8.4 Hz, 1H), 6.72 (d, *J* = 8.4 Hz, 1H), 4.62 (s, 2H), 2.47 (s, 3H). Spectral data agree with that reported in the literature.[5]

***N*-(4-Acetyl-2-bromophenyl)-4-methylbenzenesulfonamide (1n)**

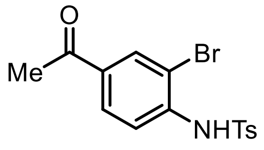

Prepares according to general procedure. 1-(4-Amino-3-bromophenyl)ethan-1-one (4.23 g, 19.8 mmol, 1.0 equiv.), pyridine (10 mL, 1.98M), and *p*-toluenesulfonyl chloride (3.78 g, 19.8 mmol, 1.0 equiv.) were used. The recrystallized product was obtained as a white crystalline solid (5.94 g, 16.1 mmol, 81%).

^{1}H NMR (500 MHz, DMSO-d6) δ 8.00 (d, *J* = 2.4 Hz, 1H), 7.77 (dd, *J* = 8.6, 2.0 Hz, 1H), 7.68 (d, *J* = 8.3 Hz, 2H), 7.65 (d, *J* = 8.6 Hz, 1H), 7.21 (d, *J* = 8.5 Hz, 2H), 2.48 (s, 3H), 2.34 (s, 3H). ^{13}C NMR (125 MHz, DMSO-d6) δ 195.5, 144.9, 139.0, 135.7, 134.2, 133.0, 130.0, 129.0, 127.4, 119.8, 114.4, 77.2, 26.5, 21.7.

HRMS (ESI) m/z calculated for $C_{15}H_{15}BrNO_3S$ $[M+H]^+$: 367.9956, found 367.9953.

Preparation of 3-bromo-*N,N*-diisopropyl-4-((4-methylphenyl)sulfonamido)benzamide (1o)

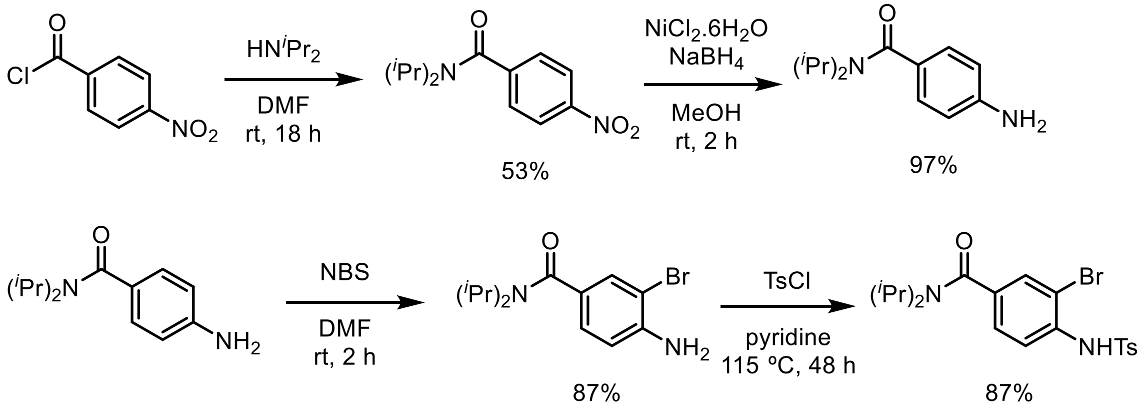

N,N-Diisopropyl-4-nitrobenzamide

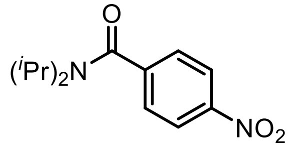

Prepared according to a modified literature procedure.[6] To a solution of 4-nitrobenzoyl chloride (4.63 g, 25.0 mmol, 1.0 equiv.) in DMF (20 mL, 1.25M) was added *N,N*-diisopropylamine (5.00 mL, 68.9 mmol, 2.76 equiv.). The reaction mixture was stirred at rt for 18 h. Water (100 mL) was then added to the reaction mixture resulting in precipitation of crude product which was collected by filtration under reduced pressure and washed with water. The crude product was redissolved in EtOAc, washed with 1M NaOH solution, and washed with brine. The organic layer was dried over Na_2SO_4 and the volatiles were removed under reduced pressure to afford the product as a yellow solid (3.31 g, 13.3 mmol, 53%).

^{1}H NMR (400 MHz, DMSO-d6) δ 8.27 (d, *J* = 8.5 Hz, 1H), 7.57 (d, *J* = 8.5 Hz, 1H), 3.60–3.55 (m, 1H), 1.47–1.42 (m, 3H), 1.13–1.08 (m, 3H). Spectral data agree with that reported in the literature.[6]

4-Amino-N,N-diisopropylbenzamide

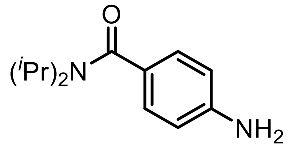

Prepared according to a modified literature procedure.[7] To a solution of *N,N*-diisopropyl-4-nitrobenzamide (1.40 g, 5.60 mmol, 1.0 equiv.) and $NiCl_2 \cdot 6H_2O$ (2.66 g, 11.2 mmol, 2.0 equiv.) in MeOH (25 mL, 0.22M) at 0 °C was added portionwise (CAUTION: vigorous gas evolution) sodium borohydride (640 mg, 16.8 mmol, 3.0 equiv.). The reaction mixture was warmed up to rt and stirred for 2 h. Then, it was cooled down to 0 °C and another portion of sodium borohydride (320 mg, 8.40 mmol, 1.5 equiv.) was cautiously added. The reaction mixture was warmed up to rt and stirred for additional 30 min. The reaction was quenched with aq. NH_3 (30% solution, 10 mL). Water (100 mL) and EtOAc (100 mL) were added, the organic layer was separated, dried over Na_2SO_4, filtered through celite, and the volatiles were removed under reduced pressure to afford the product as a white solid (1.20 g, 5.43 mmol, 97%).

^{1}H NMR (400 MHz, $CDCl_3$) δ 7.14 (d, *J* = 7.0 Hz, 2H), 6.61 (d, *J* = 7.0 Hz, 2H), 3.90–3.55 (m, 4H), 1.33–1.28 (m, 12H). Spectral data agree with that reported in the literature.[8]

4-amino-3-bromo-N,N-diisopropylbenzamide

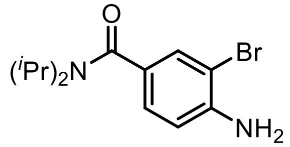

Prepared according to a modified literature procedure.[4] To a solution of 4-amino-*N,N*-diisopropylbenzamide (1.15 g, 5.22 mmol, 1.0 equiv.) in DMF (10 mL, 0.55M) at 0 °C was added *N*-bromosuccinimide (929 mg, 5.22 mmol, 1.0 equiv.). The reaction mixture was warmed up to rt and stirred for 2 h. Upon completion, water (100 mL) was added resulting in immediate formation of a white precipitate. Filtration of the suspension and wash of the precipitate with water afforded the product as a white solid (1.35 g, 4.54 mmol, 87%).

^{1}H NMR (500 MHz, $CDCl_3$, 55 °C) δ 7.41 (d, *J* = 1.8 Hz, 1H), 7.07 (dd, *J* = 8.2, 1.8 Hz, 1H), 6.70 (d, *J* = 8.1 Hz, 1H), 4.03 (s, 2H), 3.86 – 3.59 (m, 2H), 1.33 (d, *J* = 6.7 Hz, 12H).

^{13}C NMR (125 MHz, $CDCl_3$, 55 °C) δ 169.9, 145.0, 130.9, 130.3, 126.6, 115.2, 108.8, 48.6, 21.1, 21.1, 21.0.

HRMS (ESI) m/z calculated for $C_{13}H_{20}BrN_2O$ $[M+H]^+$: 299.0759, found 299.0757.

3-Bromo-*N,N*-diisopropyl-4-((4-methylphenyl)sulfonamido)benzamide (1o)

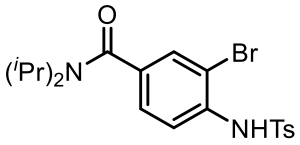

Prepared according to modified general procedure. To a solution of 4-amino-3-bromo-*N,N*-diisopropylbenzamide (1.25 g, 4.18 mmol, 1.0 equiv.) in pyridine (6.0 mL, 0.70M) at rt was added *p*-toluenesulfonyl chloride (796 mg, 4.18 mmol, 1.0 equiv.). The reaction mixture was heated to 115 ºC for 44 h. Upon completion, the reaction mixture was cooled down to rt, water (100 mL) was added, and the reaction mixture was stirred for 1 h resulting in precipitation of the crude product. This mixture was filtered, then the solid residue was washed with water and recrystallized in refluxing EtOH/hexanes to afford the pure product as a white crystalline solid (1.64 g, 3.62 mmol, 87%).

^{1}H NMR (500 MHz, $CDCl_3$, 55 °C) 7.68 (d, *J* = 8.2 Hz, 2H), 7.65 (d, *J* = 8.4 Hz, 1H), 7.41 (s, 1H), 7.23 (d, *J* = 8.0 Hz, 2H), 7.19 (d, *J* = 8.3 Hz, 1H), 7.00 (s, 1H), 3.72 – 3.52 (m, 2H), 2.39 (s, 3H), 1.32 (d, *J* = 6.0 Hz, 12H).

^{13}C NMR (100 MHz, $CDCl_3$) δ 168.6, 144.7, 136.5, 135.8, 135.3, 130.5, 130.0, 127.5, 126.1, 121.4, 115.2, 49.9 (br, see cross-peak in HSQC), 21.7, 20.8.

HRMS (ESI) m/z calculated for $C_{20}H_{26}BrN_2O_3S$ $[M+H]^+$: 453.0848, found 453.0849.

Preparation of 1,3-Dienes

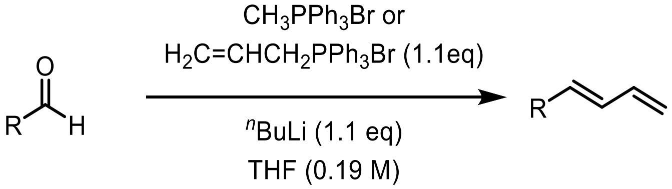

General procedure for 1,3-diene preparation from aldehydes: Prepared according to a modified literature procedure.[9] Methyl triphenylphosphonium bromide or allyl triphenylphosphonium bromide (1.10 equiv.) was measured into an oven–dried round bottom flask equipped with stir bar and septum. The flask was evacuated and refilled with argon three times before the addition of dry THF (0.20 M). *n*–Butyllithium (1.10 equiv.) was added dropwise to the suspension of methyltriphenylphosphonium bromide at –78 °C, the solution was warmed up to 0 °C and stirred for 1 h, lastly aldehyde (1.00 equiv.) added dropwise at 0 °C and the reaction mixture stirred for 2–3 hours. Upon completion, the solution was diluted with diethyl ether and solids were filtered through celite. Solvent was removed under reduced pressure; then crude residue was purified via column chromatography on SiO_2. Purified dienes were stored at –28 °C for no longer than one week.

(*E*)-1-Phenyl-1,3-butadiene (2b)

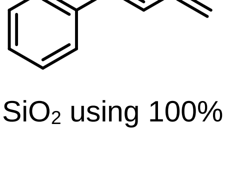

Prepared according to general procedure. Methyltriphenylphosphonium bromide (6.00 g, 16.7 mmol, 1.10 equiv.), THF (80 mL, 0.19 M), *n*–butyllithium (6.75 mL, 16.7 mmol, 1.00 equiv.), cinnamaldehyde (2.00 g, 15.1 mmol, 1.11 equiv.) were used. Crude material was purified via column chromatography on SiO_2 using 100% hexanes. Product was isolated as a colorless liquid (1.14 g, 8.80 mmol, 58%).

^{1}H NMR (400 MHz, $CDCl_3$) δ 7.42–7.40 (m, 2H), 7.34–7.31 (m, 2H), 7.25–7.22 (m, 1H), 6.81–6.76 (dd, *J* = 11.0, 11.0 Hz, 1H), 6.58 (d, *J* = 15.5 Hz, 1H), 6.53–6.49 (m, 1H), 5.34 (d, *J* = 17.2 Hz, 1H), 5.18 (d, *J* = 10.4 Hz, 1H). Spectral data agree with that reported in the literature.[10]

(*E*)-1-(buta-1,3-dien-1-yl)-4-methoxybenzene (2c)

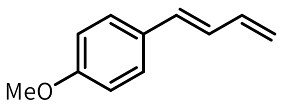

Prepared according to general procedure. Methyltriphenylphosphonium bromide (6.00 g, 16.7 mmol, 1.10 equiv.), THF (80 mL, 0.19M), *n*–butyllithium (6.75 mL, 16.7 mmol, 1.10 equiv.), 4–methoxycinnamaldehyde (2.45 g, 15.1 mmol, 1.00 equiv.) were used. Crude material was purified via column chromatography on SiO_2 using 7% EtOAc/hexanes. Product was isolated as a colorless liquid (1.95 g, 12.2 mmol, 82%).

^{1}H NMR (400 MHz, $CDCl_3$): δ 7.34 (d, *J* = 8.6 Hz, 2H), 6.86 (d, *J* = 8.6 Hz, 2H), 6.67 (dd, *J* = 10.4, 15.2 Hz, 1H), 6.52 (d, *J* = 10.4 Hz, 1H), 6.50—6.44 (m, 1H), 5.28 (d, *J* = 16.0 Hz, 1H), 5.11 (d, *J* = 9.2 Hz, 1H), 3.81 (s, 3H). Spectral data agree with that reported in the literature.[9]

5-(buta-1,3-dien-1-yl)-1,2,3-trimethoxybenzene (2d)

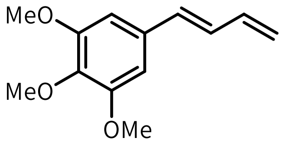

Prepared according to general procedure. Allylltriphenylphosphonium bromide (6.40 g, 16.7 mmol, 1.10 equiv.), THF (80 mL, 0.19 M), *n*–butyllithium (6.75 mL, 16.7 mmol, 1.10 equiv.), 3,4,5–trimethoxy–benzaldehyde (2.96 g, 15.1 mmol, 1.00 equiv.) were used. Crude material was purified via column chromatography on SiO_2 using 10% → 15% EtOAc/hexanes. Pure product was isolated as white solid 1:1 mixture of *E/Z* isomers (1.63 g, 7.39 mmol, 49%).

E isomer: ^{1}H NMR (400 MHz, $CDCl_3$) δ 6.96–6.84 (m, 1H), 6.64 (s, 2H), 6.50–6.37 (m, 2H), 5.35 (d, *J* =17.1 Hz, 1H), 5.16 (dd, *J* =9.6, 1.5 Hz, 1H), 3.87 (s, 6H), 3.86 (s, 3H);

Z isomer: ^{1}H NMR (400 MHz, $CDCl_3$) δ 6.73–6.66 (m, 1H), 6.54 (s, 2H), 6.51–6.46 (m, 1H), 6.23 (t, *J* =11.3, 1H), 5.43–5.30 (m, 1H), 5.23 (d, *J* =10.2 Hz, 1H), 3.89 (s, 3H), 3.87 (s, 6H). Spectral data agree with that reported in the literature.[11]

(*E*)-1-(buta-1,3-dien-1-yl)-3-(trifluoromethyl)benzene (2e)

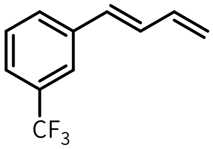

Prepared according to general procedure. Allylltriphenylphosphonium bromide (9.32 g, 26.1 mmol, 1.10 equiv.), THF (131 mL, 0.18 M), *n*–butyllithium (10.4 mL, 26.1 mmol, 1.10 equiv.), *trans*-3-octen-2-one (3.00 g, 23.7 mmol, 1.00 equiv.) were used. Crude material was purified via column chromatography on SiO_2 using pentane. Product was isolated as colorless oil (1.94 g, 15.6 mmol, 66%).

^{1}H NMR (400 MHz, $CDCl_3$) δ 7.63 (d, *J* = 1.4 Hz, 1H), 7.54 (d, *J* = 7.7 Hz, 1H), 7.47 – 7.38 (m, 2H), 6.83 (m, 1H), 6.59 – 6.45 (m, 2H), 5.42 – 5.37 (m, 1H), 5.26 – 5.23 (m, 1H). Spectral data agree with that reported in the literature.[12]

(*E*)-5-(buta-1,3-dien-1-yl)-2,3-dihydrobenzofuran (2f)

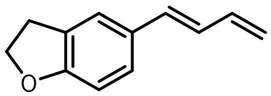

Prepared according to general procedure. Allylltriphenylphosphonium bromide (5.68 g, 14.8 mmol, 1.10 equiv.), THF (67.4 mL, 0.20 M), *n*–butyllithium (9.20 mL, 14.7 mmol 1.09 equiv.), 2,3-dihydro-benzofuran-5-carbaldehyde (2.00 g, 13.5 mmol, 1.00 equiv.) were used. Crude material was purified via column chromatography on SiO_2 using 10% EtOAc/hexanes. Product was isolated as colorless oil (1.81 g, 10.5 mmol, 78%).

^{1}H NMR (400 MHz, $CDCl_3$) δ 7.28 – 6.72 (m, *Z*+*E*), 6.64 (dd, *J* = 15.3, 10.6 Hz, 1H, *E*), 6.53 – 6.42 (m, 2H, *E*), 6.38 (d, *J* = 11.5 Hz, 1H, *Z*), 6.19 – 6.12 (m, 1H, Z), 5.36 – 5.31 (m, 1H, *Z*), 5.28 – 5.23 (m, 1H, *E*), 5.20 – 5.16 (m, 1H, *Z*), 5.10 – 5.07 (m, 1H, *E*), 4.58 (t, *J* = 8.7, 2H, *Z*), 4.57 (t, *J* = 8.7, 2H, *E*), 3.20 (t, *J* = 8.7, 2H, *Z*), 3.18 (t, *J* = 8.7, 2H, *E*). Spectral data agree with that reported in the literature.[12]

2-(buta-1,3-dien-1-yl)thiophene (2g)

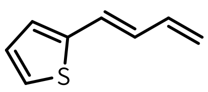

Prepared according to general procedure. Allylltriphenylphosphonium bromide (6.09 g, 15.9 mmol, 1.13 equiv.), THF (52.5 mL. 0.27 M), *n*–butyllithium (6.37 mL, 15.9 mmol, 1.13 equiv.), thiophene-2-carbaldehyde (1.58 g, 14.1 mmol, 1.00 equiv.) were used. Crude material was purified via column chromatography on SiO_2 using hexanes. Product was isolated as yellow oil (0.734 g, 5.39 mmol, 38%). Material was used immediately as it decomposes over time.

^{1}H NMR (400 MHz, $CDCl_3$) δ 5.15 (1H, ddt, *J* = 10.1, 1.5, 0.7 Hz), 5.31 (1H, ddt, *J* = 16.9, 1.6, 0.7 Hz), 6.44 (1H, dt, *J* = 16.8, 10.0 Hz), 6.57–6.75 (2H, m), 6.94–6.99 (2H, m), 7.14–7.19 (1H, m). Spectral data agree with that reported in the literature.[13]

(*E*)-3-(buta-1,3-dien-1-yl)-Me-1*H*-indole (2h)

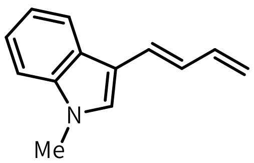

Prepared according to general procedure. Allylltriphenylphosphonium bromide (5.29 g, 13.8 mmol. 1.10 equiv.), THF (62.8 mL, 0.20 M), *n*–butyllithium (8.60 mL, 13.8 mmol, 1.10 equiv.), 1-methylindole-3-carboxaldehyde (2.01 g, 12.6 mmol. 1.00 equiv.) were used. Crude material was purified via column chromatography on SiO_2 using hexanes → 5% EtOAc/hexanes. Product was isolated as yellow oil (0.706 g, 3.85 mmol, 28%). Material was used immediately as it decomposes over time.

^{1}H NMR (400 MHz, $CDCl_3$) δ 7.67 (d, *J* = 7.9 Hz, 1H), 7.35 – 7.24 (m, 2H), 7.17 (dd, *J* = 15.6, 8.5 Hz, 2H), 6.98 (tt, *J* = 19.9, 10.1 Hz, 1H), 6.62 (d, *J* = 11.2 Hz, 1H), 6.23 (t, *J* = 11.2 Hz, 1H), 5.37 (d, *J* = 16.9 Hz, 1H), 5.18 (d, *J* = 10.1 Hz, 1H), 3.81 (s, 3H). Spectral data agree with that reported in the literature.[14]

Preparation of (*E*)-hexa-3,5-dien-1-ol (2i)

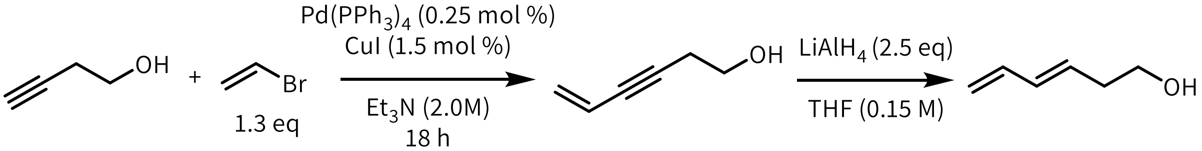

Hex-5-en-3-yn-1-ol

In the glovebox, $Pd(PPh_3)_4$ (34.0 mg, 0.0295 mmol, 0.00250 equiv.) and CuI (33.0 mg, 0.177 mmol, 0.0150 equiv.) were measured into an oven–dried round bottomed flask equipped with a stir bar. The remaining reagents were added outside the glovebox under a stream of N_2. After the addition of triethylamine (5.90 mL, 2.00 M), the solution was degassed with N_2 for 5 min. After cooling the reaction mixture to 0°C, 1-butyn-4-ol (1.00 g, 11.8 mmol 1.00 equiv.) and vinyl bromide (7.65 mL, 15.3 mmol, 2.53 equiv.) were added dropwise to the reaction mixture, then reaction was slowly brought to rt. After 18h the reaction was quenched with sat. aq. NH_4Cl at 0 °C and extracted with Et_2O (3 x 5 mL), then the combined organic layers were dried over $MgSO_4$. Upon concentration, yellow oil (0.59 g, 6.1 mmol) was used immediately without further purification.

(*E*)-Hexa-3,5-dien-1-ol (2i)

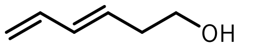

$LiAlH_4$ (0.940 g, 24.8 mmol, 4.00 equiv.) was suspended in dry THF (40 mL, 0.150 M) in a round–bottom flask equipped with stir bar and septum, then the flask was cooled to 0 °C and left to stir for 1h. Hex-5-en-3-yn-1-ol (0.590 g, 6.10 mmol, 1.00 equiv.) was added at 0 °C dropwise, the solution was warmed up to rt (20 °C) and then placed in 60 °C oil bath for 3 days. Upon completion, reaction mixture was cooled to 0 °C and quenched using the Fieser method: diethyl ether (10 mL), water (2 mL), 15 wt % aq. NaOH (2 mL), water (4 mL), and $MgSO_4$ (1.00 g) were added sequentially, then the reaction mixture warmed to rt before filtering through celite. Crude material was purified via column chromatography on SiO_2 with 70% pentane/diethyl ether to afford **2i** as a colorless oil (0.370 g, 3.76 mmol, 62%). Material was used immediately as it decomposes over time.

^{1}H NMR (400 MHz, $CDCl_3$) δ 6.37 (dd, *J* = 17.6, 10.8 Hz, 1H), 5.35 – 5.27 (m, 1H), 5.09 (d, *J* = 10.9 Hz, 1H), 5.02 (d, *J* = 3.8 Hz, 2H), 2.71 – 2.61 (m, 2H), 2.52 – 2.40 (m, 2H). Spectral data agree with that reported in the literature.[15]

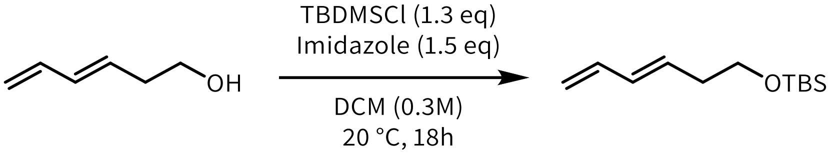

(*E*)-Tert-butyl(hexa-3,5-dien-1-yloxy)dimethylsilane (2j)

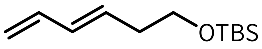

Alcohol **2i** (300.0 mg 3.06 mmol 1.00 equiv.), tert-butyldimethylsilyl chloride (609.8 mg 4.05 mmol, 1.30 equiv.), and imidazole (317 mg, 4.65 mmol, 1.50 equiv.) were dissolved in DCM (10 mL, 0.30 M) and left to stir at rt (20 °C) for 18h. Reaction mixture was quenched with water (5 mL), washed with sat. aq. $NaHCO_3$ (10 mL), and extracted with hexanes (3 x 5 mL), then the combined organic layers were dried over Na_2SO_4. Crude material was purified via column chromatography on SiO_2 with 10% EtOAc/hexanes to afford product as a colorless oil (0.390 g, 3.76 mmol, 60%).

^{1}H NMR (400 MHz, $CDCl_3$) δ 6.29 (1H, ddd, J= 17.0, 10.2, 10.2 Hz), 6.08 (1H, dd, J= 15.3, 10.5 Hz), 5.68 (1H, dt, J= 15.3, 7.1 Hz), 5.09 (1H, d, J= 17.0), 4.96 (1H, d, J= 10.2), 3.64 (2H, t, J= 6.8 Hz), 2.32–2.27 (2H, m), 0.88 (9H, s), 0.03 (6H, s). Spectral data agree with that reported in the literature.[16]

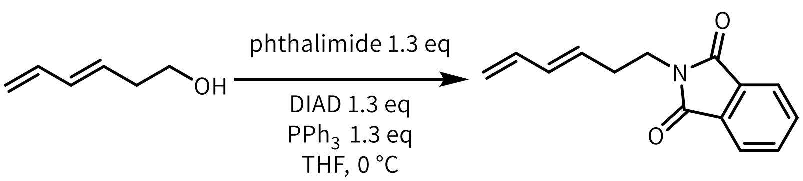

(*E*)- 2-(Hexa-3,5-dien-1-yl)isoindoline-1,3-dione (2k)

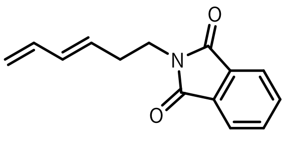

Alcohol **2i** (1.00 g, 10.2 mmol, 1.00 equiv.), triphenylphosphine (3.40 g, 13.2 mmol, 1.30 equiv.), phthalimide (1.91 g, 13.2 mmol, 1.30 equiv.) and 70 mL of THF (0.146 M) were cooled to 0°C. To the cold reaction mixture added diisopropyl azodicarboxylate (2.60 mL, 13.2 mmol, 1.30 equiv.). Upon consumption of starting material as monitored by TLC, the reaction mixture was diluted with 30 mL of water and extracted with hexanes (3 x 5 mL). The organic layer was washed with brine (3 x 5 mL) and dried over $MgSO_4$. Crude material was purified via column

chromatography on SiO_2 using hexanes → 5% EtOAc/hexanes. Pure product was isolated as a white solid (1.03 g, 4.53 mmol, 44%). Material was used immediately as it decomposes over time.

^1H NMR (400 MHz, $CDCl_3$) δ 7.76 (d, *J* = 8.3 Hz, 2H), 7.32 (d, *J* = 8.3 Hz, 2H), 6.21 (ddd, *J* = 16.9, 10.3, 10.3 Hz, 1H), 6.03 (dd, *J* = 15.3, 10.6 Hz, 1H), 5.94 (dt, *J* = 15.3, 7.0 Hz, 1H), 5.09 (d, *J* = 16.7 Hz, 1H), 5.01 (d, *J* = 10.8 Hz, 1H), 4.04 (t, *J* = 6.7 Hz, 2H), 2.45–2.37 (m, 2H). Spectral data agree with that reported in the literature.[16]

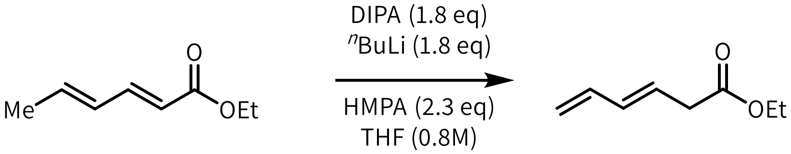

Ethyl (*E*)-3,5-hexadienoate (2l)

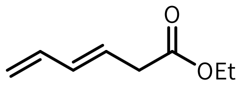

Prepared according to a modified literature procedure.[17] Diisopropylamine (5.70 mL, 40.7 mmol 1.79 equiv.) was taken up in dry THF (27.5 mL, 0.80 M) in an oven–dried round bottom flask equipped with stir bar and septum under argon. The flask was cooled to –78°C and n–butyllithium (25.5 mL, 41.0 mmol, 1.80 equiv.) was added the solution was left to stir for 20 min before the addition of HMPA (9.20 mL, 52.9 mmol, 2.32 equiv.). The reaction mixture was left to still for another 20 minutes at –78°C before the addition of ethyl sorbate (3.20 g, 22.8 mmol, 1.00 equiv.) in THF (9.00 mL, 2.50 M). After 1 hour the reaction mixture was poured into water/ice mixture (50 mL) and extracted with hexanes. Organic layer was washed with sat. aq. $NaHCO_3$, brine (3 x 5 mL), and dried over $MgSO_4$. Crude material was purified via column chromatography on SiO_2 using hexanes → 5% EtOAc/hexanes. Pure product was isolated as colorless oil (1.98 g, 14.1 mmol, 62%). Material was used immediately as it decomposes over time.

^1H NMR (400 MHz, $CDCl_3$) δ 6.33 (dt, *J* = 16.9, 10.3 Hz, 1H), 6.14 (dd, *J* = 15.2, 10.5 Hz, 1H), 5.85 – 5.72 (m, 1H), 5.16 (d, *J* = 16.8 Hz, 1H), 5.05 (d, *J* = 10.1 Hz, 1H), 4.14 (q, *J* = 7.2 Hz, 2H), 3.11 (d, *J* = 7.1 Hz, 2H), 1.26 (t, *J* = 7.1 Hz, 3H). Spectral data agree with that reported in the literature.[17]

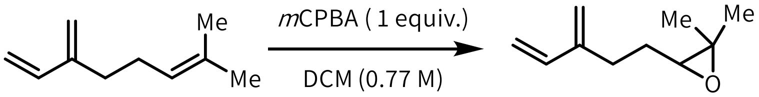

6,7-epoxymyrcene (2n)

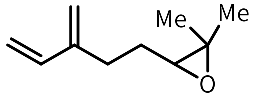

Prepared according to modified literature procedure.[18] Myrcene **2a** (10.6 g, 77.8 mmol 1.00 equiv.) was taken up in CH_2Cl_2 (100 mL, 0.77 M) in a round bottom flask. After cooling to 0°C, *m*CPBA (17.0 g, 77.8 mmol, 1.00 equiv.) was added, then the reaction mixture stirred for 10 min at 0°C. Upon completion, the reaction mixture was quenched with NaOH (2.00 M aq. sol.) and extracted with DCM (3 x 10 mL). The combined organic layers were washed with sat. aq. NaS_2O_3, water, and brine (3 x 7 mL), then dried over $MgSO_4$. Crude material was purified via column chromatography on SiO_2 using 5% → 10% EtOAc/hexanes. Pure product was isolated as colorless oil (6.28 g, 41.2 mmol, 53%).

^1H NMR (400 MHz, $CDCl_3$) δ 6.38 (dd, *J* = 17.5, 11.0 Hz, 1 H), 5.24 (d, *J* = 17.5 Hz, 1 H), 5.08 (d, *J* = 11.0 Hz, 1 H), 5.02 (s, 1 H), 5.05 (s, 1 H), 2.76 (t, *J* = 6.2 Hz, 1 H), 2.40–2.48 (m, 1 H),

2.28–2.36 (m, 1 H), 1.71–1.76 (m, 2 H), 1.31 (s, 3 H), 1.26 (s, 3 H). Spectral data agree with that reported in the literature.[18]

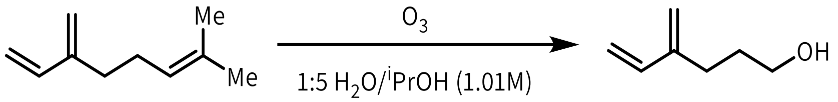

4-methylene-hex-5-en-1-ol (2o)

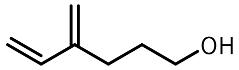

Prepared according to modified literature procedure.[19] Myrcene **2a** (3.44 mL, 20.1 mmol, 1.00 equiv.) was taken up in 1:5 $H_2O/^{i}PrOH$ (20 mL, 1.01 M) in a pear-shaped flask equipped with stir bar. Ozone gas was bubbled into this solution for 50 min. Upon completion, argon was bubbled through reaction mixture for 10 min. $NaBH_4$ (467.0 mg 12.3 mmol, 0.610 equiv.) was added, then the reaction mixture stirred for 5 minutes. The reaction mixture was diluted with water and extracted with Et_2O (3 x 5 mL), then the combined organic layers were washed with sat. aq. NH_4Cl and brine (3 x 5 mL) three times and dried over $MgSO_4$. Crude material was purified via column chromatography on SiO_2 using 5% → 25% EtOAc/hexanes. Pure product was isolated as a colorless oil (1.06 g, 9.43 mmol, 47%).

^1H NMR (500 MHz, $CDCl_3$) δ 6.38 (dd, *J* = 17.6, 10.8 Hz, 1H), 5.27 (dd, *J* = 17.6, 0.5 Hz, 1H), 5.08 (d, *J* = 10.8 Hz, 1H), 5.04 (d, *J* = 0.5 Hz, 1H), 5.01 (s, 1H), 3.69 (td, *J* = 6.4, 5.5 Hz, 2H), 2.31 (t, *J* = 7.3 Hz, 2H), 1.75–1.81 (m, 2H), 1.26 (t, *J* = 5.5 Hz, 1H). Spectral data agree with that reported in the literature.[20]

Preparation of 4-Me-(*E*)-hexa-3,5-dien-1-ol (2p)

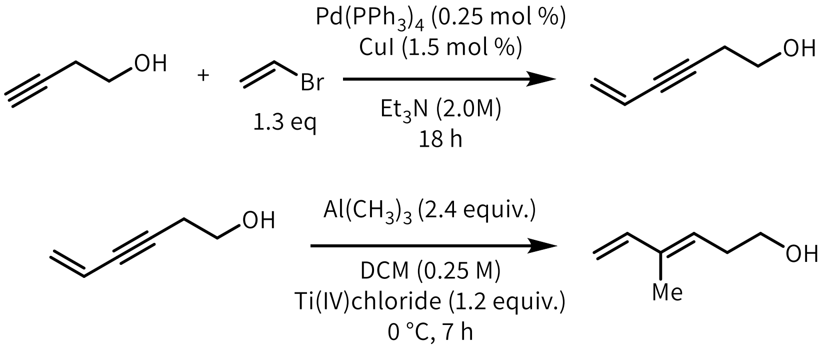

Hex-5-en-3-yn-1-ol

In the glovebox, $Pd(PPh_3)_4$ (34.0 mg, 0.0295 mmol, 0.00250 equiv.) and CuI (33.0 mg, 0.177 mmol, 0.0150 equiv.) were measured into an oven–dried round bottomed flask equipped with a stir bar. The remaining reagents were added outside the glovebox under a stream of N_2. After the addition of triethylamine (5.90 mL, 2.00 M), the solution was degassed with N_2 for 5 min. After cooling the reaction mixture to 0°C, 1-butyn-4-ol (1.00 g, 11.8 mmol 1.00 equiv.) and vinyl bromide (7.65 mL, 15.3 mmol, 2.53 equiv.) were added dropwise to the reaction mixture, then reaction was slowly brought to rt. After 18h the reaction was quenched with sat. aq. NH_4Cl at 0 °C and extracted with Et_2O (3 x 5 mL), then the combined organic layers were dried over $MgSO_4$. Upon concentration, yellow oil (0.59 g, 6.1 mmol) was used immediately without further purification.

4-Me-(*E*)-hexa-3,5-dien-1-ol (2p)

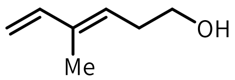

Prepared according to a modified literature procedure.[21] Trimethylaluminum solution (24.0 mL, 48.0 mmol, 2.40 equiv.) was taken up in dry DCM (40.0 mL, 0.50 M) in an oven–dried round bottom flask equipped with stir bar and septum under argon. The flask was cooled to 0 °C and hex-5-en-3-yn-1-ol (1.64 g, 20.0 mmol, 1.00 equiv.) was added dropwise, generated methane was vented to release the pressure. $TiCl_4$ (24.0 mL, 24.0 mmol, 1.20 equiv.) was taken up in dry DCM (40.0 mL, 0.50 M) in a separate flame–dried round bottom flask equipped with stir bar and septum under argon. After cooling both flasks to –20 °C, the $TiCl_4$ solution was cannulated into the trimethylaluminum solution. The reaction was stirred for 30 sec after the addition of $TiCl_4$ and then quenched via syringe addition of MeOH (10 mL) pre–cooled to 0 °C as rapidly as gas evolution could be controlled (1 min). After, HCl (10 % aq. sol.) saturated with NaCl (40 mL) was added to the reaction mixture, which was allowed to warm to room temp over 30 min. The reaction mixture was washed with water (3 x 10 mL) and extracted with DCM (3 x 5 mL), combined organic layers were dried over $MgSO_4$. Crude material was purified via column chromatography on SiO_2 with 65% Et_2O/hexanes. Pure product was isolated as colorless oil (1.53 g, 13.6 mmol, 68%).

^{1}H NMR (400 MHz, $CDCl_3$) δ 6.38 (dd, *J* = 17.5, 10.5 Hz, 1 H), 5.49 (t, *J* = 7.5 Hz, 1 H), 5.12 (d, *J* = 17.5 Hz, 1 H), 4.97 (d, *J* = 10.5 Hz, 1 H), 3.66 (t, *J* = 6.5 Hz, 2 H), 2.42 (app. q, *J* = 7.0 Hz, 2 H), 1.96 (br s, 1 H), 1.77 (s, 3 H). Spectral data agree with that reported in the literature.[22]

(*E*)-2-Me-1-phenyl-1,3-butadiene (2q)

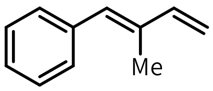

Dry KO^{t}Bu (1.99 g, 17.7 mmol 1.30 equiv.), methyltriphenylphosphonium bromide (6.11 g, 17.1 mmol 1.20 equiv.), THF (55 mL, 0.25 M), and *α*-methyl-*trans*-cinnamaldehyde (2.02 g, 13.8 mmol, 1.00 equiv.) were combined in a round-bottom flask equipped with a stir bar under inert atmosphere. Reaction was allowed to stir at rt (20 °C) for 2 days. Upon completion, reaction mixture was filtered through a celite plug with diethyl ether. Crude material was purified via column chromatography on SiO_2 using hexanes. Product was isolated as colorless oil (1.91 g, 13.2 mmol, 96%).

^{1}H NMR (400 MHz, $CDCl_3$) δ 7.49 – 7.33 (5H, m), 6.70 (2H, m), 5.45 (1H, d, *J* = 17.6 Hz), 5.28 (1H, d, *J* = 10.4 Hz), 2.15 (3H, s). Spectral data agree with that reported in the literature.[23]

***Trans*-2-Me-1,3-octadiene (2r)**

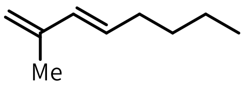

Prepared according to general procedure. Methyltriphenylphosphonium bromide (6.40 g, 16.7 mmol, 1.10 equiv.), THF (80 mL, 0.19 M), *n*-butyllithium (6.75 mL, 16.7 mmol, 1.10 equiv.), 3-trifluoromethylbenzaldehyde (2.59 g, 15.1 mmol, 1.00 equiv.) were used. Crude material was purified via column chromatography on SiO_2 using hexanes. Product was isolated as viscous colorless oil (1.46 g, 7.39 mmol, 49%).

^{1}H NMR (400 MHz, $CDCl_3$) δ 6.14 (d, *J* = 15.6 Hz, 1H), 5.66 (dt, *J* = 15.5, 7.0 Hz, 1H), 4.86 (s, 2H), 2.11 (q, *J* = 6.8 Hz, 2H), 1.83 (s, 3H), 1.44–1.25 (m, 4H), 0.91 (t, *J* = 7.1 Hz, 3H). Spectral data agree with that reported in the literature.[24]

Preparation of Ureas

General procedure for preparation of monosubstituted and 1,1-disubstituted ureas from amines: Prepared according to a modified literature procedure.[25] To a solution of corresponding primary or secondary amine (1.0 equiv.) in water/glacial acetic acid (1:1, *ca.* 0.25M) was added potassium cyanate (1.5 or 3.0 equiv.). The resulting suspension was stirred for 18 hours and then quenched with ice. The resulting precipitate was collected by filtration and washed with water to afford the crude product. The crude product was recrystallized in refluxing ethanol/hexanes to give the desired urea.

1-Methyl-1-phenylurea (4c)

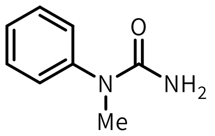

Prepared according to general procedure. *N*-Methylaniline (1.99 g, 18.6 mmol, 1.0 equiv.), water/glacial acetic acid (1:1, 75 mL, 0.25M), and potassium cyanate (4.53 g, 55.8 mmol, 3.0 equiv.) were used. The recrystallized product was obtained as an off-white crystalline solid (2.40 g, 16.0 mmol, 86%).

^{1}H NMR (400 MHz, $CDCl_3$) δ 7.41 (t, *J* = 7.6 Hz, 2H), 7.19–7.35 (m, 3H), 5.11 (s, 2H), 3.23 (s, 3H). Spectral data agree with that reported in the literature.[26]

1-Butyl-3-phenylurea (4d)

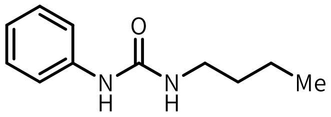

To a solution of phenyl isocyanate (1.00 mL, 9.16 mmol, 1.0 equiv.) in hexanes (40 mL, 0.23 M) was added dropwise *n*-butylamine (0.91 mL, 9.16 mmol, 1.0 equiv.). The immediately formed white suspension was stirred vigorously for 10 minutes, filtered, and washed with hexanes (2x 100 mL) to give urea **4d** as a white solid (1.62 g, 8.42 mmol, 92%).

^{1}H NMR (400 MHz, DMSO-d_6) δ 8.36 (s, 1H), 7.38 (d, *J* = 7.6 Hz, 2H), 7.20 (dd, *J* = 7.6, 7.2 Hz, 2H), 6.87 (t, *J* = 7.2 Hz, 1H), 6.01 (t, *J* = 5.6 Hz, 1H), 3.08 (q, *J* = 5.6 Hz, 2H), 1.44–1.26 (m, 4H), 0.89 (t, *J* = 7.2 Hz, 3H). Spectral data agree with that reported in the literature.[27]

1,1-Diethyl-3-phenylurea (4e)

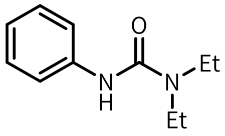

To a solution of phenyl isocyanate (1.00 mL, 9.16 mmol, 1.0 equiv.) in hexanes (40 mL) was added dropwise diethylamine (1.00 mL, 9.55 mmol, 1.04 equiv.). The immediately formed white suspension was stirred vigorously for 10 minutes, filtered, and washed with hexanes (2x 100 mL) to give urea **4e** as a white solid (1.67 g, 8.70 mmol, 95%).

^{1}H NMR (400 MHz, $CDCl_3$) δ 7.38 (d, *J* = 8.0 Hz, 2H), 7.27 (dd, *J* = 8.0, 7.2 Hz, 2H), 7.01 (t, *J* = 7.2 Hz, 1H), 6.29 (s, 1H), 3.37 (q, *J* = 7.2 Hz, 4H), 1.22 (t, *J* = 7.2 Hz, 3H). Spectral data agree with that reported in the literature.[27]

1,1-Diethyl-3-methyl-3-phenylurea (4f)

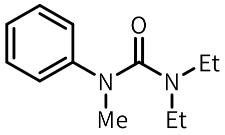

To a cooled (0 °C) solution of urea **4e** (300 mg, 1.56 mmol, 1.0 equiv.) in DMF (6.0 mL, 0.26M) was added portionwise sodium hydride (62 mg of 60% dispersion in mineral oil, 1.56 mmol, 1.0 equiv.). After cessation of hydrogen gas evolution (~5 min.) the solution was warmed up to rt and stirred for additional 30 min. Then, iodomethane (100 uL, 1.56 mmol, 1.0 equiv.) was added and the reaction was stirred at room temperature overnight (14h). The reaction was quenched by addition of water (20 mL) and extracted with ethyl acetate (3x 20 mL). The combined organic layers were washed with brine (60 mL) and upon removal of volatiles under reduced

pressure a crude product was obtained. Subsequent purification via column chromatography on SiO_2 (ethyl acetate/hexanes 1:4) afforded urea **4f** as a yellow oil (250 mg, 1.20 mmol, 77%).

^{1}H NMR (400 MHz, $CDCl_3$) δ 7.32–7.24 (m, 2H), 7.10–7.01 (m, 3H), 3.12 (s, 3H), 3.08 (q, *J* = 7.1 Hz, 4H), 0.89 (t, *J* = 7.1 Hz, 6H). Spectral data agree with that reported in the literature.[28]

1-Benzylurea (4h)

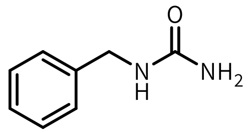

Prepared according to general procedure. Benzylamine (1.80 g, 16.8 mmol, 1.0 equiv.), water/glacial acetic acid (1:1, 24 mL, 0.70M), and potassium cyanate (2.04 g, 25.2 mmol, 1.5 equiv.) were used. The recrystallized product was obtained as a white crystalline solid (990 mg, 6.59 mmol, 39%).

^{1}H NMR (400 MHz, $CDCl_3$) δ 7.33–7.14 (m, 5H), 6.40 (s, 1H), 5.22 (s, 2H), 4.17 (s, 2H). Spectral data agree with that reported in the literature.[26]

1-Cyclohexylurea (4i)

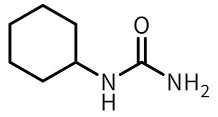

Prepared according to general procedure. Cyclohexylamine (992 mg, 10.0 mmol, 1.0 equiv.), water/glacial acetic acid (1:1, 40 mL, 0.25M), and potassium cyanate (2.43 g, 30.0 mmol, 3.0 equiv.) were used. The recrystallized product was obtained as a white solid (1.07 g, 7.52 mmol, 75%).

^{1}H NMR (400 MHz, DMSO-d6) δ 5.81 (d, *J* = 7.7 Hz, 1H), 5.28 (s, 2H), 3.33–3.26 (m, 1H), 1.75–1.70 (m, 2H), 1.66–1.59 (m, 2H), 1.58–1.46 (m, 1H), 1.32–1.20 (m, 2H), 1.17–0.97 (m, 3H). Spectral data agree with that reported in the literature.[26]

Methyl 4-ureidobenzoate (4j)

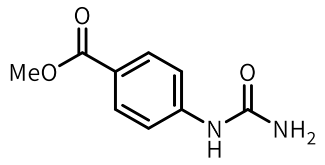

Prepared according to general procedure. Methyl 4-aminobenzoate (997 mg, 6.60 mmol, 1.0 equiv.), water/glacial acetic acid (1:1, 25 mL, 0.26M), and potassium cyanate (1.61 g, 19.8 mmol, 3.0 equiv.) were used. The recrystallized product was obtained as a white solid (538 mg, 2.77 mmol, 42%).

^{1}H NMR (400 MHz, DMSO-d6) δ 8.94 (s, 1H), 7.82 (d, *J* = 8.1 Hz, 2H), 7.52 (d, *J* = 8.1 Hz, 2H), 6.04 (s, 2H), 3.79 (s, 3H). Spectral data agree with that reported in the literature.[29]

1-(4-Methoxyphenyl)urea (4k)

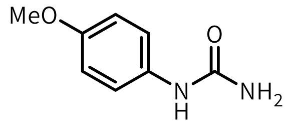

Prepared according to general procedure. 4-Methoxyaniline (1.00 g, 8.10 mmol, 1.0 equiv.), water/glacial acetic acid (1:1, 30 mL, 0.27M), and potassium cyanate (1.97 g, 24.3 mmol, 3.0 equiv.) were used. The recrystallized product was obtained as an off-white solid (713 mg, 4.29 mmol, 53%).

^{1}H NMR (400 MHz, $CDCl_3$) δ 7.21 (d, *J* = 8.8 Hz, 2H), 6.82 (d, *J* = 8.8 Hz, 2H), 3.73 (s, 3H). Spectral data agree with that reported in the literature.[29]

1-(2-Methylphenyl)urea (4l)

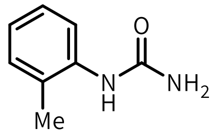

Prepared according to general procedure. *o*-Toluidine (2.02 g, 18.8 mmol, 1.0 equiv.), water/glacial acetic acid (1:1, 75 mL, 0.25M), and potassium cyanate (4.56 g, 56.4 mmol, 3.0 equiv.) were used. The recrystallized product was obtained as an off-white solid (875 mg, 5.83 mmol, 31%).

^{1}H NMR (400 MHz, DMSO-d6) δ 7.76 (m, 1H), 7.70 (s, 1H), 7.11–7.03 (m, 2H), 6.87 (m, 1H), 6.01 (s, 2H), 2.17 (s, 3H). Spectral data agree with that reported in the literature.[30]

1-([1,1'-Biphenyl]-2-yl)urea (4m)

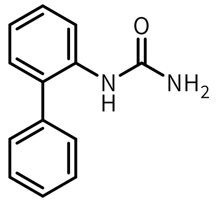

Prepared according to general procedure. [1,1'-Biphenyl]-2-amine (1.00 g, 5.96 mmol, 1.0 equiv.), water/glacial acetic acid (1:1, 25 mL, 0.24M), and potassium cyanate (1.45 g, 17.9 mmol, 3.0 equiv.) were used. The recrystallized product was obtained as a white solid (1.14 g, 5.30 mmol, 89%).

^{1}H NMR (400 MHz, $CDCl_3$) δ 7.79 (d, *J* = 8.0 Hz, 1H), 7.48–7.42 (m, 2H), 7.41–7.36 (m, 4H), 7.30–7.26 (m, 2H), 7.22–7.19 (m, 1H), 6.25 (s, 1H). Spectral data agree with that reported in the literature.[31]

1-(2,4-Dimethylphenyl)urea (4n)

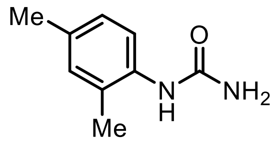

Prepared according to general procedure. 2,4-Dimethylaniline (1.01 g, 8.35 mmol, 1.0 equiv.), water/glacial acetic acid (1:1, 30 mL, 0.28M), and potassium cyanate (2.03 g, 25.1 mmol, 3.0 equiv.) were used. The recrystallized product was obtained as a white solid (1.16 g, 7.10 mmol, 85%).

^{1}H NMR (400 MHz, DMSO-d6) δ 7.62–7.57 (m, 2H), 6.92 (s, 1H), 6.88 (d, *J* = 8.0 Hz, 1H), 5.90 (s, 2H), 2.19 (s, 3H), 2.13 (s, 3H).

^{13}C NMR (125 MHz, DMSO-d6) δ 156.3, 135.5, 130.9, 130.5, 127.4, 126.4, 121.4, 20.3, 17.8.

HRMS (ESI) m/z calculated for $C_9H_{13}N_2O$ $[M+H]^+$: 165.1028, found 165.1021.

1-(2,4,6-Trimethylphenyl)urea (4o)

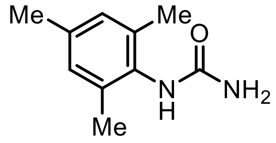

Prepared according to general procedure. 2,4,6-Trimethylaniline (1.00 g, 7.41 mmol, 1.0 equiv.), water/glacial acetic acid (1:1, 30 mL, 0.25M), and potassium cyanate (1.80 g, 22.2 mmol, 3.0 equiv.) were used. The recrystallized product was obtained as a white solid (910 g, 5.11 mmol, 69%).

^{1}H NMR (500 MHz, DMSO-d6) δ 7.39 (br, 1H), 6.83 (s, 2H), 5.61 (br, 2H), 2.20 (s, 3H), 2.11 (s, 6H).

^{13}C NMR (125 MHz, DMSO-d6) δ 156.8, 135.3, 133.5, 128.2, 20.5, 18.1.

HRMS (ESI) m/z calculated for $C_{10}H_{15}N_2O$ $[M+H]^+$: 179.1184, found 179.1177.

1-(4-Methoxy-2-methylphenyl)urea (4p)

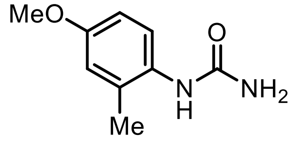

Prepared according to general procedure. 4-Methoxy-2-methylaniline (1.04 g, 7.32 mmol, 1.0 equiv.), water/glacial acetic acid (1:1, 30 mL, 0.24M), and potassium cyanate (1.78 g, 22.0 mmol, 3.0 equiv.) were used. The recrystallized product was obtained as a off-white solid (1.2 g, 6.66 mmol, 91%).

^{1}H NMR (500 MHz, DMSO-d6) δ 7.63 – 7.38 (m, 2H), 6.77 – 6.64 (m, 2H), 5.81 (br, 2H), 3.94 – 3.50 (m, 3H), 2.15 (s, 3H).

^{13}C NMR (125 MHz, DMSO-d6) δ 156.6, 155.0, 131.0, 130.6, 124.0, 115.3, 111.1, 55.1, 18.1.

HRMS (ESI) m/z calculated for $C_9H_{13}N_2O_2$ $[M+H]^+$: 181.0977, found 181.0973.

Ligand Structure-reactivity Relationship Studies

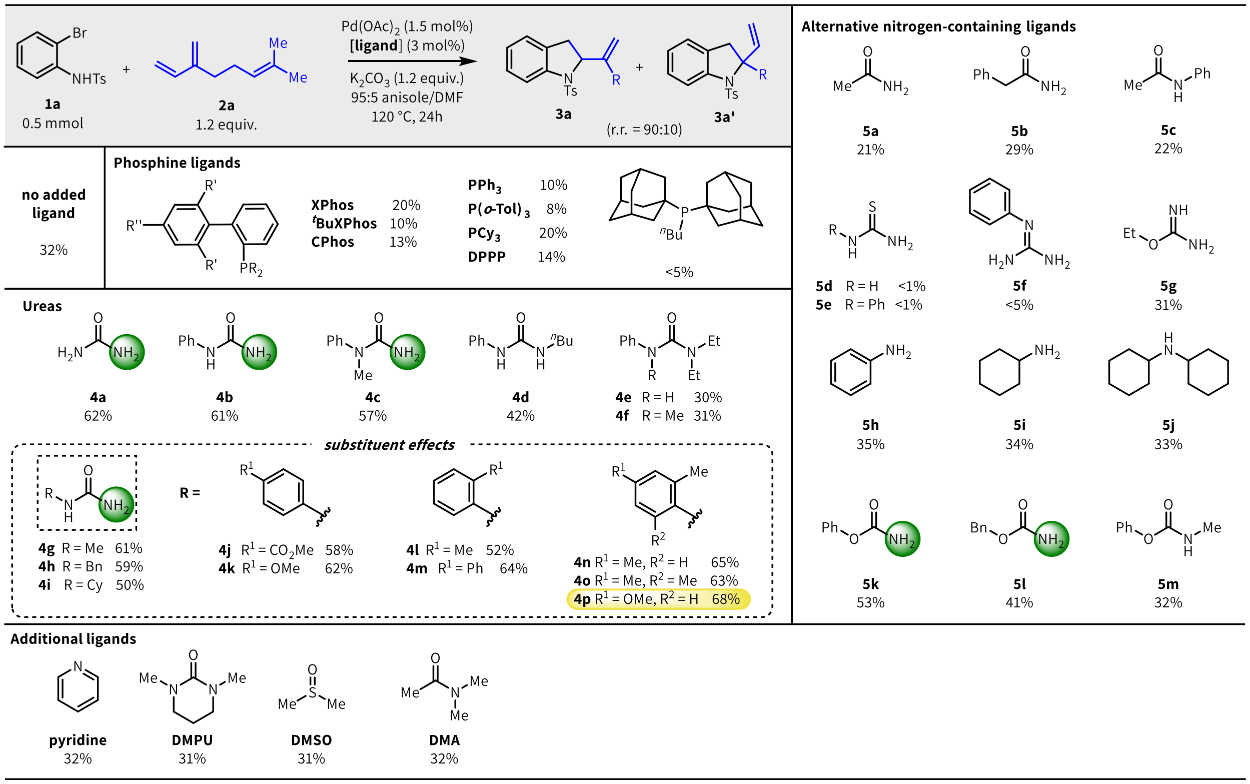

General procedure for the ligand structure-reactivity relationship studies: Bromoaniline **1a** (163 mg, 0.500 mmol, 1.0 equiv.), myrcene **2a** (103 uL, 0.600 mmol, 1.2 equiv.), ligand (0.015 mmol, 0.03 equiv.), potassium carbonate (83 mg, 0.600 mmol, 1.2 equiv.), and palladium acetate (1.7 mg, 0.0075 mmol, 0.015 equiv.) were weighed out in the above-mentioned order into a 1-dram vial equipped with a stir bar and a cap with a silicone septum. The vial was then placed under nitrogen atmosphere and charged with 1 mL of freshly degassed anisole/dimethylformamide (95:5) solvent mixture. The reaction was stirred at 120 °C for 24 hours. After cooling to room temperature, regioisomeric ratios were determined by HPLC analysis of the crude reaction mixture and the reaction mixture was filtered with ethyl acetate through cotton plug. The solvents were removed under reduced pressure and the crude mixture was purified by flash column chromatography (silica, hexanes/ethyl acetate 19:1) to obtain products **3a** and **3a'** as a mixture.

2-(6-methylhepta-1,5-dien-2-yl)-*N*-tosylindoline (3a)

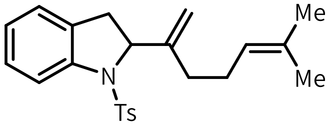

^{1}H NMR (400 MHz, $CDCl_3$) δ 7.68 (d, J = 8.1 Hz, 1H), 7.58 (d, J = 8.2 Hz, 2H), 7.23 – 7.15 (m, 3H), 7.04 – 6.97 (m, 2H), 5.13 (s, 1H), 5.09 (dd, J = 9.2, 4.5 Hz, 1H), 4.89 (s, 1H), 4.68 (dd, J = 10.2, 3.4 Hz, 1H), 2.97 (dd, J = 16.2, 10.2 Hz, 1H), 2.68 (dd, J = 16.2, 3.5 Hz, 1H), 2.35 (s, 3H), 2.20 – 2.05 (m, 3H), 1.99 (dt, J = 15.6, 7.6 Hz, 1H), 1.67 (s, 3H), 1.59 (s, 3H).

^{13}C NMR (100 MHz, $CDCl_3$) δ 148.5, 143.9, 142.2, 135.3, 132.0, 131.6, 129.6, 127.8, 127.3, 125.0, 124.5, 124.0, 116.7, 110.6, 66.3, 34.9, 31.3, 26.3, 25.8, 21.6, 17.8.

HRMS (ESI) *m/z* calculated for $C_{23}H_{28}NO_2S$ $[M+H]^+$: 382.1841, found 382.1822.

2-(4-methylpent-3-en-1-yl)-*N*-tosyl-2-vinylindoline (3a')

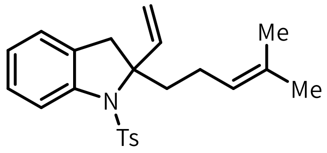

^1H NMR (400 MHz, $CDCl_3$) δ 7.77 (d, *J* = 8.3 Hz, 2H), 7.47 (d, *J* = 8.2 Hz, 1H), 7.21 (d, *J* = 7.9 Hz, 2H), 7.13 (t, *J* = 7.9 Hz, 1H), 7.09 (d, *J* = 6.9 Hz, 1H), 6.93 (td, *J* = 7.4, 0.8 Hz, 1H), 6.22 (dd, *J* = 17.5, 10.8 Hz, 1H), 5.26 (d, *J* = 17.5 Hz, 1H), 5.18 (d, *J* = 10.8 Hz, 1H), 5.12 (d, *J* = 7.0 Hz, 1H), 3.18 (d, *J* = 16.2 Hz, 1H), 3.09 (d, *J* = 16.2 Hz, 1H), 2.37 (s, 3H), 2.14 – 1.91 (m, 3H), 1.66 (s, 3H), 1.55 (s, 3H).

^{13}C NMR (100 MHz, $CDCl_3$) δ 143.5, 142.3, 140.5, 138.8, 132.3, 129.5, 128.7, 127.8, 127.4, 124.7, 123.7, 122.9, 114.9, 114.1, 74.8, 40.9, 39.2, 25.8, 23.0, 21.6, 17.8.

HRMS (ESI) *m/z* calculated for $C_{23}H_{28}NO_2S$ $[M+H]^+$: 382.1841, found 382.1840.

No Added Ligand

Entry 1: No ligand. General procedure for ligand screening was followed. **1a** (163 mg, 0.500 mmol, 1.0 equiv.), **2a** (103 uL, 0.600 mmol, 1.2 equiv.), K_2CO_3 (83 mg, 0.600 mmol, 1.2 equiv.), $Pd(OAc)_2$ (1.7 mg, 0.0075 mmol, 0.015 equiv.), and 95:5 anisole/DMF (1.0 mL, 0.5M) were used. The crude material was purified by flash column chromatography (silica, hexanes/ethyl acetate 19:1) to obtain products **3a** and **3a'** in 90:10 r.r. (determined by HPLC).

<u>Run 1</u>: (63 mg, 0.165 mmol, 33%)
<u>Run 2</u>: (59 mg, 0.155 mmol, 31%)
<u>Run 3</u>: (61 mg, 0.160 mmol, 32%)
Average: 32% yield

Phosphine Ligands

Entry 2: XPhos. General procedure for ligand screening was followed. **1a** (163 mg, 0.500 mmol, 1.0 equiv.), **2a** (103 uL, 0.600 mmol, 1.2 equiv.), K_2CO_3 (83 mg, 0.600 mmol, 1.2 equiv.), XPhos (7.2 mg, 0.015 mmol, 0.03 equiv.), $Pd(OAc)_2$ (1.7 mg, 0.0075 mmol, 0.015 equiv.), and 95:5 anisole/DMF (1.0 mL, 0.5M) were used. The crude material was purified by flash column chromatography (silica, hexanes/ethyl acetate 19:1) to obtain products **3a** and **3a'** in 90:10 r.r. (determined by HPLC).

<u>Run 1</u>: (36 mg, 0.094 mmol, 19%)
<u>Run 2</u>: (42 mg, 0.110 mmol, 22%)
<u>Run 3</u>: (34 mg, 0.089 mmol, 18%)
Average: 20% yield

Entry 3: *t*BuXPhos. General procedure for ligand screening was followed. **1a** (163 mg, 0.500 mmol, 1.0 equiv.), **2a** (103 uL, 0.600 mmol, 1.2 equiv.), K_2CO_3 (83 mg, 0.600 mmol, 1.2 equiv.), *t*BuXPhos (6.4 mg, 0.015 mmol, 0.03 equiv.), $Pd(OAc)_2$ (1.7 mg, 0.0075 mmol, 0.015 equiv.), and 95:5 anisole/DMF (1.0 mL, 0.5M) were used. The crude material was purified by flash column chromatography (silica, hexanes/ethyl acetate 19:1) to obtain products **3a** and **3a'** in 90:10 r.r. (determined by HPLC).

Run 1: (17 mg, 0.045 mmol, 9%)
Run 2: (18 mg, 0.047 mmol, 9%)
Run 3: (23 mg, 0.060 mmol, 12%)
Average: 10% yield

Entry 4: CPhos. General procedure for ligand screening was followed. **1a** (163 mg, 0.500 mmol, 1.0 equiv.), **2a** (103 uL, 0.600 mmol, 1.2 equiv.), K_2CO_3 (83 mg, 0.600 mmol, 1.2 equiv.), CPhos (6.5 mg, 0.015 mmol, 0.03 equiv.), $Pd(OAc)_2$ (1.7 mg, 0.0075 mmol, 0.015 equiv.), and 95:5 anisole/DMF (1.0 mL, 0.5M) were used. The crude material was purified by flash column chromatography (silica, hexanes/ethyl acetate 19:1) to obtain products **3a** and **3a'** in 91:9 r.r. (determined by HPLC).

Run 1: (21 mg, 0.055 mmol, 11%)
Run 2: (29 mg, 0.076 mmol, 15%)
Run 3: (27 mg, 0.071 mmol, 14%)
Average: 13% yield

Entry 5: Triphenylphosphine. General procedure for ligand screening was followed. **1a** (163 mg, 0.500 mmol, 1.0 equiv.), **2a** (103 uL, 0.600 mmol, 1.2 equiv.), K_2CO_3 (83 mg, 0.600 mmol, 1.2 equiv.), PPh_3 (3.9 mg, 0.015 mmol, 0.03 equiv.), $Pd(OAc)_2$ (1.7 mg, 0.0075 mmol, 0.015 equiv.), and 95:5 anisole/DMF (1.0 mL, 0.5M) were used. The crude material was purified by flash column chromatography (silica, hexanes/ethyl acetate 19:1) to obtain products **3a** and **3a'** in 91:9 r.r. (determined by HPLC).

Run 1: (17 mg, 0.045 mmol, 9%)
Run 2: (15 mg, 0.039 mmol, 8%)
Run 3: (25 mg, 0.066 mmol, 13%)
Average: 10% yield

Entry 6: Tri(o-tolyl)-phosphine. General procedure for ligand screening was followed. **1a** (163 mg, 0.500 mmol, 1.0 equiv.), **2a** (103 uL, 0.600 mmol, 1.2 equiv.), K_2CO_3 (83 mg, 0.600 mmol, 1.2 equiv.), P(*o*-Tol)$_3$ (4.6 mg, 0.015 mmol, 0.03 equiv.), $Pd(OAc)_2$ (1.7 mg, 0.0075 mmol, 0.015 equiv.), and 95:5 anisole/DMF (1.0 mL, 0.5M) were used. The crude material was purified by flash column chromatography (silica, hexanes/ethyl acetate 19:1) to obtain products **3a** and **3a'** in 91:9 r.r. (determined by HPLC).

Run 1: (12 mg, 0.030 mmol, 6%)
Run 2: (14 mg, 0.035 mmol, 7%)
Run 3: (21 mg, 0.055 mmol, 11%)
Average: 8% yield

Entry 7: Tricyclohexylphosphine. General procedure for ligand screening was followed. **1a** (163 mg, 0.500 mmol, 1.0 equiv.), **2a** (103 uL, 0.600 mmol, 1.2 equiv.), K_2CO_3 (83 mg, 0.600 mmol, 1.2 equiv.), PCy_3 (4.2 mg, 0.015 mmol, 0.03 equiv.), $Pd(OAc)_2$ (1.7 mg, 0.0075 mmol, 0.015 equiv.), and 95:5 anisole/DMF (1.0 mL, 0.5M) were used. The crude material was purified by flash column chromatography (silica, hexanes/ethyl acetate 19:1) to obtain products **3a** and **3a'** in 89:11 r.r. (determined by HPLC).

Run 1: (36 mg, 0.094 mmol, 19%)
Run 2: (38 mg, 0.100 mmol, 20%)
Run 3: (39 mg, 0.102 mmol, 20%)
Average: 20% yield

Entry 8: 1,3-Bis(diphenylphosphino)propane. General procedure for ligand screening was followed. **1a** (163 mg, 0.500 mmol, 1.0 equiv.), **2a** (103 uL, 0.600 mmol, 1.2 equiv.), K_2CO_3 (83 mg, 0.600 mmol, 1.2 equiv.), DPPP (6.2 mg, 0.015 mmol, 0.03 equiv.), $Pd(OAc)_2$ (1.7 mg, 0.0075 mmol, 0.015 equiv.), and 95:5 anisole/DMF (1.0 mL, 0.5M) were used. The crude material was purified by flash column chromatography (silica, hexanes/ethyl acetate 19:1) to obtain products **3a** and **3a'** in 92:8 r.r. (determined by HPLC).

Run 1: (23 mg, 0.060 mmol, 12%)
Run 2: (30 mg, 0.080 mmol, 16%)
Run 3: (28 mg, 0.075 mmol, 15%)
Average: 14% yield

Entry 9: Di(1-adamantyl)-n-butylphosphine. General procedure for ligand screening was followed. **1a** (163 mg, 0.500 mmol, 1.0 equiv.), **2a** (103 uL, 0.600 mmol, 1.2 equiv.), K_2CO_3 (83 mg, 0.600 mmol, 1.2 equiv.), $PAd_2{}^{n}Bu$ (5.4 mg, 0.015 mmol, 0.03 equiv.), $Pd(OAc)_2$ (1.7 mg, 0.0075 mmol, 0.015 equiv.), and 95:5 anisole/DMF (1.0 mL, 0.5M) were used. The yield of **3a** + **3a'** was determined by HPLC analysis of the crude reaction mixture.

Run 1: <5%
Run 2: <5%
Run 3: <5%
Average: <5% yield

Ureas

Entry 10: Urea 4a. General procedure for ligand screening was followed. **1a** (163 mg, 0.500 mmol, 1.0 equiv.), **2a** (103 uL, 0.600 mmol, 1.2 equiv.), K_2CO_3 (83 mg, 0.600 mmol, 1.2 equiv.), urea **4a** (0.9 mg, 0.015 mmol, 0.03 equiv.), $Pd(OAc)_2$ (1.7 mg, 0.0075 mmol, 0.015 equiv.), and 95:5 anisole/DMF (1.0 mL, 0.5M) were used. The crude material was purified by flash column chromatography (silica, hexanes/ethyl acetate 19:1) to obtain products **3a** and **3a'** in 90:10 r.r. (determined by HPLC).

Run 1: (123 mg, 0.322 mmol, 64%)
Run 2: (113 mg, 0.296 mmol, 59%)
Run 3: (120 mg, 0.314 mmol, 63%)
Average: 62% yield

Entry 11: Urea 4b. General procedure for ligand screening was followed. **1a** (163 mg, 0.500 mmol, 1.0 equiv.), **2a** (103 uL, 0.600 mmol, 1.2 equiv.), K_2CO_3 (83 mg, 0.600 mmol, 1.2 equiv.), urea **4b** (2.0 mg, 0.015 mmol, 0.03 equiv.), $Pd(OAc)_2$ (1.7 mg, 0.0075 mmol, 0.015 equiv.), and 95:5 anisole/DMF (1.0 mL, 0.5M) were used. The crude material was purified by flash column chromatography (silica, hexanes/ethyl acetate 19:1) to obtain products **3a** and **3a'** in 90:10 r.r. (determined by HPLC).

Run 1: (114 mg, 0.299 mmol, 60%)
Run 2: (112 mg, 0.295 mmol, 59%)
Run 3: (122 mg, 0.321 mmol, 64%)
Average: 61% yield

Entry 12: Urea 4c. General procedure for ligand screening was followed. **1a** (163 mg, 0.500 mmol, 1.0 equiv.), **2a** (103 uL, 0.600 mmol, 1.2 equiv.), K_2CO_3 (83 mg, 0.600 mmol, 1.2 equiv.), urea **4c** (2.3 mg, 0.015 mmol, 0.03 equiv.), $Pd(OAc)_2$ (1.7 mg, 0.0075 mmol, 0.015 equiv.), and 95:5 anisole/DMF (1.0 mL, 0.5M) were used. The crude material was purified by flash column

chromatography (silica, hexanes/ethyl acetate 19:1) to obtain products **3a** and **3a'** in 89:11 r.r. (determined by HPLC).

Run 1: (112 mg, 0.295 mmol, 59%)
Run 2: (109 mg, 0.286 mmol, 57%)
Run 3: (103 mg, 0.270 mmol, 54%)
Average: 57% yield

Entry 13: Urea 4d. General procedure for ligand screening was followed. **1a** (163 mg, 0.500 mmol, 1.0 equiv.), **2a** (103 uL, 0.600 mmol, 1.2 equiv.), K_2CO_3 (83 mg, 0.600 mmol, 1.2 equiv.), urea **4d** (2.9 mg, 0.015 mmol, 0.03 equiv.), $Pd(OAc)_2$ (1.7 mg, 0.0075 mmol, 0.015 equiv.), and 95:5 anisole/DMF (1.0 mL, 0.5M) were used. The crude material was purified by flash column chromatography (silica, hexanes/ethyl acetate 19:1) to obtain products **3a** and **3a'** in 91:9 r.r. (determined by HPLC).

Run 1: (76 mg, 0.200 mmol, 40%)
Run 2: (77 mg, 0.202 mmol, 40%)
Run 3: (85 mg, 0.223 mmol, 45%)
Average: 42% yield

Entry 14: Urea 4e. General procedure for ligand screening was followed. **1a** (163 mg, 0.500 mmol, 1.0 equiv.), **2a** (103 uL, 0.600 mmol, 1.2 equiv.), K_2CO_3 (83 mg, 0.600 mmol, 1.2 equiv.), urea **4e** (2.9 mg, 0.015 mmol, 0.03 equiv.), $Pd(OAc)_2$ (1.7 mg, 0.0075 mmol, 0.015 equiv.), and 95:5 anisole/DMF (1.0 mL, 0.5M) were used. The crude material was purified by flash column chromatography (silica, hexanes/ethyl acetate 19:1) to obtain products **3a** and **3a'** in 91:9 r.r. (determined by HPLC).

Run 1: (61 mg, 0.160 mmol, 32%)
Run 2: (52 mg, 0.136 mmol, 27%)
Run 3: (57 mg, 0.150 mmol, 30%)
Average: 30% yield

Entry 15: Urea 4f. General procedure for ligand screening was followed. **1a** (163 mg, 0.500 mmol, 1.0 equiv.), **2a** (103 uL, 0.600 mmol, 1.2 equiv.), K_2CO_3 (83 mg, 0.600 mmol, 1.2 equiv.), urea **4f** (3.1 mg, 0.015 mmol, 0.03 equiv.), $Pd(OAc)_2$ (1.7 mg, 0.0075 mmol, 0.015 equiv.), and 95:5 anisole/DMF (1.0 mL, 0.5M) were used. The crude material was purified by flash column chromatography (silica, hexanes/ethyl acetate 19:1) to obtain products **3a** and **3a'** in 89:11 r.r. (determined by HPLC).

Run 1: (61 mg, 0.160 mmol, 32%)
Run 2: (53 mg, 0.139 mmol, 28%)
Run 3: (61 mg, 0.160 mmol, 32%)
Average: 31% yield

Urea Substituent Effects

Entry 16: Urea 4g. General procedure for ligand screening was followed. **1a** (163 mg, 0.500 mmol, 1.0 equiv.), **2a** (103 uL, 0.600 mmol, 1.2 equiv.), K_2CO_3 (83 mg, 0.600 mmol, 1.2 equiv.), urea **4g** (1.1 mg, 0.015 mmol, 0.03 equiv.), $Pd(OAc)_2$ (1.7 mg, 0.0075 mmol, 0.015 equiv.), and 95:5 anisole/DMF (1.0 mL, 0.5M) were used. The crude material was purified by flash column chromatography (silica, hexanes/ethyl acetate 19:1) to obtain products **3a** and **3a'** in 89:11 r.r. (determined by HPLC).

Run 1: (116 mg, 0.304 mmol, 61%)
Run 2: (116 mg, 0.304 mmol, 61%)
Run 3: (114 mg, 0.299 mmol, 60%)
Average: 61% yield

Entry 17: Urea 4h. General procedure for ligand screening was followed. **1a** (163 mg, 0.500 mmol, 1.0 equiv.), **2a** (103 uL, 0.600 mmol, 1.2 equiv.), K_2CO_3 (83 mg, 0.600 mmol, 1.2 equiv.), urea **4h** (2.3 mg, 0.015 mmol, 0.03 equiv.), $Pd(OAc)_2$ (1.7 mg, 0.0075 mmol, 0.015 equiv.), and 95:5 anisole/DMF (1.0 mL, 0.5M) were used. The crude material was purified by flash column chromatography (silica, hexanes/ethyl acetate 19:1) to obtain products **3a** and **3a'** in 91:9 r.r. (determined by HPLC).

Run 1: (115 mg, 0.301 mmol, 60%)
Run 2: (111 mg, 0.291 mmol, 58%)
Run 3: (114 mg, 0.299 mmol, 60%)
Average: 59% yield

Entry 18: Urea 4i. General procedure for ligand screening was followed. **1a** (163 mg, 0.500 mmol, 1.0 equiv.), **2a** (103 uL, 0.600 mmol, 1.2 equiv.), K_2CO_3 (83 mg, 0.600 mmol, 1.2 equiv.), urea **4i** (2.1 mg, 0.015 mmol, 0.03 equiv.), $Pd(OAc)_2$ (1.7 mg, 0.0075 mmol, 0.015 equiv.), and 95:5 anisole/DMF (1.0 mL, 0.5M) were used. The crude material was purified by flash column chromatography (silica, hexanes/ethyl acetate 19:1) to obtain products **3a** and **3a'** in 90:10 r.r. (determined by HPLC).

Run 1: (99 mg, 0.260 mmol, 52%)
Run 2: (90 mg, 0.236 mmol, 47%)
Run 3: (95 mg, 0.249 mmol, 50%)
Average: 50% yield

Entry 19: Urea 4j. General procedure for ligand screening was followed. **1a** (163 mg, 0.500 mmol, 1.0 equiv.), **2a** (103 uL, 0.600 mmol, 1.2 equiv.), K_2CO_3 (83 mg, 0.600 mmol, 1.2 equiv.), urea **4j** (2.9 mg, 0.015 mmol, 0.03 equiv.), $Pd(OAc)_2$ (1.7 mg, 0.0075 mmol, 0.015 equiv.), and 95:5 anisole/DMF (1.0 mL, 0.5M) were used. The crude material was purified by flash column chromatography (silica, hexanes/ethyl acetate 19:1) to obtain products **3a** and **3a'** in 90:10 r.r. (determined by HPLC).

Run 1: (107 mg, 0.280 mmol, 56%)
Run 2: (115 mg, 0.301 mmol, 60%)
Run 3: (111 mg, 0.291 mmol, 58%)
Average: 58% yield

Entry 20: Urea 4k. General procedure for ligand screening was followed. **1a** (163 mg, 0.500 mmol, 1.0 equiv.), **2a** (103 uL, 0.600 mmol, 1.2 equiv.), K_2CO_3 (83 mg, 0.600 mmol, 1.2 equiv.), urea **4k** (2.5 mg, 0.015 mmol, 0.03 equiv.), $Pd(OAc)_2$ (1.7 mg, 0.0075 mmol, 0.015 equiv.), and 95:5 anisole/DMF (1.0 mL, 0.5M) were used. The crude material was purified by flash column chromatography (silica, hexanes/ethyl acetate 19:1) to obtain products **3a** and **3a'** in 90:10 r.r. (determined by HPLC).

Run 1: (122 mg, 0.320 mmol, 64%)
Run 2: (116 mg, 0.304 mmol, 61%)
Run 3: (118 mg, 0.309 mmol, 62%)
Average: 62% yield

Entry 21: Urea 4l. General procedure for ligand screening was followed. **1a** (163 mg, 0.500 mmol, 1.0 equiv.), **2a** (103 uL, 0.600 mmol, 1.2 equiv.), K_2CO_3 (83 mg, 0.600 mmol, 1.2 equiv.), urea **4l** (2.3 mg, 0.015 mmol, 0.03 equiv.), $Pd(OAc)_2$ (1.7 mg, 0.0075 mmol, 0.015 equiv.), and 95:5 anisole/DMF (1.0 mL, 0.5M) were used. The crude material was purified by flash column chromatography (silica, hexanes/ethyl acetate 19:1) to obtain products **3a** and **3a'** in 90:10 r.r. (determined by HPLC).

Run 1: (95 mg, 0.249 mmol, 50%)
Run 2: (103 mg, 0.270 mmol, 54%)
Run 3: (99 mg, 0.260 mmol, 52%)
Average: 52% yield

Entry 22: Urea 4m. General procedure for ligand screening was followed. **1a** (163 mg, 0.500 mmol, 1.0 equiv.), **2a** (103 uL, 0.600 mmol, 1.2 equiv.), K_2CO_3 (83 mg, 0.600 mmol, 1.2 equiv.), urea **4m** (3.2 mg, 0.015 mmol, 0.03 equiv.), $Pd(OAc)_2$ (1.7 mg, 0.0075 mmol, 0.015 equiv.), and 95:5 anisole/DMF (1.0 mL, 0.5M) were used. The crude material was purified by flash column chromatography (silica, hexanes/ethyl acetate 19:1) to obtain products **3a** and **3a'** in 90:10 r.r. (determined by HPLC).

Run 1: (126 mg, 0.330 mmol, 66%)
Run 2: (120 mg, 0.315 mmol, 63%)
Run 3: (118 mg, 0.309 mmol, 62%)
Average: 64% yield

Entry 23: Urea 4n. General procedure for ligand screening was followed. **1a** (163 mg, 0.500 mmol, 1.0 equiv.), **2a** (103 uL, 0.600 mmol, 1.2 equiv.), K_2CO_3 (83 mg, 0.600 mmol, 1.2 equiv.), urea **4n** (2.5 mg, 0.015 mmol, 0.03 equiv.), $Pd(OAc)_2$ (1.7 mg, 0.0075 mmol, 0.015 equiv.), and 95:5 anisole/DMF (1.0 mL, 0.5M) were used. The crude material was purified by flash column chromatography (silica, hexanes/ethyl acetate 19:1) to obtain products **3a** and **3a'** in 90:10 r.r. (determined by HPLC).

Run 1: (128 mg, 0.336 mmol, 67%)
Run 2: (124 mg, 0.325 mmol, 65%)
Run 3: (122 mg, 0.320 mmol, 64%)
Average: 65% yield

Entry 24: Urea 4o. General procedure for ligand screening was followed. **1a** (163 mg, 0.500 mmol, 1.0 equiv.), **2a** (103 uL, 0.600 mmol, 1.2 equiv.), K_2CO_3 (83 mg, 0.600 mmol, 1.2 equiv.), urea **4o** (2.7 mg, 0.015 mmol, 0.03 equiv.), $Pd(OAc)_2$ (1.7 mg, 0.0075 mmol, 0.015 equiv.), and 95:5 anisole/DMF (1.0 mL, 0.5M) were used. The crude material was purified by flash column chromatography (silica, hexanes/ethyl acetate 19:1) to obtain products **3a** and **3a'** in 90:10 r.r. (determined by HPLC).

Run 1: (126 mg, 0.330 mmol, 66%)
Run 2: (118 mg, 0.309 mmol, 62%)
Run 3: (116 mg, 0.304 mmol, 61%)
Average: 63% yield

Entry 25: Urea 4p. General procedure for ligand screening was followed. **1a** (163 mg, 0.500 mmol, 1.0 equiv.), **2a** (103 uL, 0.600 mmol, 1.2 equiv.), K_2CO_3 (83 mg, 0.600 mmol, 1.2 equiv.), urea **4p** (2.7 mg, 0.015 mmol, 0.03 equiv.), $Pd(OAc)_2$ (1.7 mg, 0.0075 mmol, 0.015 equiv.), and 95:5 anisole/DMF (1.0 mL, 0.5M) were used. The crude material was purified by flash column

chromatography (silica, hexanes/ethyl acetate 19:1) to obtain products **3a** and **3a'** in 90:10 r.r. (determined by HPLC).

Run 1: (126 mg, 0.330 mmol, 66%)
Run 2: (132 mg, 0.346 mmol, 69%)
Run 3: (131 mg, 0.343 mmol, 69%)
Average: 68% yield

Alternative nitrogen-containing ligands

Entry 26: Acetamide 5a. General procedure for ligand screening was followed. **1a** (163 mg, 0.500 mmol, 1.0 equiv.), **2a** (103 uL, 0.600 mmol, 1.2 equiv.), K_2CO_3 (83 mg, 0.600 mmol, 1.2 equiv.), acetamide **5a** (0.9 mg, 0.015 mmol, 0.03 equiv.), $Pd(OAc)_2$ (1.7 mg, 0.0075 mmol, 0.015 equiv.), and 95:5 anisole/DMF (1.0 mL, 0.5M) were used. The crude material was purified by flash column chromatography (silica, hexanes/ethyl acetate 19:1) to obtain products **3a** and **3a'** in 89:11 r.r. (determined by HPLC).

Run 1: (36 mg, 0.094 mmol, 19%)
Run 2: (44 mg, 0.115 mmol, 23%)
Run 3: (40 mg, 0.105 mmol, 21%)
Average: 21% yield

Entry 27: 2-Phenylacetamide 5b. General procedure for ligand screening was followed. **1a** (163 mg, 0.500 mmol, 1.0 equiv.), **2a** (103 uL, 0.600 mmol, 1.2 equiv.), K_2CO_3 (83 mg, 0.600 mmol, 1.2 equiv.), acetamide **5b** (2.0 mg, 0.015 mmol, 0.03 equiv.), $Pd(OAc)_2$ (1.7 mg, 0.0075 mmol, 0.015 equiv.), and 95:5 anisole/DMF (1.0 mL, 0.5M) were used. The crude material was purified by flash column chromatography (silica, hexanes/ethyl acetate 19:1) to obtain products **3a** and **3a'** in 91:9 r.r. (determined by HPLC).

Run 1: (56 mg, 0.145 mmol, 29%)
Run 2: (60 mg, 0.155 mmol, 31%)
Run 3: (49 mg, 0.130 mmol, 26%)
Average: 29% yield

Entry 28: *N*-Phenylacetamide 5c. General procedure for ligand screening was followed. **1a** (163 mg, 0.500 mmol, 1.0 equiv.), **2a** (103 uL, 0.600 mmol, 1.2 equiv.), K_2CO_3 (83 mg, 0.600 mmol, 1.2 equiv.), acetamide **5c** (2.0 mg, 0.015 mmol, 0.03 equiv.), $Pd(OAc)_2$ (1.7 mg, 0.0075 mmol, 0.015 equiv.), and 95:5 anisole/DMF (1.0 mL, 0.5M) were used. The crude material was purified by flash column chromatography (silica, hexanes/ethyl acetate 19:1) to obtain products **3a** and **3a'** in 91:9 r.r. (determined by HPLC).

Run 1: (42 mg, 0.110 mmol, 22%)
Run 2: (44 mg, 0.115 mmol, 23%)
Run 3: (42 mg, 0.110 mmol, 22%)
Average: 22% yield

Entry 29: Thiourea 5d. General procedure for ligand screening was followed. **1a** (163 mg, 0.500 mmol, 1.0 equiv.), **2a** (103 uL, 0.600 mmol, 1.2 equiv.), K_2CO_3 (83 mg, 0.600 mmol, 1.2 equiv.), thiourea **5d** (1.1 mg, 0.015 mmol, 0.03 equiv.), $Pd(OAc)_2$ (1.7 mg, 0.0075 mmol, 0.015 equiv.),

and 95:5 anisole/DMF (1.0 mL, 0.5M) were used. The yield of **3a** + **3a'** was determined by HPLC analysis of the crude reaction mixture.

Run 1: <1%
Run 2: <1%
Run 3: <1%
Average: <1% yield

Entry 29: *N*-Phenylthiourea 5e. General procedure for ligand screening was followed. **1a** (163 mg, 0.500 mmol, 1.0 equiv.), **2a** (103 uL, 0.600 mmol, 1.2 equiv.), K_2CO_3 (83 mg, 0.600 mmol, 1.2 equiv.), *N*-phenylthiourea **5e** (2.3 mg, 0.015 mmol, 0.03 equiv.), $Pd(OAc)_2$ (1.7 mg, 0.0075 mmol, 0.015 equiv.), and 95:5 anisole/DMF (1.0 mL, 0.5M) were used. The yield of **3a** + **3a'** was determined by HPLC analysis of the crude reaction mixture.

Run 1: <1%
Run 2: <1%
Run 3: <1%
Average: <1% yield

Entry 30: Phenylguanidine 5f. General procedure for ligand screening was followed. **1a** (163 mg, 0.500 mmol, 1.0 equiv.), **2a** (103 uL, 0.600 mmol, 1.2 equiv.), K_2CO_3 (83 mg, 0.600 mmol, 1.2 equiv.), phenylguanidine carbonate (3.1 mg, 0.015 mmol, 0.03 equiv.), $Pd(OAc)_2$ (1.7 mg, 0.0075 mmol, 0.015 equiv.), and 95:5 anisole/DMF (1.0 mL, 0.5M) were used. The yield of **3a** + **3a'** was determined by HPLC analysis of the crude reaction mixture.

Run 1: <5%
Run 2: <5%
Run 3: <5%
Average: <5% yield

Entry 31: Ethyl carbamimidate 5g. General procedure for ligand screening was followed. **1a** (163 mg, 0.500 mmol, 1.0 equiv.), **2a** (103 uL, 0.600 mmol, 1.2 equiv.), K_2CO_3 (83 mg, 0.600 mmol, 1.2 equiv.), ethyl Imidocarbamate hydrochloride (1.9 mg, 0.015 mmol, 0.03 equiv.), $Pd(OAc)_2$ (1.7 mg, 0.0075 mmol, 0.015 equiv.), and 95:5 anisole/DMF (1.0 mL, 0.5M) were used. The crude material was purified by flash column chromatography (silica, hexanes/ethyl acetate 19:1) to obtain products **3a** and **3a'** in 90:10 r.r. (determined by HPLC).

Run 1: (65 mg, 0.170 mmol, 34%)
Run 2: (57 mg, 0.150 mmol, 30%)
Run 3: (55 mg, 0.145 mmol, 29%)
Average: 31% yield

Entry 32: Aniline 5h. General procedure for ligand screening was followed. **1a** (163 mg, 0.500 mmol, 1.0 equiv.), **2a** (103 uL, 0.600 mmol, 1.2 equiv.), K_2CO_3 (83 mg, 0.600 mmol, 1.2 equiv.), aniline **5h** (1.4 mg, 0.015 mmol, 0.03 equiv.), $Pd(OAc)_2$ (1.7 mg, 0.0075 mmol, 0.015 equiv.), and 95:5 anisole/DMF (1.0 mL, 0.5M) were used. The crude material was purified by flash column chromatography (silica, hexanes/ethyl acetate 19:1) to obtain products **3a** and **3a'** in 91:9 r.r. (determined by HPLC).

Run 1: (68 mg, 0.180 mmol, 36%)
Run 2: (66 mg, 0.175 mmol, 35%)
Run 3: (63 mg, 0.165 mmol, 33%)

Average: 35% yield

Entry 33: Cyclohexylamine 5i. General procedure for ligand screening was followed. **1a** (163 mg, 0.500 mmol, 1.0 equiv.), **2a** (103 uL, 0.600 mmol, 1.2 equiv.), K_2CO_3 (83 mg, 0.600 mmol, 1.2 equiv.), cyclohexylamine **5i** (1.5 mg, 0.015 mmol, 0.03 equiv.), $Pd(OAc)_2$ (1.7 mg, 0.0075 mmol, 0.015 equiv.), and 95:5 anisole/DMF (1.0 mL, 0.5M) were used. The crude material was purified by flash column chromatography (silica, hexanes/ethyl acetate 19:1) to obtain products **3a** and **3a'** in 92:8 r.r. (determined by HPLC).

Run 1: (66 mg, 0.175 mmol, 35%)
Run 2: (64 mg, 0.170 mmol, 34%)
Run 3: (61 mg, 0.160 mmol, 32%)
Average: 34% yield

Entry 34: Dicyclohexylamine 5j. General procedure for ligand screening was followed. **1a** (163 mg, 0.500 mmol, 1.0 equiv.), **2a** (103 uL, 0.600 mmol, 1.2 equiv.), K_2CO_3 (83 mg, 0.600 mmol, 1.2 equiv.), dicyclohexylamine **5j** (2.7 mg, 0.015 mmol, 0.03 equiv.), $Pd(OAc)_2$ (1.7 mg, 0.0075 mmol, 0.015 equiv.), and 95:5 anisole/DMF (1.0 mL, 0.5M) were used. The crude material was purified by flash column chromatography (silica, hexanes/ethyl acetate 19:1) to obtain products **3a** and **3a'** in 90:10 r.r. (determined by HPLC).

Run 1: (64 mg, 0.170 mmol, 34%)
Run 2: (64 mg, 0.170 mmol, 34%)
Run 3: (60 mg, 0.155 mmol, 31%)
Average: 33% yield

Entry 35: Phenyl carbamate 5k. General procedure for ligand screening was followed. **1a** (163 mg, 0.500 mmol, 1.0 equiv.), **2a** (103 uL, 0.600 mmol, 1.2 equiv.), K_2CO_3 (83 mg, 0.600 mmol, 1.2 equiv.), phenyl carbamate **5k** (2.1 mg, 0.015 mmol, 0.03 equiv.), $Pd(OAc)_2$ (1.7 mg, 0.0075 mmol, 0.015 equiv.), and 95:5 anisole/DMF (1.0 mL, 0.5M) were used. The crude material was purified by flash column chromatography (silica, hexanes/ethyl acetate 19:1) to obtain products **3a** and **3a'** in 90:10 r.r. (determined by HPLC).

Run 1: (101 mg, 0.265 mmol, 53%)
Run 2: (100 mg, 0.260 mmol, 52%)
Run 3: (105 mg, 0.275 mmol, 55%)
Average: 53% yield

Entry 36: Benzyl carbamate 5l. General procedure for ligand screening was followed. **1a** (163 mg, 0.500 mmol, 1.0 equiv.), **2a** (103 uL, 0.600 mmol, 1.2 equiv.), K_2CO_3 (83 mg, 0.600 mmol, 1.2 equiv.), benzyl carbamate **5l** (2.3 mg, 0.015 mmol, 0.03 equiv.), $Pd(OAc)_2$ (1.7 mg, 0.0075 mmol, 0.015 equiv.), and 95:5 anisole/DMF (1.0 mL, 0.5M) were used. The crude material was purified by flash column chromatography (silica, hexanes/ethyl acetate 19:1) to obtain products **3a** and **3a'** in 90:10 r.r. (determined by HPLC).

Run 1: (79 mg, 0.205 mmol, 41%)
Run 2: (81 mg, 0.210 mmol, 42%)
Run 3: (79 mg, 0.205 mmol, 41%)
Average: 41% yield

Entry 37: Phenyl methylcarbamate 5m. General procedure for ligand screening was followed. **1a** (163 mg, 0.500 mmol, 1.0 equiv.), **2a** (103 uL, 0.600 mmol, 1.2 equiv.), K_2CO_3 (83 mg, 0.600

mmol, 1.2 equiv.), phenyl methylcarbamate **5m** (2.3 mg, 0.015 mmol, 0.03 equiv.), $Pd(OAc)_2$ (1.7 mg, 0.0075 mmol, 0.015 equiv.), and 95:5 anisole/DMF (1.0 mL, 0.5M) were used. The crude material was purified by flash column chromatography (silica, hexanes/ethyl acetate 19:1) to obtain products **3a** and **3a’** in 91:9 r.r. (determined by HPLC).

Run 1: (65 mg, 0.170 mmol, 34%)
Run 2: (58 mg, 0.150 mmol, 30%)
Run 3: (62 mg, 0.160 mmol, 32%)
Average: 32% yield

Additional ligands

Entry 38: Pyridine. General procedure for ligand screening was followed. **1a** (163 mg, 0.500 mmol, 1.0 equiv.), **2a** (103 uL, 0.600 mmol, 1.2 equiv.), K_2CO_3 (83 mg, 0.600 mmol, 1.2 equiv.), pyridine (1.2 mg, 0.015 mmol, 0.03 equiv.), $Pd(OAc)_2$ (1.7 mg, 0.0075 mmol, 0.015 equiv.), and 95:5 anisole/DMF (1.0 mL, 0.5M) were used. The crude material was purified by flash column chromatography (silica, hexanes/ethyl acetate 19:1) to obtain products **3a** and **3a’** in 91:9 r.r. (determined by HPLC).

Run 1: (64 mg, 0.170 mmol, 34%)
Run 2: (56 mg, 0.145 mmol, 29%)
Average: 32% yield

Entry 39: *N,N′*-Dimethylpropylene urea (DMPU). General procedure for ligand screening was followed. **1a** (163 mg, 0.500 mmol, 1.0 equiv.), **2a** (103 uL, 0.600 mmol, 1.2 equiv.), K_2CO_3 (83 mg, 0.600 mmol, 1.2 equiv.), DMPU (1.9 mg, 0.015 mmol, 0.03 equiv.), $Pd(OAc)_2$ (1.7 mg, 0.0075 mmol, 0.015 equiv.), and 95:5 anisole/DMF (1.0 mL, 0.5M) were used. The crude material was purified by flash column chromatography (silica, hexanes/ethyl acetate 19:1) to obtain products **3a** and **3a’** in 91:9 r.r. (determined by HPLC).

Run 1: (58 mg, 0.150 mmol, 30%)
Run 2: (61 mg, 0.160 mmol, 32%)
Average: 31% yield

Entry 40: Dimethyl sulfoxide (DMSO). General procedure for ligand screening was followed. **1a** (163 mg, 0.500 mmol, 1.0 equiv.), **2a** (103 uL, 0.600 mmol, 1.2 equiv.), K_2CO_3 (83 mg, 0.600 mmol, 1.2 equiv.), DMSO (1.2 mg, 0.015 mmol, 0.03 equiv.), $Pd(OAc)_2$ (1.7 mg, 0.0075 mmol, 0.015 equiv.), and 95:5 anisole/DMF (1.0 mL, 0.5M) were used. The crude material was purified by flash column chromatography (silica, hexanes/ethyl acetate 19:1) to obtain products **3a** and **3a’** in 90:10 r.r. (determined by HPLC).

Run 1: (60 mg, 0.155 mmol, 31%)
Run 2: (59 mg, 0.155 mmol, 31%)
Average: 31% yield

Entry 40: *N,N*-Dimethylacetamide (DMA). General procedure for ligand screening was followed. **1a** (163 mg, 0.500 mmol, 1.0 equiv.), **2a** (103 uL, 0.600 mmol, 1.2 equiv.), K_2CO_3 (83 mg, 0.600 mmol, 1.2 equiv.), DMA (1.3 mg, 0.015 mmol, 0.03 equiv.), $Pd(OAc)_2$ (1.7 mg, 0.0075 mmol, 0.015 equiv.), and 95:5 anisole/DMF (1.0 mL, 0.5M) were used. The crude material was purified by flash column chromatography (silica, hexanes/ethyl acetate 19:1) to obtain products **3a** and **3a’** in 91:9 r.r. (determined by HPLC).

Run 1: (61 mg, 0.155 mmol, 32%)
Run 2: (61 mg, 0.155 mmol, 32%)
Average: 32% yield

Reaction Condition Optimization

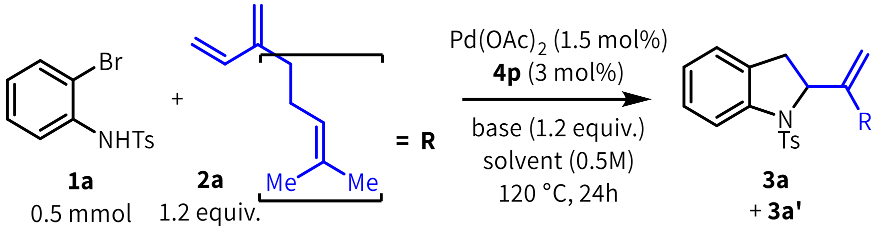

Table S1. Reaction optimization studies.

Entry	Base	Solvent	**3a** + **3a'**	r.r.
		**Solvent Screen**		
1	K_2CO_3	DMF	23%	85:15
2	K_2CO_3	50:50 toluene/DMF	35%	86:14
3	K_2CO_3	95:5 toluene/DMF	29%	91:9
4	K_2CO_3	anisole	43%[a]	88:12
5	K_2CO_3	50:50 anisole/DMF	38%	87:13
6	K_2CO_3	95:5 anisole/DMF	64%	90:10
		**Base Screen**		
7	Li_2CO_3	95:5 anisole/DMF	<5%	N/A
8	Na_2CO_3	95:5 anisole/DMF	28%	75:25
9	Cs_2CO_3	95:5 anisole/DMF	22%	84:16
10	KO^{t}Bu	95:5 anisole/DMF	57%	90:10
11	KOAc	95:5 anisole/DMF	49%	72:28
		**Reaction Tolerance** (K_2CO_3, 95:5 anisole/DMF)		
12	**4p** (1.5 mol%)		53%	90:10
13	under air		38%	87:13
14	H_2O (1.0 equiv.) added		58%	90:10
15	H_2O (5.0 equiv.) added		54%	90:10
		**Additional Experiments** (K_2CO_3, 95:5 anisole/DMF)		
16	80 °C, 48 h, $Pd(OAc)_2$ (3 mol%)		43%	89:11

[a] Solubility issues

General procedure for reaction condition optimization studies: Bromoaniline **1a** (163 mg, 0.500 mmol, 1.0 equiv.), myrcene **2a** (103 uL, 0.600 mmol, 1.2 equiv.), urea **4p** (2.7 mg, 0.015

mmol, 0.03 equiv.), palladium acetate (1.7 mg, 0.0075 mmol, 0.015 equiv.), and 1,3,5-trimethoxybenze (internal standard, 40 mg, 0.238 mmol, 0.48 equiv.) were weighed out in the above-mentioned order into a 1-dram vial equipped with a stir bar and a cap with a silicone septum. The vial was then placed under nitrogen atmosphere and charged with 1 mL of freshly degassed solvent mixture (0.5M). An aliquot for HPLC analysis was taken. Then, base (0.600 mmol, 1.2 equiv.) was added to the reaction mixture. The reaction mixture was degassed with nitrogen and the reaction was stirred at 120 °C for 24 hours. After cooling to room temperature, the yield of **3a** + **3a'** and the regioisomeric ratio were determined by HPLC analysis of the crude reaction mixture.

Solvent Screen

Entry 1: General procedure for condition optimization was followed. **1a** (163 mg, 0.500 mmol, 1.0 equiv.), **2a** (103 uL, 0.600 mmol, 1.2 equiv.), K_2CO_3 (83 mg, 0.600 mmol, 1.2 equiv.), urea **4p** (2.7 mg, 0.015 mmol, 0.03 equiv.), $Pd(OAc)_2$ (1.7 mg, 0.0075 mmol, 0.015 equiv.), 1,3,5-trimethoxybenze (40 mg, 0.238 mmol, 0.48 equiv.) and DMF (1.0 mL, 0.5M) were used. The yield of **3a** + **3a'** and the regioisomeric ratio were determined by HPLC analysis of the crude reaction mixture.

<u>Run 1</u>: 23%
<u>Run 2</u>: 23%
Average: 23% yield (85:15 r.r.)

Entry 2: General procedure for condition optimization was followed. **1a** (163 mg, 0.500 mmol, 1.0 equiv.), **2a** (103 uL, 0.600 mmol, 1.2 equiv.), K_2CO_3 (83 mg, 0.600 mmol, 1.2 equiv.), urea **4p** (2.7 mg, 0.015 mmol, 0.03 equiv.), $Pd(OAc)_2$ (1.7 mg, 0.0075 mmol, 0.015 equiv.), 1,3,5-trimethoxybenze (40 mg, 0.238 mmol, 0.48 equiv.) and 50:50 toluene/DMF (1.0 mL, 0.5M) were used. The yield of **3a** + **3a'** and the regioisomeric ratio were determined by HPLC analysis of the crude reaction mixture.

<u>Run 1</u>: 33%
<u>Run 2</u>: 36%
Average: 35% yield (86:14 r.r.)

Entry 3: General procedure for condition optimization was followed. **1a** (163 mg, 0.500 mmol, 1.0 equiv.), **2a** (103 uL, 0.600 mmol, 1.2 equiv.), K_2CO_3 (83 mg, 0.600 mmol, 1.2 equiv.), urea **4p** (2.7 mg, 0.015 mmol, 0.03 equiv.), $Pd(OAc)_2$ (1.7 mg, 0.0075 mmol, 0.015 equiv.), 1,3,5-trimethoxybenze (40 mg, 0.238 mmol, 0.48 equiv.) and 95:5 toluene/DMF (1.0 mL, 0.5M) were used. The yield of **3a** + **3a'** and the regioisomeric ratio were determined by HPLC analysis of the crude reaction mixture.

<u>Run 1</u>: 29%
<u>Run 2</u>: 28%
Average: 29% yield (91:9 r.r.)

Entry 4: General procedure for condition optimization was followed. **1a** (163 mg, 0.500 mmol, 1.0 equiv.), **2a** (103 uL, 0.600 mmol, 1.2 equiv.), K_2CO_3 (83 mg, 0.600 mmol, 1.2 equiv.), urea **4p** (2.7 mg, 0.015 mmol, 0.03 equiv.), $Pd(OAc)_2$ (1.7 mg, 0.0075 mmol, 0.015 equiv.), 1,3,5-trimethoxybenze (40 mg, 0.238 mmol, 0.48 equiv.) and anisole (1.0 mL, 0.5M) were used. The yield of **3a** + **3a'** and the regioisomeric ratio were determined by HPLC analysis of the crude reaction mixture.

<u>Run 1</u>: 45%

Run 2: 41%
Average: 43% yield (88:12 r.r.)

Entry 5: General procedure for condition optimization was followed. **1a** (163 mg, 0.500 mmol, 1.0 equiv.), **2a** (103 uL, 0.600 mmol, 1.2 equiv.), K_2CO_3 (83 mg, 0.600 mmol, 1.2 equiv.), urea **4p** (2.7 mg, 0.015 mmol, 0.03 equiv.), $Pd(OAc)_2$ (1.7 mg, 0.0075 mmol, 0.015 equiv.), 1,3,5-trimethoxybenze (40 mg, 0.238 mmol, 0.48 equiv.) and 50:50 anisole/DMF (1.0 mL, 0.5M) were used. The yield of **3a** + **3a'** and the regioisomeric ratio were determined by HPLC analysis of the crude reaction mixture.

Run 1: 40%
Run 2: 35%
Average: 38% yield (87:13 r.r.)

Entry 6: General procedure for condition optimization was followed. **1a** (163 mg, 0.500 mmol, 1.0 equiv.), **2a** (103 uL, 0.600 mmol, 1.2 equiv.), K_2CO_3 (83 mg, 0.600 mmol, 1.2 equiv.), urea **4p** (2.7 mg, 0.015 mmol, 0.03 equiv.), $Pd(OAc)_2$ (1.7 mg, 0.0075 mmol, 0.015 equiv.), 1,3,5-trimethoxybenze (40 mg, 0.238 mmol, 0.48 equiv.) and 95:5 anisole/DMF (1.0 mL, 0.5M) were used. The yield of **3a** + **3a'** and the regioisomeric ratio were determined by HPLC analysis of the crude reaction mixture.

Run 1: 61%
Run 2: 66%
Average: 64% yield (90:10 r.r.)

Base Screen

Entry 7: General procedure for condition optimization was followed. **1a** (163 mg, 0.500 mmol, 1.0 equiv.), **2a** (103 uL, 0.600 mmol, 1.2 equiv.), Li_2CO_3 (44 mg, 0.600 mmol, 1.2 equiv.), urea **4p** (2.7 mg, 0.015 mmol, 0.03 equiv.), $Pd(OAc)_2$ (1.7 mg, 0.0075 mmol, 0.015 equiv.), 1,3,5-trimethoxybenze (40 mg, 0.238 mmol, 0.48 equiv.) and 95:5 anisole/DMF (1.0 mL, 0.5M) were used. The yield of **3a** + **3a'** and the regioisomeric ratio were determined by HPLC analysis of the crude reaction mixture.

Run 1: <5%
Run 2: <5%
Average: <5% yield

Entry 8: General procedure for condition optimization was followed. **1a** (163 mg, 0.500 mmol, 1.0 equiv.), **2a** (103 uL, 0.600 mmol, 1.2 equiv.), Na_2CO_3 (64 mg, 0.600 mmol, 1.2 equiv.), urea **4p** (2.7 mg, 0.015 mmol, 0.03 equiv.), $Pd(OAc)_2$ (1.7 mg, 0.0075 mmol, 0.015 equiv.), 1,3,5-trimethoxybenze (40 mg, 0.238 mmol, 0.48 equiv.) and 95:5 anisole/DMF (1.0 mL, 0.5M) were used. The yield of **3a** + **3a'** and the regioisomeric ratio were determined by HPLC analysis of the crude reaction mixture.

Run 1: 29%
Run 2: 27%
Average: 28% yield (75:25 r.r.)

Entry 9: General procedure for condition optimization was followed. **1a** (163 mg, 0.500 mmol, 1.0 equiv.), **2a** (103 uL, 0.600 mmol, 1.2 equiv.), Cs_2CO_3 (195 mg, 0.600 mmol, 1.2 equiv.), urea **4p** (2.7 mg, 0.015 mmol, 0.03 equiv.), $Pd(OAc)_2$ (1.7 mg, 0.0075 mmol, 0.015 equiv.), 1,3,5-trimethoxybenze (40 mg, 0.238 mmol, 0.48 equiv.) and 95:5 anisole/DMF (1.0 mL, 0.5M) were

used. The yield of **3a** + **3a’** and the regioisomeric ratio were determined by HPLC analysis of the crude reaction mixture.

Run 1: 24%
Run 2: 19%
Average: 22% yield (84:16 r.r.)

Entry 10: General procedure for condition optimization was followed. **1a** (163 mg, 0.500 mmol, 1.0 equiv.), **2a** (103 uL, 0.600 mmol, 1.2 equiv.), KO^{t}Bu (73 mg, 0.600 mmol, 1.2 equiv.), urea **4p** (2.7 mg, 0.015 mmol, 0.03 equiv.), $Pd(OAc)_2$ (1.7 mg, 0.0075 mmol, 0.015 equiv.), 1,3,5-trimethoxybenze (40 mg, 0.238 mmol, 0.48 equiv.) and 95:5 anisole/DMF (1.0 mL, 0.5M) were used. The yield of **3a** + **3a’** and the regioisomeric ratio were determined by HPLC analysis of the crude reaction mixture.

Run 1: 56%
Run 2: 58%
Average: 57% yield (90:10 r.r.)

Entry 11: General procedure for condition optimization was followed. **1a** (163 mg, 0.500 mmol, 1.0 equiv.), **2a** (103 uL, 0.600 mmol, 1.2 equiv.), KOAc (59 mg, 0.600 mmol, 1.2 equiv.), urea **4p** (2.7 mg, 0.015 mmol, 0.03 equiv.), $Pd(OAc)_2$ (1.7 mg, 0.0075 mmol, 0.015 equiv.), 1,3,5-trimethoxybenze (40 mg, 0.238 mmol, 0.48 equiv.) and 95:5 anisole/DMF (1.0 mL, 0.5M) were used. The yield of **3a** + **3a’** and the regioisomeric ratio were determined by HPLC analysis of the crude reaction mixture.

Run 1: 46%
Run 2: 51%
Average: 49% yield (72:28 r.r.)

Reaction Tolerance

Entry 12: General procedure for condition optimization was followed. **1a** (163 mg, 0.500 mmol, 1.0 equiv.), **2a** (103 uL, 0.600 mmol, 1.2 equiv.), K_2CO_3 (83 mg, 0.600 mmol, 1.2 equiv.), urea **4p** (1.4 mg, 0.0075 mmol, 0.015 equiv.), $Pd(OAc)_2$ (1.7 mg, 0.0075 mmol, 0.015 equiv.), 1,3,5-trimethoxybenze (40 mg, 0.238 mmol, 0.48 equiv.) and 95:5 anisole/DMF (1.0 mL, 0.5M) were used. The yield of **3a** + **3a’** and the regioisomeric ratio were determined by HPLC analysis of the crude reaction mixture.

Run 1: 53%
Run 2: 52%
Average: 53% yield (90:10 r.r.)

Entry 13: General procedure for condition optimization was followed but reaction was run in presence of air. **1a** (163 mg, 0.500 mmol, 1.0 equiv.), **2a** (103 uL, 0.600 mmol, 1.2 equiv.), K_2CO_3 (83 mg, 0.600 mmol, 1.2 equiv.), urea **4p** (2.7 mg, 0.015 mmol, 0.03 equiv.), $Pd(OAc)_2$ (1.7 mg, 0.0075 mmol, 0.015 equiv.), 1,3,5-trimethoxybenze (40 mg, 0.238 mmol, 0.48 equiv.) and non-degassed 95:5 anisole/DMF (1.0 mL, 0.5M) were used. The yield of **3a** + **3a’** and the regioisomeric ratio were determined by HPLC analysis of the crude reaction mixture.

Run 1: 40%
Run 2: 35%
Average: 38% yield (87:13 r.r.)

Entry 14: General procedure for condition optimization was followed. **1a** (163 mg, 0.500 mmol, 1.0 equiv.), **2a** (103 uL, 0.600 mmol, 1.2 equiv.), K_2CO_3 (83 mg, 0.600 mmol, 1.2 equiv.), urea **4p** (2.7 mg, 0.015 mmol, 0.03 equiv.), $Pd(OAc)_2$ (1.7 mg, 0.0075 mmol, 0.015 equiv.), 1,3,5-trimethoxybenze (40 mg, 0.238 mmol, 0.48 equiv.) and 95:5 anisole/DMF (1.0 mL, 0.5M) were used. H_2O (9.0 uL, 0.500 mmol, 1.0 equiv.) was added to the reaction mixture before heating it up to 120 °C. The yield of **3a** + **3a'** and the regioisomeric ratio were determined by HPLC analysis of the crude reaction mixture.

Run 1: 57%
Run 2: 59%
Average: 58% yield (90:10 r.r.)

Entry 15: General procedure for condition optimization was followed. **1a** (163 mg, 0.500 mmol, 1.0 equiv.), **2a** (103 uL, 0.600 mmol, 1.2 equiv.), K_2CO_3 (83 mg, 0.600 mmol, 1.2 equiv.), urea **4p** (2.7 mg, 0.015 mmol, 0.03 equiv.), $Pd(OAc)_2$ (1.7 mg, 0.0075 mmol, 0.015 equiv.), 1,3,5-trimethoxybenze (40 mg, 0.238 mmol, 0.48 equiv.) and 95:5 anisole/DMF (1.0 mL, 0.5M) were used. H_2O (45 uL, 2.500 mmol, 5.0 equiv.) was added to the reaction mixture before heating it up to 120 °C. The yield of **3a** + **3a'** and the regioisomeric ratio were determined by HPLC analysis of the crude reaction mixture.

Run 1: 56%
Run 2: 52%
Average: 54% yield (90:10 r.r.)

Additional Experiments

Entry 16: Low temperature run to rule out formation of Pd nanoparticles. General procedure for condition optimization was followed. **1a** (163 mg, 0.500 mmol, 1.0 equiv.), **2a** (103 uL, 0.600 mmol, 1.2 equiv.), K_2CO_3 (83 mg, 0.600 mmol, 1.2 equiv.), urea **4p** (5.4 mg, 0.030 mmol, 0.06 equiv.), $Pd(OAc)_2$ (3.4 mg, 0.015 mmol, 0.03 equiv.), 1,3,5-trimethoxybenze (40 mg, 0.238 mmol, 0.48 equiv.) and 95:5 anisole/DMF (1.0 mL, 0.5M) were used. The reaction was stirred at 80 °C for 48 hours The yield of **3a** + **3a'** and the regioisomeric ratio were determined by HPLC analysis of the crude reaction mixture.

Run 1: 41%
Run 2: 44%
Average: 43% yield (89:11 r.r.)

Reaction Time Course Experiment

General procedure for kinetic comparison studies: Bromoaniline **1a** (163 mg, 0.500 mmol, 1.0 equiv.), myrcene **2a** (103 uL, 0.600 mmol, 1.2 equiv.), urea **4p** (2.7 mg, 0.015 mmol, 0.03 equiv.), palladium acetate (1.7 mg, 0.0075 mmol, 0.015 equiv.), and 1,3,5-trimethoxybenze (internal standard, 40 mg, 0.238 mmol, 0.48 equiv.) were weighed out in the above-mentioned order into a 1-dram vial equipped with a stir bar and a cap with a silicone septum. The vial was then placed under nitrogen atmosphere and charged with 1 mL of freshly degassed solvent mixture (0.5M). An aliquot for HPLC analysis was taken. Then, base (0.600 mmol, 1.2 equiv.) was added to the reaction mixture. The reaction mixture was degassed with nitrogen and the reaction was stirred at 120 °C additional aliquot for HPLC analysis were taken at 4, 8 and 24 h. After cooling to room temperature, the yield of **3a** + **3a’** and the regioisomeric ratio were determined by HPLC analysis of the crude reaction mixture. Control reactions were run using above procedure omitting the addition of urea **4p**.

Table S2. Kinetic comparison Studies.

Run	Time (h)	**3a** + **3a'**	r.r.
		**Ligand**	
1	4	35%	88:12
1	8	43%	88:12
1	24	67%	89:11
2	4	34%	89:11
2	8	43%	89:11
2	24	63%	89:11
		**No Ligand**	
3	4	18%	89:11
3	8	20%	89:11
3	24	26%	89:11
4	4	19%	90:10
4	8	23%	89:11
4	24	27%	89:11

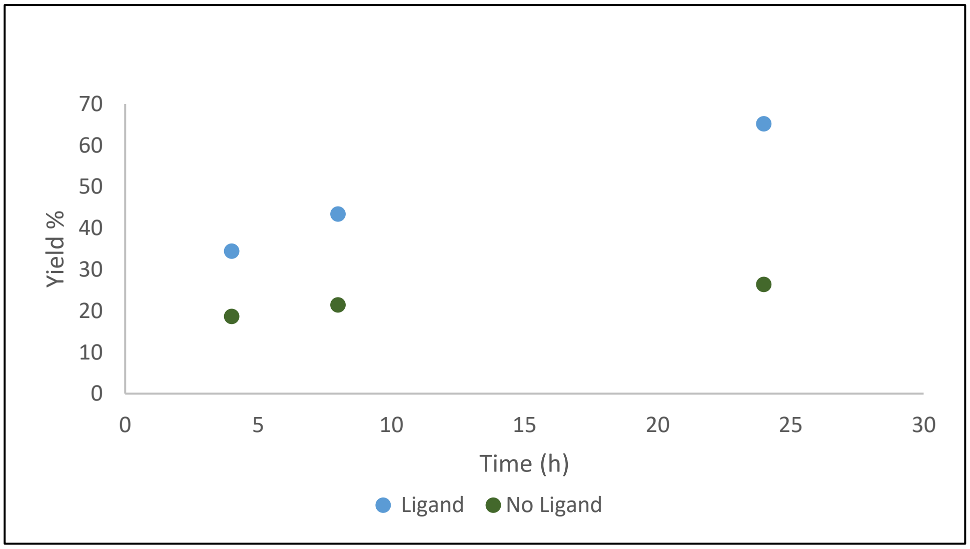

Reaction Scope

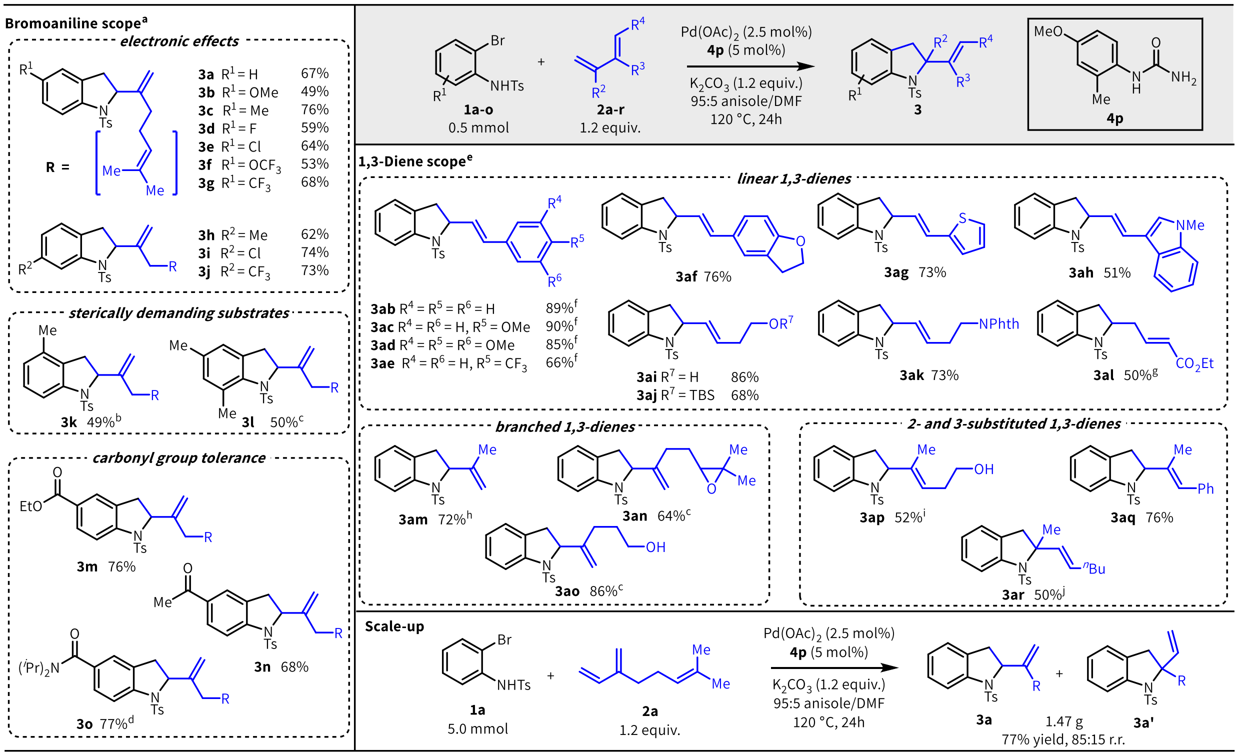

Legend: a) Product ratios of **3/3'** generally 88:12-93:7.[19] b) 82:18 r.r. c) >99:1 r.r. d) 5 mol% Pd(OAc)$_2$ and 10 mol% **4p** used. e) Diene scope was run with 1.3 equiv. of diene. f) 1.5 mol% Pd(OAc)$_2$ and 3 mol% **4p** used. g) **2l** added in 2 portions. h) 97:3 r.r. i) 1.8:1 *E*/*Z*. j) Added 1.0 equiv. *n*-Bu$_4$NCl.

General heteroannulation procedure: *N*-Tosylbromoaniline **1** (0.500 mmol, 1.0 equiv.), diene **2** (0.600 or 0.650 mmol, 1.2 or 1.3 equiv.), urea **4p** (2.7 or 4.5 mg, 0.015 or 0.025 mmol, 0.03 or 0.05 equiv.), potassium carbonate (83 mg, 0.600 mmol, 1.2 equiv.), and palladium acetate (0.015, 0.025 or 0.050 equiv.) were weighed out in the above-mentioned order into a 1-dram vial equipped with a stir bar and a cap with a silicone septum. The vial was then placed under nitrogen atmosphere and charged with 1 mL of a freshly degassed anisole/dimethylformamide (95:5) solvent mixture. The reaction was stirred at 120 °C for 24 h. After cooling to room temperature, the reaction mixture was filtered with ethyl acetate through a cotton plug. The solvents were removed under reduced pressure and the crude mixture was purified by flash column chromatography on silica to obtain products **3**.

Bromoaniline Scope

2-(6-methylhepta-1,5-dien-2-yl)-*N*-tosylindoline (3a)

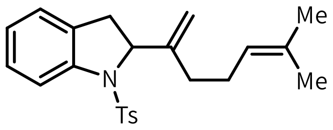

Prepared according to the general procedure. Bromoaniline **1a** (163 mg, 0.500 mmol, 1.0 equiv.), myrcene **2a** (103 uL, 0.600 mmol, 1.2 equiv.), K_2CO_3 (83 mg, 0.600 mmol, 1.2 equiv.), urea **4p** (4.5 mg, 0.025 mmol, 0.05 equiv.), $Pd(OAc)_2$ (2.8 mg, 0.0125 mmol, 0.025 equiv.), and 95:5 anisole/DMF (1 mL, 0.5M) were used. The crude material was purified by flash column chromatography (silica, hexanes/ethyl acetate 19:1) to obtain products **3a** and **3a'** (90:10 r.r.) as pale yellow oil.

Run 1: (126 mg, 0.330 mmol, 66%)
Run 2: (127 mg, 0.333 mmol, 66%)
Run 3: (130 mg, 0.341 mmol, 68%)
Average yield: 67%

5-Methoxy-2-(6-methylhepta-1,5-dien-2-yl)-*N*-tosylindoline (3b)

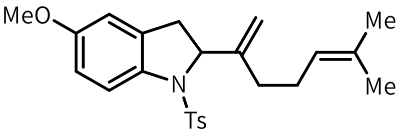

Prepared according to the general procedure. Bromoaniline **1b** (178 mg, 0.500 mmol, 1.0 equiv.), myrcene **2a** (103 uL, 0.600 mmol, 1.2 equiv.), K_2CO_3 (83 mg, 0.600 mmol, 1.2 equiv.), urea **4p** (4.5 mg, 0.025 mmol, 0.05 equiv.), $Pd(OAc)_2$ (2.8 mg, 0.0125 mmol, 0.025 equiv.), and 95:5 anisole/DMF (1 mL, 0.5M) were used. The crude material was purified by flash column chromatography (silica, hexanes/ethyl acetate 19:1) to obtain products **3b** and **3b'** (92:8 r.r.) as pale yellow oil.

Run 1: (101 mg, 0.245 mmol, 49%)
Run 2: (95 mg, 0.231 mmol, 46%)
Run 3: (105 mg, 0.255 mmol, 51%)
Average yield: 49%

^{1}H NMR (500 MHz, $CDCl_3$) δ 7.59 (d, *J* = 8.8 Hz, 1H), 7.54 – 7.50 (m, 2H), 7.17 (d, *J* = 8.3 Hz, 2H), 6.75 (dd, *J* = 8.8, 2.6 Hz, 1H), 6.57 – 6.55 (m, 1H), 5.11 (d, *J* = 3.3 Hz, 1H), 5.09 (ddd, *J* = 6.9, 4.1, 2.8 Hz, 1H), 4.87 (s, 1H), 4.63 (dd, *J* = 9.9, 2.9 Hz, 1H), 3.79 – 3.72 (m, 3H), 2.80 (dd, *J* = 16.2, 9.9 Hz, 1H), 2.59 (dd, *J* = 16.3, 3.1 Hz, 1H), 2.36 (s, 3H), 2.21 – 2.07 (m, 3H), 1.98 (dt, *J* = 15.3, 7.5 Hz, 1H), 1.67 (d, *J* = 8.1 Hz, 3H), 1.60 (s, 3H).

^{13}C NMR (125 MHz, $CDCl_3$) δ 157.5, 148.3, 143.8, 135.5, 135.0, 133.8, 132.0, 129.6, 127.3, 124.1, 118.3, 112.9, 110.8, 110.6, 66.5, 55.7, 34.9, 31.6, 26.4, 25.8, 21.7.

HRMS (ESI) *m/z* calculated for $C_{24}H_{30}NO_3S$ $[M+H]^+$: 412.1946, found 412.1933.

5-Methyl-2-(6-methylhepta-1,5-dien-2-yl)-N-tosylindoline (3c)

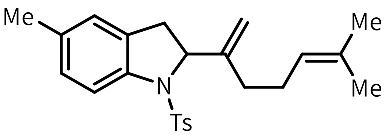

Prepared according to the general procedure. Bromoaniline **1c** (170 mg, 0.500 mmol, 1.0 equiv.), myrcene **2a** (103 uL, 0.600 mmol, 1.2 equiv.), K_2CO_3 (83 mg, 0.600 mmol, 1.2 equiv.), urea **4p** (4.5 mg, 0.025 mmol, 0.05 equiv.), $Pd(OAc)_2$ (2.8 mg, 0.0125 mmol, 0.025 equiv.), and 95:5 anisole/DMF (1 mL, 0.5M) were used. The crude material was purified by flash column chromatography (silica, hexanes/ethyl acetate 19:1) to obtain products **3c** and **3c'** (91:9 r.r.) as pale yellow oil.

Run 1: (151 mg, 0.382 mmol, 76%)
Run 2: (150 mg, 0.379 mmol, 76%)

Run 3: (153 mg, 0.387 mmol, 77%)
Average yield: 76%

^{1}H NMR (500 MHz, $CDCl_3$) δ 7.58 – 7.53 (m, 3H), 7.17 (d, *J* = 8.0 Hz, 2H), 7.00 (d, *J* = 8.2 Hz, 1H), 6.82 (s, 1H), 5.12 (s, 1H), 5.11 – 5.07 (m, 1H), 4.87 (s, 1H), 4.64 (dd, *J* = 10.0, 3.2 Hz, 1H), 2.89 (dd, *J* = 16.1, 10.1 Hz, 1H), 2.62 (dd, *J* = 16.2, 3.3 Hz, 1H), 2.36 (s, 3H), 2.26 (d, *J* = 5.1 Hz, 3H), 2.21 – 2.06 (m, 3H), 2.03 – 1.94 (m, 1H), 1.67 (t, *J* = 3.1 Hz, 3H), 1.59 (s, 3H).

^{13}C NMR (125 MHz, $CDCl_3$) δ 148.5, 143.8, 139.8, 135.2, 134.31, 132.0, 131.8, 129.6, 128.5, 127.3, 125.7, 124.1, 116.7, 110.5, 66.4, 34.8, 31.4, 26.3, 25.8, 21.7, 21.1, 17.9.

HRMS (ESI) *m/z* calculated for $C_{24}H_{30}NO_2S$ $[M+H]^+$: 396.1997, found 396.1986.

5-Fluoro-2-(6-methylhepta-1,5-dien-2-yl)-1-tosylindoline (3d)

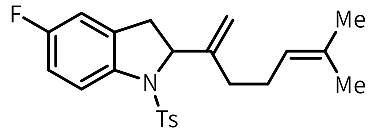

Prepared according to the general procedure. Bromoaniline **1d** (172 mg, 0.500 mmol, 1.0 equiv.), myrcene **2a** (103 uL, 0.600 mmol, 1.2 equiv.), K_2CO_3 (83 mg, 0.600 mmol, 1.2 equiv.), urea **4p** (4.5 mg, 0.025 mmol, 0.05 equiv.), $Pd(OAc)_2$ (2.8 mg, 0.0125 mmol, 0.025 equiv.), and 95:5 anisole/DMF (1 mL, 0.5M) were used. The crude material was purified by flash column chromatography (silica, hexanes/ethyl acetate 19:1) to obtain products **3d** and **3d'** (92:8 r.r.) as pale yellow oil.

Run 1: (119 mg, 0.297 mmol, 59%)
Run 2: (122 mg, 0.305 mmol, 61%)
Run 3: (112 mg, 0.280 mmol, 56%)
Average yield: 59%

^{1}H NMR (400 MHz, $CDCl_3$) δ 7.62 (dd, *J* = 8.7, 4.6 Hz, 1H), 7.54 (d, *J* = 8.3 Hz, 2H), 7.19 (d, *J* = 8.3 Hz, 2H), 6.90 (td, *J* = 8.9, 2.8 Hz, 1H), 6.72 (dd, *J* = 8.1, 2.8 Hz, 1H), 5.10 (s, 1H), 5.09 (t, *J* = 5.4 Hz, 1H), 4.89 (s, 1H), 4.68 (dd, *J* = 10.0, 3.5 Hz, 1H), 2.87 (dd, *J* = 16.6, 10.0 Hz, 1H), 2.63 (dd, *J* = 16.6, 3.5 Hz, 1H), 2.36 (s, 3H), 2.22–1.92 (m, 4H), 1.67 (s, 3H), 1.59 (s, 3H).

^{13}C NMR (100 MHz, $CDCl_3$) δ 160.3 (d, $J_{C\text{-}F}$ = 241 Hz), 148.0, 144.1, 138.2, 134.8, 134.1 (d, $J_{C\text{-}F}$ = 9 Hz), 132.1, 129.7, 127.2, 123.9, 118.1 (d, $J_{C\text{-}F}$ = 9 Hz), 114.4 (d, $J_{C\text{-}F}$ = 23 Hz), 112.2 (d, $J_{C\text{-}F}$ = 24 Hz), 110.8, 66.6, 34.7, 31.4, 26.3, 25.8, 21.6, 17.8.

HRMS (ESI) *m/z* calculated for $C_{23}H_{27}FNO_2S$ $[M+H]^+$: 400.1747, found 400.1731.

5-Chloro-2-(6-methylhepta-1,5-dien-2-yl)-*N*-tosylindoline (3e)

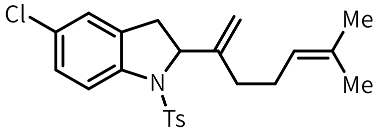

Prepared according to the general procedure. Bromoaniline **1e** (180 mg, 0.500 mmol, 1.0 equiv.), myrcene **2a** (103 uL, 0.600 mmol, 1.2 equiv.), K_2CO_3 (83 mg, 0.600 mmol, 1.2 equiv.), urea **4p** (4.5 mg, 0.025 mmol, 0.05 equiv.), $Pd(OAc)_2$ (2.8 mg, 0.0125 mmol, 0.025 equiv.), and 95:5 anisole/DMF (1 mL, 0.5M) were used. The crude material was purified by flash column chromatography (silica, hexanes/ethyl acetate 19:1) to obtain products **3e** and **3e'** (93:7 r.r.) as an off-white solid.

Run 1: (136 mg, 0.327 mmol, 65%)
Run 2: (127 mg, 0.306 mmol, 61%)
Run 3: (137 mg, 0.330 mmol, 66%)
Average yield: 64%

^1H NMR (500 MHz, $CDCl_3$) δ 7.60 (d, *J* = 8.6 Hz, 1H), 7.57 (d, *J* = 8.3 Hz, 2H), 7.23 – 7.14 (m, 3H), 6.99 (s, 1H), 5.11 (s, 1H), 5.08 (t, *J* = 5.9 Hz, 1H), 4.89 (s, 1H), 4.68 (dd, *J* = 10.1, 3.7 Hz, 1H), 2.93 (dd, *J* = 16.5, 10.1 Hz, 1H), 2.66 (dd, *J* = 16.5, 3.7 Hz, 1H), 2.37 (s, 3H), 2.23 – 2.05 (m, 3H), 2.04 – 1.90 (m, 1H), 1.67 (s, 3H), 1.59 (s, 3H).

^{13}C NMR (125 MHz, $CDCl_3$) δ 148.1, 144.2, 140.9, 134.9, 133.6, 132.1, 129.8, 129.7, 127.9, 127.2, 125.2, 123.9, 117.7, 110.9, 66.6, 34.6, 31.2, 26.2, 25.8, 21.7, 17.9.

HRMS (ESI) *m/z* calculated for $C_{23}H_{27}ClNO_2S$ $[M+H]^+$: 416.1451, found 416.1443.

2-(6-Methylhepta-1,5-dien-2-yl)-*N*-tosyl-5-(trifluoromethoxy)indoline (3f)

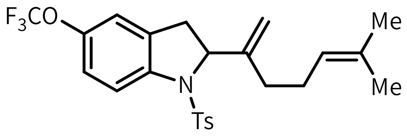

Prepared according to the general procedure. Bromoaniline **1f** (205 mg, 0.500 mmol, 1.0 equiv.), myrcene **2a** (103 uL, 0.600 mmol, 1.2 equiv.), K_2CO_3 (83 mg, 0.600 mmol, 1.2 equiv.), urea **4p** (4.5 mg, 0.025 mmol, 0.05 equiv.), $Pd(OAc)_2$ (2.8 mg, 0.0125 mmol, 0.025 equiv.), and 95:5 anisole/DMF (1 mL, 0.5M) were used. The crude material was purified by flash column chromatography (silica, hexanes/ethyl acetate 19:1) to obtain products **3f** and **3f’** (92:8 r.r.) as pale yellow oil.

Run 1: (131 mg, 0.280 mmol, 56%)
Run 2: (119 mg, 0.255 mmol, 51%)
Run 3: (122 mg, 0.262 mmol, 52%)
Average yield: 53%

^1H NMR (500 MHz, $CDCl_3$) δ 7.67 (d, *J* = 8.8 Hz, 1H), 7.58 (d, *J* = 8.3 Hz, 2H), 7.21 (d, *J* = 8.3 Hz, 2H), 7.06 (d, *J* = 8.8 Hz, 1H), 6.88 (s, 1H), 5.12 (s, 1H), 5.09 (t, *J* = 5.6 Hz, 1H), 4.91 (s, 1H), 4.71 (dd, *J* = 10.2, 3.7 Hz, 1H), 2.98 (dd, *J* = 16.6, 10.2 Hz, 1H), 2.70 (dd, *J* = 16.6, 3.7 Hz, 1H), 2.38 (s, 3H), 2.23 – 1.92 (m, 4H), 1.68 (s, 3H), 1.59 (s, 3H).

^{13}C NMR (125 MHz, $CDCl_3$) δ 148.0, 146.0, 144.3, 140.9, 135.0, 133.5, 132.2, 129.8, 127.3, 123.9, 120.7, 120.6 (q, $J_{C\text{-}F}$ = 255.4 Hz), 118.2, 117.3, 111.1, 66.8, 34.8, 31.3, 26.3, 25.8, 21.7, 17.9.

HRMS (ESI) *m/z* calculated for $C_{24}H_{27}F_3NO_3S$ $[M+H]^+$: 466.1664, found 466.1655.

2-(6-Methylhepta-1,5-dien-2-yl)-*N*-tosyl-5-(trifluoromethyl)indoline (3g)

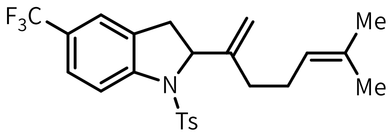

Prepared according to the general procedure. Bromoaniline **1g** (197 mg, 0.500 mmol, 1.0 equiv.), myrcene **2a** (103 uL, 0.600 mmol, 1.2 equiv.), K_2CO_3 (83 mg, 0.600 mmol, 1.2 equiv.), urea **4p** (4.5 mg, 0.025 mmol, 0.05 equiv.), $Pd(OAc)_2$ (2.8 mg, 0.0125 mmol, 0.025 equiv.), and 95:5 anisole/DMF (1 mL, 0.5M) were used. The crude material was purified by flash column chromatography (silica, hexanes/ethyl acetate 19:1) to obtain products **3g** and **3g’** (91:9 r.r.) as pale yellow oil.

Run 1: (156 mg, 0.346 mmol, 69%)
Run 2: (146 mg, 0.325 mmol, 65%)
Run 3: (158 mg, 0.351 mmol, 70%)
Average yield: 68%

^1H NMR (500 MHz, $CDCl_3$) δ 7.74 (d, *J* = 8.4 Hz, 1H), 7.62 (d, *J* = 8.3 Hz, 2H), 7.47 (d, *J* = 8.4 Hz, 1H), 7.27 (s, 1H), 7.23 (d, *J* = 8.3 Hz, 2H), 5.12 (s, 1H), 5.10 – 5.04 (m, 1H), 4.91 (s, 1H), 4.76 (dd, *J* = 10.4, 3.8 Hz, 1H), 3.10 (dd, *J* = 16.5, 10.4 Hz, 1H), 2.77 (dd, *J* = 16.5, 3.8 Hz, 1H),

2.38 (d, J = 4.2 Hz, 3H), 2.21 – 2.12 (m, 2H), 2.09 – 1.92 (m, 2H), 1.67 (t, J = 3.0 Hz, 3H), 1.59 (s, 3H).

^{13}C NMR (125 MHz, $CDCl_3$) δ 148.1, 145.3, 144.5, 135.2, 132.3, 131.9, 129.9, 127.3, 126.4 (q, J_{C-F} = 32.4 Hz), 125.6 (q, J_{C-F} = 3.9 Hz), 124.3 (q, J_{C-F} = 269.9 Hz), 123.8, 122.3 (q, J_{C-F} = 3.8 Hz), 115.7, 111.2, 66.9, 34.8, 31.1, 26.2, 25.8, 21.7, 17.9.

HRMS (ESI) *m/z* calculated for $C_{24}H_{27}F_3NO_2S$ $[M+H]^+$: 450.1715, found 450.1705.

6-methyl-2-(6-methylhepta-1,5-dien-2-yl)-*N*-tosylindoline (3h)

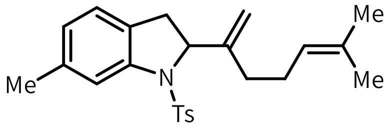

Prepared according to the general procedure. Bromoaniline **1h** (170 mg, 0.500 mmol, 1.0 equiv.), myrcene **2a** (103 uL, 0.600 mmol, 1.2 equiv.), K_2CO_3 (83 mg, 0.600 mmol, 1.2 equiv.), urea **4p** (4.5 mg, 0.025 mmol, 0.05 equiv.), $Pd(OAc)_2$ (2.8 mg, 0.0125 mmol, 0.025 equiv.), and 95:5 anisole/DMF (1 mL, 0.5M) were used. The crude material was purified by flash column chromatography (silica, hexanes/ethyl acetate 19:1) to obtain products **3h** and **3h'** (93:7 r.r.) as pale yellow oil.

Run 1: (117 mg, 0.295 mmol, 59%)
Run 2: (123 mg, 0.311 mmol, 62%)
Run 3: (127 mg, 0.321 mmol, 64%)
Average yield: 62%

^{1}H NMR (500 MHz, $CDCl_3$) δ 7.61 – 7.55 (m, 2H), 7.52 (s, 1H), 7.18 (d, J = 8.0 Hz, 2H), 6.89 (d, J = 7.6 Hz, 1H), 6.81 (d, J = 7.5 Hz, 1H), 5.11 (s, 1H), 5.11 – 5.06 (m, 1H), 4.87 (s, 1H), 4.66 (dd, J = 10.2, 3.2 Hz, 1H), 2.90 (dd, J = 15.9, 10.2 Hz, 1H), 2.62 (dd, J = 16.0, 3.2 Hz, 1H), 2.37 (s, 4H), 2.36 (s, 3H), 2.21 – 2.05 (m, 3H), 2.02 – 1.93 (m, 1H), 1.67 (d, J = 6.7 Hz, 3H), 1.59 (s, 3H).

^{13}C NMR (125 MHz, $CDCl_3$) δ 148.5, 143.8, 142.3, 137.8, 135.3, 131.9, 129.6, 128.7, 127.2, 125.4, 124.6, 124.1, 117.4, 110.5, 66.6, 34.5, 31.3, 26.3, 25.8, 21.7, 21.6, 17.8.

HRMS (ESI) *m/z* calculated for $C_{24}H_{30}NO_2S$ $[M+H]^+$: 396.1997, found 396.1985.

6-Chloro-2-(6-methylhepta-1,5-dien-2-yl)-*N*-tosylindoline (3i)

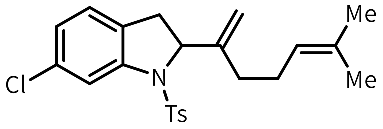

Prepared according to the general procedure. Bromoaniline **1i** (180 mg, 0.500 mmol, 1.0 equiv.), myrcene **2a** (103 uL, 0.600 mmol, 1.2 equiv.), K_2CO_3 (83 mg, 0.600 mmol, 1.2 equiv.), urea **4p** (4.5 mg, 0.025 mmol, 0.05 equiv.), $Pd(OAc)_2$ (2.8 mg, 0.0125 mmol, 0.025 equiv.), and 95:5 anisole/DMF (1 mL, 0.5M) were used. The crude material was purified by flash column chromatography (silica, hexanes/ethyl acetate 19:1) to obtain products **3i** and **3i'** (91:9 r.r.) as an off-white solid.

Run 1: (157 mg, 0.376 mmol, 75%)
Run 2: (149 mg, 0.359 mmol, 72%)
Run 3: (154 mg, 0.371 mmol, 74%)
Average yield: 74%

^{1}H NMR (500 MHz, $CDCl_3$) δ 7.69 (d, J = 1.8 Hz, 1H), 7.63 – 7.58 (m, 2H), 7.24 – 7.19 (m, 2H), 6.98 – 6.95 (m, 1H), 6.93 (d, J = 8.0 Hz, 1H), 5.12 – 5.10 (m, 1H), 5.08 (dddd, J = 7.0, 5.5, 2.8, 1.4 Hz, 1H), 4.89 (s, 1H), 4.70 (dd, J = 10.2, 3.5 Hz, 1H), 2.96 (dd, J = 16.3, 10.3 Hz, 1H), 2.65 (dd, J = 16.3, 3.6 Hz, 1H), 2.38 (s, 3H), 2.16 (q, J = 7.6 Hz, 2H), 2.06 (dt, J = 8.0, 6.7 Hz, 1H), 1.95 (dt, J = 15.7, 7.7 Hz, 1H), 1.67 (s, 3H), 1.59 (s, 3H).

^{13}C NMR (125 MHz, $CDCl_3$) δ 148.1, 144.3, 143.4, 135.0, 133.5, 132.1, 130.0, 129.8, 127.2, 125.8, 124.5, 123.9, 116.7, 110.9, 67.0, 34.5, 31.1, 26.2, 25.8, 21.7, 17.8.

HRMS (ESI) *m/z* calculated for $C_{23}H_{27}ClNO_2S$ $[M+H]^+$: 416.1451, found 416.1442.

2-(6-Methylhepta-1,5-dien-2-yl)-*N*-tosyl-6-(trifluoromethyl)indoline (3j)

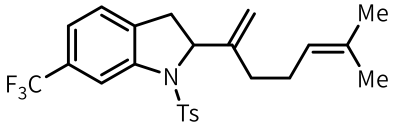

Prepared according to the general procedure. Bromoaniline **1j** (197 mg, 0.500 mmol, 1.0 equiv.), myrcene **2a** (103 uL, 0.600 mmol, 1.2 equiv.), K_2CO_3 (83 mg, 0.600 mmol, 1.2 equiv.), urea **4p** (4.5 mg, 0.025 mmol, 0.05 equiv.), $Pd(OAc)_2$ (2.8 mg, 0.0125 mmol, 0.025 equiv.), and 95:5 anisole/DMF (1 mL, 0.5M) were used. The crude material was purified by flash column chromatography (silica, hexanes/ethyl acetate 19:1) to obtain products **3j** and **3j'** (90:10 r.r.) as pale yellow oil.

Run 1: (168 mg, 0.371 mmol, 74%)
Run 2: (164 mg, 0.365 mmol, 73%)
Run 3: (159 mg, 0.354 mmol, 71%)
Average yield: 73%

^1H NMR (500 MHz, $CDCl_3$) δ 7.92 (s, 1H), 7.60 (d, *J* = 8.3 Hz, 2H), 7.26 (d, *J* = 7.8 Hz, 1H), 7.21 (d, *J* = 8.3 Hz, 2H), 7.12 (d, *J* = 7.8 Hz, 1H), 5.12 (s, 1H), 5.08 (ddd, *J* = 6.9, 4.1, 1.3 Hz, 1H), 4.91 (s, 1H), 4.75 (dd, *J* = 10.3, 3.7 Hz, 1H), 3.06 (dd, *J* = 16.7, 10.4 Hz, 1H), 2.76 (dd, *J* = 16.8, 3.5 Hz, 1H), 2.37 (s, 3H), 2.17 (q, *J* = 7.4 Hz, 2H), 2.12 – 2.03 (m, 1H), 1.97 (dt, *J* = 15.7, 7.7 Hz, 1H), 1.68 (s, 3H), 1.59 (s, 3H).

^{13}C NMR (125 MHz, $CDCl_3$) δ 148.0, 144.4, 142.9, 135.5, 135.0, 132.2, 130.6 (q, J_{C-F} = 33.6 Hz), 129.9, 127.3, 125.3, 124.2 (q, J_{C-F} = 270.8 Hz), 123.8, 121.4 (q, J_{C-F} = 3.9 Hz), 113.2 (q, J_{C-F} = 4.1 Hz), 111.1, 66.7, 34.9, 31.2, 26.3, 25.8, 21.7, 17.8.

HRMS (ESI) *m/z* calculated for $C_{24}H_{27}F_3NO_2S$ $[M+H]^+$: 450.1715, found 450.1706.

4-Methyl-2-(6-methylhepta-1,5-dien-2-yl)-*N*-tosylindoline (3k)

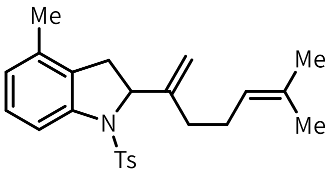

Prepared according to the general procedure. Bromoaniline **1k** (170 mg, 0.500 mmol, 1.0 equiv.), myrcene **2a** (103 uL, 0.600 mmol, 1.2 equiv.), K_2CO_3 (83 mg, 0.600 mmol, 1.2 equiv.), urea **4p** (4.5 mg, 0.025 mmol, 0.05 equiv.), $Pd(OAc)_2$ (2.8 mg, 0.0125 mmol, 0.025 equiv.), and 95:5 anisole/DMF (1 mL, 0.5M) were used. The crude material was purified by flash column chromatography (silica, hexanes/ethyl acetate 19:1) to obtain products **3k** and **3k'** (82:18 r.r.) as pale yellow oil.

Run 1: (97 mg, 0.245 mmol, 49%)
Run 2: (92 mg, 0.232 mmol, 46%)
Run 3: (101 mg, 0.255 mmol, 51%)
Average yield: 49%

^1H NMR (500 MHz, $CDCl_3$) δ 7.60 (d, *J* = 8.0 Hz, 2H), 7.51 (d, *J* = 8.0 Hz, 1H), 7.18 (d, *J* = 8.4 Hz, 2H), 7.11 (t, *J* = 7.8 Hz, 1H), 6.81 (d, *J* = 7.5 Hz, 1H), 5.14 (d, *J* = 0.7 Hz, 1H), 5.13 – 5.07 (m, 1H), 4.89 (s, 1H), 4.69 (dd, *J* = 10.3, 3.8 Hz, 1H), 2.91 (dd, *J* = 16.1, 10.4 Hz, 1H), 2.61 (dd, *J* = 16.2, 3.8 Hz, 1H), 2.36 (s, 3H), 2.23 – 2.11 (m, 3H), 2.09 (s, 3H), 2.03 – 1.95 (m, 1H), 1.68 (s, 3H), 1.60 (s, 3H).

^{13}C NMR (125 MHz, $CDCl_3$) δ 148.9, 143.8, 141.9, 135.3, 134.4, 132.0, 130.1, 129.6, 127.9, 127.3, 125.4, 124.1, 113.8, 110.4, 66.3, 34.0, 31.3, 26.3, 25.8, 21.7, 18.8, 17.9.

HRMS (ESI) *m/z* calculated for $C_{24}H_{30}NO_2S$ $[M+H]^+$: 396.1997, found 396.1984.

5,7-Dimethyl-2-(6-methylhepta-1,5-dien-2-yl)-1-tosylindoline (3l)

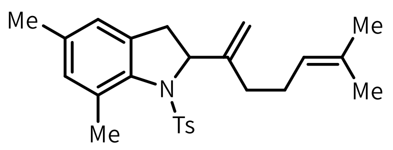

Prepared according to the general procedure. Bromoaniline **1l** (177 mg, 0.500 mmol, 1.0 equiv.), myrcene **2a** (103 uL, 0.600 mmol, 1.2 equiv.), K_2CO_3 (83 mg, 0.600 mmol, 1.2 equiv.), urea **4p** (4.5 mg, 0.025 mmol, 0.05 equiv.), $Pd(OAc)_2$ (2.8 mg, 0.0125 mmol, 0.025 equiv.), and 95:5 anisole/DMF (1 mL, 0.5M) were used. The crude material was purified by flash column chromatography (silica, hexanes/ethyl acetate 19:1) to obtain products **3l** and **3l'** (>99:1 r.r.) as pale yellow oil.

Run 1: (106 mg, 0.259 mmol, 52%)
Run 2: (102 mg, 0.249 mmol, 50%)
Run 3: (96 mg, 0.234 mmol, 47%)
Average yield: 50%

^{1}H NMR (500 MHz, $CDCl_3$) δ 7.40 (d, *J* = 8.2 Hz, 2H), 7.16 (d, *J* = 7.9 Hz, 2H), 6.90 (s, 1H), 6.64 (s, 1H), 5.07 (dd, *J* = 7.6, 6.2 Hz, 1H), 4.94 (s, 1H), 4.78 (s, 1H), 4.66 (d, *J* = 8.2 Hz, 1H), 2.54 (s, 3H), 2.52 (d, *J* = 5.7 Hz, 1H), 2.39 (s, 3H), 2.32 (d, *J* = 4.5 Hz, 1H), 2.26 (s, 3H), 2.13 – 1.93 (m, 4H), 1.68 (s, 3H), 1.59 (s, 3H).

^{13}C NMR (125 MHz, $CDCl_3$) δ 146.8, 143.8, 138.7, 136.6, 136.4, 135.0, 132.1, 132.0, 131.0, 129.5, 127.8, 124.0, 122.8, 110.6, 66.9, 33.4, 32.7, 26.7, 25.8, 21.7, 21.2, 19.9, 17.9.

HRMS (ESI) *m/z* calculated for $C_{25}H_{32}NO_2S$ $[M+H]^+$: 410.2154, found 410.2141.

Ethyl 2-(6-methylhepta-1,5-dien-2-yl)-*N*-tosylindoline-5-carboxylate (3m)

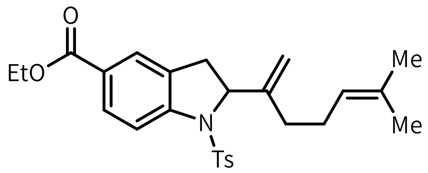

Prepared according to the general procedure. Bromoaniline **1m** (199 mg, 0.500 mmol, 1.0 equiv.), myrcene **2a** (103 uL, 0.600 mmol, 1.2 equiv.), K_2CO_3 (83 mg, 0.600 mmol, 1.2 equiv.), urea **4p** (4.5 mg, 0.025 mmol, 0.05 equiv.), $Pd(OAc)_2$ (2.8 mg, 0.0125 mmol, 0.025 equiv.), and 95:5 anisole/DMF (1 mL, 0.5M) were used. The crude material was purified by flash column chromatography (silica, hexanes/ethyl acetate 4:1) to obtain products **3m** and **3m'** (88:12 r.r.) as pale yellow oil.

Run 1: (177 mg, 0.390 mmol, 78%)
Run 2: (175 mg, 0.386 mmol, 77%)
Run 3: (166 mg, 0.366 mmol, 73%)
Average yield: 76%

^{1}H NMR (500 MHz, $CDCl_3$) δ 7.94 – 7.90 (m, 1H), 7.71 – 7.67 (m, 2H), 7.62 (d, *J* = 8.3 Hz, 2H), 7.20 (d, *J* = 8.0 Hz, 2H), 5.11 (s, 1H), 5.09 – 5.03 (m, 1H), 4.90 (s, 1H), 4.76 (dd, *J* = 10.4, 3.7 Hz, 1H), 4.36 – 4.29 (m, 2H), 3.09 (dd, *J* = 16.4, 10.5 Hz, 1H), 2.75 (dd, *J* = 16.4, 3.6 Hz, 1H), 2.36 (s, 3H), 2.15 (dd, *J* = 15.0, 7.3 Hz, 2H), 2.03 (dq, *J* = 15.0, 7.5 Hz, 1H), 1.99 – 1.90 (m, 1H), 1.64 (d, *J* = 18.1 Hz, 3H), 1.58 (s, 3H), 1.36 (t, *J* = 7.1 Hz, 3H).

^{13}C NMR (125 MHz, $CDCl_3$) δ 166.3, 148.2, 146.3, 144.4, 135.3, 132.2, 131.5, 130.3, 129.8, 127.2, 126.5, 126.5, 123.9, 115.3, 111.0, 67.1, 61.0, 34.7, 31.0, 26.2, 25.8, 21.7, 17.9, 14.5.

HRMS (ESI) *m/z* calculated for $C_{26}H_{32}NO_4S$ $[M+H]^+$: 454.2052, found 454.2039.

1-(2-(6-Methylhepta-1,5-dien-2-yl)-*N*-tosylindolin-5-yl)ethan-1-one (3n)

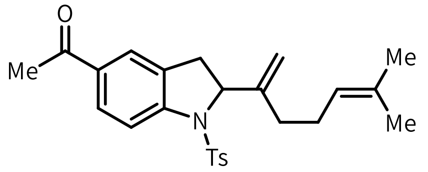

Prepared according to the general procedure. Bromoaniline **1n** (184 mg, 0.500 mmol, 1.0 equiv.), myrcene **2a** (103 uL, 0.600 mmol, 1.2 equiv.), K_2CO_3 (83 mg, 0.600 mmol, 1.2 equiv.), urea **4p** (4.5 mg, 0.025 mmol, 0.05 equiv.), $Pd(OAc)_2$ (2.8 mg, 0.0125 mmol, 0.025 equiv.), and 95:5 anisole/DMF (1 mL, 0.5M) were used. The crude material was purified by flash column chromatography (silica, hexanes/ethyl acetate 4:1) to obtain products **3n** and **3n'** (88:12 r.r.) as pale yellow oil.

Run 1: (141 mg, 0.333 mmol, 67%)
Run 2: (151 mg, 0.356 mmol, 71%)
Run 3: (139 mg, 0.328 mmol, 66%)
Average yield: 68%

^1H NMR (500 MHz, $CDCl_3$) δ 7.83 (dd, *J* = 8.4, 1.7 Hz, 1H), 7.70 (d, *J* = 8.5 Hz, 1H), 7.66 (d, *J* = 1.1 Hz, 1H), 7.63 (d, *J* = 8.3 Hz, 2H), 7.22 (d, *J* = 8.0 Hz, 2H), 5.11 (s, 1H), 5.09 – 5.03 (m, 1H), 4.90 (s, 1H), 4.77 (dd, *J* = 10.4, 3.7 Hz, 1H), 3.11 (dd, *J* = 16.4, 10.4 Hz, 1H), 2.77 (dd, *J* = 16.4, 3.7 Hz, 1H), 2.54 (s, 3H), 2.37 (s, 3H), 2.19 – 2.12 (m, 2H), 2.08 – 1.91 (m, 2H), 1.67 (d, *J* = 1.0 Hz, 3H), 1.58 (s, 3H).

^{13}C NMR (120 MHz, $CDCl_3$) δ 197.0, 148.2, 146.6, 144.5, 135.3, 133.5, 132.2, 131.7, 129.9, 129.6, 127.2, 125.3, 123.8, 115.1, 111.1, 67.2, 34.7, 31.0, 26.6, 26.2, 25.8, 21.7, 17.9.

HRMS (ESI) *m/z* calculated for $C_{25}H_{30}NO_3S$ $[M+H]^+$: 424.1946, found 424.1946.

***N,N*-diisopropyl-2-(6-methylhepta-1,5-dien-2-yl)-*N*-tosylindoline-5-carboxamide (3o)**

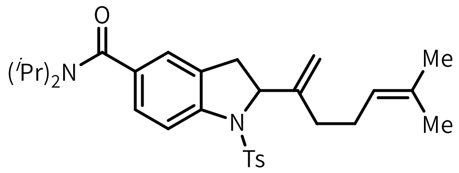

Prepared according to the general procedure. Bromoaniline **1o** (184 mg, 0.500 mmol, 1.0 equiv.), myrcene **2a** (103 uL, 0.600 mmol, 1.2 equiv.), K_2CO_3 (83 mg, 0.600 mmol, 1.2 equiv.), urea **4p** (9.0 mg, 0.050 mmol, 0.010 equiv.), $Pd(OAc)_2$ (5.6 mg, 0.025 mmol, 0.05 equiv.), and 95:5 anisole/DMF (1 mL, 0.5M) were used. The crude material was purified by flash column chromatography (silica, hexanes/ethyl acetate 4:1) to obtain products **3o** and **3o'** (88:12 r.r.) as pale yellow oil.

Run 1: (203 mg, 0.399 mmol, 80%)
Run 2: (190 mg, 0.374 mmol, 75%)
Run 3: (196 mg, 0.385 mmol, 77%)
Average yield: 77%

^1H NMR (500 MHz, $CDCl_3$, 55 °C) δ 7.64 – 7.59 (m, 3H), 7.19 (d, *J* = 8.0 Hz, 2H), 7.16 – 7.11 (m, 1H), 7.01 (s, 1H), 5.11 (s, 1H), 5.11 – 5.07 (m, 1H), 4.90 (s, 1H), 4.74 (dd, *J* = 10.3, 3.6 Hz, 1H), 3.73 – 3.64 (m, 2H), 3.05 (dd, *J* = 16.3, 10.3 Hz, 1H), 2.72 (dd, *J* = 16.3, 3.7 Hz, 1H), 2.37 (s, 3H), 2.22 – 1.96 (m, 4H), 1.68 (s, 3H), 1.60 (s, 3H), 1.33 (s, 12H).

^{13}C NMR (100 MHz, $CDCl_3$, 55 °C) δ 170.8, 148.6, 144.1, 143.0, 135.9, 135.3, 132.0, 131.9, 129.7, 127.5, 125.7, 124.2, 123.2, 115.9, 111.1, 66.8, 49.4, 35.1, 31.6, 26.6, 25.7, 21.6, 21.04, 21.00, 17.8.

HRMS (ESI) *m/z* calculated for $C_{30}H_{41}N_2O_3S$ $[M+H]^+$: 509.2838, found 509.2843.

1,3-Diene Scope

(*E*)-2-Styryl-*N*-tosylindoline (3ab)

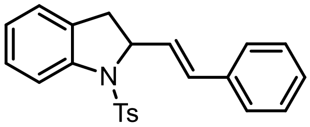

Prepared according to the general procedure. Bromoaniline **1a** (163 mg, 0.500 mmol, 1.00 equiv.), diene **2b** (84.6mg, 0.650 mmol, 1.30 equiv.), K_2CO_3 (82.9 mg, 0.600 mmol, 1.20 equiv.), urea **4p** (2.7 mg, 0.0150 mmol, 3.00 mol %), $Pd(OAc)_2$ (1.7 mg, 0.00750 mmol, 1.50 mol%), anisole/DMF (1 mL, 95:5) were used. Crude material was purified via column chromatography on SiO_2 using 5% → 10% EtOAc/hexanes. Pure product was isolated as white foam (>95:5 *E/Z*).

Run 1: (164.2 mg, 0.437 mmol, 88%), <5% rsm.
Run 2: (172.8 mg, 0.460 mmol, 92%), <5% rsm.
Run 3: (165.6 mg, 0.441 mmol, 88%), <5% rsm.
Average yield: 89%

^1H NMR (400 MHz, $CDCl_3$) δ 7.68 (d, *J* = 8.0 Hz, 1H), 7.62 (d, *J* = 8.2 Hz, 2H), 7.33 – 7.28 (m, 4H), 7.23 (d, *J* = 7.0 Hz, 2H), 7.15 (d, *J* = 8.1 Hz, 2H), 7.09 – 7.01 (m, 2H), 6.69 (d, *J* = 15.8 Hz, 1H), 6.16 (dd, *J* = 15.8, 7.2 Hz, 1H), 4.97 (td, *J* = 6.9 Hz, 2.6 Hz, 1H), 3.08 (dd, *J* = 16.0, 9.6 Hz, 1H), 2.73 (dd, *J* = 16.0, 2.6 Hz, 1H), 2.37 (d, *J* = 19.7 Hz, 3H).

^{13}C NMR (100 MHz, $CDCl_3$) δ 144.0, 141.6, 136.4, 135.9, 135.5, 131.3, 129.7, 128.8, 128.6, 128.0, 127.9, 127.3, 126.8, 125.3, 124.5, 116.7, 64.1, 35.7, 21.7. Spectral data agree with that reported in the literature.[32]

HRMS (ESI) *m/z* calculated for $C_{23}H_{22}NO_2S$ $[M+H]^+$: 376.1366, found 376.1355.

(*E*)-2-(4-Methoxystyryl)-*N*-tosylindoline (3ac)

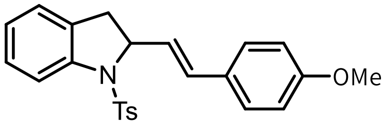

Prepared according to the general procedure. Bromoaniline **1a** (163 mg, 0.500 mmol, 1.00 equiv.), diene **2c** (104 mg, 0.650 mmol, 1.30 equiv.), K_2CO_3 (82.9 mg, 0.600 mmol, 1.20 equiv.), urea **4p** (2.7 mg, 0.0150 mmol, 3.00 mol %), $Pd(OAc)_2$ (1.7 mg, 0.00750 mmol, 1.50 mol%), anisole/DMF (1 mL, 95:5) were used. Crude material was purified via column chromatography on SiO_2 with gradient solvent mixture of hexanes, 5% →10% EtOAc /hexanes. Pure product was isolated as white foam (>95:5 *E/Z*).

Run 1: (177.1 mg, 0.437 mmol, 87%), <5% rsm.
Run 2: (185.3 mg, 0.457 mmol, 91%), <5% rsm.
Run 3: (186.6 mg, 0.460 mmol, 92%), <5% rsm.
Average yield: 90%

^1H NMR (400 MHz, $CDCl_3$) δ 7.66 (d, *J* = 8.1 Hz, 1H), 7.62 (d, *J* = 8.3 Hz, 2H), 7.25 – 7.20 (m, 2H), 7.15 (d, *J* = 8.1 Hz, 2H), 7.08 (d, *J* = 7.2 Hz, 1H), 7.02 (t, *J* = 7.3 Hz, 1H), 6.82 (d, *J* = 8.7 Hz,

2H), 6.63 (d, *J* = 15.7 Hz, 1H), 6.02 (dd, *J* = 15.7, 7.3 Hz, 1H), 5.00 – 4.90 (m, 1H), 3.80 (s, 3H), 3.08 (dd, *J* = 16.0, 9.6 Hz, 1H), 2.72 (dd, *J* = 16.1, 2.6 Hz, 1H), 2.34 (s, 3H).

^{13}C NMR (100 MHz, $CDCl_3$) δ 159.5, 143.9, 141.5, 135.9, 131.4, 130.8, 129.6, 129.2, 128.0, 127.9, 127.3, 126.6, 125.3, 124.4, 116.6, 114.0, 64.2, 55.4, 35.7, 21.6.

HRMS (ESI) *m/z* calculated for $C_{24}H_{24}NO_3S$ $[M+H]^+$: 406.1472, found 406.1462.

(*E*)-*N*-Tosyl-2-(3,4,5-trimethoxystyryl)indoline (3ad)

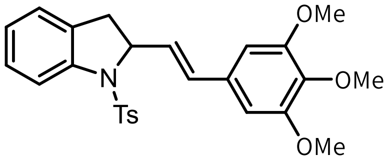

Prepared according to the general procedure. Bromoaniline **1a** (163 mg, 0.500 mmol, 1.00 equiv.), diene **2d** (143 mg, 0.650 mmol, 1.3 equiv.), K_2CO_3 (82.9 mg, 0.600 mmol, 1.20 equiv.), urea **4p** (2.7 mg, 0.0150 mmol, 3.00 mol %), $Pd(OAc)_2$ (1.7 mg, 0.00750 mmol, 1.5 mol%), 95:5 anisole/DMF (1 mL, 0.5M) were used. Crude material was purified via column chromatography on SiO_2 using 5%→ 25% EtOAc/hexanes. Pure product was isolated as a white foam (>95:5 *E/Z*).

<u>Run 1:</u> (199 mg, 0.427 mmol, 85%), <5% rsm.
<u>Run 2:</u> (199 mg, 0.428 mmol, 86%), <5% rsm.
<u>Run 3:</u> (198 mg, 0.451 mmol, 85%), <5% rsm.
Average yield: 85%

^{1}H NMR (400 MHz, $CDCl_3$) δ 7.68 (d, *J* = 8.1 Hz, 1H), 7.62 (d, *J* = 8.2 Hz, 2H), 7.24 – 7.22 (m, 1H), 7.16 (d, *J* = 8.1 Hz, 2H), 7.10 – 7.01 (m, 2H), 6.63 (d, *J* = 15.6 Hz, 1H), 6.55 (s, 2H), 6.09 (dd, *J* = 15.7, 7.1 Hz, 1H), 5.01 – 4.89 (m, 1H), 3.84 (s, 6H), 3.83 (s, 3H), 3.09 (dd, *J* = 16.1, 9.7 Hz, 1H), 2.74 (dd, *J* = 16.1, 2.4 Hz, 1H), 2.34 (s, 3H).

^{13}C NMR (100 MHz, $CDCl_3$) δ 153.3, 144.0, 141.5, 138.0, 135.8, 132.1, 131.3, 129.7, 128.3, 128.0, 127.3, 125.3, 124.5, 116.7, 103.8, 63.9, 61.0, 56.2, 35.6, 21.6.

HRMS (ESI) *m/z* calculated for $C_{26}H_{28}NO_5S$ $[M+H]^+$: 466.1716, found 466.1674.

(*E*)-*N*-Tosyl-2-(3-(trifluoromethyl)styryl)indoline (3ae)

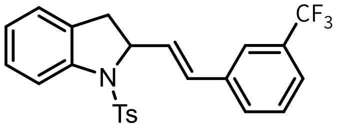

Prepared according to the general procedure. Bromoaniline **1a** (163 mg, 0.500 mmol, 1.00 equiv.), diene **2e** (129 mg, 0.650 mmol, 1.30 equiv.), K_2CO_3 (82.9 mg, 0.600 mmol, 1.20 equiv.), urea **4p** (2.7 mg, 0.0150 mmol, 3.00 mol %), $Pd(OAc)_2$ (1.7 mg, 0.00750 mmol, 1.50 mol%), anisole/DMF (1 mL, 95:5) were used. Crude material was purified via column chromatography on SiO_2 with 5% →10% EtOAc /hexanes. Pure product was isolated as white foam (>95:5 *E/Z*).

<u>Run 1:</u> (144.9 mg, 0.327 mmol, 65%), <5% rsm.
<u>Run 2:</u> (148.5 mg, 0.335 mmol, 67%), <5% rsm.
<u>Run 3:</u> (143.7 mg, 0.324 mmol, 65%), <5% rsm.
Average yield: 66%

^{1}H NMR (400 MHz, $CDCl_3$) δ 7.72 (d, *J* = 8.0 Hz, 1H), 7.63 (d, *J* = 8.0 Hz, 2H), 7.55 (s, 1H), 7.50 – 7.46 (m, 2H), 7.4 – 7.38 (m, 1H), 7.27 – 7.24 (m, 1H), 7.16 (d, *J* = 7.9 Hz, 2H), 7.10 – 7.03 (m, 2H), 6.74 (d, *J* = 15.8 Hz, 1H), 6.25 (dd, *J* = 15.8, 6.8 Hz, 1H), 5.01 – 4.98 (m, 1H), 3.11 (dd, *J* = 16.0, 9.7 Hz, 1H), 2.75 (dd, *J* = 16.0, 1,5 Hz, 1H), 2.34 (s, 3H).

^{13}C NMR (100 MHz, $CDCl_3$) δ 144.1, 141.4, 137.2, 135.7, 131.1, 130.8, 130.1, 129.8, 129.7, 129.1, 128.1, 127.2, 125.4, 124.7, 124.4, 124.4 (q, *J* = 3.5 Hz), 123.3 (q, *J* = 3.5 Hz), 122.8, 116.7, 63.6, 35.5, 21.6.

HRMS (ESI) *m/z* calculated for $C_{24}H_{21}F_3NO_2S$ $[M+H]^+$: 444.1240, found 444.1233.

(*E*)-2-(2-(2,3-Dihydrobenzofuran-5-yl)vinyl)-*N*-tosylindoline (3af)

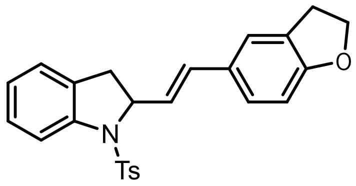

Prepared according to the general procedure. Bromoaniline **1a** (163 mg, 0.500 mmol, 1.00 equiv.), diene **2f** (111.9 mg, 0.650 mmol, 1.30 equiv.), K_2CO_3 (82.9 mg, 0.600 mmol, 1.20 equiv.), urea **4p** (4.5 mg, 0.0250 mmol, 5 mol %), $Pd(OAc)_2$ (2.8 mg, 0.0125 mmol, 2.5 mol%), anisole/DMF (1 mL, 95:5) were used. Crude material was purified via column chromatography on SiO_2 using 5% → 15% EtOAc/hexanes to afford product as an off–white foam (>95:5 *E/Z*).

<u>Run 1:</u> (157.5 mg, 0.377 mmol, 75%), <5% rsm.
<u>Run 2:</u> (158.5 mg, 0.380 mmol, 76%), <5% rsm.
<u>Run 3:</u> (159.2 mg, 0.381 mmol, 76%), <5% rsm.
Average: 76% yield

^{1}H NMR (400 MHz, $CDCl_3$) δ 7.66 (d, *J* = 8.1 Hz, 1H), 7.62 (d, *J* = 8.2 Hz, 2H), 7.25 – 7.12 (m, 4H), 7.04 (m, 3H), 6.69 (d, *J* = 8.2 Hz, 1H), 6.61 (d, *J* = 15.7 Hz, 1H), 5.99 (dd, *J* = 15.7, 7.3 Hz, 1H), 5.02 – 4.90 (m, 1H), 4.56 (t, *J* = 8.7 Hz, 2H), 3.16 (t, *J* = 8.7 Hz, 2H), 3.07 (dd, *J* = 16.0, 9.5 Hz, 1H), 2.72 (dd, *J* = 16.0, 2.5 Hz, 1H), 2.35 (s, 3H).

^{13}C NMR (100 MHz, $CDCl_3$) δ 160.1, 143.8, 141.6, 136.0, 131.4, 131.2, 129.7, 129.3, 127.9, 127.5, 127.3, 127.3, 126.0, 125.3, 124.4, 123.1, 116.6, 109.3, 71.6, 64.3, 35.7, 29.6, 21.6.

HRMS (ESI) *m/z* calculated for $C_{25}H_{24}NO_3S$ $[M+H]^+$: 418.1472, found 418.1462.

(*E*)-2-(2-(Thiophen-2-yl)vinyl)-*N*-tosylindoline (3ag)

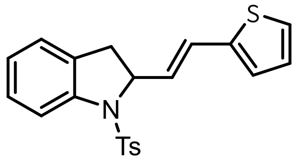

Prepared according to the general procedure. Bromoaniline **1a** (163 mg, 0.500 mmol, 1.00 equiv.), diene **2g** (88.5 mg, 0.650 mmol, 1.30 equiv.), K_2CO_3 (82.9 mg, 0.600 mmol, 1.20 equiv.), urea **4p** (4.5 mg, 0.0250 mmol, 5 mol %), $Pd(OAc)_2$ (2.8 mg, 0.0125 mmol, 2.5 mol %), anisole/DMF (1 mL, 95:5) were used. Crude material was purified via column chromatography on SiO_2 using 5% → 10% EtOAc/hexanes to afford product as off white solid (>95:5 *E/Z*).

<u>Run 1:</u> (145.1 mg, 0.380 mmol, 76%), <5% rsm.
<u>Run 2:</u> (136.2 mg, 0.357 mmol, 71%), <5% rsm.
<u>Run 3:</u> (138.7 mg, 0.364 mmol, 73%), <5% rsm.
Average: 73% yield

^{1}H NMR (400 MHz, $CDCl_3$) δ 7.67 (d, *J* = 8.1 Hz, 1H), 7.61 (d, *J* = 8.1 Hz, 2H), 7.25 – 7.11 (m, 4H), 7.05 (dt, *J* = 14.7, 7.3 Hz, 2H), 6.98 – 6.91 (m, 2H), 6.85 (d, *J* = 15.5 Hz, 1H), 5.98 (dd, *J* = 15.5, 6.9 Hz, 1H), 4.94 (m, 1H), 3.06 (dd, *J* = 16.0, 9.7 Hz, 1H), 2.71 (dd, *J* = 16.0, 2.3 Hz, 1H), 2.35 (s, 3H).

^{13}C NMR (100 MHz, $CDCl_3$) δ 144.0, 141.5, 141.4, 135.8, 131.2, 129.7, 128.2, 128.0, 127.5, 127.3, 126.6, 125.3, 124.7 (2C), 124.6, 116.8, 63.6, 35.5, 21.6.

HRMS (ESI) *m/z* calculated for $C_{21}H_{20}NO_2S_2$ $[M+H]^+$: 382.0930, found 382.0919.

(*E*)-1-Me-3-(2-(*N*-tosylindolin-2-yl)vinyl)-1H-indole (3ah)

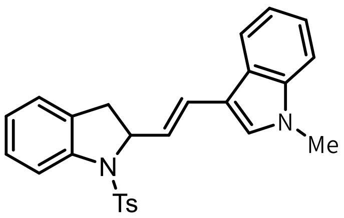

Prepared according to the general procedure. Bromoaniline **1a** (163 mg, 0.500 mmol, 1.00 equiv.), diene **2h** (119.1 mg, 0.650 mmol, 1.30 equiv.), K_2CO_3 (82.9 mg, 0.600 mmol, 1.20 equiv.), urea **4p** (4.5 mg, 0.0250 mmol, 5 mol %), $Pd(OAc)_2$ (2.8 mg, 0.0125 mmol, 2.5 mol%), anisole/DMF (1 mL, 95:5) were used. Crude material was purified via column chromatography on SiO_2 using 5% → 15% EtOAc/hexanes to afford product as an off white solid (>95:5 *E/Z*).

<u>Run 1:</u> (105.3 mg, 0.246 mmol, 49%), <5% rsm.
<u>Run 2:</u> (110.5 mg, 0.258 mmol, 52%), <5% rsm.
<u>Run 3:</u> (109.9 mg, 0.256 mmol, 51%), <5% rsm.
Average: 51% yield

^1H NMR (400 MHz, $CDCl_3$) δ 7.66 (d, *J* = 8.0 Hz, 3H), 7.30 – 7.26 (m, 2H), 7.25 – 7.19 (m, 2H), 7.10 (m, 5H), 7.05 – 6.97 (m, 1H), 6.85 (d, *J* = 15.8 Hz, 1H), 6.18 – 5.99 (m, 1H), 5.02 (dd, *J* = 12.0, 4.7 Hz, 1H), 3.75 (s, 3H), 3.15 (dd, *J* = 16.0, 9.5 Hz, 1H), 2.79 (dd, *J* = 16.0, 2.1 Hz, 1H), 2.29 (s, 1H).

^{13}C NMR (100 MHz, $CDCl_3$) δ 143.7, 141.7, 137.7, 136.3, 131.5, 129.6, 129.1, 127.9, 127.3, 126.1, 125.3, 124.6, 124.5, 124.2, 122.2, 120.4, 120.0, 116.4, 112.7, 109.5, 65.2, 36.1, 33.0, 21.6.

HRMS (ESI) *m/z* calculated for $C_{26}H_{25}N_2O_2S$ $[M+H]^+$: 429.1631, found 429.1625.

(*E*)-4-(*N*-Tosylindolin-2-yl)but-3-en-1-ol (3ai)

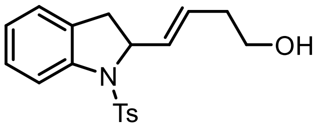

Prepared according to the general procedure. Bromoaniline **1a** (163 mg, 0.500 mmol, 1.00 equiv.), diene **2i** (63.8 mg, 0.650 mmol, 1.30 equiv.), K_2CO_3 (82.9 mg, 0.600 mmol, 1.20 equiv.), urea **4p** (4.5 mg, 0.0250 mmol, 5 mol %), $Pd(OAc)_2$ (2.8 mg, 0.0125 mmol, 2.5 mol%), anisole/DMF (1 mL, 95:5) were used. Crude material was purified via column chromatography on SiO_2 using 50% → 60% EtOAc/hexanes to afford product as white foam (>95:5 *E/Z*).

<u>Run 1:</u> (147.6 mg, 0.429 mmol, 86%), <5% rsm.
<u>Run 2:</u> (146.7 mg, 0.427 mmol, 85%), <5% rsm.
<u>Run 3:</u> (148.1 mg, 0.431 mmol, 86%), <5% rsm.
Average yield: 86%

^1H NMR (400 MHz, $CDCl_3$) δ 7.64 (d, *J* = 8.1 Hz, 1H), 7.59 (d, *J* = 8.2 Hz, 2H), 7.22 – 7.18 (m, *J* = 8.0 Hz, 3H), 7.05 – 6.98 (m, 2H), 5.81 – 5.65 (m, 2H), 4.73 – 4.59 (m, 1H), 3.67 (bs, 2H), 2.99 (dd, *J* = 16.0, 9.7 Hz, 1H), 2.67 (dd, *J* = 16.1, 4.0 Hz, 1H), 2.36 (s, 3H), 2.30 (dd, *J* = 12.3, 6.2 Hz, 2H), 1.77 (bs, 1H).

^{13}C NMR (100 MHz, $CDCl_3$) δ 144.1, 141.6, 135.3, 133.2, 131.0, 129.7, 128.8, 128.0, 127.3, 125.2, 124.5, 116.6, 63.9, 61.7, 35.6 (2C), 21.7.

HRMS (ESI) *m/z* calculated for $C_{19}H_{22}NO_3S$ $[M+H]^+$: 344.1315, found 344.1309.

(*E*)-2-(4-((*tert*-Butyldimethylsilyl)oxy)but-1-en-1-yl)-*N*-tosylindoline (3aj)

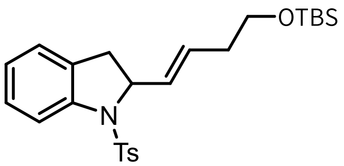

Prepared according to the general procedure. Bromoaniline **1a** (163 mg, 0.500 mmol, 1.00 equiv.), diene **2j** (138.1 mg, 0.650 mmol, 1.30 equiv.), K_2CO_3 (82.9 mg, 0.600 mmol, 1.20 equiv.), urea **4p** (4.5 mg, 0.0250 mmol, 5 mol %), $Pd(OAc)_2$ (2.8 mg, 0.0125 mmol, 2.5 mol%), anisole/DMF (1 mL, 95:5) were used. Crude material was purified via column chromatography on SiO_2 using 5% → 15% EtOAc/hexanes to afford product as colorless oil (>95:5 *E/Z*).

Run 1: (150.3 mg, 0.328 mmol, 66%), <5% rsm.
Run 2: (155.0 mg, 0.333 mmol, 68%), <5% rsm.
Run 3: (158.0 mg, 0.345 mmol, 69%), <5% rsm.
Average: 68 % yield

^{1}H NMR (400 MHz, $CDCl_3$) δ 7.63 (d, *J* = 8.1 Hz, 1H), 7.57 (d, *J* = 8.3 Hz, 2H), 7.24 – 7.13 (m, 3H), 7.01 (tt, *J* = 7.4, 3.5 Hz, 2H), 5.85 – 5.70 (m, 1H), 5.55 (dd, *J* = 15.3, 6.8 Hz, 1H), 4.79 – 4.65 (m, 1H), 3.61 (t, *J* = 6.8 Hz, 2H), 2.93 (dd, *J* = 16.0, 9.5 Hz, 1H), 2.61 (dd, *J* = 16.0, 2.9 Hz, 1H), 2.35 (s, 3H), 2.28 – 2.13 (m, 2H), 0.86 (s, *J* = 2.6 Hz, 9H), 0.01 (d, *J* = 3.2 Hz, 6H).

^{13}C NMR (100 MHz, $CDCl_3$) δ 143.8, 141.6, 135.8, 131.6, 131.5, 129.6, 129.0, 127.9, 127.3, 125.2, 124.5, 117.0, 63.8, 62.7, 35.8, 35.5, 26.1, 21.7, 18.5, –5.1.

HRMS (ESI) *m/z* calculated for $C_{25}H_{36}NO_3SSi$ $[M+H]^+$: 458.2180, found 344.1308 which corresponds to TBS deprotected compound 344.1315.

(*E*)-2-(4-(*N*-Tosylindolin-2-yl)but-3-en-1-yl)isoindoline-1,3-dione (3ak)

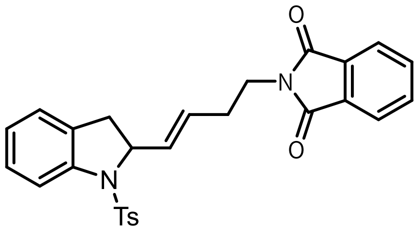

Prepared according to the general procedure. Bromoaniline **1a** (163 mg, 0.500 mmol, 1.00 equiv.), diene **2k** (147.7 mg, 0.650 mmol, 1.30 equiv.), K_2CO_3 (82.9 mg, 0.600 mmol, 1.20 equiv.), urea **4p** (4.5 mg, 0.0250 mmol, 5 mol %), $Pd(OAc)_2$ (2.8 mg, 0.0125 mmol, 2.5 mol%), anisole/DMF (1 mL, 95:5) were used. Crude material was purified via column chromatography on SiO_2 using 10% → 20% EtOAc/hexanes to afford product as white solid (>95:5 *E/Z*).

Run 1: (167.0 mg, 0.365 mmol, 73%), <5% rsm.
Run 2: (166.2 mg, 0.363 mmol, 73%), <5% rsm.
Run 3: (168.4 mg, 0.368 mmol, 74%), <5% rsm.
Average: 73% yield

^{1}H NMR (400 MHz, $CDCl_3$) δ 7.79 (m, 2H), 7.72 – 7.67 (m, 2H),7.57 (d, *J* = 8.1 Hz, 1H), 7.54 (d, *J* = 8.2, Hz, 2H) 7.17 (m, 3H), 6.99 – 6.92 (m, 2H), 5.82 – 5.69 (m, 1H), 5.55 (dd, *J* = 15.3, 6.9 Hz, 1H), 4.70 – 4.59 (m, 1H), 3.77 – 3.64 (m, 2H), 2.86 (dd, *J* = 16.0, 9.6 Hz, 1H), 2.51 – 2.38 (m, 3H), 2.35 (s, 3H).

^{13}C NMR (100 MHz, $CDCl_3$) δ 168.4, 143.9, 141.5, 135.6, 134.0, 132.8, 132.1, 131.3, 129.7, 128.0, 127.9, 127.2, 125.1, 124.5, 123.3, 116.8, 63.5, 37.3, 35.4, 31.3, 21.7.

HRMS (ESI) *m/z* calculated for $C_{27}H_{25}N_2O_4S$ $[M+H]^+$: 473.1530, found 473.1525.

Ethyl (*E*)-4-(*N*-tosylindolin-2-yl)but-2-enoate (3al)

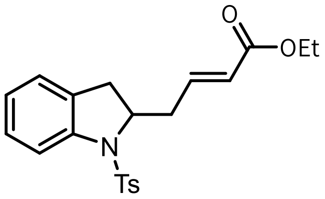

Prepared according to the modified general procedure. Bromoaniline **1a** (163 mg, 0.500 mmol, 1.00 equiv.), K_2CO_3 (69.1 mg, 0.600 mmol, 1.00 equiv.), urea **4p** (4.5 mg, 0.0250 mmol, 5 mol %), $Pd(OAc)_2$ (2.8 mg, 0.0125 mmol, 2.5 mol%), anisole/DMF (1 mL, 1:1) were added to a vial and let stir for 30 min under inert atmosphere in the rt. Diene **2l** (91.1 mg, 0.650 mmol, 1.30 equiv.) was added last in two increments with 8 h interval time reaction was heated to 120 °C. Crude material was purified via column chromatography on SiO_2 using 5% → 15% EtOAc/hexanes to afford product as colorless oil (>95:5 *E/Z*).

Run 1: (97.1 mg, 0.258 mmol, 50%), <5% rsm.
Run 2: (96.9 mg, 0.251 mmol, 50%), <5% rsm.
Run 3: (96.4 mg, 0.250 mmol, 50%), <5% rsm.
Average: 50% yield

^{1}H NMR (400 MHz, $CDCl_3$) δ 7.67 (d, *J* = 8.1 Hz, 1H), 7.53 (d, *J* = 8.2 Hz, 2H), 7.25 – 7.19 (m, 1H), 7.16 (d, *J* = 8.1 Hz, 2H), 7.03 (d, *J* = 4.3 Hz, 2H), 6.95 – 6.85 (m, 1H), 5.88 (d, *J* = 15.7 Hz, 1H), 4.40 – 4.26 (m, 1H), 4.17 (q, 7.1 Hz, 2H), 2.87 – 2.70 (m, 2H), 2.59 – 2.49 (m, 2H), 2.35 (s, 3H), 1.27 (t, *J* = 7.1 Hz, 3H).

^{13}C NMR (100 MHz, $CDCl_3$) δ 166.2, 144.1, 143.4, 141.3, 135.0, 131.5, 129.8, 128.1, 127.1, 125.3, 125.0, 124.7, 117.6, 61.0, 60.5, 39.5, 33.6, 21.7, 14.4.

HRMS (ESI) m/z calculated for $C_{21}H_{24}NO_4S$ [M+H]+: 386.1421, found 386.1412.

2-(Prop-1-en-2-yl)-*N*-tosylindoline (3am)

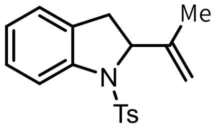

Prepared according to the general procedure. Bromoaniline **1a** (163 mg, 0.500 mmol, 1.00 equiv.), distilled isoprene **2m** (44.3 mg, 0.650 mmol, 1.30 equiv.), K_2CO_3 (82.9 mg, 0.600 mmol, 1.20 equiv.), urea **4p** (4.5 mg, 0.0250 mmol, 5 mol %), $Pd(OAc)_2$ (2.8 mg, 0.0125 mmol, 2.5 mol%), anisole/DMF (1 mL, 95:5) were used. Crude material was purified via column chromatography on SiO_2 using hexanes → 10% EtOAc/hexanes to afford product as white solid (97:3 r.r.).

Run 1: (117.5 mg, 0.375 mmol, 75%), <5% rsm.
Run 2: (112.9 mg, 0.360 mmol, 72%), <5% rsm.
Run 3: (109.4 mg, 0.349 mmol, 69%), <5% rsm.
Average: 72% yield

^{1}H NMR (400 MHz, $CDCl_3$) δ 7.68 (d, *J* = 8.1 Hz, 1H), 7.60 (d, *J* = 8.2 Hz, 2H), 7.04 – 6.98 (m, 3H), 7.08 – 6.94 (m, 2H), 5.07 (s, 1H), 4.86 (s, 1H), 4.66 (dd, *J* = 10.1, 3.7 Hz, 1H), 2.98 (dd, *J* = 16.2, 10.2 Hz, 1H), 2.69 (dd, *J* = 16.3, 3.7 Hz, 1H), 2.36 (s, 2H), 1.70 (s, 3H).

^{13}C NMR (100 MHz, CDCl3) δ 144.5, 144.0, 142.1, 135.2, 131.5, 129.7, 127.9, 127.3, 125.0, 124.5, 116.5, 112.4, 66.9, 34.4, 21.7, 17.8. Spectral data agree with that reported in the literature.[32]

HRMS (ESI) *m/z* calculated for $C_{18}H_{20}NO_2S$ $[M+H]^+$: 314.1209, found 314.1202.

2-(4-(3,3-Dimethyloxiran-2-yl)but-1-en-2-yl)-*N*-tosylindoline (3an)

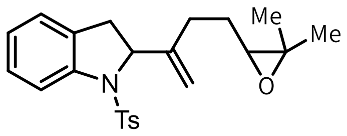

Prepared according to the general procedure. Bromoaniline **1a** (163 mg, 0.500 mmol, 1.00 equiv.), diene **2n** (72.9 mg, 0.650 mmol, 1.30 equiv.), K_2CO_3 (82.9 mg, 0.600 mmol, 1.20 equiv.), urea **4p** (4.5 mg, 0.0250 mmol, 5 mol %), $Pd(OAc)_2$ (2.8 mg, 0.0125 mmol, 2.5 mol %), anisole/DMF (1 mL, 95:5) were used. Crude material was purified via column chromatography on SiO_2 using 30% → 50% EtOAc/hexanes to afford product as off–white viscous oil (>99:1 r.r.).

Run 1: (122.2 mg, 0.307 mmol, 62%), <5% rsm.
Run 2: (124.5 mg, 0.313 mmol, 63%), <5% rsm.
Run 3: (131.7 mg, 0.331 mmol, 66%), <5% rsm.
Average: 64% yield

^{1}H NMR (400 MHz, $CDCl_3$) δ 7.68 (d, *J* = 8.1 Hz, 1H), 7.57 (d, *J* = 8.1 Hz, 2H), 7.24 – 7.14 (m, 3H), 7.04 – 6.96 (m, 2H), 5.16 (d, *J* = 8.4 Hz, 1H), 4.90 (d, *J* = 3.1 Hz, 1H), 4.72 – 4.62 (m, 1H), 2.97 (dd, *J* = 16.2, 10.2 Hz, 1H), 2.83 – 2.75 (m, 1H), 2.73 – 2.66 (m, 1H), 2.35 (s, 3H), 2.31 – 2.01 (m, 2H), 1.90 – 1.57 (m, 2H), 1.30 (d, *J* = 3.7 Hz, 3H), 1.25 (d, *J* = 13.5 Hz, 3H).

^{13}C NMR (100 MHz, $CDCl_3$) δ 147.9, 147.8, 144.0, 142.1, 142.1, 135.1, 135.0, 131.5, 129.7, 128.0, 127.3, 1251, 124.7, 116.9, 116.8, 111.1, 111.1, 66.3, 66.2, 64.1, 63.9, 58.7, 34.9, 28.1, 28.0, 27.3, 27.2, 25.0, 21.7, 18.9, 18.9.

HRMS (ESI) *m/z* calculated for $C_{23}H_{28}NO_3S$ $[M+H]^+$: 398.1785, found 398.1772.

4-(*N*-Tosylindolin-2-yl)pent-4-en-1-ol (3ao)

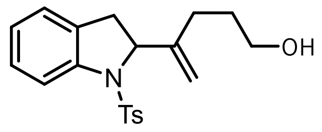

Prepared according to the general procedure. Bromoaniline **1a** (163 mg, 0.500 mmol, 1.00 equiv.), diene **2o** (99.0 mg, 0.650 mmol, 1.30 equiv.), K_2CO_3 (82.9 mg, 0.600 mmol, 1.20 equiv.), urea **4p** (4.5 mg, 0.0250 mmol, 5 mol %), $Pd(OAc)_2$ (2.8 mg, 0.0125 mmol, 2.5 mol%), anisole/DMF (1 mL, 95:5) were used. Crude material was purified via column chromatography on SiO_2 using 30% → 50% EtOAc/hexanes to afford product as colorless viscous oil (>99:1 r.r.).

Run 1: (155.8 mg, 0.436 mmol, 87%), <5% rsm.
Run 2: (154.1 mg, 0.431 mmol, 85%), <5% rsm.
Run 3: (152.0 mg, 0.425 mmol, 85%), <5% rsm.
Average: 86% yield

^{1}H NMR (400 MHz, $CDCl_3$) δ 7.68 (d, *J* = 8.1 Hz, 1H), 7.57 (d, *J* = 8.2 Hz, 2H), 7.24 – 7.12 (m, 3H), 7.05 – 6.95 (m, 2H), 5.14 (s, 1H), 4.90 (s, 1H), 4.67 (dd, *J* = 10.1, 3.6 Hz, 1H), 3.72 – 3.61 (m, 2H), 2.97 (dd, *J* = 16.2, 10.2 Hz, 1H), 2.70 (dd, *J* = 16.2, 3.6 Hz, 1H), 2.35 (s, 3H), 2.27 – 1.97 (m, 2H), 1.92 – 1.72 (m, 2H).

^{13}C NMR (100 MHz, $CDCl_3$) δ 148.0, 144.1, 142.0, 135.0, 131.5, 129.7, 127.9, 127.3, 125.1, 124.6, 116.7, 110.9, 66.2, 62.4, 34.8, 30.6, 27.5, 21.7.

HRMS (ESI) *m/z* calculated for $C_{20}H_{24}NO_3S$ $[M+H]^+$: 358.1472, found 358.1459.

(*E*)-4-(*N*-Tosylindolin-2-yl)pent-3-en-1-ol (3ap)

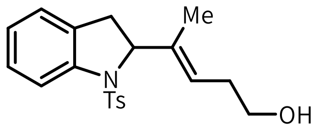

Prepared according to the general procedure. Bromoaniline **1a** (163 mg, 0.500 mmol, 1.00 equiv.), diene **2p** (72.0 mg, 0.650 mmol, 1.30 equiv.), K_2CO_3 (82.9 mg, 0.600 mmol, 1.20 equiv.), urea **4p** (4.5 mg, 0.0250 mmol, 5 mol %), $Pd(OAc)_2$ (2.8 mg, 0.0125 mmol, 2.5 mol%), anisole/DMF (1 mL, 95:5) were used. Crude material was purified via column chromatography on SiO_2 using 35% → 50% EtOAc/hexanes to afford product as white solid (1:1.8 *E/Z*).

<u>Run 1:</u> (92.9 mg, 0.259 mmol, 51.8%), <5% rsm.
<u>Run 2:</u> (89.4 mg, 0.250 mmol, 50.0%), <5% rsm.
<u>Run 3:</u> (95.6 mg, 0.267 mmol, 53.4%), <5% rsm.
Average: 52% yield

E isomer: ^{1}H NMR (400 MHz, $CDCl_3$) δ 7.69 (m, 3H), 7.21–7.20 (m, 3H), 7.03 (m, 2H), 5.26 (t, *J* = 7.6 Hz, 1H), 5.17 (dd, *J* = 10.6, 4.8 Hz, 1H), 3.81–3.69 (m, 2H), 3.14 (dd, *J* = 16.4, 10.7 Hz, 1H), 2.65 (dd, *J* = 16.4, 4.7 Hz, 1H), 2.61–2.52 (m, 1H), 2.36 (s, 3H), 2.42 – 2.29 (m, 1H), 2.17 (s, 1H), 1.65 (s, 3H).

Z isomer: ^{1}H NMR (400 MHz, $CDCl_3$) δ 7.63 (dd, *J* = 17.7, 8.2 Hz, 3H), 7.22–7.17 (m, 3H), 7.03 – 6.94 (m, 2H), 5.53 (t, *J* = 7.2 Hz, 1H), 4.56 (dd, *J* = 10.2, 5.1 Hz, 1H), 3.81–3.69 (m, 2H), 3.05 (dd, *J* = 16.4, 10.3 Hz, 1H), 2.75 (dd, *J* = 16.4, 5.1 Hz, 1H), 2.36 (s, 3H), 2.31–2.22 (m, 2H), 1.94 (s, 1H), 1.61 (s, 3H).

^{13}C NMR (100 MHz, $CDCl_3$) δ 143.1, 143.1, 141.4, 141.3, 138.2, 137.2, 133.9 (2C), 130.1, 130.0, 128.8, 128.7, 126.9 (2C), 126.4, 126.3, 124.0, 123.8, 123.1, 123.1, 122.7, 122.4, 114.8, 114.7, 67.9, 61.3, 61.2, 59.8, 33.7, 33.4, 30.3, 30.3, 20.7, 17.1, 10.9, 10.9.

HRMS (ESI) m/z calculated for $C_{20}H_{24}NO_3S$ [M+H]+: 358.1472, found 358.1467.

(*E*)-2-(*N*-Phenylprop-1-en-2-yl)-1-tosylindoline (3aq)

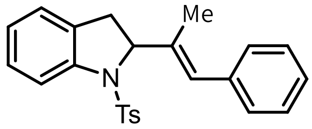

Prepared according to the general procedure. Bromoaniline **1a** (163 mg, 0.500 mmol, 1.00 equiv.), diene **2q** (93.7 mg, 0.650 mmol, 1.30 equiv.), K_2CO_3 (82.9 mg, 0.600 mmol, 1.20 equiv.), urea **4p** (2.7 mg, 0.0150 mmol, 3.00 mol %), $Pd(OAc)_2$ (1.7 mg, 0.00750 mmol, 1.5 mol%), anisole/DMF (1 mL, 95:5) were used. Crude material was purified via column chromatography on SiO_2 using 5% → 10% EtOAc/hexanes. Pure product was isolated as white solid (>95:5 *E/Z*).

<u>Run 1:</u> (148.0 mg, 0.380 mmol, 76%), <5% rsm.
<u>Run 2:</u> (148.5 mg, 0.381 mmol, 76%), <5% rsm.
<u>Run 3:</u> (149.7 mg, 0.384 mmol, 77%), <5% rsm.
Average: 76% yield

^{1}H NMR (400 MHz, $CDCl_3$) δ 7.72 (d, *J* = 8.1 Hz, 1H), 7.67 (d, *J* = 8.2 Hz, 2H), 7.36 – 7.29 (m, 2H), 7.29 – 7.15 (m, 6H), 7.08 – 6.99 (m, 2H), 6.60 (s, 1H), 4.82 (dd, *J* = 10.2, 4.1 Hz, 1H), 3.16 (dd, *J* = 16.4, 10.3 Hz, 1H), 2.82 (dd, *J* = 16.4, 4.0 Hz, 1H), 2.36 (s, 3H), 1.75 (s, 3H).

^{13}C NMR (100 MHz, $CDCl_3$) δ 143.8, 142.3, 137.2, 137.0, 135.3, 131.2, 129.5, 129.1, 128.0, 127.8, 127.2, 126.7, 126.5, 124.8, 124.1, 115.8, 68.9, 34.6, 21.5, 13.3.

HRMS (ESI) *m/z* calculated for $C_{24}H_{24}NO_2S$ $[M+H]^+$: 390.1522, found 344.1517.

(*E*)-2-(Hex-1-en-1-yl)-2-Me-*N*-tosylindoline (3ar)

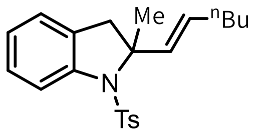

Prepared according to the modified general procedure. Bromoaniline **1a** (163 mg, 0.500 mmol, 1.00 equiv.), diene **2r** (80.7 mg, 0.650 mmol, 1.30 equiv.), K_2CO_3 (82.9 mg, 0.600 mmol, 1.20 equiv.), urea **4p** (4.5 mg, 0.0250 mmol, 5 mol %), $Pd(OAc)_2$ (2.8 mg, 0.0125 mmol, 2.5 mol%), TBACl (138.9 mg, 0.5 mmol, 1.00 equiv.), anisole/DMF (1 mL, 1:1) were used. Crude material was purified via column chromatography on SiO_2 using 5% → 10% EtOAc/hexanes to afford product as colorless oil (>95:5 *E/Z*).

Run 1: (93.7 mg, 0.254 mmol, 51%), <5% rsm.
Run 2: (92.5 mg, 0.250 mmol, 50%), <5% rsm.
Run 3: (92.7 mg, 0.251 mmol, 50%), <5% rsm.
Average: 50% yield

^{1}H NMR (400 MHz, $CDCl_3$) δ 7.75 (d, *J* = 8.0 Hz, 2H), 7.56 (d, *J* = 8.2 Hz, 1H), 7.23 – 7.13 (m, 3H), 7.08 (d, *J* = 7.3 Hz, 1H), 6.93 (m, 1H), 5.75 – 5.65 (m, 1H), 5.59 (d, *J* = 15.8 Hz, 1H), 3.10 (d, *J* = 15.9 Hz, 1H), 2.96 (d, *J* = 15.9 Hz, 1H), 2.38 (s, *J* = 9.5 Hz, 3H), 2.11 – 1.89 (m, 2H), 1.77 (s, 3H), 1.43 – 1.21 (m, 4H), 0.89 (m, 3H).

^{13}C NMR (100 MHz, $CDCl_3$) δ 143.4, 142.1, 139.1, 133.0, 130.4, 129.4, 128.5, 127.8, 127.2, 125.0, 122.8, 114.2, 71.8, 45.3, 32.1, 31.2, 26.3, 22.4, 21.6, 14.1.

HRMS (ESI) *m/z* calculated for $C_{22}H_{28}NO_2S$ $[M+H]^+$: 370.1835, found 370.1827.

Method Limitations

Table S3. Unsuccessful substrates.

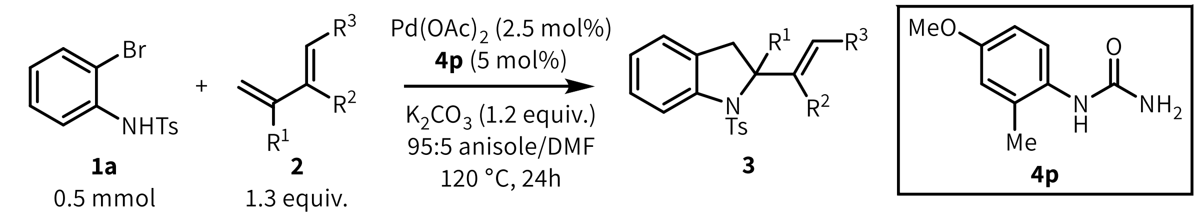

Decompose rapidly

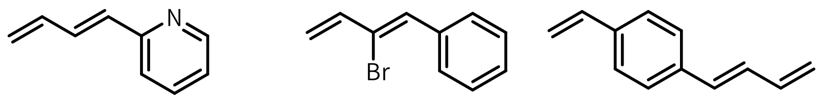

Generally unrective

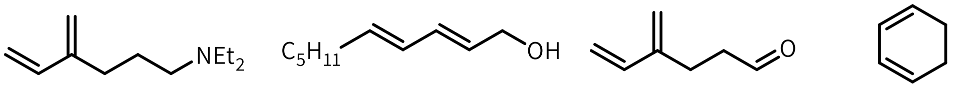

Poor mass balance

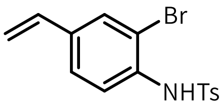

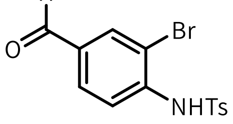

Gram Scale Reaction

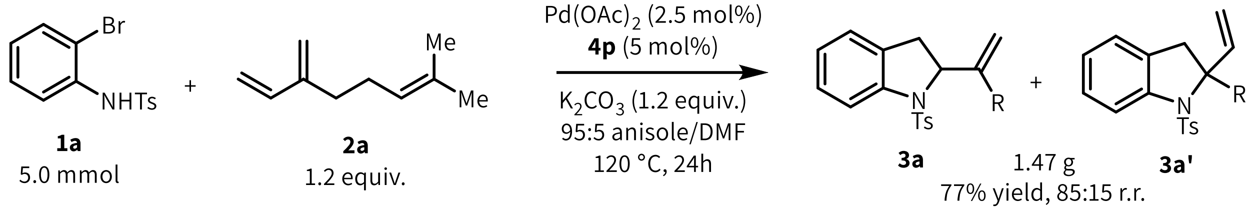

1a
5.0 mmol

2a
1.2 equiv.

3a 1.47 g **3a'**
77% yield, 85:15 r.r.

N-Tosylbromoaniline **1a** (1.63 g, 5.00 mmol, 1.0 equiv.), myrcene **2a** (1.03 mL, 6.00 mmol, 1.2 equiv.), urea **4p** (45 mg, 0.25 mmol, 0.05 equiv.), potassium carbonate (830 mg, 6.00 mmol, 1.2 equiv.), and palladium acetate (28 mg, 0.125 mmol, 0.025 equiv.) were weighed out in the above-mentioned order into a 4-dram vial equipped with a stir bar and a cap with a silicone septum. The vial was then placed under nitrogen atmosphere and charged with 10 mL of a freshly degassed anisole/dimethylformamide (95:5) solvent mixture. The reaction was stirred at 120 °C for 24 h. After cooling to room temperature, the reaction mixture was filtered with ethyl acetate through celite. The solvents were removed under reduced pressure and the crude mixture was purified by flash column chromatography (silica, hexanes/ethyl acetate 19:1) to obtain products **3a** and **3a'** (85:15 r.r.).

<u>Run 1:</u> (1.51 g, 3.96 mmol, 79%)
<u>Run 2:</u> (1.42 g, 3.72 mmol, 74%)
Average: 77% yield

Model *N*-phenylurea-$PdCl_2$ complex

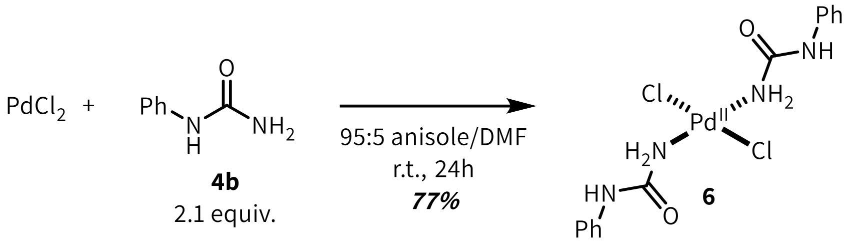

***trans*-κ*N'*-Bis(*N*-phenylurea)palladium(II) chloride (6)**

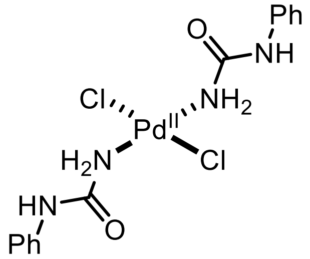

To a solution of urea **4b** (162 mg, 1.187 mmol, 2.1 equiv.) in 95:5 anisole/DMF (10 mL, 0.1M) in 10-dram vial was added $PdCl_2$ (100 mg, 0.565 mmol, 1.0 equiv.). The resulting brown suspension was stirred under air for 24 h until it turned into a bright yellow suspension. Upon completion of the reaction, stirring was ceased, the suspension was allowed to settle for 3 h, then the clear top layer was syringed away, leaving behind concentrated suspension (*ca.* 5 mL). To this suspension 2:1 anisole/toluene (30 mL) was added. The suspension was stirred for 30 min, then the stirring was ceased, the suspension was allowed to settle for 3 h, and the clear top layer was syringed away. Toluene (30 mL) was added to the remaining suspension, the suspension was stirred for 30 min, then the stirring was ceased, the suspension was let to settle for 3 h, and the clear top layer was syringed away. The remaining solvents were removed under reduced pressure affording complex **6** as a yellow solid (196 mg, 0.435 mmol, 77%). When the identical procedure was followed with only one equiv. of **4b** (77 mg, 0.565 mmol, 1.0 equiv.), 95:5 anisole/DMF (10 mL, 0.1M), and $PdCl_2$ (100 mg, 0.565 mmol, 1.0 equiv.) complex **6** was still obtained but in a lower yield as a yellow solid (59 mg, 0.130 mmol, 23%).

Anal. calculated for $C_{14}H_{16}Cl_2N_4O_2Pd$ (FW = 449.63), C 37.40, H 3.59, N 12.46; found C 37.18, H 3.36, N 12.27.

IR (cm^{-1}): 3283, 3210, 3148, 1701, 1601, 1539, 1501, 1443, 1335, 1323, 1304, 1281, 1238, 1165, 1130, 1076, 980, 910, 864, 752, 694, 621, 610.

Raman (cm^{-1}): 1706, 1603, 1550, 1449, 1336, 1324, 1307, 1285, 1240, 1182, 1158, 1122, 1028, 1002, 986, 860, 777, 731, 618, 573, 509, 431, 410, 375, 287, 207, 159, 131.

Comparison of IR spectra of urea 4b and complex 6

Table S4. Assignment of the relevant bands in the IR spectra (units = cm^{-1}).[33]

	**4b**	**6**	Shift
$\nu_{asym}(NH_2)$	3442 (s)	3210 (s)	–232
ν(N-H)	3314 (s)	3278 (m)	–36
$\nu_{sym}(NH_2)$	3217 (sh)	3152 (sh)	–65
ν(C=O)	1654 (s)	1701 (s)	+47
δ(N-H)	1547 (s)	1539 (s)	–8
ρ(NH_2)	1115 (w)	984 (m)	–131

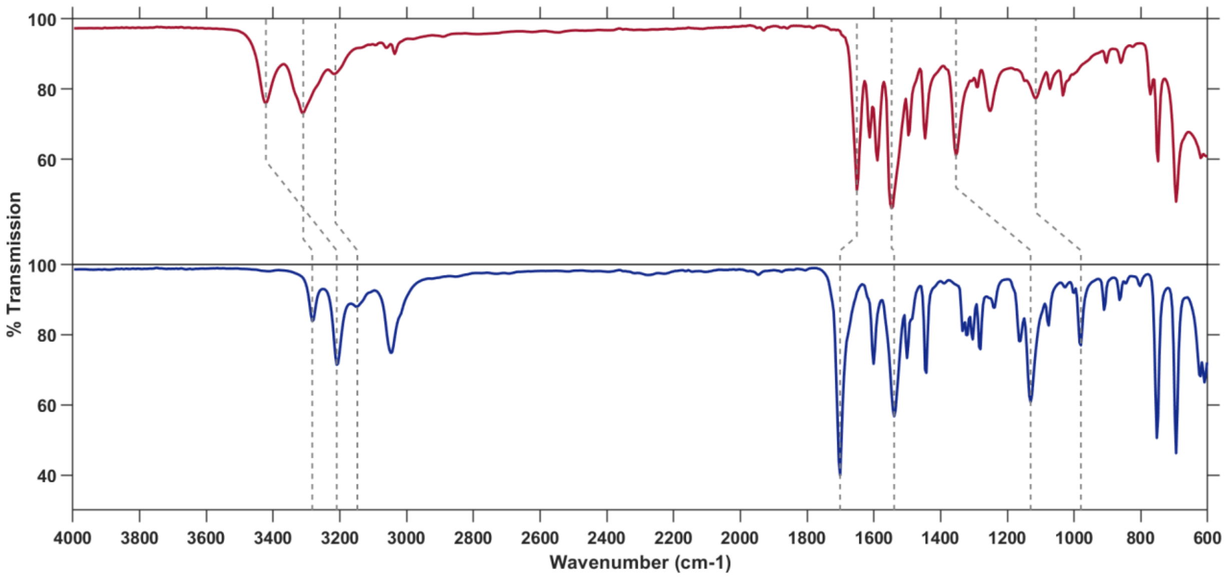

Figure S1. Infrared spectra of urea **4b** (top) and complex **6** (bottom).

Raman Spectroscopy

Sample for Raman spectroscopy was prepared by drop casting suspension of complex **6** (20 mg) in acetone/DCM/EtOAc (1:1:1, 2mL) onto a sapphire substrate (Esco Optics, 19.05 mm diameter, 1 mm thickness) at 50 °C. Raman spectra were aquired under 594 nm CW excitation (Cobolt diode laser) with an incident power of 3.2 mW in a backscattering geometry using a triple subtractive TriVista spectrometer paired with Princeton Instruments Pixis 400 CCD. Thee spectra were averaged to produce the final spectrum, each collected with 2 min integration time. The relative intensities were corrected for detector and grating efficiencies. The relative wavenumbers were calibrated in reference to cyclohexane.

Based on selection rules, in a *cis*-$PdCl_2$ complex (C_{2v} symmetry) two bands corresponding to ν_{sym}(Cl-Pd-Cl) and ν_{asym}(Cl-Pd-Cl) are both expected to be Raman active, while only the symmetric stretch is expected to be Raman active in the *trans*-complex (D_{2h} symmetry). Previously, bands corresponding to ν(Cl-Pd-Cl) have been reported around 310 cm^{-1} in coordination complexes of $PdCl_2$. In the Raman spectrum of complex **6** a single band is observed in the 250-350 cm^{-1} region at 287 cm^{-1} which supports assignment of **6** as the *trans*-complex. For similar assignment of geometry of $PdCl_2$ complexes, see: Navarro-Ranninger, M. C.; Camazon, M. J.; Alvarez-Valdes, A.; Masaguer, J. R.; Martinez-Carrera, S.; Garcia-Blanco, S. Reactions of palladium(II) chloride with 1,4-diphenyl-2,3-dimethyl-1,4-diazabutadiene and 1,4-di(*p*-methoxyphenyl)-2,3-dimethyl-1,4– diazabutadiene. *Polyhedron* **1987**, *6*, 1059-1064.

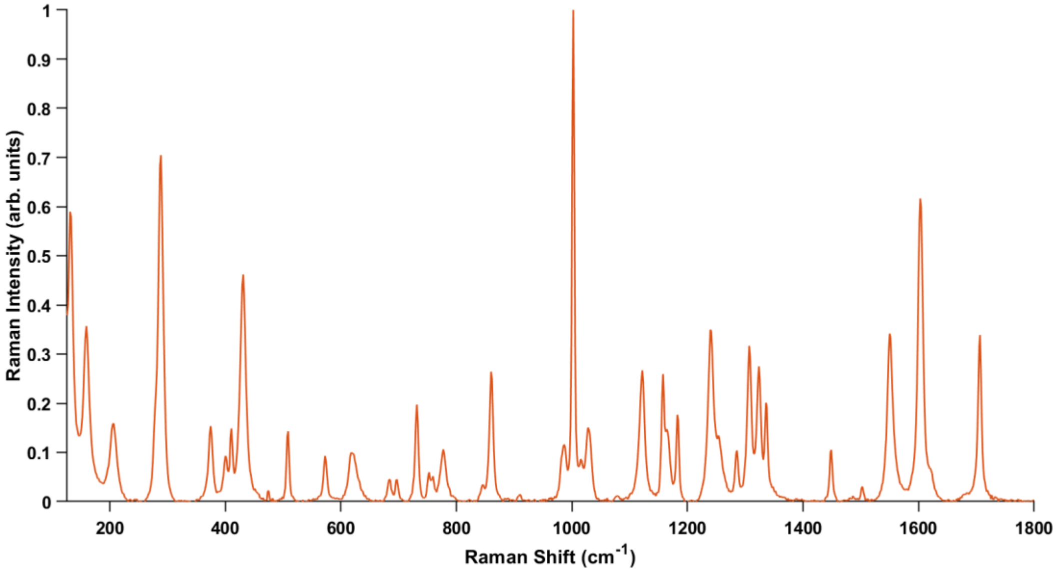

Figure S2. Raman spectrum of complex **6**.

Deterium Exchange ^{1}H NMR Experiment

Both NMR samples were prepared from the same 2:1 DMA/acetone-d_6 stock solution. Proton resonances are referenced to the DMA peak at 2.077 ppm. The top spectrum is of urea **4b** (15 mg, 0.11 mmol) in 2:1 DMA/acetone-d_6 (0.8 mL, 0.14M). The bottom spectrum is of complex **6** (25 mg, 0.056 mmol) in 2:1 DMA/acetone-d_6 (0.8 mL, 0.07M) – urea **4b** appears to be readily displaced from complex **6** in this solvent mixture. We ascribe the disappearance of the –NH_2 peak (5.88 ppm) in the bottom spectrum to rapid deuterium exchange affected by increased acidity of the –NH_2 protons upon coordination to Pd. No exchange at –NH (8.70 ppm) was observed.

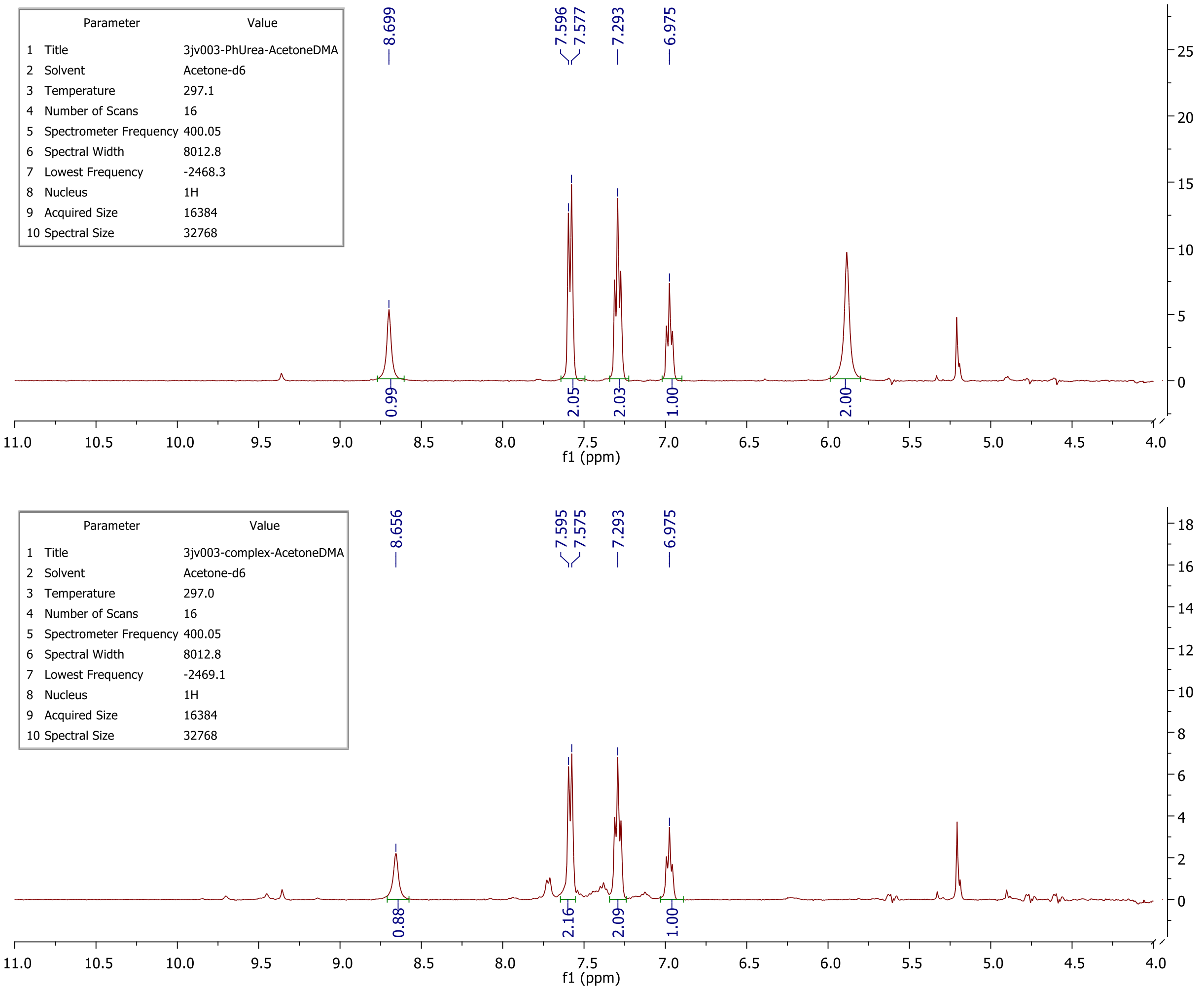

Figure S3. Close-up of ^{1}H NMR spectra of urea **4b** (top) and complex **6** (bottom) in 2:1 DMA/acetone-*d*6.

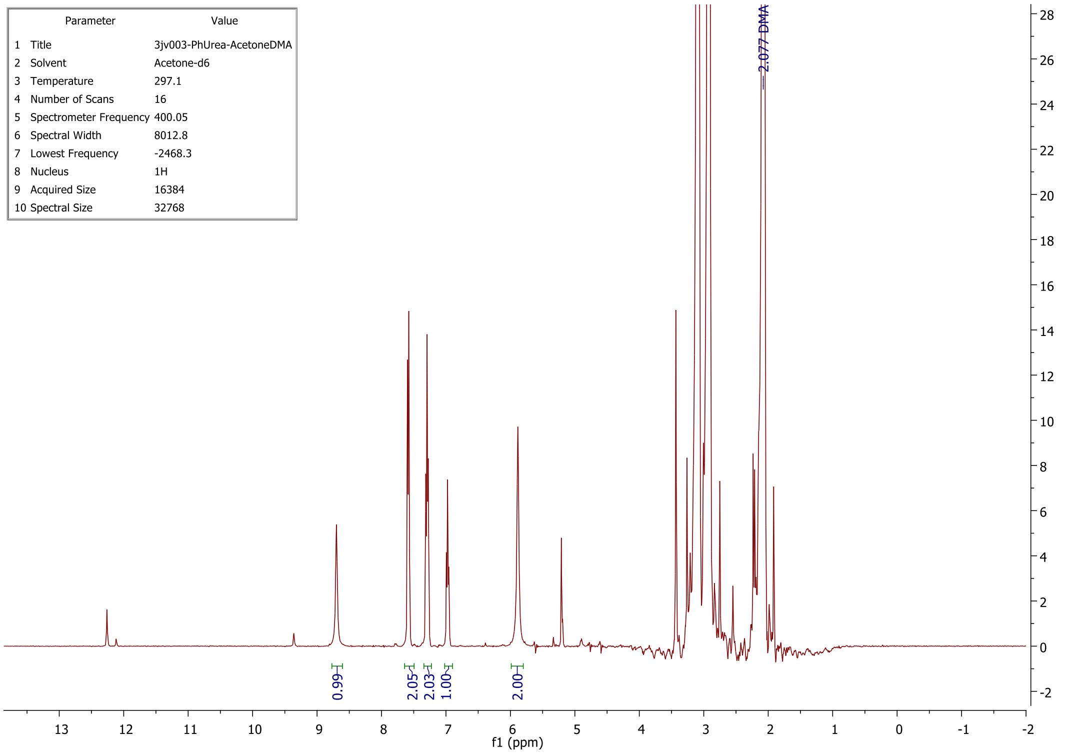

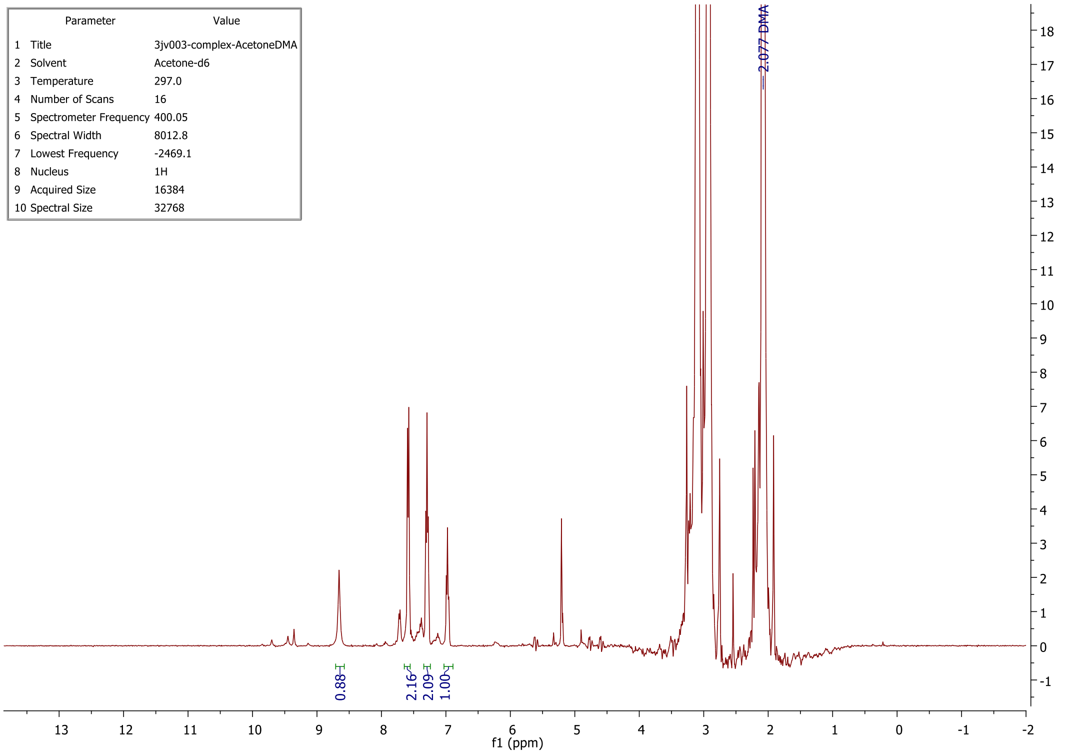

Figure S4. ^{1}H NMR spectra of urea **4b** (top) and complex **6** (bottom) in 2:1 DMA/acetone-*d*6.

Competence of Complex 6 as Precatalyst

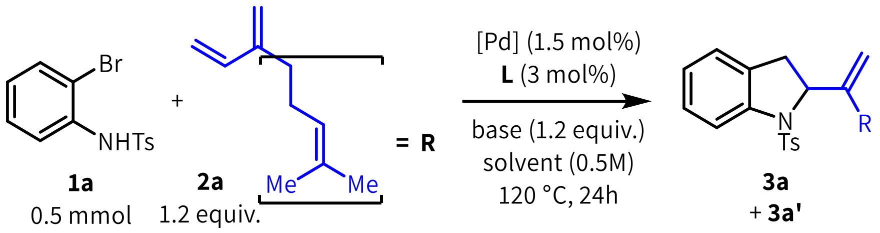

Table S5. Comparison of competence of complex **6** as precatalyst.

Entry	Pd precatalyst	Additional ligand	**3a** + **3a'**	r.r.
1	$PdCl_2$	No ligand	26%	91:9
2	$PdCl_2$	Urea **4b**	45%	91:1
3	**6**	No ligand	56%	90:10

General procedure for complex 6 precatalyst competence studies: Bromoaniline **1a** (163 mg, 0.500 mmol, 1.0 equiv.), myrcene **2a** (103 uL, 0.600 mmol, 1.2 equiv.), urea **4b** (2.0 mg, 0.015 mmol, 0.03 equiv.), Pd precatalyst (0.0075 mmol, 0.015 equiv.), and 1,3,5-trimethoxybenze (internal standard, 40 mg, 0.238 mmol, 0.48 equiv.) were weighed out in the above-mentioned order into a 1-dram vial equipped with a stir bar and a cap with a silicone septum. The vial was then placed under nitrogen atmosphere and charged with 1 mL of freshly degassed solvent mixture (0.5M). An aliquot for HPLC analysis was taken. Then, base (0.600 mmol, 1.2 equiv.) was added to the reaction mixture. The reaction mixture was degassed with nitrogen and the reaction was stirred at 120 °C for 24 hours. After cooling to room temperature, the yield of **3a** + **3a'** and the regioisomeric ratio were determined by HPLC analysis of the crude reaction mixture.

Entry 1: General procedure was followed. **1a** (163 mg, 0.500 mmol, 1.0 equiv.), **2a** (103 uL, 0.600 mmol, 1.2 equiv.), K_2CO_3 (83 mg, 0.600 mmol, 1.2 equiv.), $PdCl_2$ (1.3 mg, 0.0075 mmol, 0.015 equiv.), 1,3,5-trimethoxybenze (40 mg, 0.238 mmol, 0.48 equiv.) and 95:5 anisole/DMF (1.0 mL, 0.5M) were used. The yield of **3a** + **3a'** and the regioisomeric ratio were determined by HPLC analysis of the crude reaction mixture.

Run 1: 25%
Run 2: 27%
Average: 26% yield (91:9 r.r.)

Entry 2: General procedure was followed. **1a** (163 mg, 0.500 mmol, 1.0 equiv.), **2a** (103 uL, 0.600 mmol, 1.2 equiv.), K_2CO_3 (83 mg, 0.600 mmol, 1.2 equiv.), urea **4b** (2.0 mg, 0.015 mmol, 0.03 equiv.), $PdCl_2$ (1.3 mg, 0.0075 mmol, 0.015 equiv.), 1,3,5-trimethoxybenze (40 mg, 0.238 mmol, 0.48 equiv.) and 95:5 anisole/DMF (1.0 mL, 0.5M) were used. The yield of **3a** + **3a'** and the regioisomeric ratio were determined by HPLC analysis of the crude reaction mixture.

Run 1: 46%
Run 2: 43%
Average: 45% yield (91:9 r.r.)

Entry 3: General procedure was followed. **1a** (163 mg, 0.500 mmol, 1.0 equiv.), **2a** (103 uL, 0.600 mmol, 1.2 equiv.), K_2CO_3 (83 mg, 0.600 mmol, 1.2 equiv.), complex **6** (3.4 mg, 0.0075 mmol, 0.015 equiv.), 1,3,5-trimethoxybenze (40 mg, 0.238 mmol, 0.48 equiv.) and 95:5 anisole/DMF (1.0 mL, 0.5M) were used. The yield of **3a** + **3a'** and the regioisomeric ratio were determined by HPLC analysis of the crude reaction mixture.

Run 1: 57%
Run 2: 55%
Average: 56% yield (90:10 r.r.)

^{13}C NMR urea-Pd binding studies

Four NMR samples of ^{13}C-urea were prepared to investigate urea-Pd binding under neutral and basic conditions. Carbon resonances are referenced for samples A/B and C/D to the DCM peak at 53.86 ppm or to the acetone-d_6 peak at 29.84 ppm, respectively.

Sample A: ^{13}C-urea (0.020 g, 0.328 mmol) was dissolved in 0.5 mL of D_2O/DCM (100:1).

Sample B: ^{13}C-urea (0.020 g, 0.328 mmol, 2.1 eq.) and K_2PdCl_4 (0.051 g, 0.156 mmol, 1.0 eq.) was dissolved in 0.5 mL of D_2O/DCM (100:1) and stirred for 1 h.

Sample C: ^{13}C-urea (0.010 g, 0.164 mmol, 2.1 eq.) and $PdCl_2$ (0.014 g, 0.078 mmol, 1.0 eq.) were stirred in 1.0 mL of acetone-d6 for 12 h resulting in yellow suspension. Then, 2.0 mL DMF was added to this mixture and it was stirred for additional 2 h.

Sample D: ^{13}C-urea (0.010 g, 0.164 mmol, 2.1 eq.), $PdCl_2$ (0.014 g, 0.078 mmol, 1.0 eq.) and K_2CO_3 was stirred in 1.0 mL of acetone-d6 for 12 h resulting in yellow suspension. Then, 2.0 mL DMF was added to this mixture and it was stirred for additional 2 h.

Sample	^{13}C shift (ppm)[a]	Assignment/comments
A	162.7	Urea (data agree with that reported in the literature)[34]
B	168.7	Pd-(κ*O*-urea)
	168.2	
	159.8	Pd-(κ*N*-urea)
C	157.4	Pd-(κ*N*-urea)
	156.9	
D	175.7	Likely Pd-(κ*N*-carbamate) complexes based on literature precedent
	169.0	Pd-(κ*N*-ureate) based on DFT calculations

[a] Solvent peaks are omitted and so is urea peak in samples **B-D**.

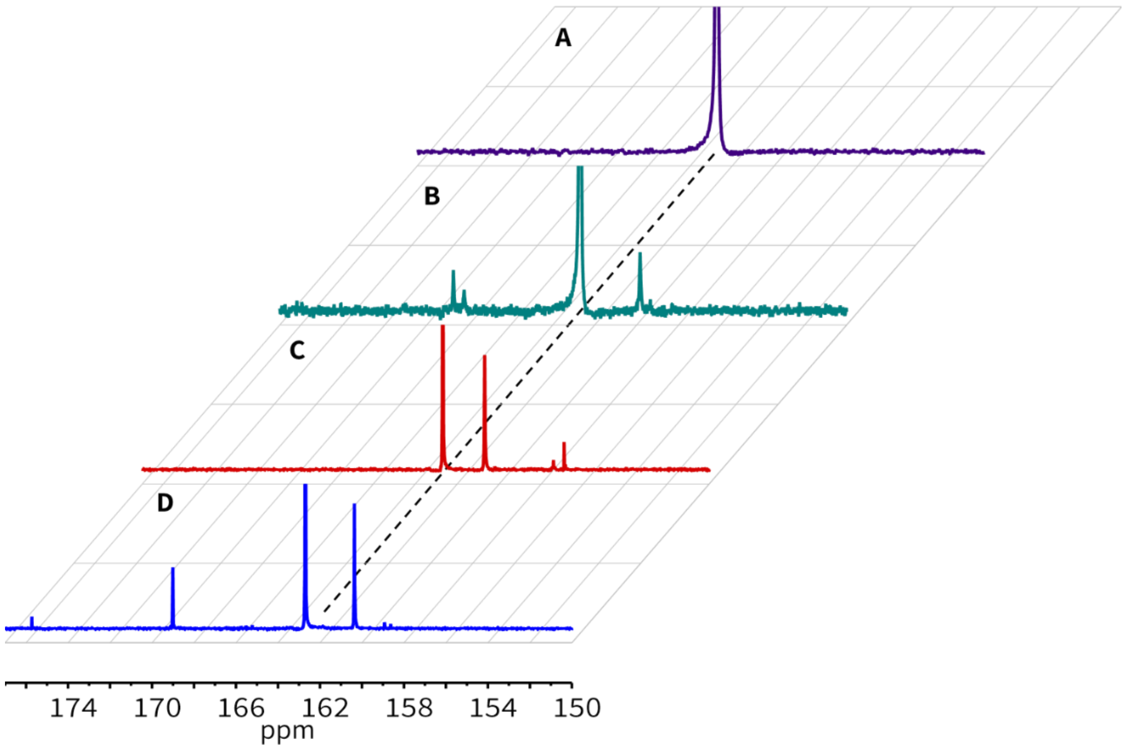

Figure S5. ^{13}C NMR spectra of samples **A-D.**

Computational studies

Density functional theory (DFT) calculations were performed with Gaussian 16 package using University of Rochester's BlueHive computer cluster.[35] Geometry optimizations and frequency calculations were performed at the SMD(acetone)-MN15-def2TZVPP computational level unless otherwise indicated.[36] This computational level was benchmarked on a prior solution-state study of urea binding of urea to Pd(en)(H2O)$^{2+}$ cation. NMR shielding tensors were calculated using Gauge-Independent Atomic Orbital (GIAO) method at the same computational level.[37] Harmonic analysis was used to establish the nature of all optimized structures as minima (0 imaginary frequencies). When the substrates showed conformational freedom, conformational analysis was performed and only the most stable conformer of each stationary point was considered and reported, unless otherwise noted. The Percent Buried Volume (%V_{bur}) calculations were performed using the SambVca 2.1 web application.[38]

Computational level benchmarking

Kosctic 1997

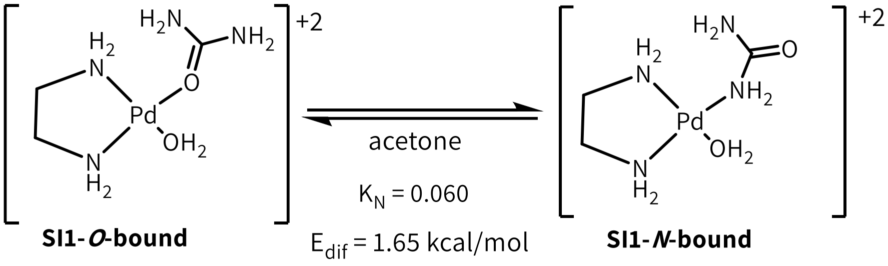

SI1-*O*-bound **SI1-*N*-bound**

The goal of our calculations was to compare relative energies of different urea-Pd or ureate-Pd binding modes. Therefore, we decided to benchmark the functional, basis set, and solvation model on a prior solution-state study of urea binding of urea to Pd(en)(H2O)$^{2+}$ in which urea binding through both O and N was observed, with slight preference for the former (1.6 kcal/mol). Best agreement of our calculations with literature data was achieved with SMD(acetone)-MN15-def2TZVPP level of theory. The def2TZVPP was chosen over ccpVTZPP due to lower computational requirements.

Table S6. Results of benchmarking of the functional, basis set, and solvation model on a prior solution-state study of urea binding of urea to Pd(en)(H2O)$^{2+}$

Functional	Basis Set	Solvation Model	E_{diff} (kcal/mol)
MN15	def2TZVPP	SMD(acetone)	2.50
MN15	LanL2TZp	SMD(acetone)	2.62
MN15	ccpVTZPP	SMD(acetone)	2.50
M06	LanL2TZp	SMD(acetone)	3.01
B3LYP	LanL2TZp	SMD(acetone)	3.82
O3LYP	LanL2TZp	SMD(acetone)	3.97
MN15	def2TZVPP	CPCM(acetone)	5.03
MN15	def2TZVPP	IEFPCM(acetone)	5.77

SI1-*O*-Bound (SMD(acetone)-MN15-def2TZVPP)

		Hartree
Sum of electronic and zero-point Energies	=	-619.157520
Sum of electronic and thermal Energies	=	-619.143812
Sum of electronic and thermal Enthalpies	=	-619.142867
Sum of electronic and thermal Free Energies	=	-619.197832
Lowest energy vibration	=	44.76 cm^{-1}

2 1							
Pd	-0.317354	0.409200	-0.098647	H	-2.565533	-1.099000	1.510806
N	-0.743268	-1.529938	-0.357839	H	-3.931932	-0.774470	0.426151
H	-0.242873	-1.917350	-1.159002	C	2.500045	-0.415896	0.082096
N	-2.290450	0.574452	0.286705	N	3.727083	-0.540557	-0.420817
H	-2.734498	1.004236	-0.527781	H	4.462810	-1.009033	0.088510
O	0.097383	2.418297	0.200030	H	3.933951	-0.137052	-1.322772
H	-0.530732	3.069447	-0.151023	N	2.217988	-0.981334	1.258930
O	1.630540	0.231084	-0.584696	H	2.930418	-1.463588	1.789847
C	-2.210926	-1.678918	-0.522244	H	1.329506	-0.794668	1.704451
H	-2.466735	-1.346423	-1.527336	H	0.979166	2.648283	-0.136874
H	-2.506944	-2.719140	-0.401840	H	-0.427650	-2.044351	0.467480
C	-2.846892	-0.783788	0.506828	H	-2.486882	1.178093	1.085548

SI1-*N*-Bound (SMD(acetone)-MN15-def2TZVPP)

		Hartree
Sum of electronic and zero-point Energies	=	-619.154619
Sum of electronic and thermal Energies	=	-619.142154
Sum of electronic and thermal Enthalpies	=	-619.141209
Sum of electronic and thermal Free Energies	=	-619.193844
Lowest energy vibration	=	42.27 cm^{-1}

2 1							
Pd	0.262829	0.240641	-0.171336	H	3.305298	0.112128	-0.924070
N	1.284417	-1.480066	-0.308359	H	4.005713	0.233590	0.702083
H	0.741637	-2.278511	0.022814	C	-2.574103	-0.390478	0.092380
N	2.026424	0.986104	0.480650	N	-3.014966	-1.469471	0.723269
H	1.963617	1.099933	1.494423	H	-2.684894	-2.399390	0.502174
O	-0.730308	2.043908	-0.048665	H	-3.720435	-1.364002	1.440930
H	-0.436296	2.662568	0.636924	N	-1.524059	-0.610762	-0.863675
O	-2.966428	0.756920	0.285529	H	-1.402947	-1.597288	-1.099252
C	2.552139	-1.348484	0.453038	H	-1.736101	-0.103686	-1.726663

H	2.312626	-1.429580	1.512462	H	-1.685181	1.808490	0.110235
H	3.246388	-2.140726	0.180536	H	1.484139	-1.643325	-1.297776
C	3.098228	0.018498	0.141275	H	2.229894	1.905849	0.088357

Comparison of urea-Pd binding modes

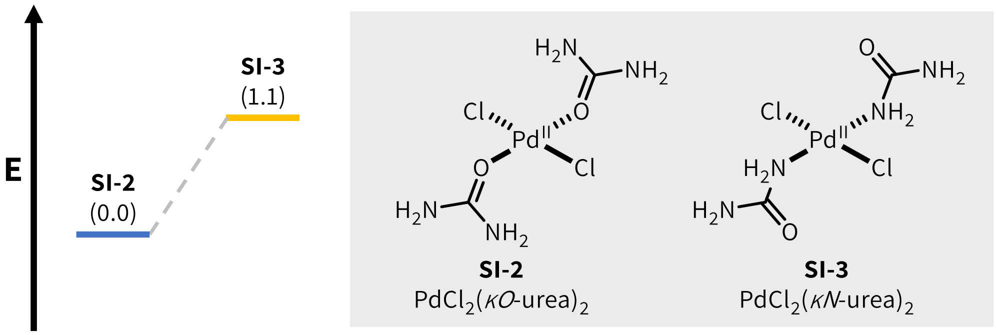

Figure S6. Comparison of urea-Pd binding modes (values in parentheses are Gibbs free energies in kcal mol^{-1}). Ground state energy calculations were carried out at the SMD(acetone)-MN15-def2TZVPP level of theory.

Urea (SMD(acetone)-MN15-def2TZVPP)

		Hartree
Sum of electronic and zero-point Energies	=	-225.076452
Sum of electronic and thermal Energies	=	-225.071695
Sum of electronic and thermal Enthalpies	=	-225.070751
Sum of electronic and thermal Free Energies	=	-225.102991
Lowest energy vibration	=	300.64 cm^{-1}

0 1							
C	-0.000022	0.137164	0.000019	H	1.136228	-1.582311	0.172642
N	-1.150044	-0.598176	0.050867	O	-0.000214	1.364977	-0.000039
H	-1.135746	-1.582410	-0.173507	H	2.015362	-0.102787	0.104922
N	1.150206	-0.597831	-0.050638	H	-2.015133	-0.103247	-0.105465

SI-2 [$PdCl_2$(*κO*-urea)$_2$] (SMD(acetone)-MN15-def2TZVPP)

		Hartree
Sum of electronic and zero-point Energies	=	-1498.194525
Sum of electronic and thermal Energies	=	-1498.178572
Sum of electronic and thermal Enthalpies	=	-1498.177628
Sum of electronic and thermal Free Energies	=	-1498.239597
Lowest energy vibration	=	35.88 cm^{-1}

0 1							
Pd	-0.000011	-0.000246	-0.000087	H	3.704749	0.642861	-1.703529
Cl	-0.318928	-2.261094	-0.275789	N	-4.077083	0.429162	-0.545364
Cl	0.319150	2.260512	0.275633	H	-4.110505	0.867658	-1.453214
C	-2.887716	0.119413	-0.014240	H	-4.938579	0.258335	-0.048802
C	2.887615	-0.118799	0.014442	N	4.077232	-0.426080	0.546479
N	-2.852494	-0.404646	1.216934	H	4.110817	-0.865081	1.454081
H	-3.705231	-0.642430	1.703462	H	4.938513	-0.257006	0.048940
H	-1.977927	-0.781046	1.558846	O	1.844130	-0.349624	0.688279
N	2.852164	0.404041	-1.217228	O	-1.844180	0.349402	-0.688300
H	1.977148	0.778687	-1.559941				

SI-3 [$PdCl_2$(***κ****N*-urea)$_2$] (SMD(acetone)-MN15-def2TZVPP)

		Hartree
Sum of electronic and zero-point Energies	=	-1498.194424
Sum of electronic and thermal Energies	=	-1498.179978
Sum of electronic and thermal Enthalpies	=	-1498.179033
Sum of electronic and thermal Free Energies	=	-1498.238018
Lowest energy vibration	=	30.13 cm^{-1}

0 1							
Pd	0.000000	0.000538	-0.000145	O	-3.577780	1.119533	-0.126551
Cl	0.080459	2.293697	-0.153461	N	-3.144206	-1.093258	-0.477790
Cl	-0.079991	-2.292563	0.153399	H	-3.884337	-1.201660	-1.156477
N	1.804908	-0.131654	-0.978000	H	-2.473936	-1.843476	-0.350656
H	1.841736	0.649918	-1.636333	N	3.144185	1.090200	0.480937
N	-1.804986	0.132815	0.977632	H	2.474362	1.841070	0.355302
H	-1.841014	-0.647339	1.637674	H	3.884081	1.196626	1.160198
C	-2.934304	0.111328	0.068446	H	-1.825359	1.013501	1.493427
C	2.933999	-0.112942	-0.068435	H	1.824691	-1.011230	-1.495692
O	3.577075	-1.121841	0.124145				

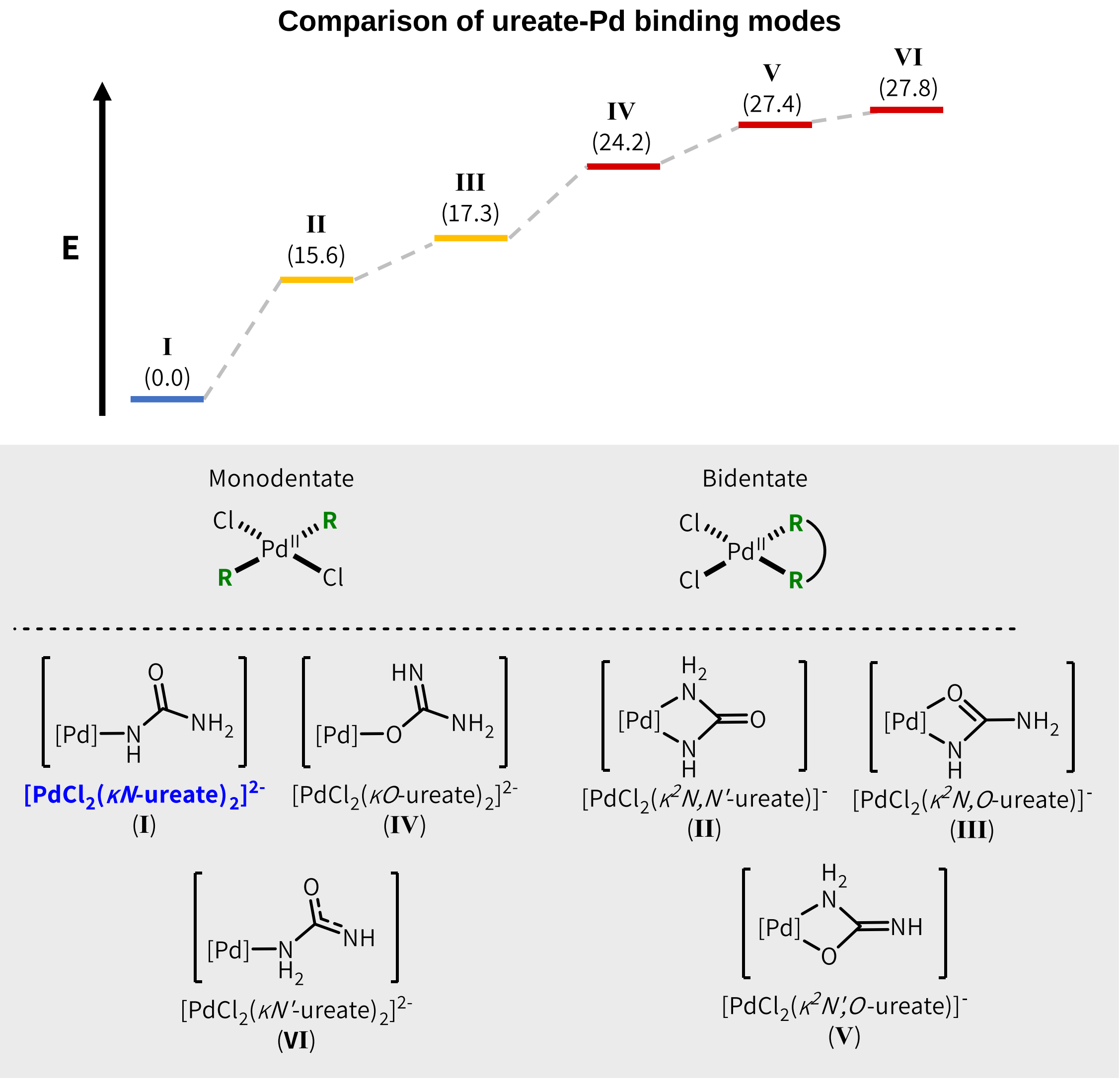

Figure S7. Comparison of ureate-Pd binding modes (values in parentheses are Gibbs free energies in kcal mol^{-1}). Ground state energy calculations were carried out at the SMD(acetone)-MN15-def2TZVPP level of theory. Sums of energies of bidentate complexes plus free ureate are compared to monodentate complexes.

***N*-Ureate$^-$** (SMD(acetone)-MN15-def2TZVPP)

		Hartree
Sum of electronic and zero-point Energies	=	-224.574877
Sum of electronic and thermal Energies	=	-224.570867
Sum of electronic and thermal Enthalpies	=	-224.569923
Sum of electronic and thermal Free Energies	=	-224.600886
Lowest energy vibration	=	380.16 cm^{-1}

0 1							
C	-0.111022	0.044989	-0.009022	O	-0.365025	1.277065	0.007185

N	-0.951781	-0.978513	0.008646	H	1.870290	0.391955	0.294635
N	1.258063	-0.314694	-0.090649	H	-1.891347	-0.581365	0.025082
H	1.463417	-1.244590	0.250959				

(**I**) $[PdCl_2(\boldsymbol{\kappa}N\text{-ureate})_2]^{2-}$ (SMD(acetone)-MN15-def2TZVPP)

		Hartree
Sum of electronic and zero-point Energies	=	-1497.262130
Sum of electronic and thermal Energies	=	-1497.247993
Sum of electronic and thermal Enthalpies	=	-1497.247048
Sum of electronic and thermal Free Energies	=	-1497.305813
Lowest energy vibration	=	22.19 cm^{-1}

-2 1							
Pd	-0.000079	0.000008	-0.000218	N	-3.119917	0.839770	0.818376
Cl	0.401294	-2.270920	0.334723	H	-4.042859	1.238123	0.921548
Cl	-0.401322	2.270968	-0.335194	H	-2.368520	1.514787	0.716905
N	1.866477	0.539227	0.560707	N	3.120535	-0.839774	-0.817834
N	-1.866694	-0.539111	-0.561106	H	2.369117	-1.514821	-0.716622
C	-3.073485	-0.234869	-0.061892	H	4.043529	-1.238183	-0.920319
C	3.073577	0.234843	0.062479	H	-1.911652	-1.425870	-1.051591
O	4.121970	0.858952	0.319755	H	1.911195	1.425792	1.051539
O	-4.121976	-0.859158	-0.318248				

(**II**) $[PdCl_2(\boldsymbol{\kappa}N,N'\text{-ureate})]^{-}$ (SMD(acetone)-MN15-def2TZVPP)

		Hartree
Sum of electronic and zero-point Energies	=	-1272.644333
Sum of electronic and thermal Energies	=	-1272.635634
Sum of electronic and thermal Enthalpies	=	-1272.634689
Sum of electronic and thermal Free Energies	=	-1272.680127
Lowest energy vibration	=	55.65 cm^{-1}

-1 1							
C	-2.367211	-0.092036	0.000032	O	-3.583418	-0.039669	-0.000424
N	-1.484593	-1.072302	0.000646	H	-1.683070	1.704804	-0.825160
H	-1.782057	-2.040022	-0.000238	Pd	0.191770	-0.005120	0.000025

N	-1.513439	1.131589	0.000015	Cl	1.739596	-1.706375	-0.000148
H	-1.682299	1.704256	0.825747	Cl	1.800573	1.666435	-0.000025

(**III**) $[PdCl_2$(***κ****N*,*O*-ureate)$]^-$ (SMD(acetone)-MN15-def2TZVPP)

		Hartree
Sum of electronic and zero-point Energies	=	-1272.640981
Sum of electronic and thermal Energies	=	-1272.631571
Sum of electronic and thermal Enthalpies	=	-1272.630627
Sum of electronic and thermal Free Energies	=	-1272.677329
Lowest energy vibration	=	70.48 cm^{-1}

-1 1							
C	2.213445	0.028743	0.035595	O	1.540158	1.121565	0.136334
N	1.477727	-1.064108	0.192959	H	3.997633	0.941208	-0.342833
H	1.811352	-1.949591	-0.165775	Pd	-0.211266	0.001202	0.044314
N	3.533043	0.055989	-0.208248	Cl	-1.751061	-1.678761	-0.071636
H	4.060094	-0.798769	-0.303503	Cl	-1.827067	1.658980	-0.070926

(**IV**) $[PdCl_2$(***κ****O*-ureate$)_2]^{2-}$ (SMD(acetone)-MN15-def2TZVPP)

		Hartree
Sum of electronic and zero-point Energies	=	-1497.223341
Sum of electronic and thermal Energies	=	-1497.208858
Sum of electronic and thermal Enthalpies	=	-1497.207914
Sum of electronic and thermal Free Energies	=	-1497.268109
Lowest energy vibration	=	20.61cm^{-1}

-2 1							
Pd	-0.000014	0.000112	0.000044	H	4.077850	0.413090	-1.474080
Cl	0.014512	2.316358	0.053184	H	4.875108	0.262797	0.017969
Cl	-0.014524	-2.316123	-0.053027	N	-2.855365	0.119391	-1.391965
C	-2.864668	0.032825	-0.094553	H	-1.884654	0.173211	-1.701309
C	2.864706	-0.033132	0.094414	N	2.855557	-0.120139	1.391795
N	-4.093265	0.014187	0.558441	H	1.884866	-0.173884	1.701218
H	-4.875023	-0.263400	-0.018343	O	1.859316	0.010880	-0.721832
H	-4.077978	-0.413220	1.473872	O	-1.859380	-0.010661	0.721855

N	4.093213	-0.014534	-0.558751				

(**V**) [$PdCl_2$(***κ****N’*,*O*-ureate)]$^{-}$ (SMD(acetone)-MN15-def2TZVPP)

		Hartree
Sum of electronic and zero-point Energies	=	-1272.625272
Sum of electronic and thermal Energies	=	-1272.616606
Sum of electronic and thermal Enthalpies	=	-1272.615661
Sum of electronic and thermal Free Energies	=	-1272.661290
Lowest energy vibration	=	41.89 cm^{-1}

-1 1							
C	2.315538	0.080942	-0.000429	Cl	-1.741330	1.697081	-0.000115
N	1.490513	-1.137470	0.000515	Cl	-1.793204	-1.631548	-0.000279
H	1.653560	-1.708580	0.827916	N	3.582750	-0.004862	-0.000582
H	1.653522	-1.709741	-0.826079	O	1.517130	1.098253	0.000289
Pd	-0.190477	0.002206	0.000133	H	3.998812	0.927412	-0.000534

(**VI**) [$PdCl_2$(***κ****N’*-ureate)$_2$]$^{2-}$ (SMD(acetone)-MN15-def2TZVPP)

		Hartree
Sum of electronic and zero-point Energies	=	-1497.216721
Sum of electronic and thermal Energies	=	-1497.203111
Sum of electronic and thermal Enthalpies	=	-1497.202166
Sum of electronic and thermal Free Energies	=	-1497.259104
Lowest energy vibration	=	49.44 cm^{-1}

-2 1							
Pd	-0.000039	-0.000098	0.000106	O	-3.864301	-0.460117	0.487506
Cl	0.619088	2.221331	0.167120	O	3.864709	0.459378	-0.488346
Cl	-0.619012	-2.221448	-0.167237	N	-1.798626	0.491711	0.846145
C	-3.006212	0.298306	0.002038	H	-1.694412	1.482386	1.068036
C	3.006308	-0.298129	-0.002087	N	1.798344	-0.491434	-0.846002
N	-2.960159	0.987432	-1.108440	H	1.694893	-1.481997	-1.068814
H	-3.830503	0.813847	-1.611380	H	-1.941713	-0.027176	1.710429
N	2.959968	-0.986375	1.108907	H	1.941111	0.028143	-1.709932
H	3.830592	-0.813210	1.611509				

NMR shielding tensor calculations using Gauge-Independent Atomic Orbital (GIAO) method

NMR shielding tensors were calculated using Gauge-Independent Atomic Orbital (GIAO) method at SMD(acetone)-MN15-def2TZVPP level of theory for urea and selected complexes in Figures S6 and S7. The calculated ^{13}C shift of urea was first referenced to the TMS B3LYP/6-311+G(2d,p) GIAO standard and then adjusted to its experimental value (162.7 ppm) and used as a reference for the remaining calculations.

Compound	^{13}C shift (ppm)	
	Calculated	Adjusted
**Urea**	7.899	162.7
**SI-2** $[PdCl_2(\kappa O\text{-urea})_2]$	2.910	167.7
**SI-3** $[PdCl_2(\kappa N\text{-urea})_2]$	11.832	158.8
(**I**) $[PdCl_2(\kappa N\text{-ureate})_2]^{2-}$	-4.862	175.5
(**IV**) $[PdCl_2(\kappa O\text{-ureate})_2]^{2-}$	-5.171	175.8

Percent Buried Volume (%Vbur) calculations

The Percent Buried Volume (%V_{bur}) calculations were performed using the SambVca 2.1 web application.[38] The recommended parameters were used (i.e. Bondii radii scaled by 1.17, sphere radius = 3.5, mesh spacing for numerical integration = 0.10, H atoms not included). %V_{bur} was calculated for $[PdCl_2(\kappa N\text{-ureate})_2]^{2-}$ (**I**), $[PdCl_2(\kappa O\text{-ureate})_2]^{2-}$ (**IV**) and a model *N'*-arylureate complex **SI-4**. For the purposes of contrasting the %V_{bur} differences (ureate vs phosphine ligands) in the Figure 1, %V_{bur} was also calculated for literature complexes **SI-5** and **SI-6** with PMe_3 and P^tBu_3 as ligands, respectively.[39]

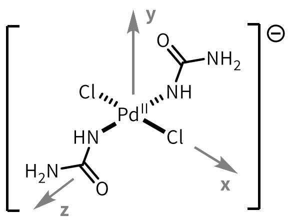

(**I**)
$[PdCl_2(\kappa N\text{-ureate})_2]^{2-}$
%V_{bur} = 18.0

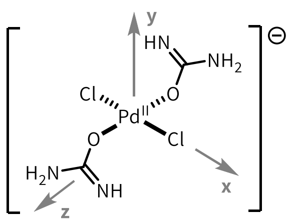

(**IV**)
$[PdCl_2(\kappa O\text{-ureate})_2]^{2-}$
%V_{bur} = 18.8

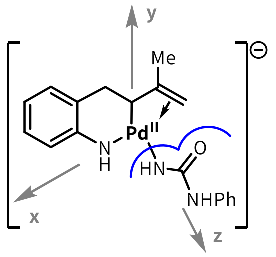

SI-4
%V_{bur} = 17.2

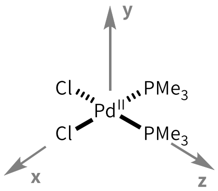

SI-5
%V_{bur} = 23.5

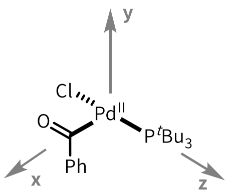

SI-6
%V_{bur} = 37.8

(**I**) $[PdCl_2(\kappa N\text{-ureate})_2]^{2-}$ (SMD(acetone)-MN15-def2TZVPP)

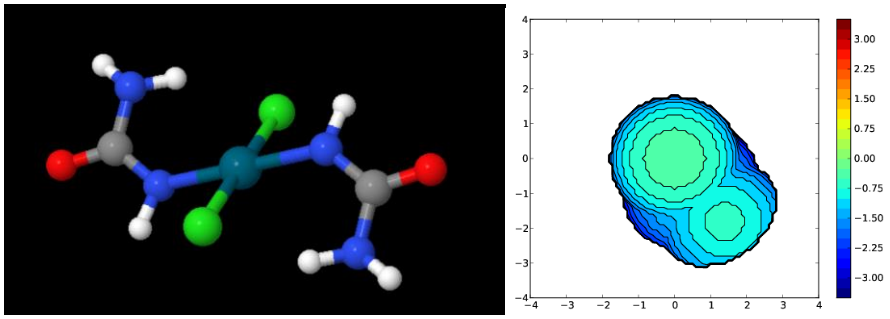

(**IV**) $[PdCl_2(\kappa O\text{-ureate})_2]^{2-}$ (SMD(acetone)-MN15-def2TZVPP)

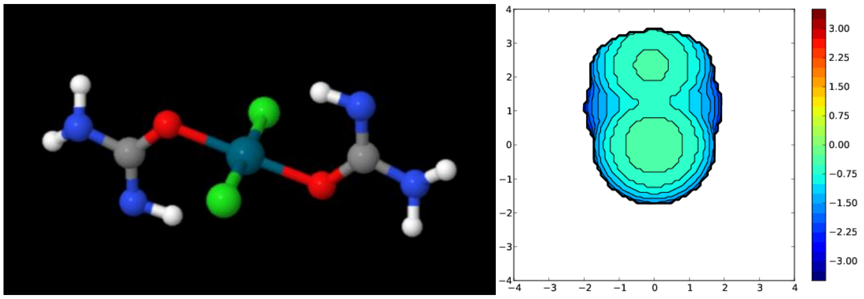

SI-4 (SMD(acetone)-MN15-def2SVP)

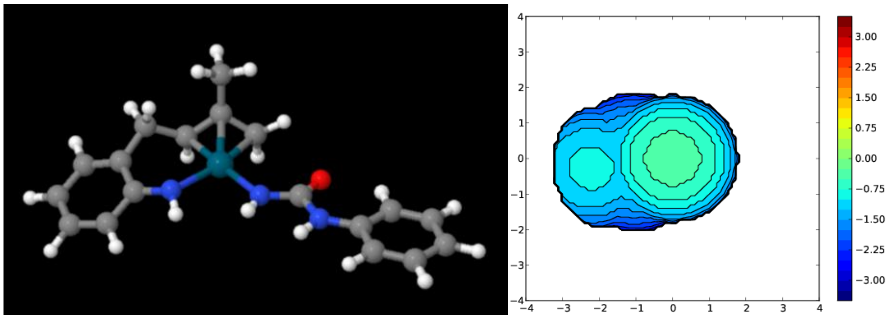

SI-5[39a]

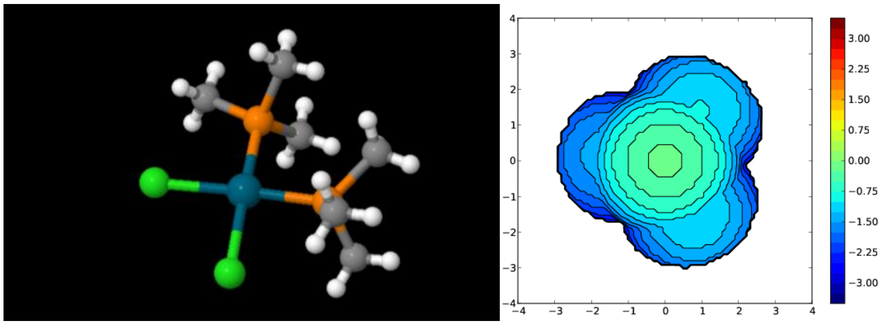

SI-6[39b]

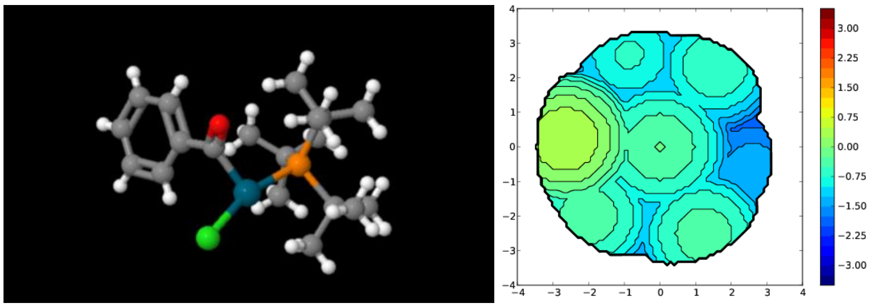

SI-4 (SMD(acetone)-MN15-def2SVP)

		Hartree
Sum of electronic and zero-point Energies	=	-1062.924190
Sum of electronic and thermal Energies	=	-1062.902002
Sum of electronic and thermal Enthalpies	=	-1062.901058
Sum of electronic and thermal Free Energies	=	-1062.978600
Lowest energy vibration	=	11.70 cm^{-1}

-1 1							
C	-5.095268	-2.924075	-0.301331	H	-0.564438	3.407453	1.656912
C	-3.756830	-2.685833	-0.010910	Pd	-0.843256	0.510741	-0.053404
C	-3.228979	-1.375239	0.179332	C	2.196758	0.022605	-0.105243
C	-4.178689	-0.298895	0.089287	O	2.483207	1.112669	-0.601791
C	-5.511741	-0.570021	-0.210001	N	0.971158	-0.444370	0.161414
C	-6.001163	-1.866403	-0.416319	N	3.249155	-0.885565	0.241378
H	-5.432766	-3.955614	-0.447515	N	-1.918126	-1.167381	0.454267
H	-3.058926	-3.527290	0.065965	H	-1.405880	-2.049244	0.483493
H	-6.200309	0.281156	-0.273296	H	0.961663	-1.363357	0.601176
H	-7.052958	-2.039716	-0.655218	H	2.957471	-1.772161	0.638924
C	-0.365814	2.455057	-0.784214	C	4.617231	-0.737093	0.105022
H	-0.410091	2.275439	-1.864164	C	5.241395	0.411252	-0.429738
H	0.535883	2.955946	-0.426065	C	5.446609	-1.799475	0.527558
C	-1.556468	2.484718	-0.013179	C	6.630399	0.467936	-0.525673
C	-2.572774	1.569421	-0.439627	H	4.604548	1.230733	-0.756522
H	-2.702721	1.439199	-1.524335	C	6.832703	-1.725446	0.424143

C	-3.747428	1.120591	0.392969	H	4.979428	-2.697823	0.944795
H	-3.465606	1.185325	1.459832	C	7.445097	-0.587452	-0.105237
H	-4.622999	1.781622	0.251338	H	7.087278	1.369610	-0.944086
C	-1.587380	3.191308	1.317483	H	7.438808	-2.570538	0.762910
H	-2.129676	4.147986	1.228500	H	8.532540	-0.524510	-0.188513
H	-2.088417	2.593854	2.091435				

Characterization of $Pd(OAc)_2$ precatalyst

Palladium acetate was purchased from Strem (USA); batch with lot number L01322101. The palladium acetate was predominantly in its trimeric form, i.e., $Pd_3(OAc)_6$. The content of insolubles and thus the percentage of the polymeric form $[Pd(OAc)_2]_n$ was <0.2%, as per the certificate of analysis (see below). The amount of the nitro analogue impurity, i.e. $Pd_3(OAc)_5(NO_2)$, was quantified using ^{1}H NMR spectroscopy and 1,4-dimethoxybenzene as internal standard as recommended by Colacot et al.[40] The following sample was prepared in non-dry $CDCl_3$: 1,4-diemthoxybenzene (0.0228 g, 0.165 mmol), $Pd(OAc)_2$ (0.0203 g), $CDCl_3$ (1 mL). Proton resonances are referenced to the $CDCl_3$ peak at 7.260 ppm. $Pd_3(OAc)_5(NO_2)$ resonances at 2.071 (s, 3H) and 2.061 (s, 3H) ppm were used for the analysis. The concentration of $Pd_3(OAc)_5(NO_2)$ was found to be <2%.

STREM
AN ASCENSUS™ COMPANY

CERTIFICATE OF ANALYSIS

46-1780 Palladium(II) acetate, min. 98% (99.9+%-Pd)

**Lot Number:**	L01322101
**CAS Number:**	3375-31-3

Characteristic	Specification	Result
Color	Golden Brown	Conforms
Form	Crystal	Conforms
Assay	NLT 98%	100%
Palladium	46.50 - 48.40%	47.41%
Metals Purity	NLT 99.9%	99.95%
Insolubles	NMT 2%	<0.2%

This document has been electronically generated and does not require a signature.

January 14, 2021

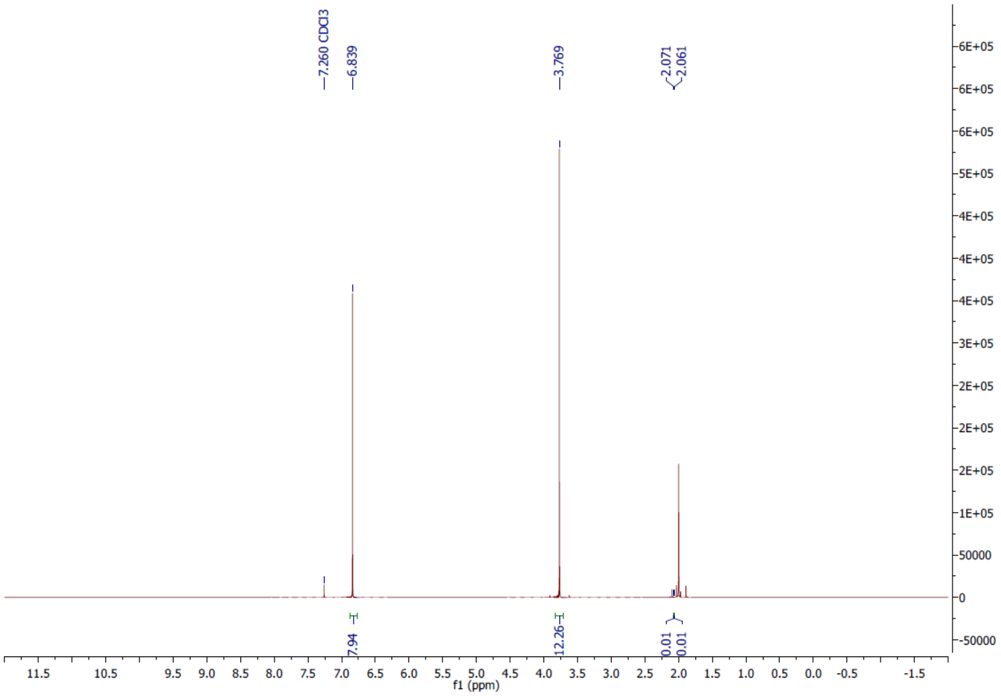

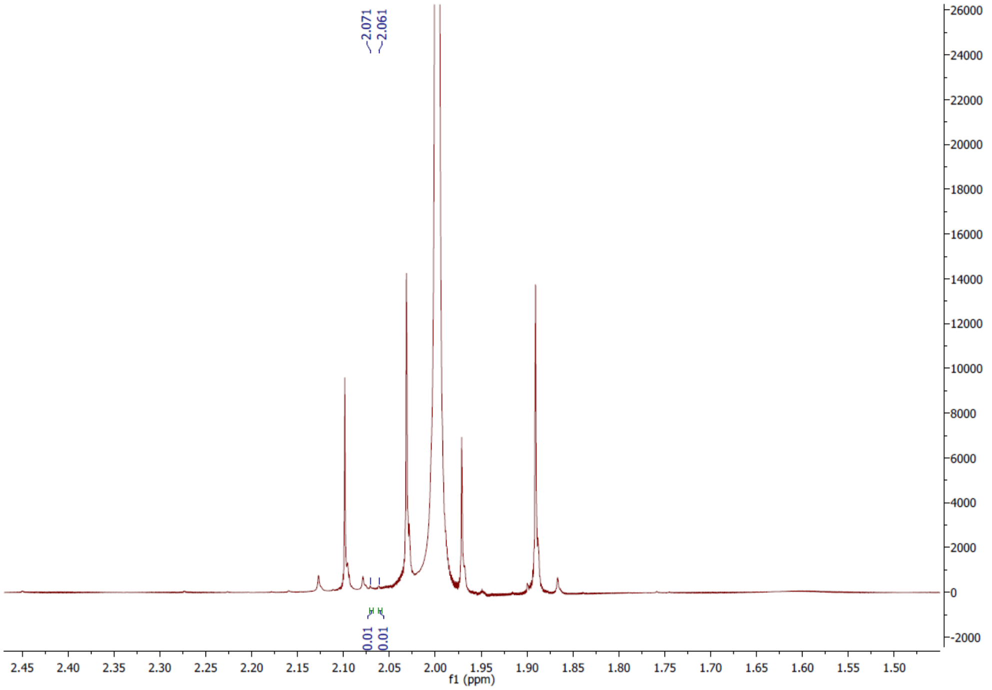

Figure S8. ^{1}H NMR spectrum of $Pd(OAc)_2$ in $CDCl_3$.

References Cited

(1) Youn, S. W.; Kim, Y. H.; Jo, J. H. Palladium-Catalyzed Regioselective Synthesis of 1-Hydroxycarbazoles Under Aerobic Conditions. *Adv. Synth. Catal.* **2019**, *361*, 462-468.

(2) Marcyk, P. T.; Jefferies, L. R.; AbuSalim, D. I.; Pink, M.; Baik, M.-H.; Cook, S. P. Stereoinversion of unactivated alcohols by tethered sulfonamides. *Angew. Chem. Int. Ed.* **2019**, *58*, 1727-1731.

(3) Song, H.; Liu, Y.; Liu, Y.; Wang, Q. Self-Induced Stereoselective in Situ Trifluoromethylation: Preparation of Spiro[indoline-3,3′-quinoline] via Palladium-Catalyzed Cascade Reaction. *Org. Lett.* **2014**, *16*, 3240−3243.
(4) Lv, J.; Liu, Q.; Tang, J.; Perdih, F.; Kranjc, K. A facile synthesis of indolo[3,2,1-jk]carbazoles via palladium-catalyzed intramolecular cyclization. *Tet. Lett.* **2012**, *53*, 5248-5252.
(5) Sartorius, F.; Trebing, M.; Brückner, C.; Brückner, R. Reducing Diastereomorphous Bis(phosphaneoxide) Atropisomers to One Atropisomerically Pure Diphosphane: A New Ligand and a Novel Ligand-Preparation Design. *Chem. - Eur. J.* **2017**, *23*, 17463−17468.
(6) Schroder, N.; Wencel-Delord, J.; Glorius, F. High-yielding, versatile, and practical [Rh(III)Cp*]-catalyzed ortho bromination and iodination of arenes. *J. Am. Chem. Soc.* **2012**, *134*, 8298−8301.
(7) Caddick, S.; Judd, D. B.; Lewis, A. K. K.; Reich, M. T.; Williams, M. R. V. A Generic Approach for the Catalytic Reduction of Nitriles. *Tetrahedron* **2003**, *59*, 5417−5423.
(8) Sun, R. F.; Wang, Z. W.; Li, Y. Q.; Xiong, L. X.; Liu, Y. X.; Wang, Q. M. Design, Synthesis, and Insecticidal Evaluation of New Benzoylureas Containing Amide and Sulfonate Groups Based on the Sulfonylurea Receptor Protein Binding Site for Diflubenzuron and Glibenclamide. *J. Agric. Food Chem.* **2013**, *61*, 517-522.
(9) Yasukawa, N.; Yokoyama, H.; Masuda, M.; Monguchi, Y.; Sajiki, H.; Sawama, Y. Highly–functionalized arene synthesis based on palladium on carbon–catalyzed aqueous dehydrogenation of cyclohexadienes and cyclohexenes. *Green Chem.* **2018**, *20*, 1213–1217.
(10) Furuta, A.; Hirobe, Y.; Fukuyama, T.; Ryu, I.; Manabe, Y.; Fukase, K. Flow Dehydration and Hydrogenation of Allylic Alcohols: Application to the Waste–Free Synthesis of Pristane. *Eur. J. Org. Chem.* **2017**, *2017*, 1365–1368.
(11) Khan, F. A.; Budanur, B. M. Superoxide mediated isomerization of 4–aryl–but–1–ynes to 1–aryl–1,3–butadienes. *Tetrahedron* **2015**, *71*, 7600–7607.
(12) Ji, D.–W.; He, G.–C.; Zhang, W.–S.; Zhao, C.–Y.; Hu, Y.–C.; Chen, Q.–A. Nickel–catalyzed allyl–allyl coupling reactions between 1,3–dienes and allylboronates. *Chem. Commun.* **2020**, *56*, 7431–7434.
(13) Madden, K. S.; David, S.; Knowles, J. P.; Whiting, A. Heck–Mizoroki coupling of vinyliodide and applications in the synthesis of dienes and trienes. *Chem. Commun.* **2015**, *51*, 11409–11412.
(14) Cheung, K. P. S.; Kurandina, D.; Yata, T.; Gevorgyan, V. Photoinduced Palladium–Catalyzed Carbofunctionalization of Conjugated Dienes Proceeding via Radical–Polar Crossover Scenario: 1,2–Aminoalkylation and Beyond. *J. Am. Chem. Soc.* **2020**, *142*, 9932–9937.
(15) Wu, J.; Jiang, X.; Xu, J.; Dai, W.–M. Tandem Wittig–intramolecular Diels–Alder cycloaddition of ester–tethered 1,3,9–decatrienes under microwave heating. *Tetrahedron* **2011**, *67*, 179–192.
(16) Adamson, N. J.; Hull, E.; Malcolmson, S. J. Enantioselective Intermolecular Addition of Aliphatic Amines to Acyclic Dienes with a Pd–PHOX Catalyst. *J. Am. Chem. Soc*. **2017**, *139*, 7180–7183.
(17) Stang, E. M.; White, M. C. Molecular Complexity via C–H Activation: A Dehydrogenative Diels–Alder Reaction. *J. Am. Chem. Soc*. **2011**, *133*, 14892–14895.
(18) Hioki, H.; Ooi, H.; Hamano, M.; Mimura, Y.; Yoshio, S.; Kodama, M.; Ohta, S.; Yanai, M.; Ikegami, S. Enantioselective total synthesis and absolute stereostructure of hippospongic acid A. *Tetrahedron* **2001**, *57*, 1235–1246.
(19) Kennedy, C. R.; Zhong, H.; Macaulay, R. L.; Chirik, P. J., Regio– and Diastereoselective Iron–Catalyzed [4+4]–Cycloaddition of 1,3–Dienes. *J. Am. Chem. Soc*. **2019**, *141*, 8557–8573.

(20) Wu, J. Y.; Moreau, B.; Ritter, T. Iron–Catalyzed 1,4–Hydroboration of 1,3–Dienes. *J. Am. Chem. Soc*. **2009,** *131*, 12915–12917.
(21) Zitzelberger, T. J.; Schiavelli, M. D.; Thompson, D. W. A facile and selective methylation of 5–en–3–yn–1–ols with titanium tetrachloride–trimethylaluminum yielding (3Z)–4–methylalka–3,5–dien–1–ols. *J. Org. Chem.* **1983,** *48*, 4781–4783.
(22) Syntrivanis, L.–D.; Robertson, J., Synthesis of (E)–4–Methylhexa–3,5–dien–1–ol and Its Diels–Alder Reaction with Thioester Dienophiles: A Short Enantioselective Synthesis of Bicyclic Lactones. *Eur. J. Org. Chem.* **2017,** *2017*, 4916–4921.
(23) Lee, R. J.; Lindley, M. R.; Pritchard, G. J.; Kimber, M. C. A biosynthetically inspired route to substituted furans using the Appel reaction: total synthesis of the furan fatty acid F5. *Chem. Commun.* **2017,** *53*, 6327–6330.
(24) Duvvuri, K.; Dewese, K. R.; Parsutkar, M. M.; Jing, S. M.; Mehta, M. M.; Gallucci, J. C.; RajanBabu, T. V. Cationic Co(I)–Intermediates for Hydrofunctionalization Reactions: Regio– and Enantioselective Cobalt–Catalyzed 1,2–Hydroboration of 1,3–Dienes. *J. Am. Chem. Soc*. **2019,** *141*, 7365–7375.
(25) Rovira, A. R.; Fin, A.; Tor, Y. Chemical Mutagenesis of an Emissive RNA Alphabet *J. Am. Chem. Soc.* **2015**, *137*, 4602−14605.
(26) Basu, P.; Dey, T.K.; Ghosh, A.; Biswas, S.; Khan, A.; Islam, S.M. An Efficient One-Pot Synthesis of Industrially Valuable Primary Organic Carbamates and *N*-Substituted Ureas by a Reusable Merrifield Anchored Iron(II)-Anthra Catalyst [Fe^{II}(Anthra-Merf)] Using Urea as a Sustainable Carbonylation Source. *New J. Chem.* **2020**, *44*, 2630–2643.
(27) Gan, Z.; Li, G.; Yan, Q.; Deng, W.; Jiang, Y.-Y.; Yang, D. Visible-light-promoted oxidative desulphurisation: a strategy for the preparation of unsymmetrical ureas from isothiocyanates and amines using molecular oxygen. *Green Chem.* **2020**, *22*, 2956−2962.
(28) Kawamorita, S.; Miyazaki, T.; Iwai, T.; Ohmiya, H.; Sawamura, M. Rh-catalyzed Borylation of N-adjacent C(sp^3)-H Bonds with a Silica-Supported Triarylphosphine Ligand. *J. Am. Chem. Soc.* **2012**, *134*, 12924−12927.
(29) Breitler, S.; Oldenhuis, N. J.; Fors, B. P.; Buchwald, S. L. Synthesis of Unsymmetrical Diarylureas via Pd-Catalyzed C–N Cross-Coupling Reactions. *Org. Lett.* **2011**, *13*, 3262-3265.
(30) Habibi, D.; Heydari, S.; Faraji, A.; Keypour, H.; Mahmoudabadi, M. A green and facile approach for the synthesis of N-monosubstituted ureas in water: Pd catalyzed reaction of arylcyanamides (an unexpected behavior of electron withdrawing groups). *Polyhedron* **2018**, *151*, 520−529.
(31) Babu, S. S.; Shahid, M.; Gopinath, P. Dual palladium–photoredox catalyzed chemoselective C–H arylation of phenylureas. *Chem. Commun.* **2020**, *56*, 5985-5988.
(32) Larock, R. C.; Hightower, T. R.; Hasvold, L. A.; Peterson, K. P., Palladium(II)-Catalyzed Cyclization of Olefinic Tosylamides. *J. Org. Chem.* **1996,** *61*, 3584-3585.
(33) (a) Saito, Y.; Machida, K.; Uno, T. Vibrational spectra of methylurea. *Spectrochim. Acta, Part A* **1975**, *31*, 1237–1244. (b) Vrielynck, L.; Lapouge. C.; Marquis, S.; Kister, J.; Dupuy, N. Theoretical and experimental vibrational study of phenylurea: structure, solvent effect and inclusion process with the beta-cyclodextrin in the solid state. *Spectrochim. Acta A Mol. Biomol. Spectrosc.* **2004**, *60,* 2553-2559. (c) Varghese, H. T.; Bhagysree, J. B.; Ulahannan, R. T.; Renjith, R.; Panicker, C. Y. FT-IR, FT-Raman and computational study of phenylurea. *Orient. J. Chem.* **2013**, *29*, 361–367. (d) Penland, R.B.; Mizushima, S.; Curran, C.; Quagliano, J.V. Infrared Absorption Spectra of Inorganic Coordination Complexes. X. Studies of Some Metal-Urea Complexes. *J. Am. Chem. Soc.* **1957**, *79*, 1575-1578.
(34) Kaminskaia, N.V.; Kostić, N.M. Kinetics and Mechanism of Urea Hydrolysis Catalyzed by Palladium(II) Complexes. *Inorg. Chem.* **1997**, *36*, 5917–5926.

(35) Gaussian 16, Revision A.03, Frisch, M. J.; Trucks, G. W.; Schlegel, H. B.; Scuseria, G. E.; Robb, M. A.; Cheeseman, J. R.; Scalmani, G.; Barone, V.; Petersson, G. A.; Nakatsuji, H.; Li, X.; Caricato, M.; Marenich, A. V.; Bloino, J.; Janesko, B. G.; Gomperts, R.; Mennucci, B.; Hratchian, H. P.; Ortiz, J. V.; Izmaylov, A. F.; Sonnenberg, J. L.; Williams-Young, D.; Ding, F.; Lipparini, F.; Egidi, F.; Goings, J.; Peng, B.; Petrone, A.; Hender-son, T.; Ranasinghe, D.; Zakrzewski, V. G.; Gao, J.; Rega, N.; Zheng, G.; Liang, W.; Hada, M.; Ehara, M.; Toyota, K.; Fukuda, R.; Haseg-awa, J.; Ishida, M.; Nakajima, T.; Honda, Y.; Kitao, O.; Nakai, H.; Vreven, T.; Throssell, K.; Montgomery, J. A., Jr.; Peralta, J. E.; Ogliaro, F.; Bearpark, M. J.; Heyd, J. J.; Brothers, E. N.; Kudin, K. N.; Staroverov, V. N.; Keith, T. A.; Kobayashi, R.; Normand, J.; Raghavachari, K.; Rendell, A. P.; Burant, J. C.; Iyengar, S. S.; To-masi, J.; Cossi, M.; Millam, J. M.; Klene, M.; Adamo, C.; Cammi, R.; Ochterski, J. W.; Martin, R. L.; Morokuma, K.; Farkas, O.; For-esman, J. B.; Fox, D. J. Gaussian, Inc., Wallingford CT, 2016.

(36) (a) Yu, H. S.; He, X.; Li, S. L.; Truhlar, D. MN15: A Kohn–Sham global-hybrid exchange–correlation density functional with broad accuracy for multi-reference and single-reference systems and noncovalent interactions. *Chem. Sci.* **2016**, *7*, 5032−5051. (b) Weigend F.; Ahlrichs, R. Balanced basis sets of split valence, triple zeta valence and quadruple zeta valence quality for H to Rn: Design and assessment of accuracy. *Phys. Chem. Chem. Phys.* **2005**, *7*, 3297-3305. (c) Marenich, A.V.; Cramer, C.J.; Truhlar, D.G. Universal solvation model based on solute electron density and a continuum model of the solvent defined by the bulk dielectric constant and atomic surface tensions, *J. Phys. Chem. B* **2009**, *113*, 6378-6396.

(37) (a) London, F. The quantic theory of inter-atomic currents in aromatic combinations. *J. Phys. Radium* **1937**, *8*, 397–409. (b) McWeeny, R. Perturbation Theory for Fock-Dirac Density Matrix. *Phys. Rev.* **1962**, *126*, 1028-1034. (c) Ditchfield, R. Self-consistent perturbation theory of diamagnetism. 1. Gauge-invariant LCAO method for N.M.R. chemical shifts. *J. Chem. Phys*. **1972**, *56*, 5688–5691. (d) Wolinski, K.; Hinton, J. F.; Pulay, P. Efficient Implementation of the Gauge-Independent Atomic Orbital Method for NMR Chemical Shift Calculations. *J. Am. Chem. Soc*. **1990**, *112*, 8251–8260. (e) Cheeseman, J.R.; Trucks, G.W.; Keith, T.A.; Frisch, M.J. A Comparison of Models for Calculating Nuclear Magnetic Resonance Shielding Tensors. *J. Chem. Phys*. **1996**, *104*, 5497-5509.

(38) Falivene, L.; Cao, Z.; Petta, A.; Serra, L.; Poater, A.; Oliva, R.; Scarano, V.; Cavallo, L. Towards the online computer-aided design of catalytic pockets. *Nat. Chem.* **2019**, *11*, 872–879.

(39) (a) Schultz, G.; Subbotina, N. Yu.; Jensen, C. M.; Golen, J. A.; Hargittai, I. Gas phase and crystal molecular structures of *cis*-$PdCl_2[P(CH_3)_3]_2$. *Inorg. Chim. Acta* **1992**, *191*, 85−90. (b) Quesnel, J.S.; Arndtsen, B.A. A Palladium-Catalyzed Carbonylation Approach to Acid Chloride Synthesis. *J. Am. Chem. Soc.* **2013**, *135*, 45, 16841–16844.

(40) Carole, W. A.; Colacot, T. J. Understanding Palladium Acetate from a User Perspective. *Chem. - Eur. J.* **2016**, *22*, 7686−7695.

NMR Spectra of New Compounds

N-(2-Bromophenyl-4-(trifluoromethoxy)phenyl)-4-methylbenzenesulfonamide (1f)

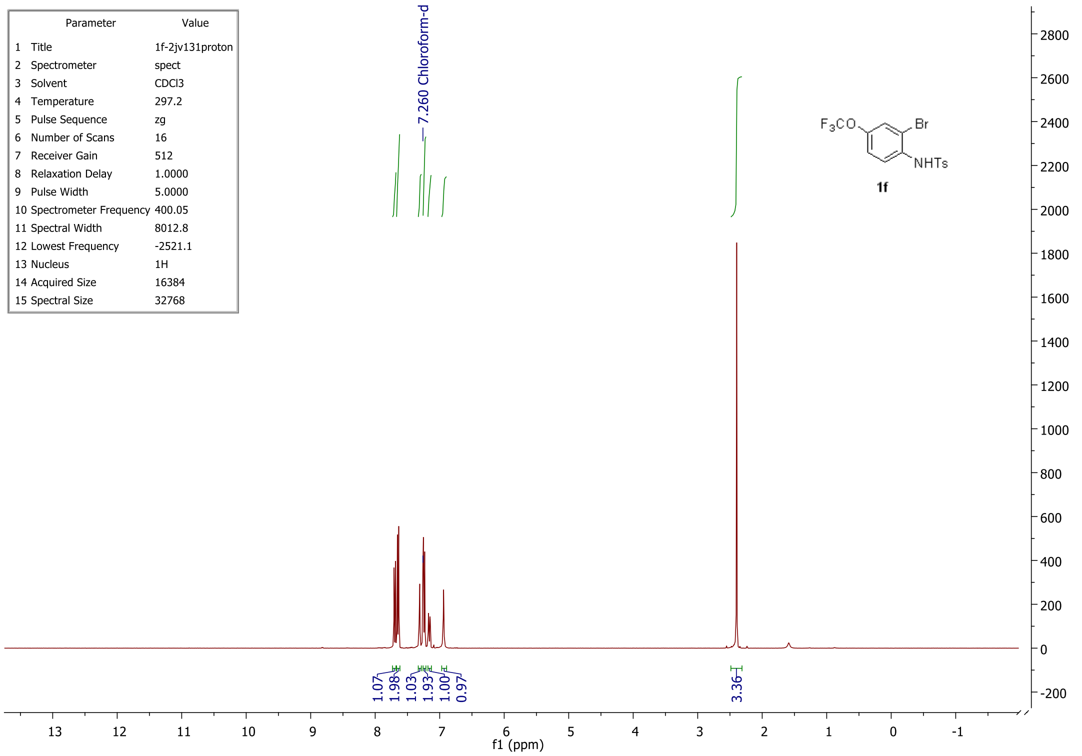

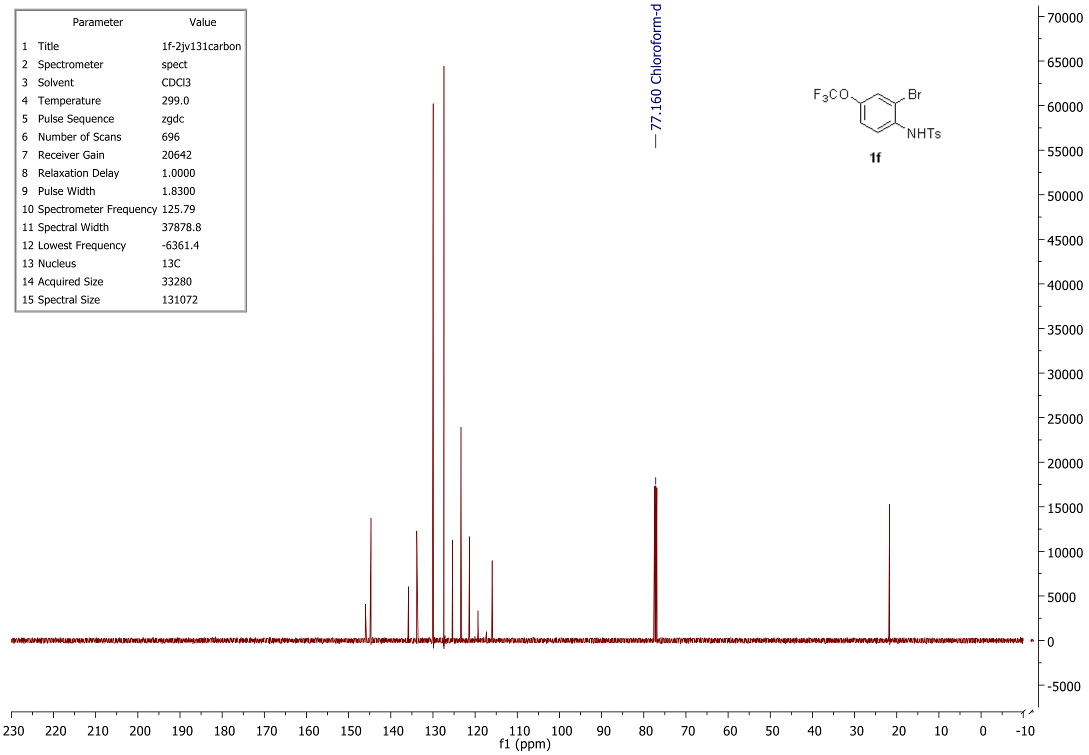

N-(2-Bromophenyl-5-methylphenyl)-4-methylbenzenesulfonamide (1h)

	Parameter	Value
1	Title	1h-2jv137bproton
2	Spectrometer	spect
3	Solvent	CDCl3
4	Temperature	297.1
5	Pulse Sequence	zg
6	Number of Scans	16
7	Receiver Gain	90
8	Relaxation Delay	1.0000
9	Pulse Width	2.2500
10	Spectrometer Frequency	500.21
11	Spectral Width	10080.6
12	Lowest Frequency	-2913.5
13	Nucleus	1H
14	Acquired Size	16384
15	Spectral Size	32768

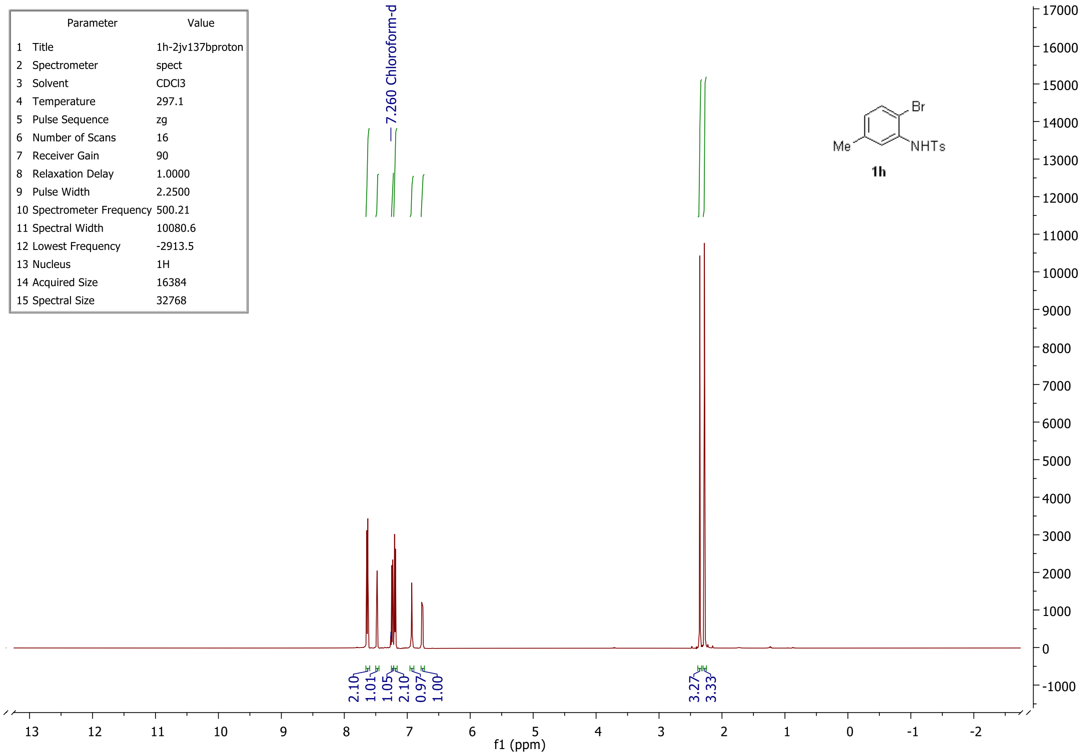

	Parameter	Value
1	Title	1h-2jv137A-C
2	Spectrometer	spect
3	Solvent	CDCl3
4	Temperature	294.6
5	Pulse Sequence	zgdc
6	Number of Scans	58
7	Receiver Gain	32768
8	Relaxation Delay	2.0000
9	Pulse Width	2.0000
10	Spectrometer Frequency	100.56
11	Spectral Width	30303.0
12	Lowest Frequency	-5085.3
13	Nucleus	13C
14	Acquired Size	32768
15	Spectral Size	65536

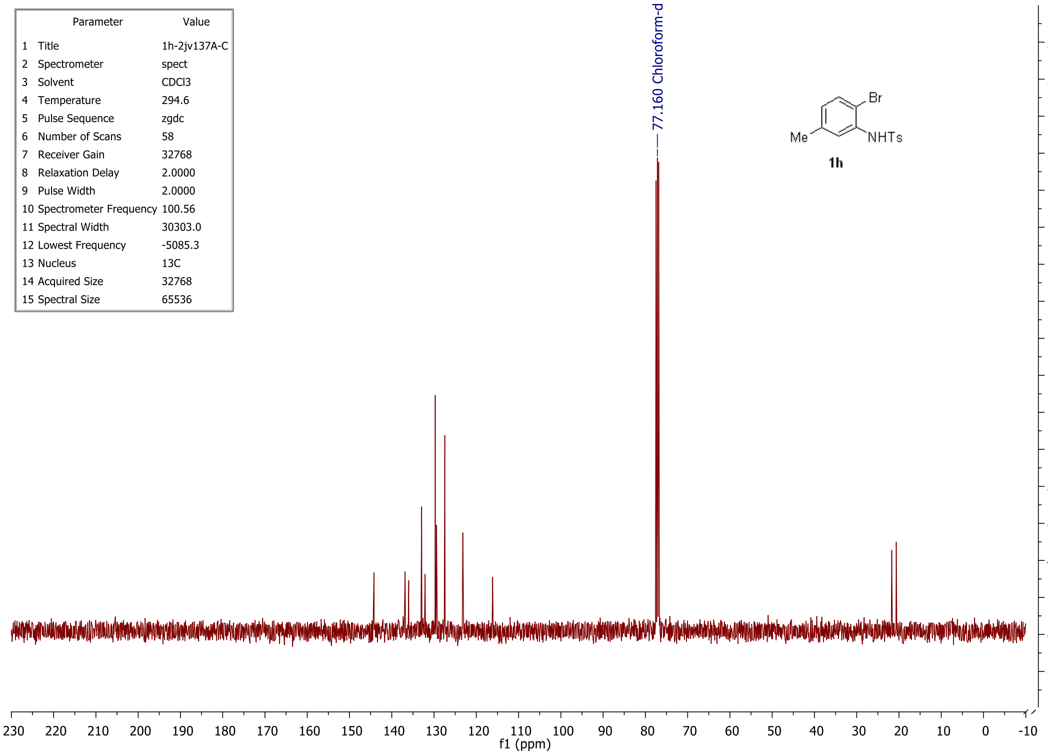

N-(2-Bromophenyl-5-chlorophenyl)-4-methylbenzenesulfonamide (1i)

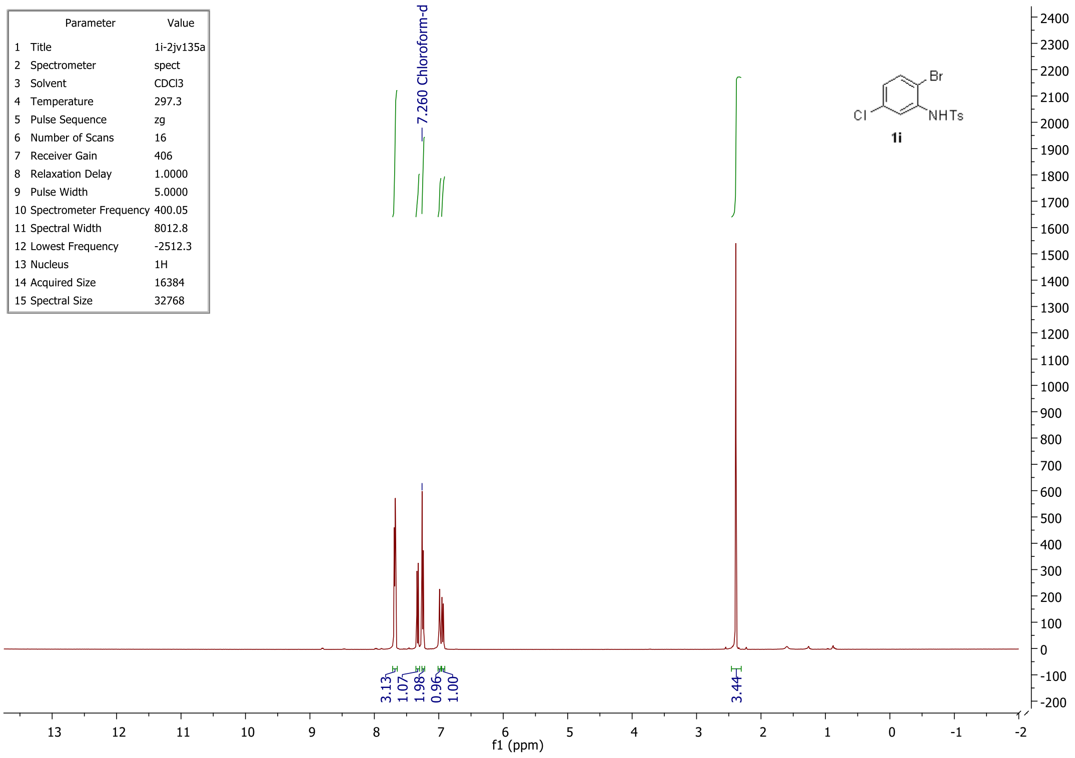

	Parameter	Value
1	Title	1i-2jv135a
2	Spectrometer	spect
3	Solvent	CDCl3
4	Temperature	297.3
5	Pulse Sequence	zg
6	Number of Scans	16
7	Receiver Gain	406
8	Relaxation Delay	1.0000
9	Pulse Width	5.0000
10	Spectrometer Frequency	400.05
11	Spectral Width	8012.8
12	Lowest Frequency	-2512.3
13	Nucleus	1H
14	Acquired Size	16384
15	Spectral Size	32768

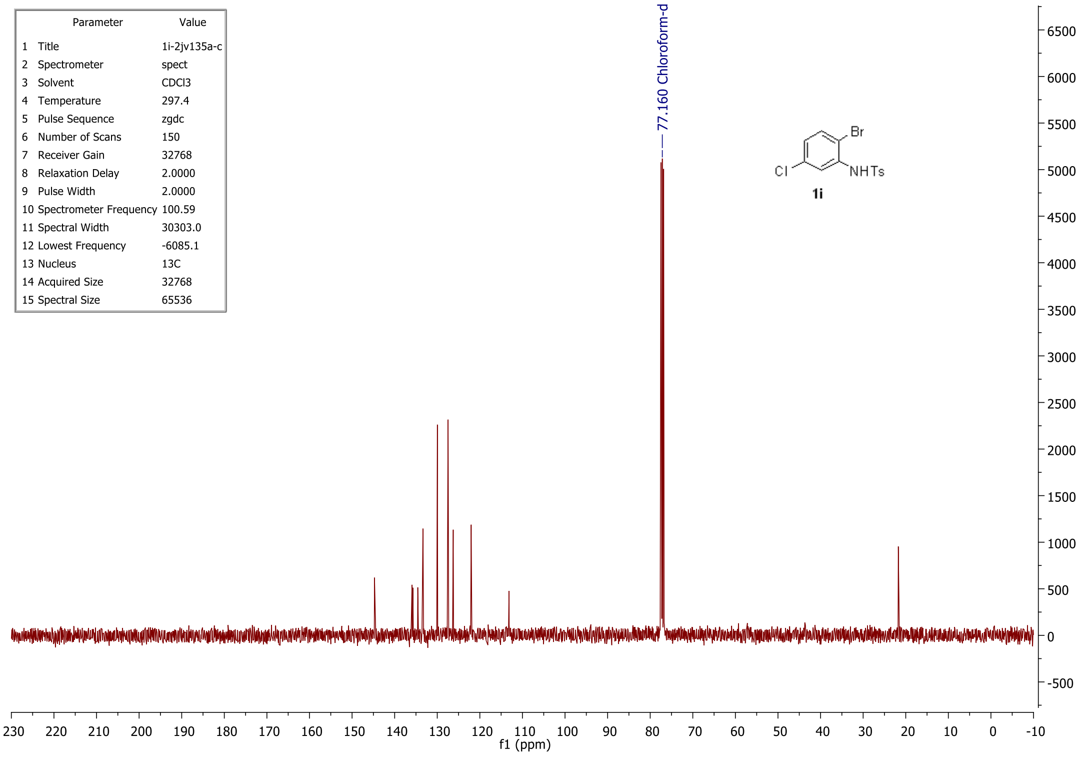

	Parameter	Value
1	Title	1i-2jv135a-c
2	Spectrometer	spect
3	Solvent	CDCl3
4	Temperature	297.4
5	Pulse Sequence	zgdc
6	Number of Scans	150
7	Receiver Gain	32768
8	Relaxation Delay	2.0000
9	Pulse Width	2.0000
10	Spectrometer Frequency	100.59
11	Spectral Width	30303.0
12	Lowest Frequency	-6085.1
13	Nucleus	13C
14	Acquired Size	32768
15	Spectral Size	65536

N-(2-Bromophenyl-5-(trifluoromethyl)phenyl)-4-methylbenzenesulfonamide (1j)

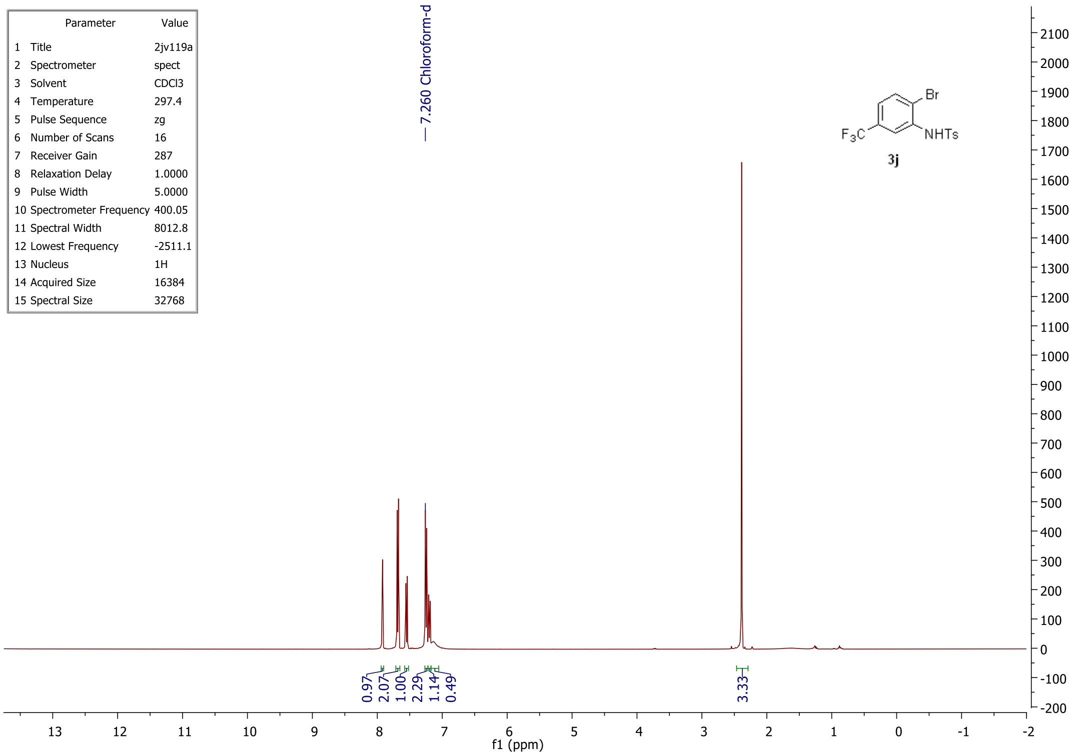

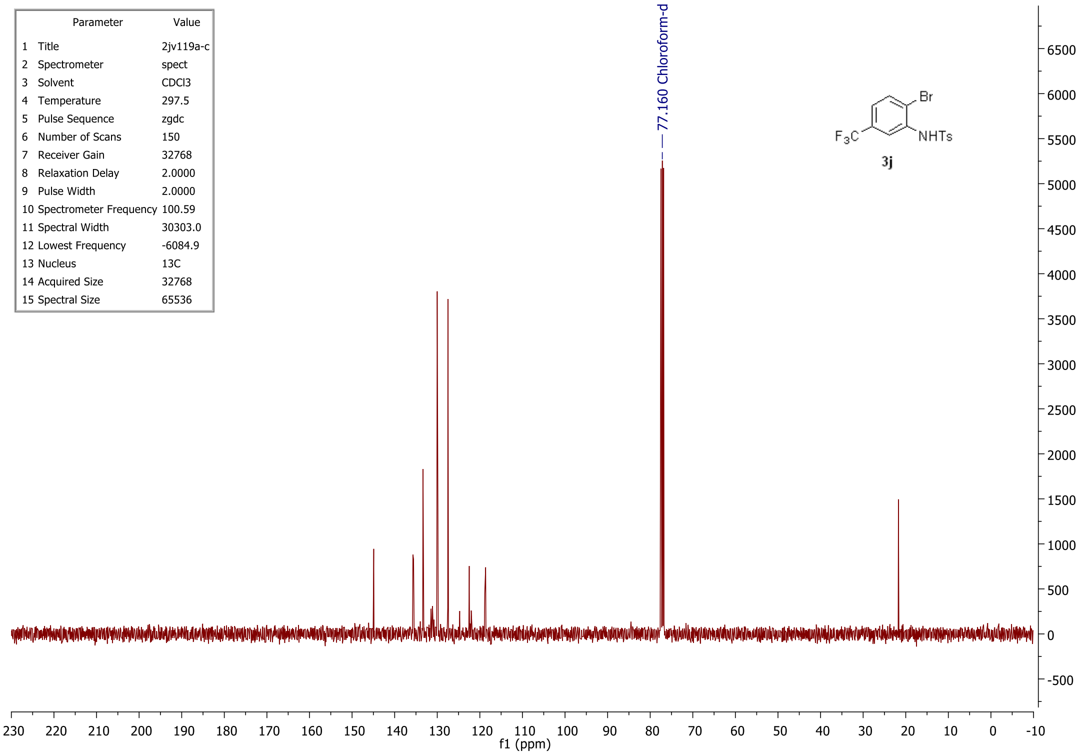

N-(2-Bromophenyl-4,6-dimethylphenyl)-4-methylbenzenesulfonamide (1k)

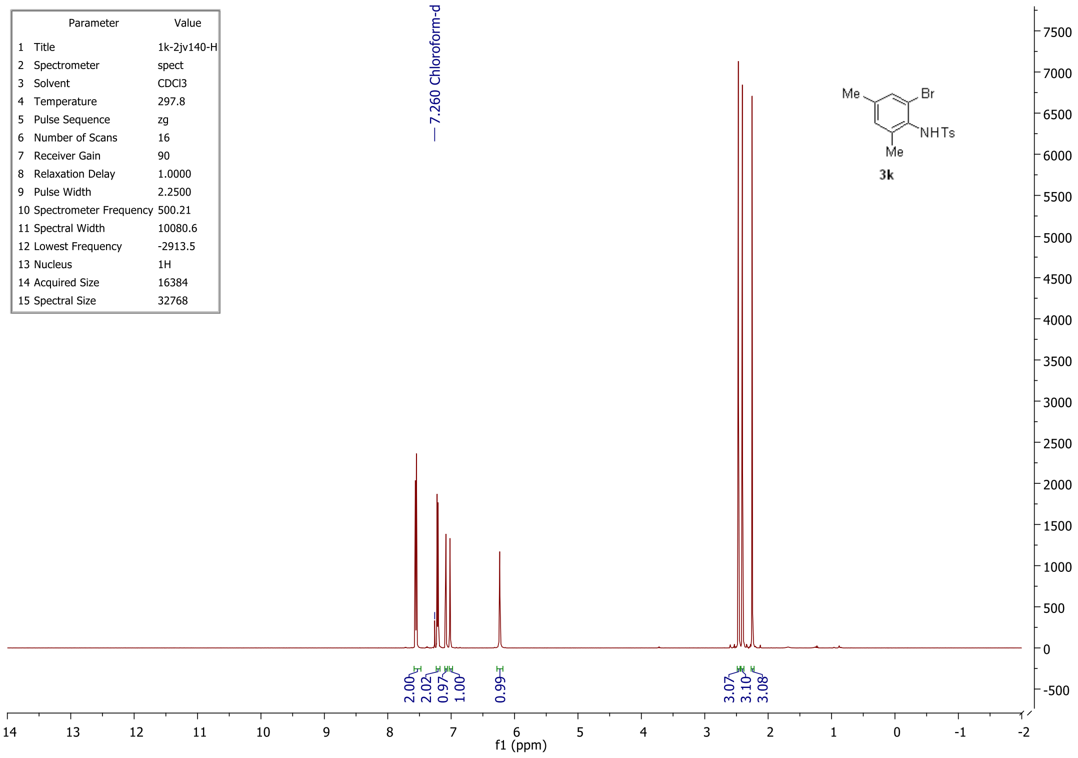

	Parameter	Value
1	Title	1k-2jv140-H
2	Spectrometer	spect
3	Solvent	CDCl3
4	Temperature	297.8
5	Pulse Sequence	zg
6	Number of Scans	16
7	Receiver Gain	90
8	Relaxation Delay	1.0000
9	Pulse Width	2.2500
10	Spectrometer Frequency	500.21
11	Spectral Width	10080.6
12	Lowest Frequency	-2913.5
13	Nucleus	1H
14	Acquired Size	16384
15	Spectral Size	32768

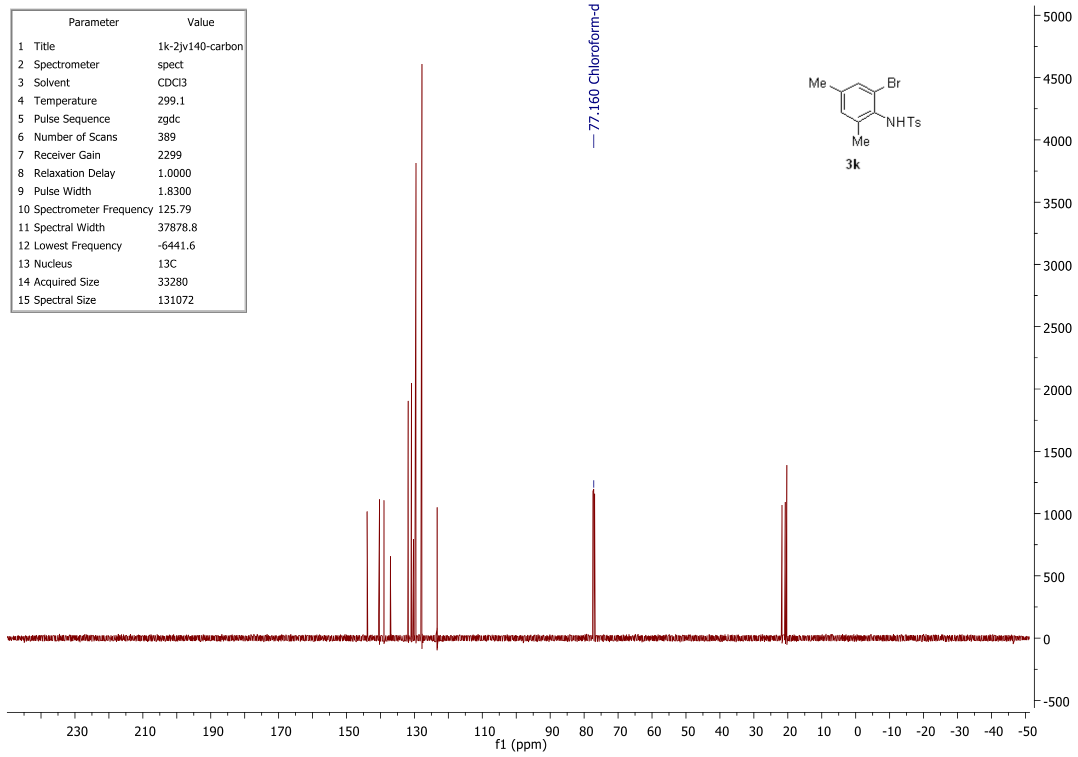

	Parameter	Value
1	Title	1k-2jv140-carbon
2	Spectrometer	spect
3	Solvent	CDCl3
4	Temperature	299.1
5	Pulse Sequence	zgdc
6	Number of Scans	389
7	Receiver Gain	2299
8	Relaxation Delay	1.0000
9	Pulse Width	1.8300
10	Spectrometer Frequency	125.79
11	Spectral Width	37878.8
12	Lowest Frequency	-6441.6
13	Nucleus	13C
14	Acquired Size	33280
15	Spectral Size	131072

N-(2-Bromophenyl-3-methylphenyl)-4-methylbenzenesulfonamide (1l)

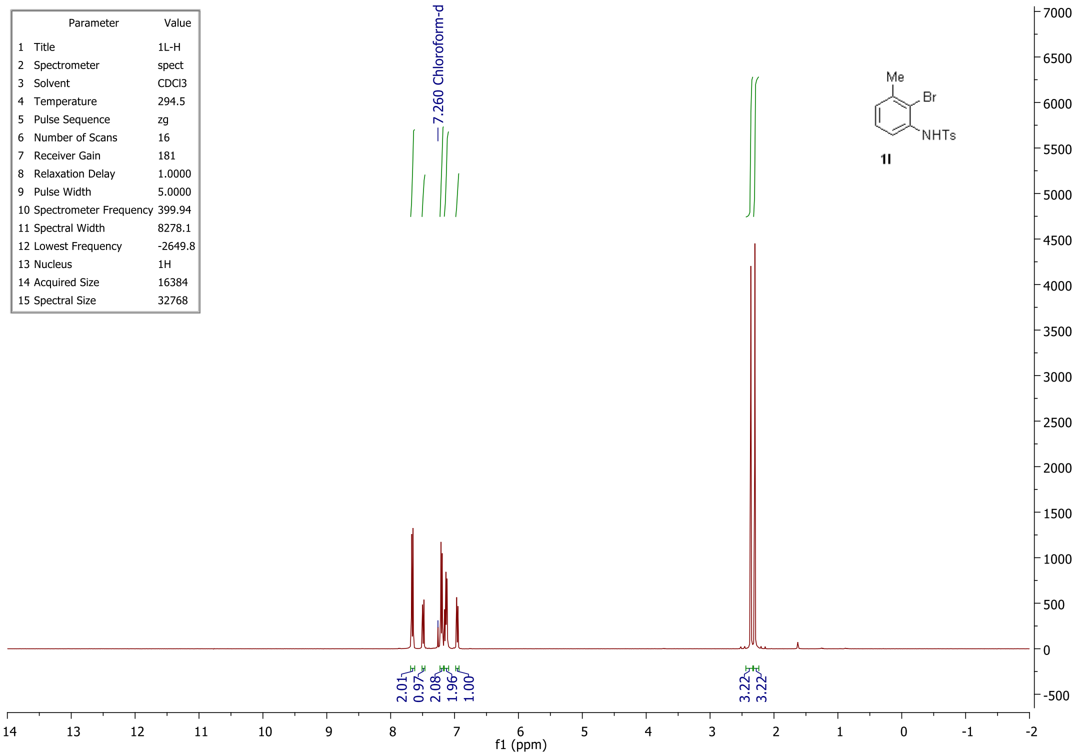

	Parameter	Value
1	Title	1L-H
2	Spectrometer	spect
3	Solvent	CDCl3
4	Temperature	294.5
5	Pulse Sequence	zg
6	Number of Scans	16
7	Receiver Gain	181
8	Relaxation Delay	1.0000
9	Pulse Width	5.0000
10	Spectrometer Frequency	399.94
11	Spectral Width	8278.1
12	Lowest Frequency	-2649.8
13	Nucleus	1H
14	Acquired Size	16384
15	Spectral Size	32768

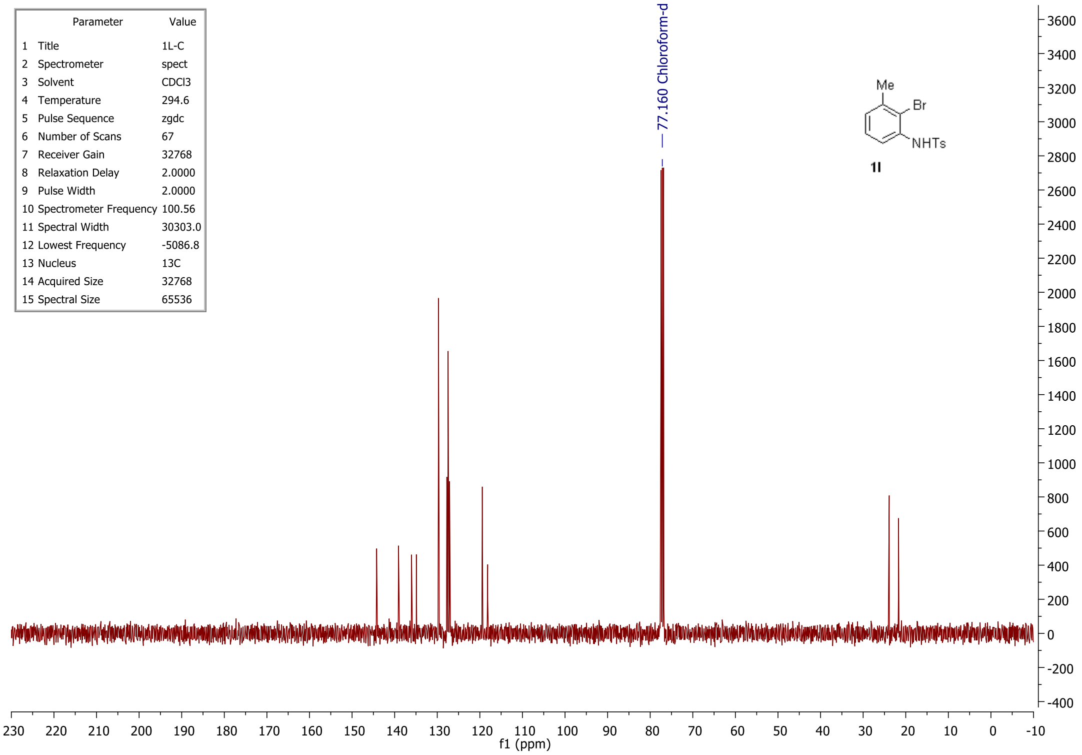

	Parameter	Value
1	Title	1L-C
2	Spectrometer	spect
3	Solvent	CDCl3
4	Temperature	294.6
5	Pulse Sequence	zgdc
6	Number of Scans	67
7	Receiver Gain	32768
8	Relaxation Delay	2.0000
9	Pulse Width	2.0000
10	Spectrometer Frequency	100.56
11	Spectral Width	30303.0
12	Lowest Frequency	-5086.8
13	Nucleus	13C
14	Acquired Size	32768
15	Spectral Size	65536

Ethyl 3-bromo-4-((4-methylphenyl)sulfonamido)benzoate (1m)

Parameter		Value
1	Title	1m-2jv146p-H
2	Spectrometer	spect
3	Solvent	CDCl3
4	Temperature	297.4
5	Pulse Sequence	zg
6	Number of Scans	16
7	Receiver Gain	512
8	Relaxation Delay	1.0000
9	Pulse Width	5.0000
10	Spectrometer Frequency	400.05
11	Spectral Width	8012.8
12	Lowest Frequency	-2512.3
13	Nucleus	1H
14	Acquired Size	16384
15	Spectral Size	32768

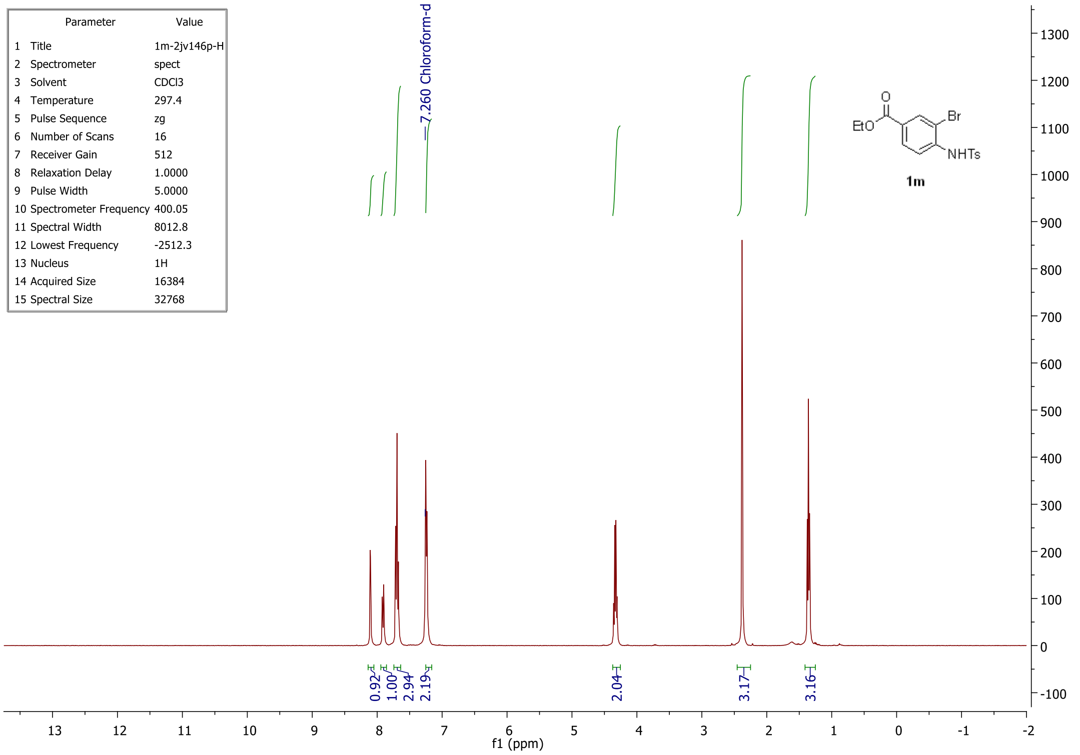

Parameter		Value
1	Title	1M-pure-C
2	Spectrometer	spect
3	Solvent	DMSO-d6
4	Temperature	298.1
5	Pulse Sequence	zgdc
6	Number of Scans	78
7	Receiver Gain	32768
8	Relaxation Delay	1.0000
9	Pulse Width	1.8300
10	Spectrometer Frequency	125.79
11	Spectral Width	37878.8
12	Lowest Frequency	-6511.6
13	Nucleus	13C
14	Acquired Size	33280
15	Spectral Size	131072

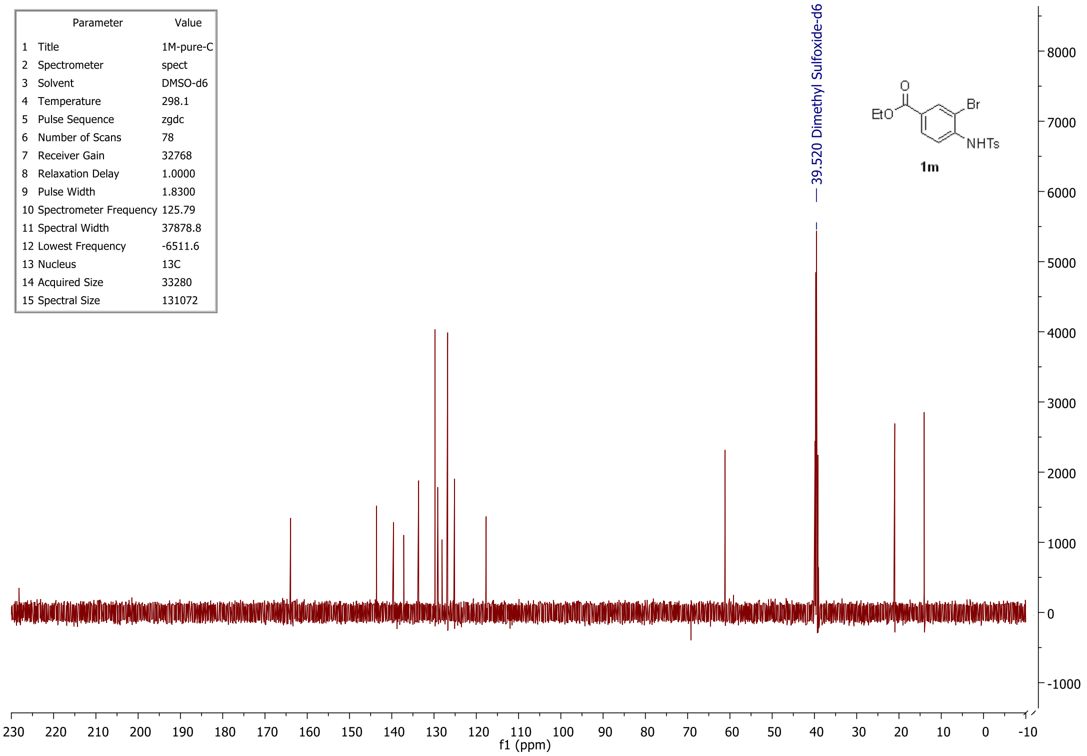

N-(4-Acetyl-2-bromophenyl)-4-methylbenzenesulfonamide (1n)

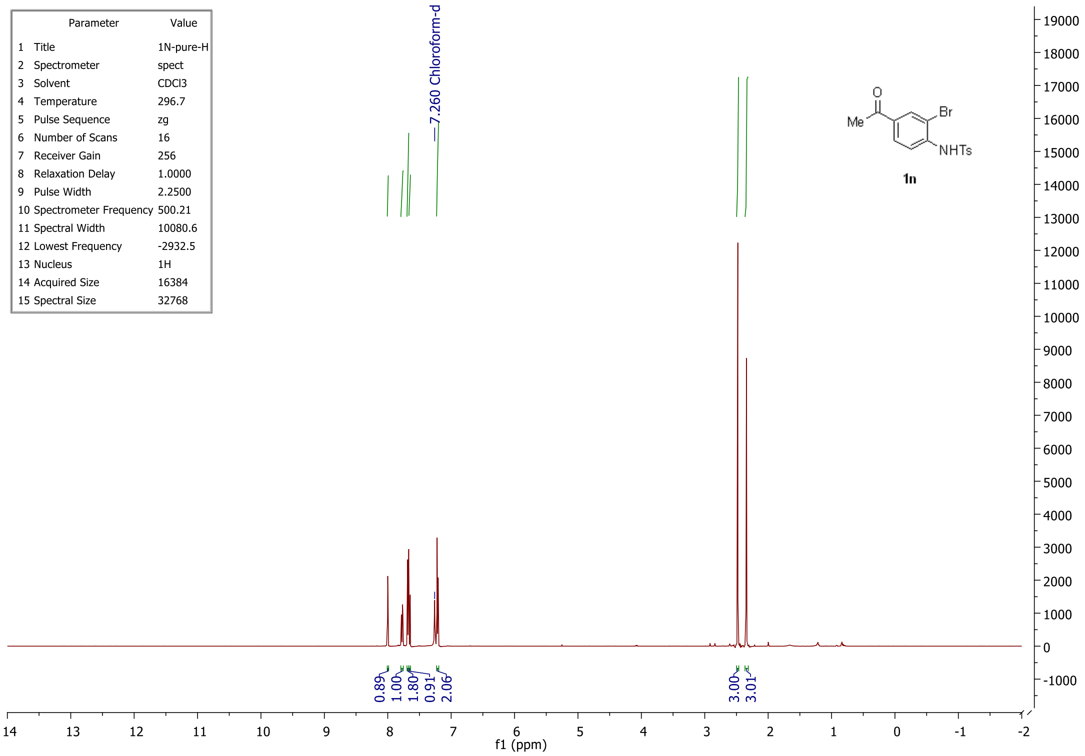

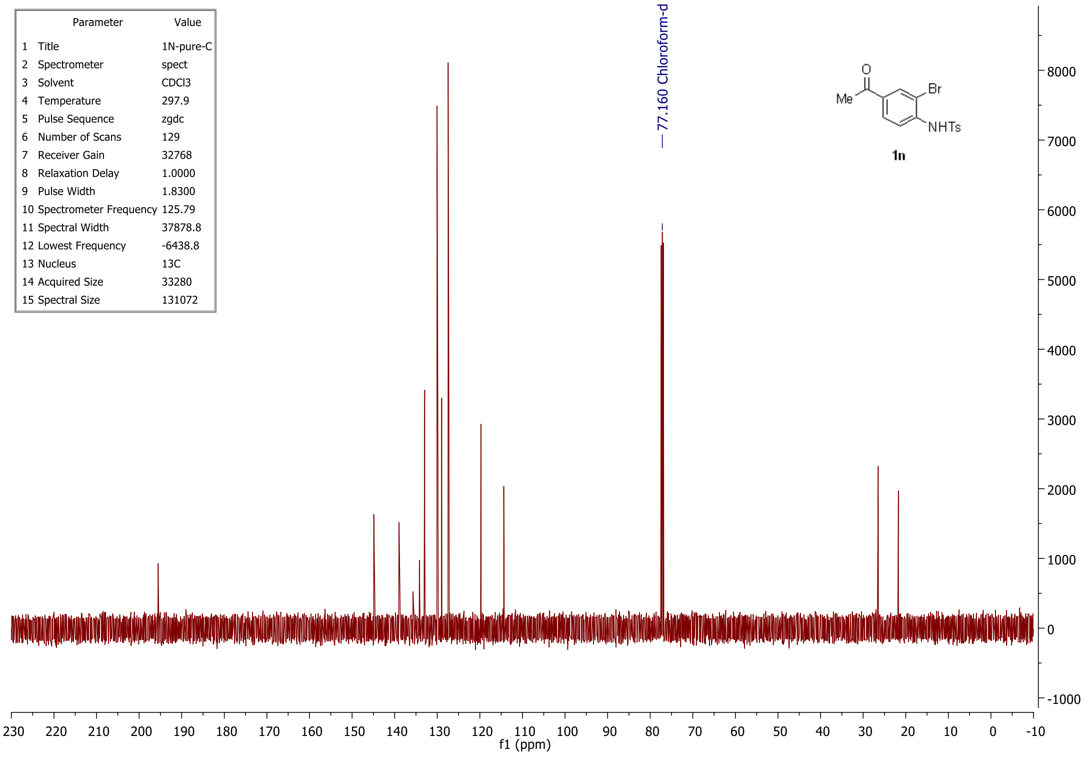

4-amino-3-bromo-*N*,*N*-diisopropylbenzamide

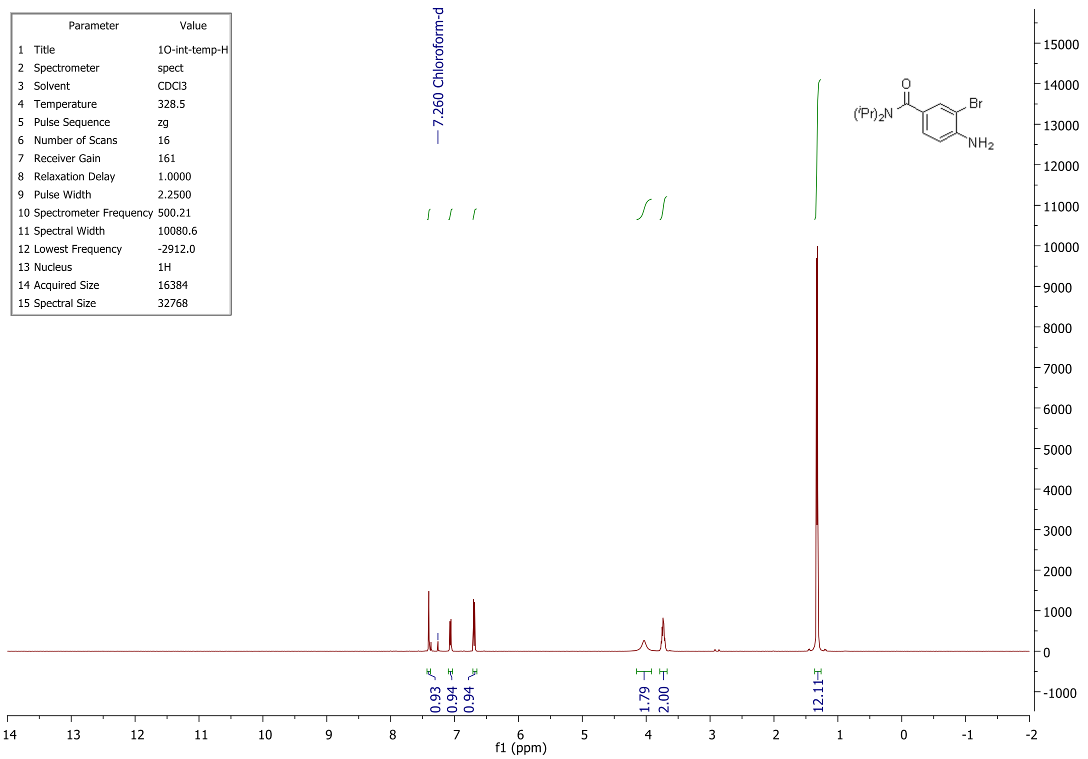

	Parameter	Value
1	Title	1O-int-temp-H
2	Spectrometer	spect
3	Solvent	CDCl3
4	Temperature	328.5
5	Pulse Sequence	zg
6	Number of Scans	16
7	Receiver Gain	161
8	Relaxation Delay	1.0000
9	Pulse Width	2.2500
10	Spectrometer Frequency	500.21
11	Spectral Width	10080.6
12	Lowest Frequency	-2912.0
13	Nucleus	1H
14	Acquired Size	16384
15	Spectral Size	32768

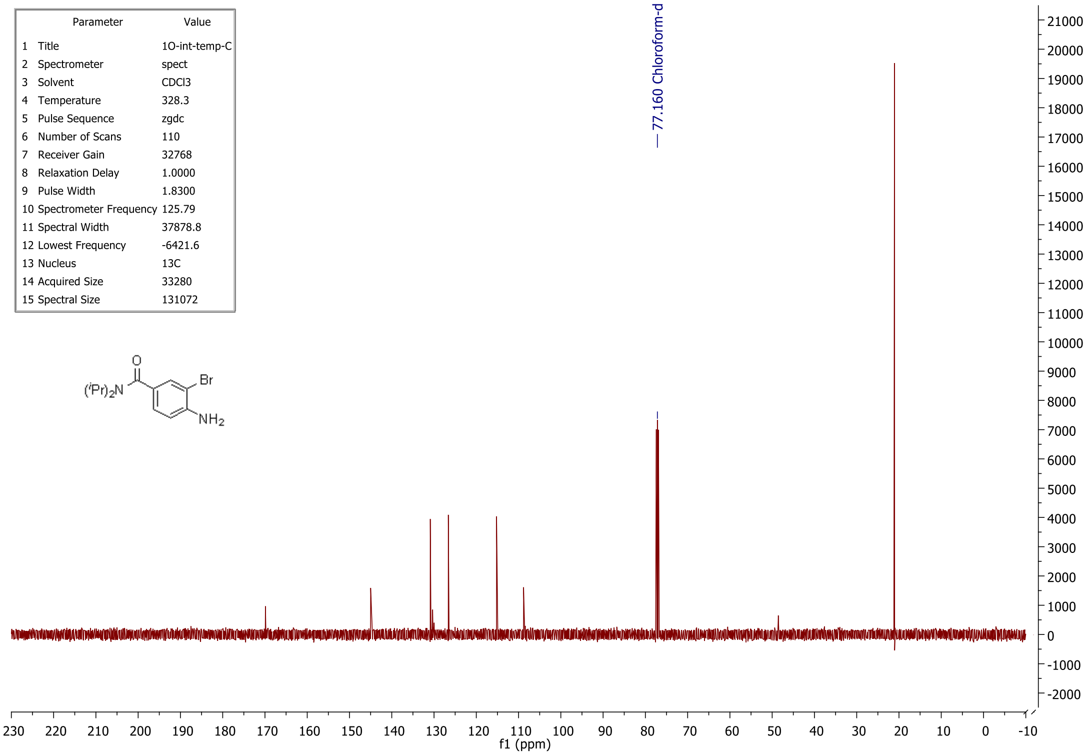

	Parameter	Value
1	Title	1O-int-temp-C
2	Spectrometer	spect
3	Solvent	CDCl3
4	Temperature	328.3
5	Pulse Sequence	zgdc
6	Number of Scans	110
7	Receiver Gain	32768
8	Relaxation Delay	1.0000
9	Pulse Width	1.8300
10	Spectrometer Frequency	125.79
11	Spectral Width	37878.8
12	Lowest Frequency	-6421.6
13	Nucleus	13C
14	Acquired Size	33280
15	Spectral Size	131072

3-Bromo-*N*,*N*-diisopropyl-4-((4-methylphenyl)sulfonamido)benzamide (1o)

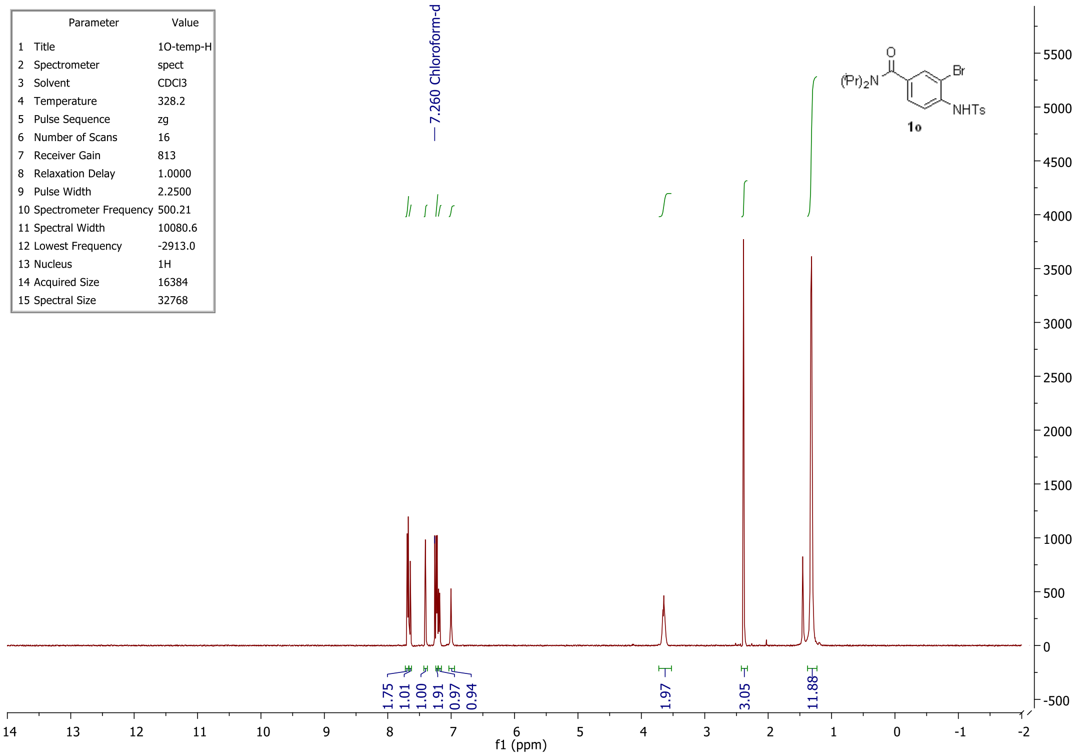

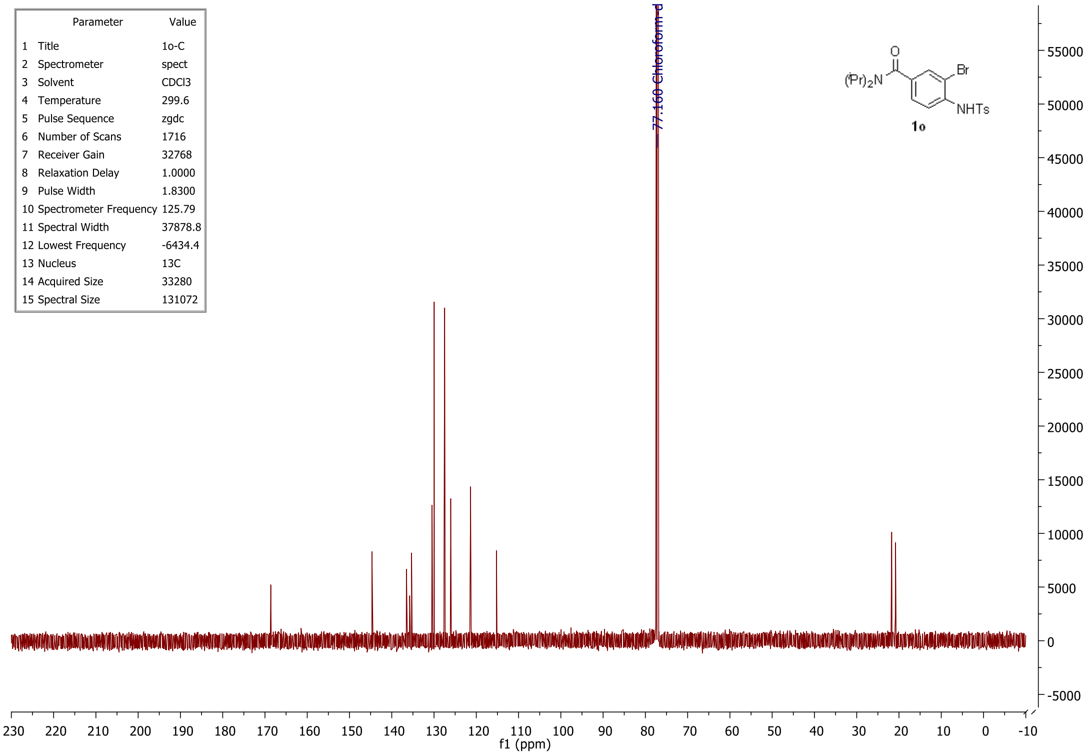

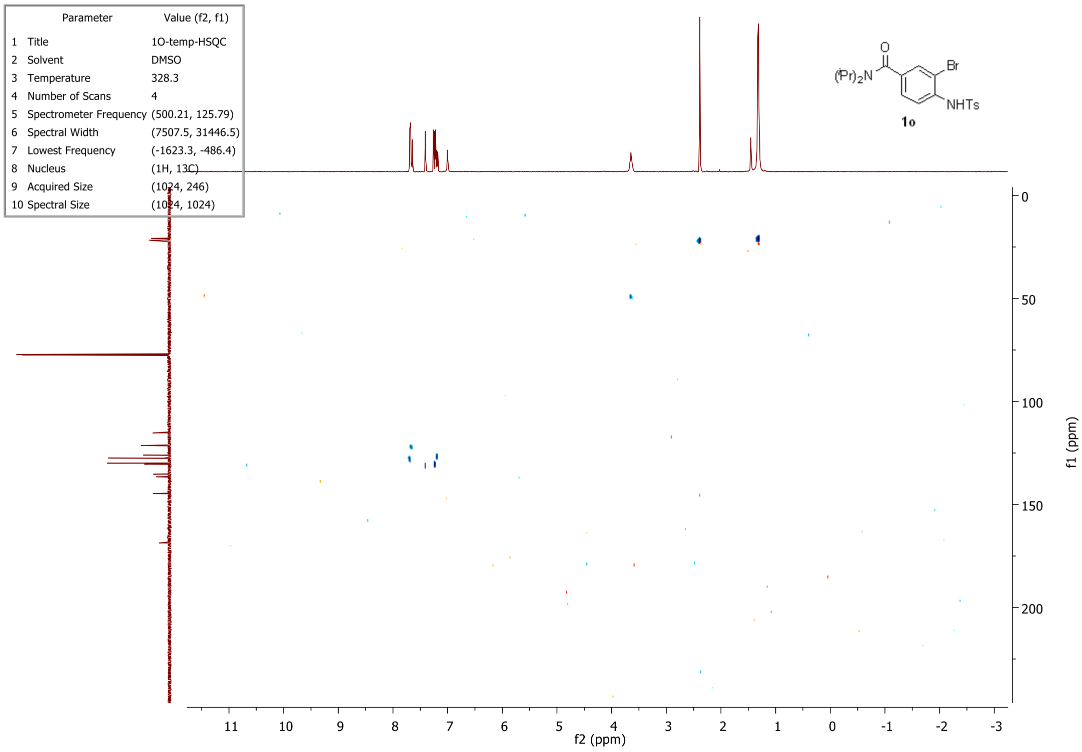

	Parameter	Value (f2, f1)
1	Title	1O-temp-HSQC
2	Solvent	DMSO
3	Temperature	328.3
4	Number of Scans	4
5	Spectrometer Frequency	(500.21, 125.79)
6	Spectral Width	(7507.5, 31446.5)
7	Lowest Frequency	(-1623.3, -486.4)
8	Nucleus	(1H, 13C)
9	Acquired Size	(1024, 246)
10	Spectral Size	(1024, 1024)

1-(2,4-Dimethylphenyl)urea (4n)

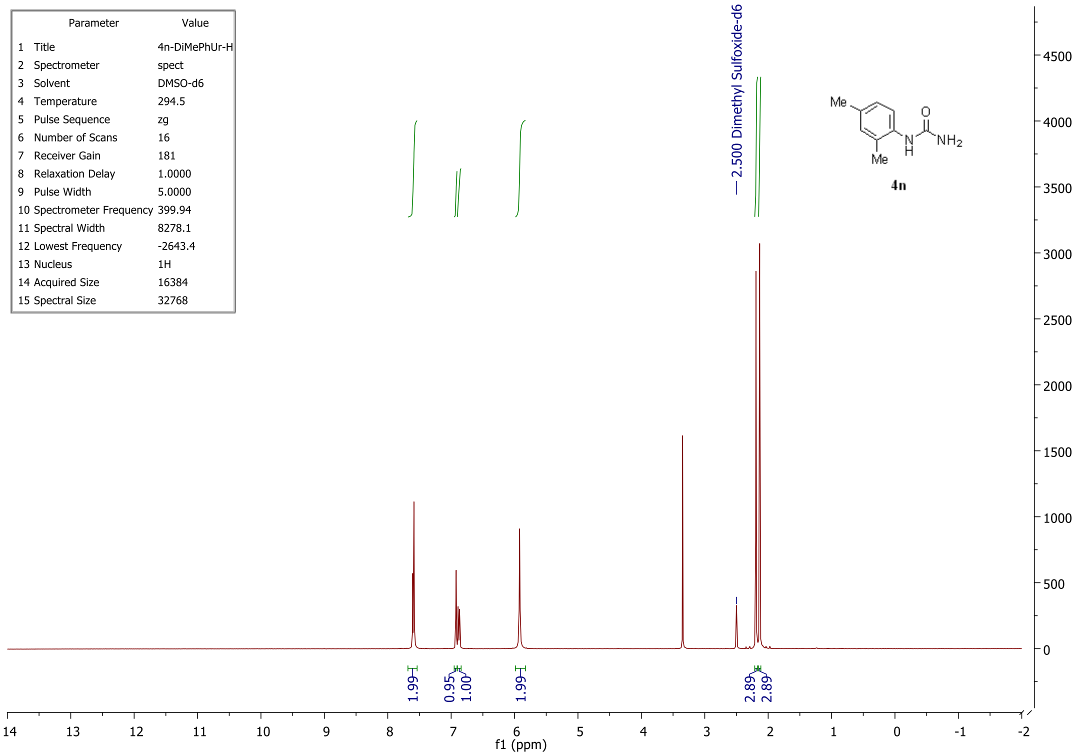

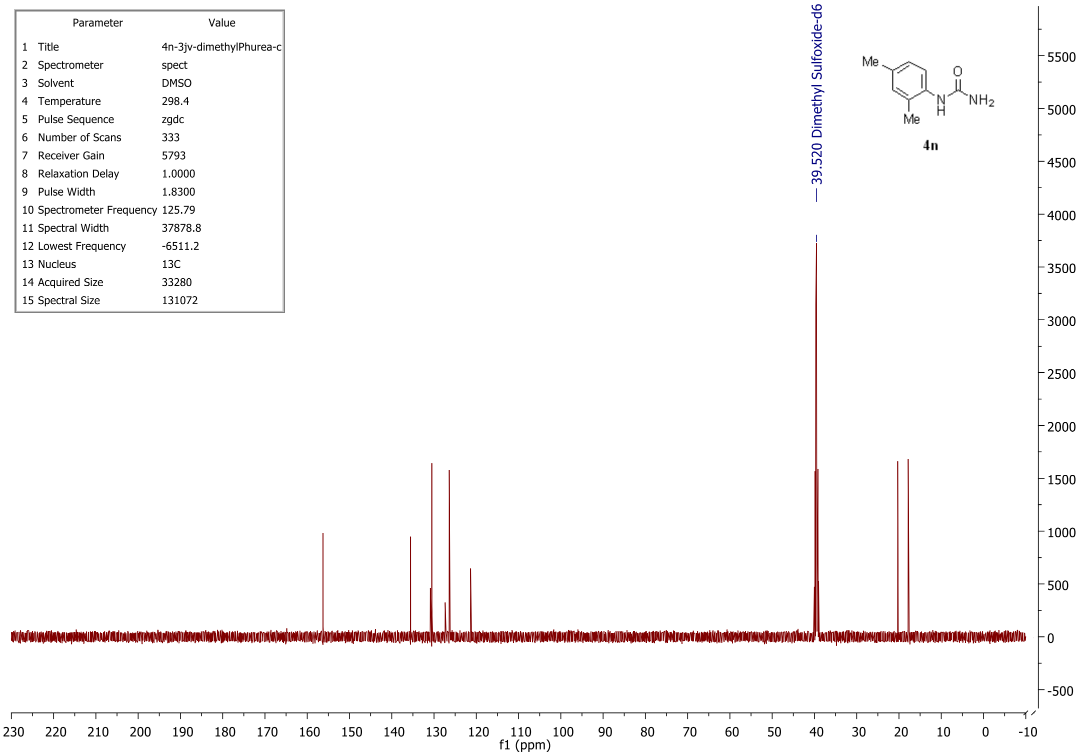

1-(2,4,6-Trimethylphenyl)urea (4o)

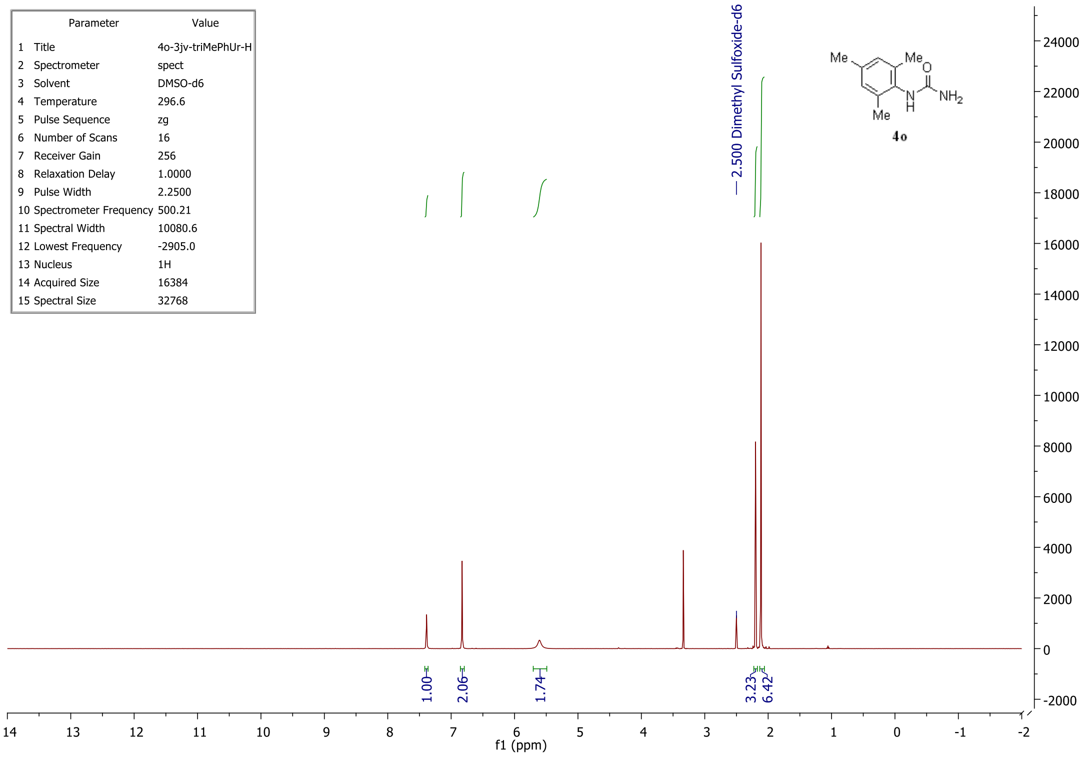

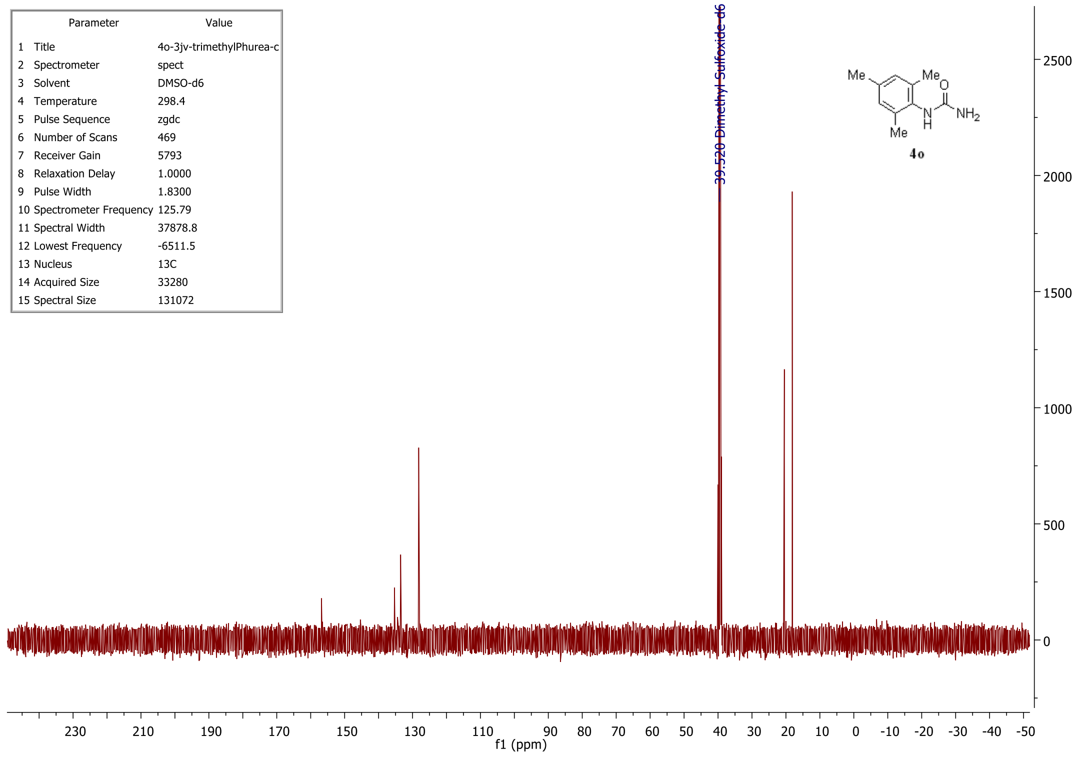

1-(4-Methoxy-2-methylphenyl)urea (4p)

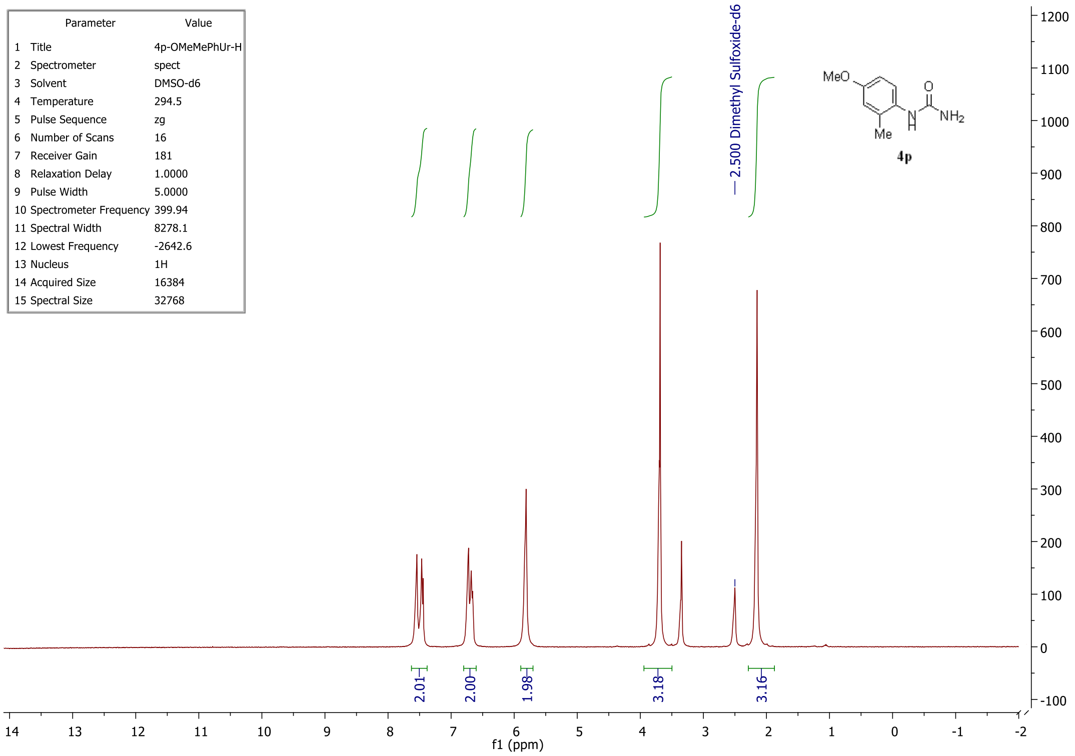

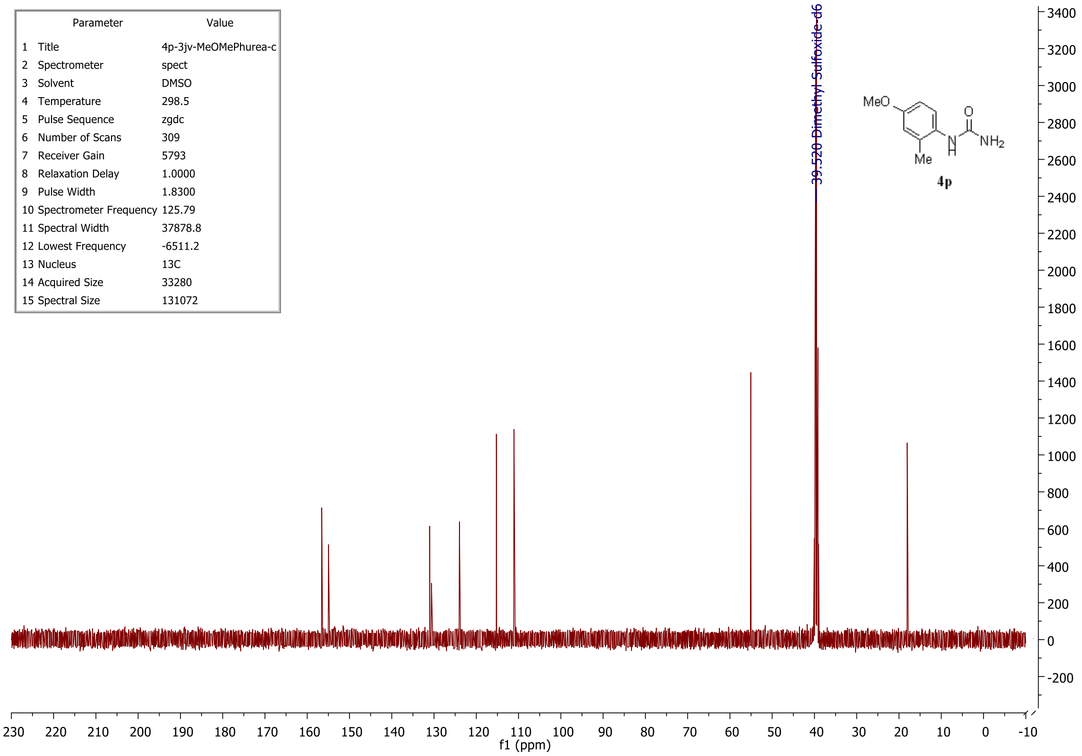

2-(6-methylhepta-1,5-dien-2-yl)-*N*-tosylindoline (3a)

	Parameter	Value
1	Title	3a-pure-H
2	Spectrometer	spect
3	Solvent	CDCl3
4	Temperature	297.7
5	Pulse Sequence	zg
6	Number of Scans	16
7	Receiver Gain	90
8	Relaxation Delay	1.0000
9	Pulse Width	2.2500
10	Spectrometer Frequency	500.21
11	Spectral Width	10080.6
12	Lowest Frequency	-2913.5
13	Nucleus	1H
14	Acquired Size	16384
15	Spectral Size	32768

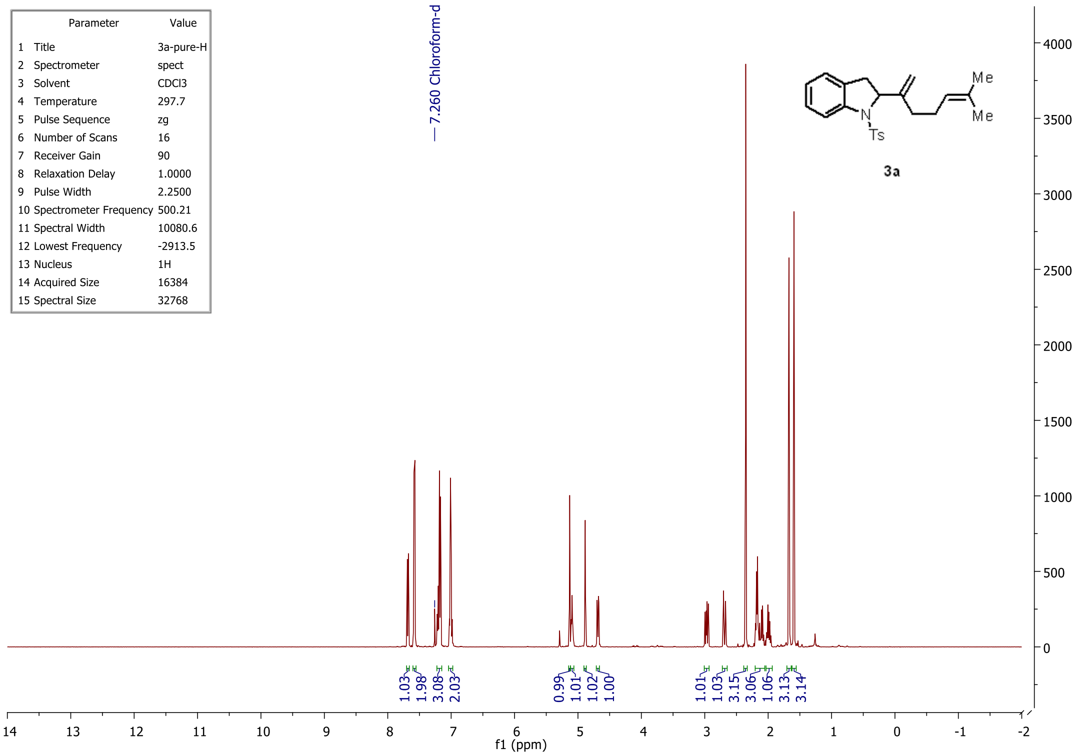

	Parameter	Value
1	Title	3a-pure-C
2	Spectrometer	spect
3	Solvent	CDCl3
4	Temperature	298.7
5	Pulse Sequence	zgdc
6	Number of Scans	95
7	Receiver Gain	32768
8	Relaxation Delay	1.0000
9	Pulse Width	1.8300
10	Spectrometer Frequency	125.79
11	Spectral Width	37878.8
12	Lowest Frequency	-6439.1
13	Nucleus	13C
14	Acquired Size	33280
15	Spectral Size	131072

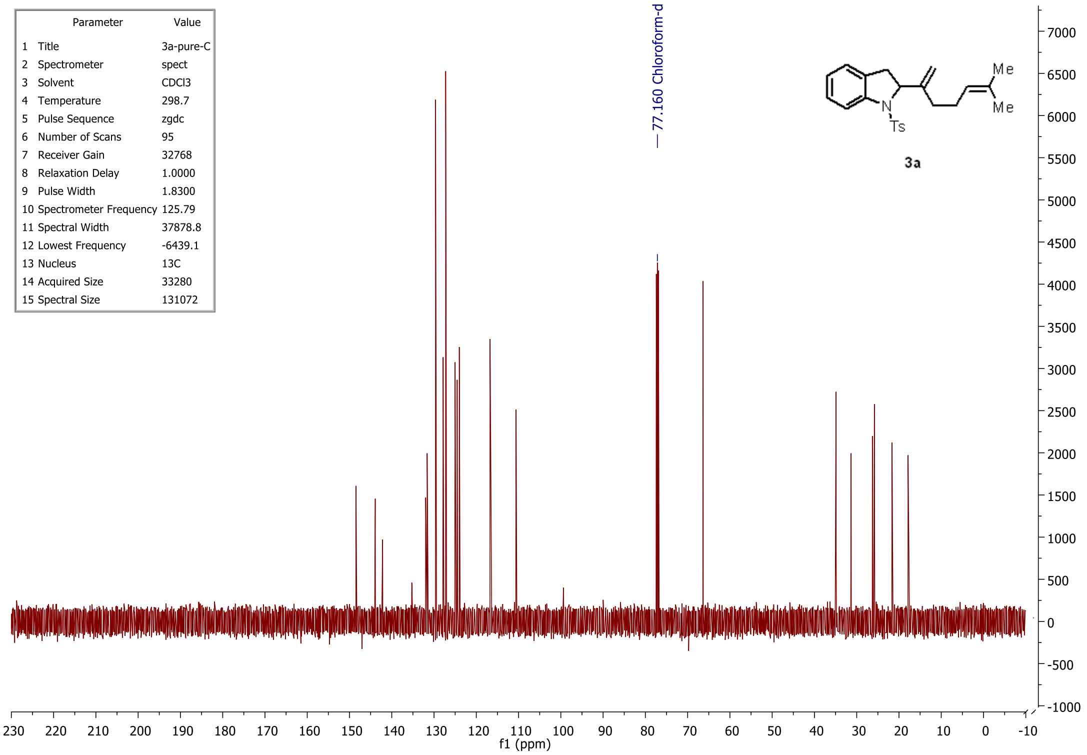

2-(4-methylpent-3-en-1-yl)-*N*-tosyl-2-vinylindoline (3a’)

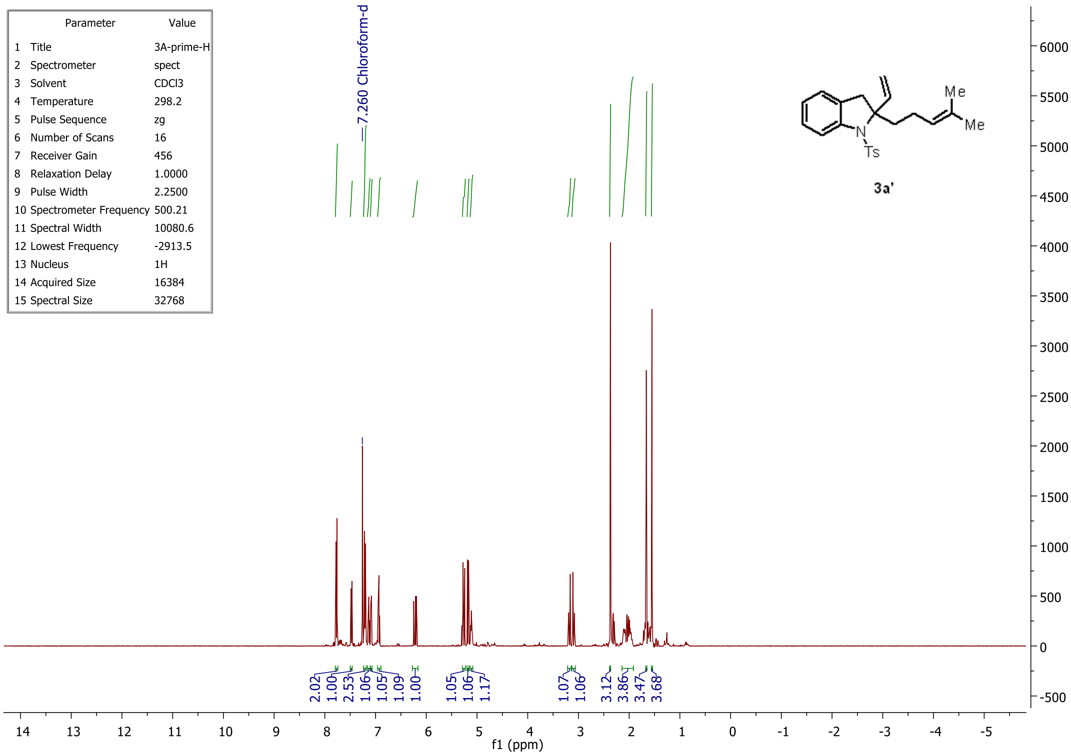

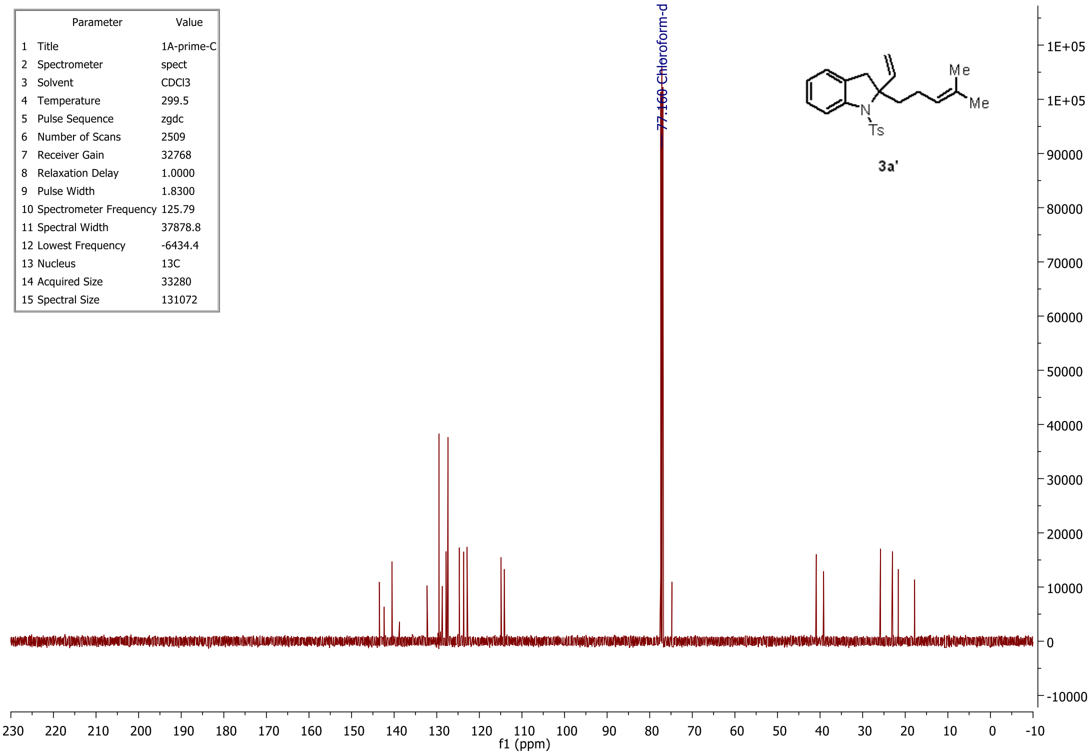

5-Methoxy-2-(6-methylhepta-1,5-dien-2-yl)-*N*-tosylindoline (3b)

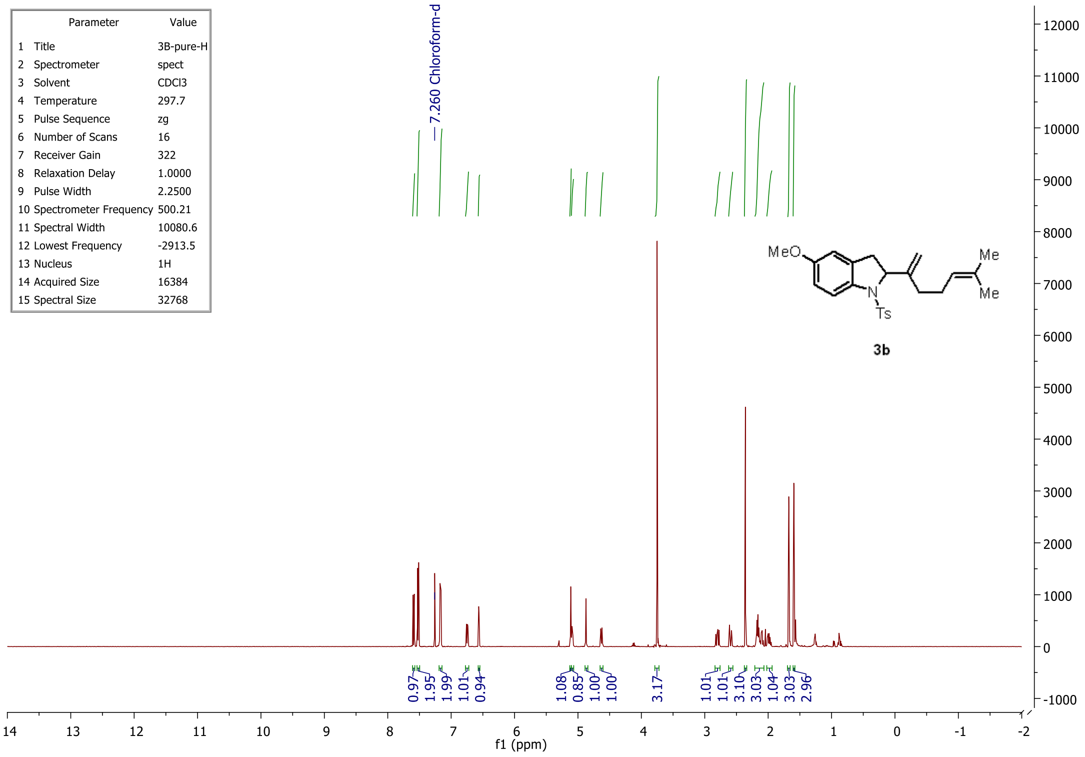

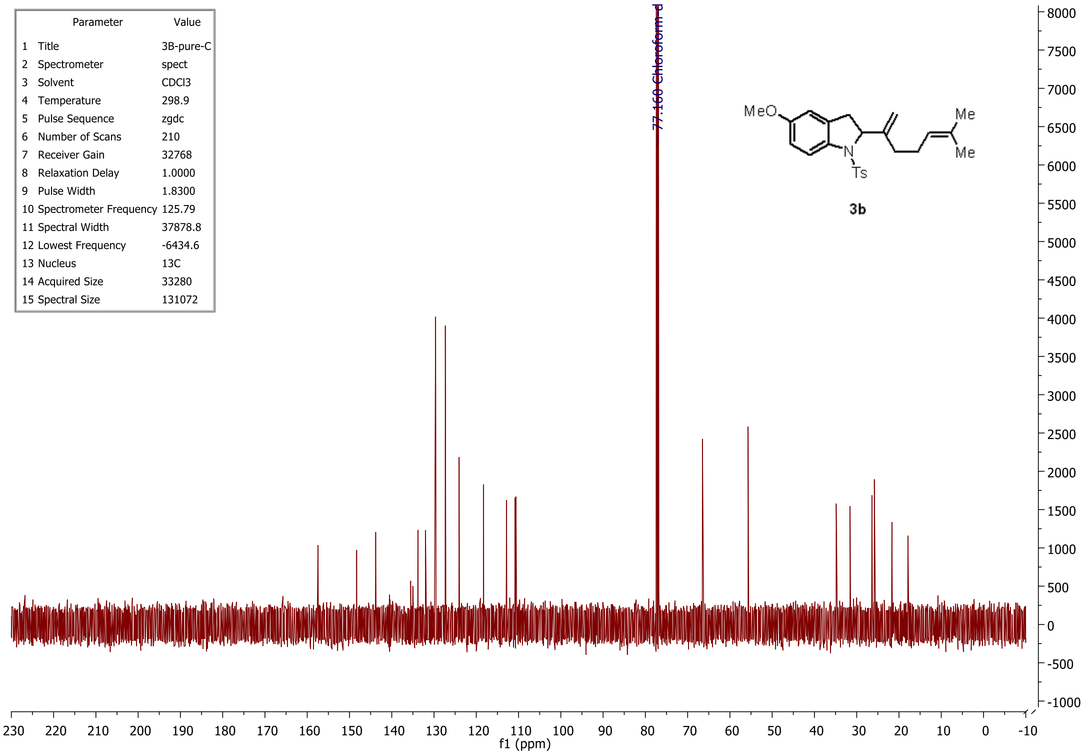

5-Methyl-2-(6-methylhepta-1,5-dien-2-yl)-N-tosylindoline (3c)

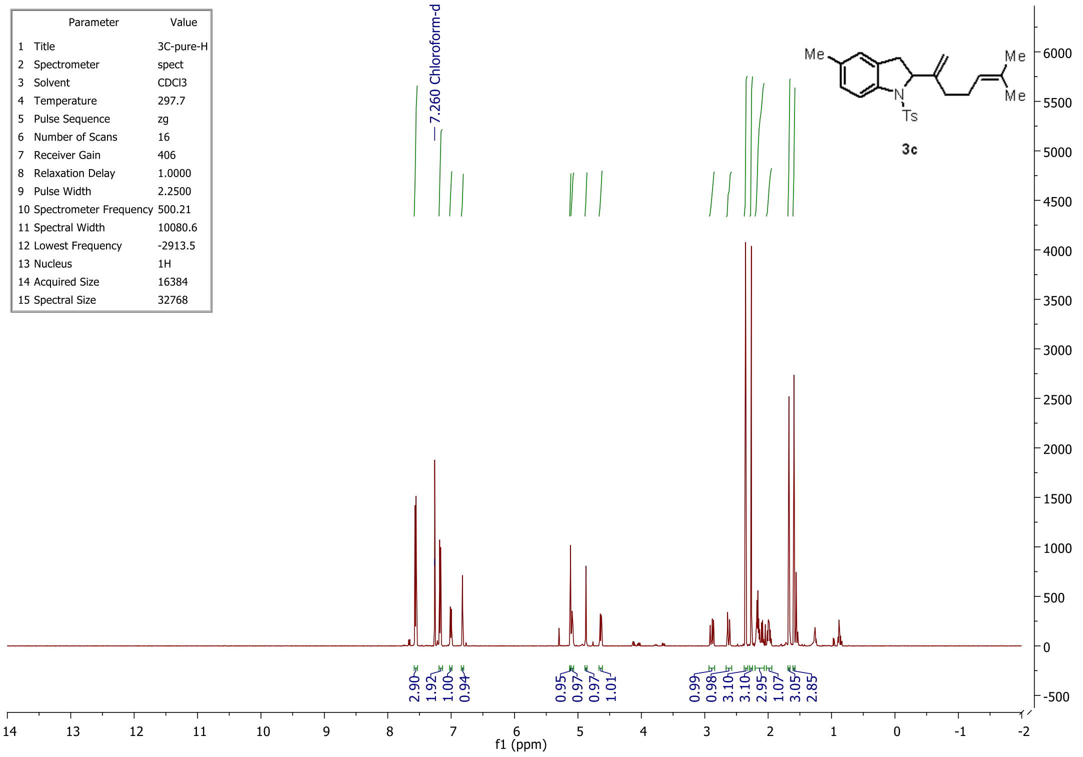

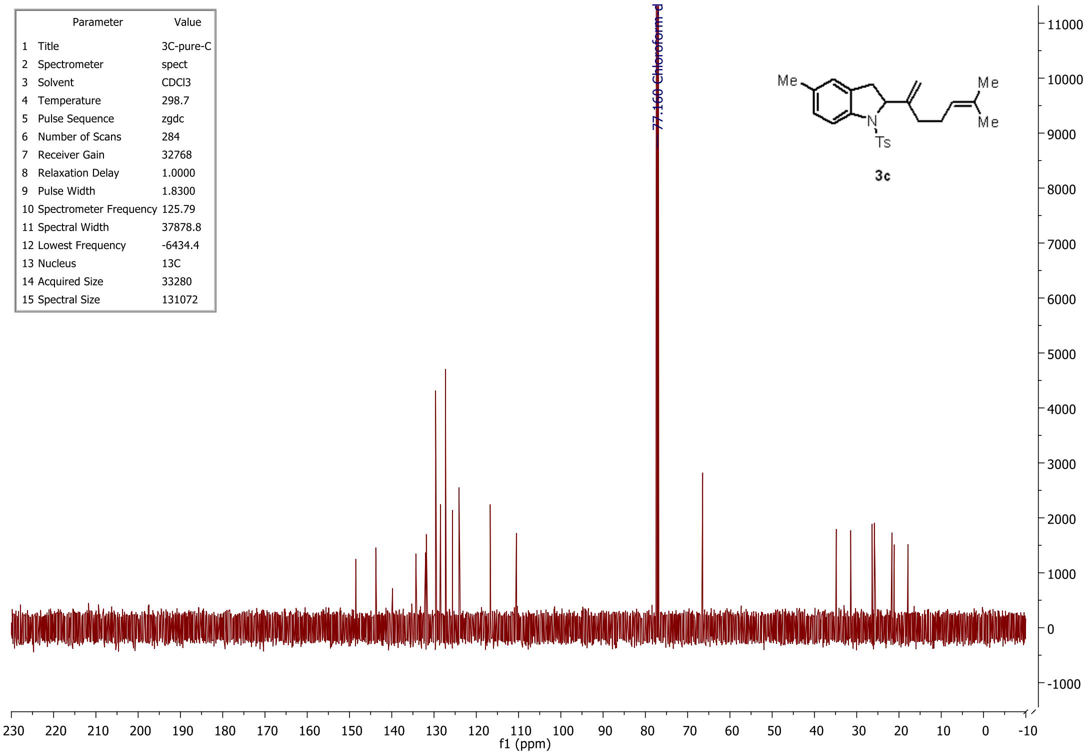

5-Fluoro-2-(6-methylhepta-1,5-dien-2-yl)-1-tosylindoline (3d)

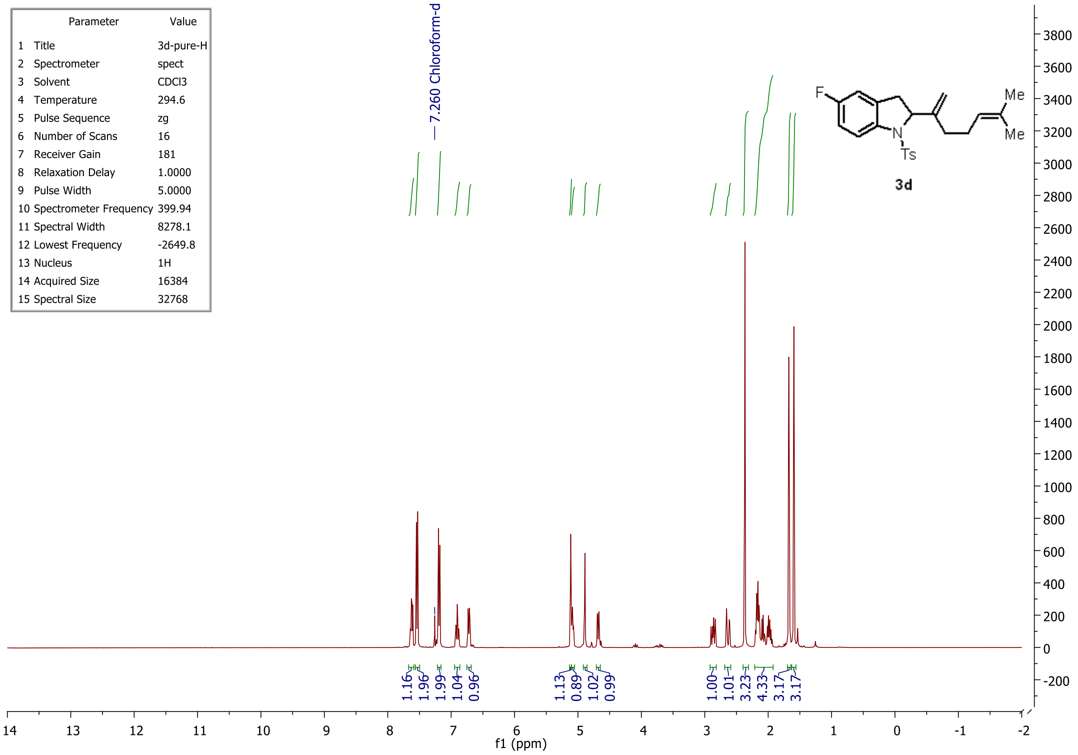

Parameter		Value
1	Title	3d-pure-H
2	Spectrometer	spect
3	Solvent	CDCl3
4	Temperature	294.6
5	Pulse Sequence	zg
6	Number of Scans	16
7	Receiver Gain	181
8	Relaxation Delay	1.0000
9	Pulse Width	5.0000
10	Spectrometer Frequency	399.94
11	Spectral Width	8278.1
12	Lowest Frequency	-2649.8
13	Nucleus	1H
14	Acquired Size	16384
15	Spectral Size	32768

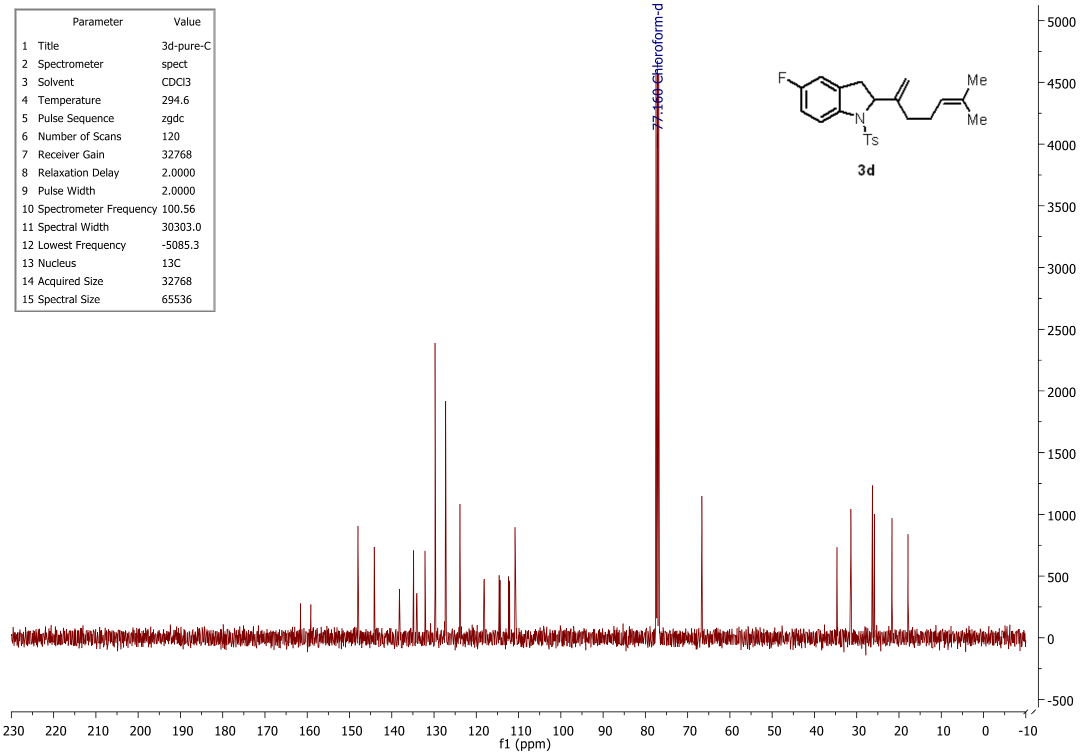

Parameter		Value
1	Title	3d-pure-C
2	Spectrometer	spect
3	Solvent	CDCl3
4	Temperature	294.6
5	Pulse Sequence	zgdc
6	Number of Scans	120
7	Receiver Gain	32768
8	Relaxation Delay	2.0000
9	Pulse Width	2.0000
10	Spectrometer Frequency	100.56
11	Spectral Width	30303.0
12	Lowest Frequency	-5085.3
13	Nucleus	13C
14	Acquired Size	32768
15	Spectral Size	65536

5-Chloro-2-(6-methylhepta-1,5-dien-2-yl)-*N*-tosylindoline (3e)

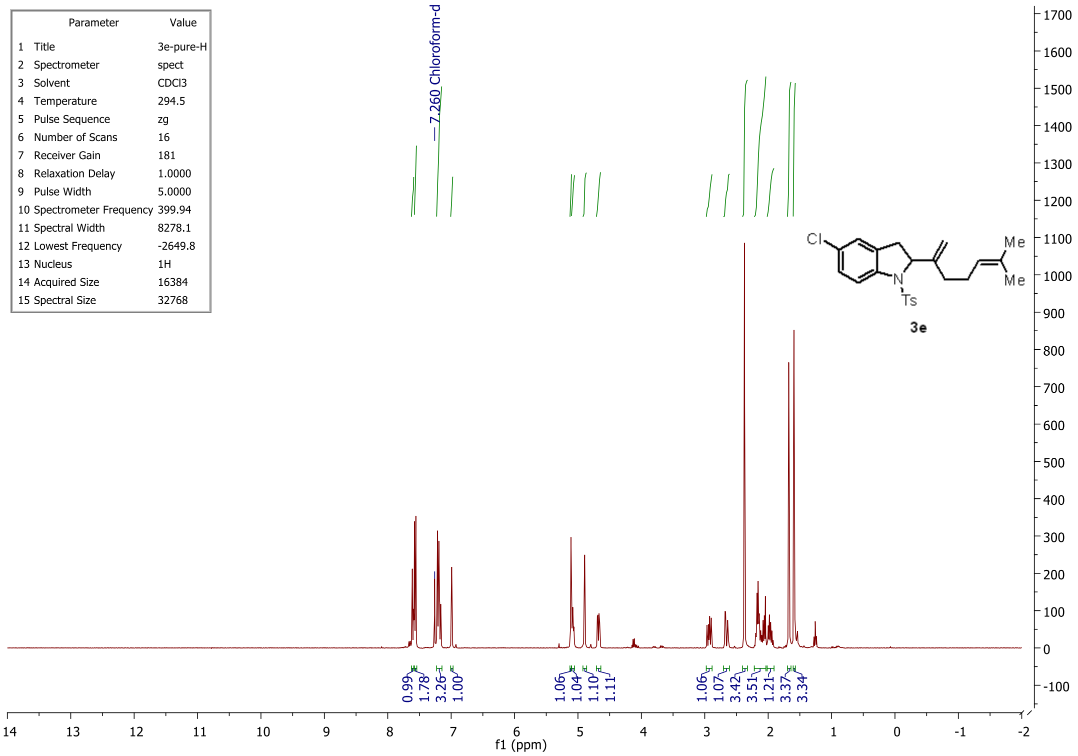

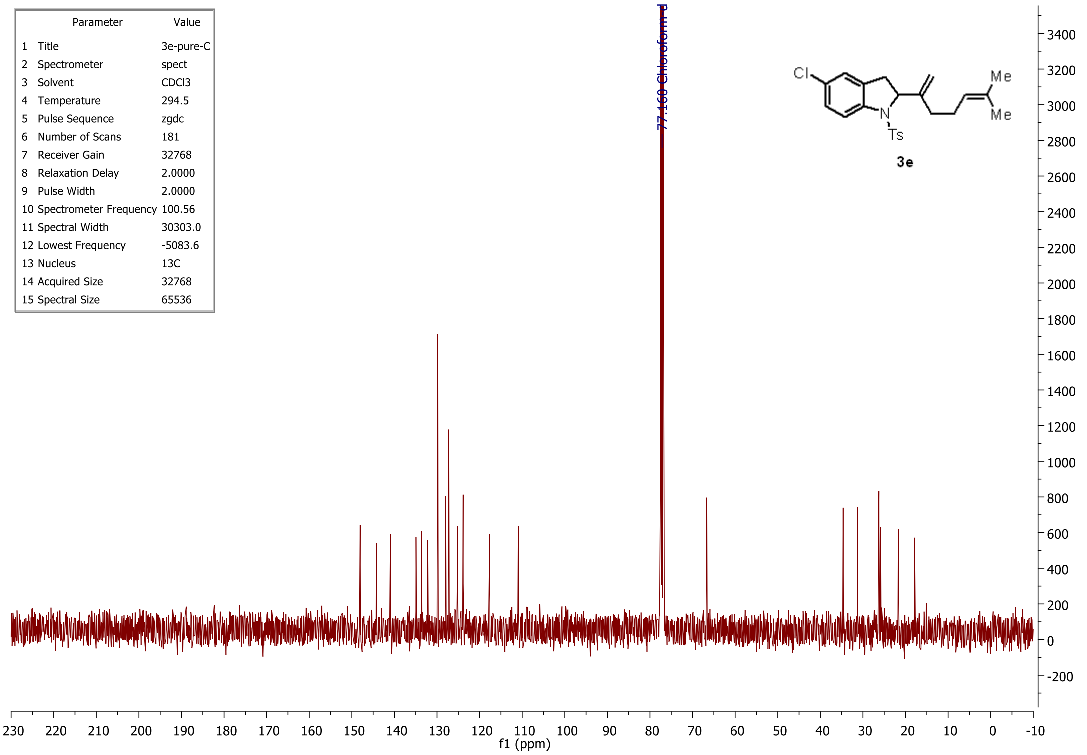

2-(6-Methylhepta-1,5-dien-2-yl)-*N*-tosyl-5-(trifluoromethoxy)indoline (3f)

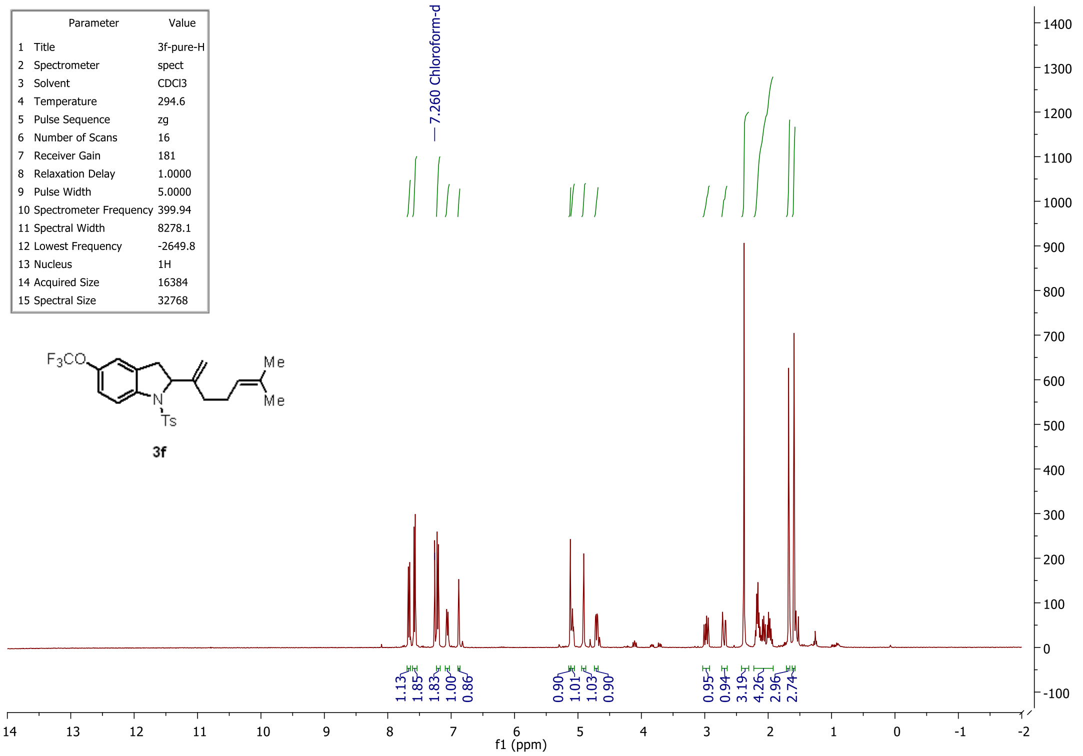

Parameter	Value
1 Title	3f-pure-H
2 Spectrometer	spect
3 Solvent	CDCl3
4 Temperature	294.6
5 Pulse Sequence	zg
6 Number of Scans	16
7 Receiver Gain	181
8 Relaxation Delay	1.0000
9 Pulse Width	5.0000
10 Spectrometer Frequency	399.94
11 Spectral Width	8278.1
12 Lowest Frequency	-2649.8
13 Nucleus	1H
14 Acquired Size	16384
15 Spectral Size	32768

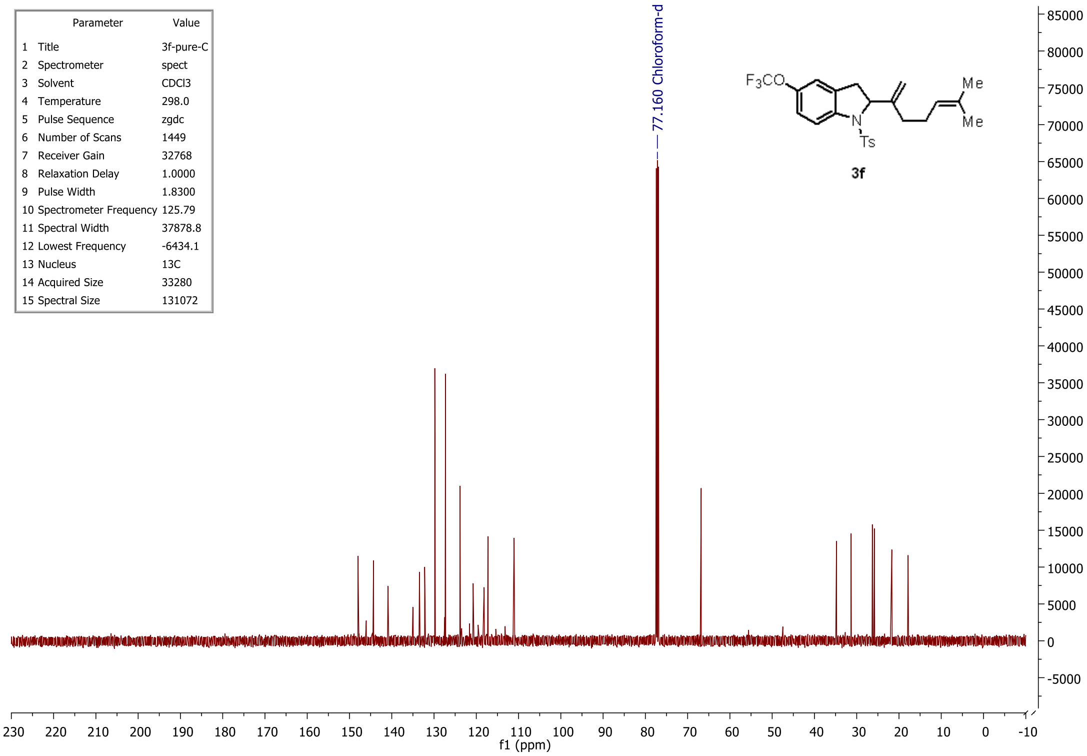

Parameter	Value
1 Title	3f-pure-C
2 Spectrometer	spect
3 Solvent	CDCl3
4 Temperature	298.0
5 Pulse Sequence	zgdc
6 Number of Scans	1449
7 Receiver Gain	32768
8 Relaxation Delay	1.0000
9 Pulse Width	1.8300
10 Spectrometer Frequency	125.79
11 Spectral Width	37878.8
12 Lowest Frequency	-6434.1
13 Nucleus	13C
14 Acquired Size	33280
15 Spectral Size	131072

2-(6-Methylhepta-1,5-dien-2-yl)-*N*-tosyl-5-(trifluoromethyl)indoline (3g)

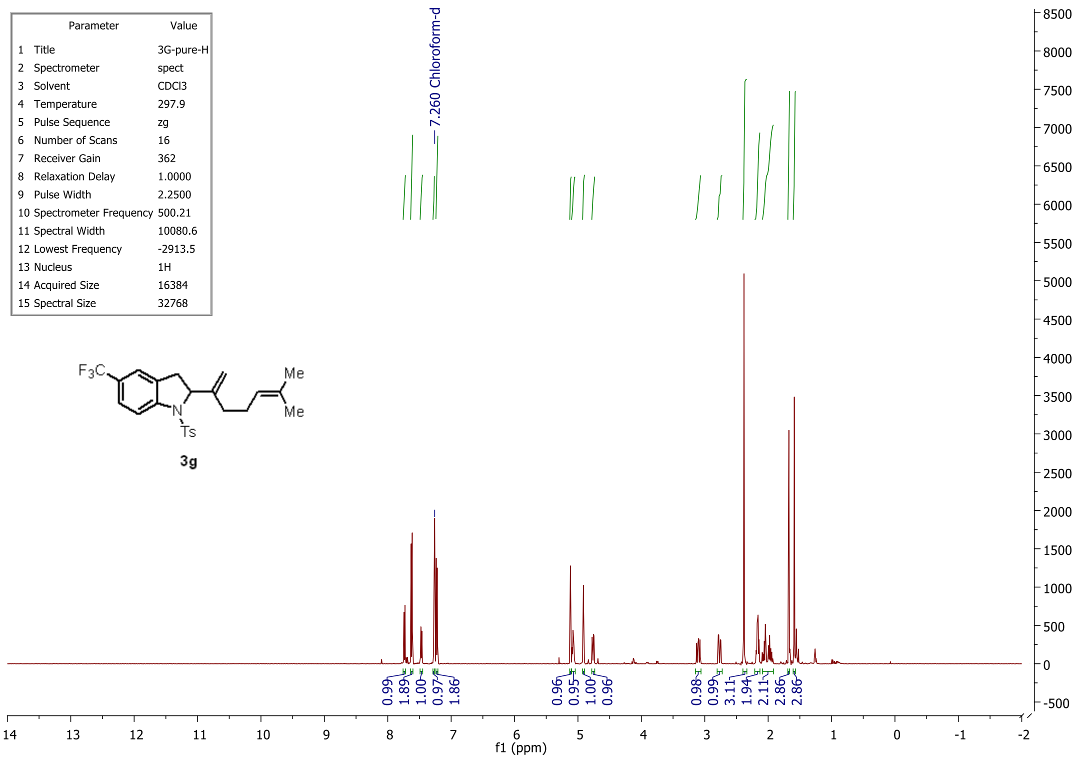

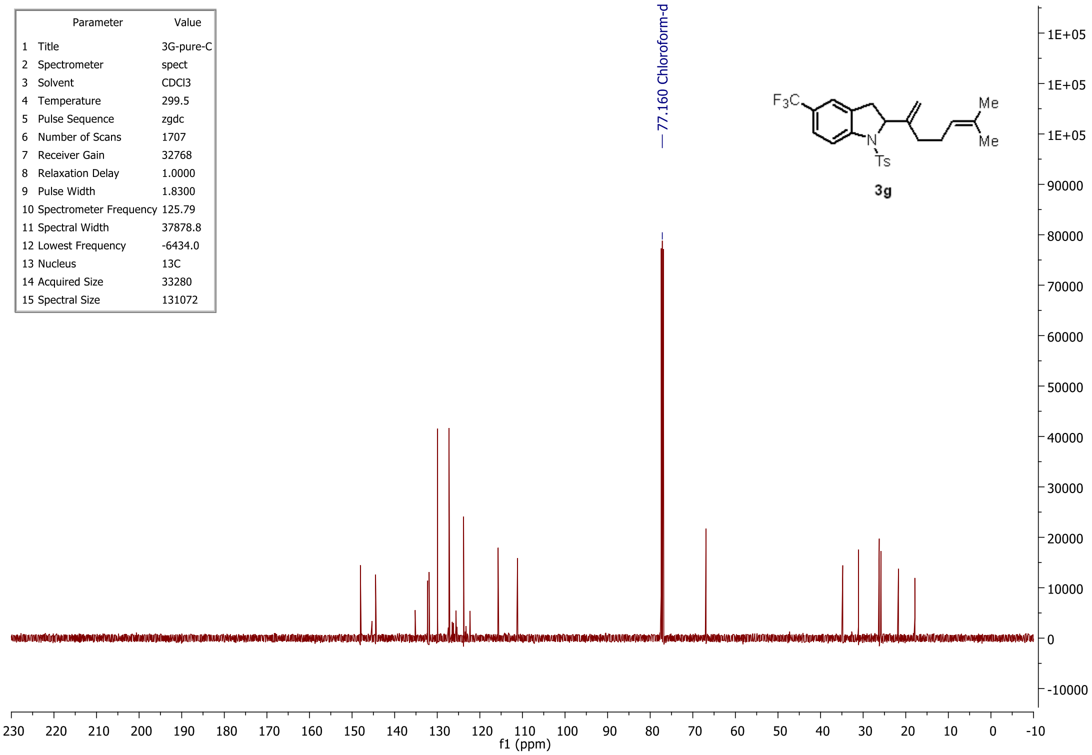

6-methyl-2-(6-methylhepta-1,5-dien-2-yl)-*N*-tosylindoline (3h)

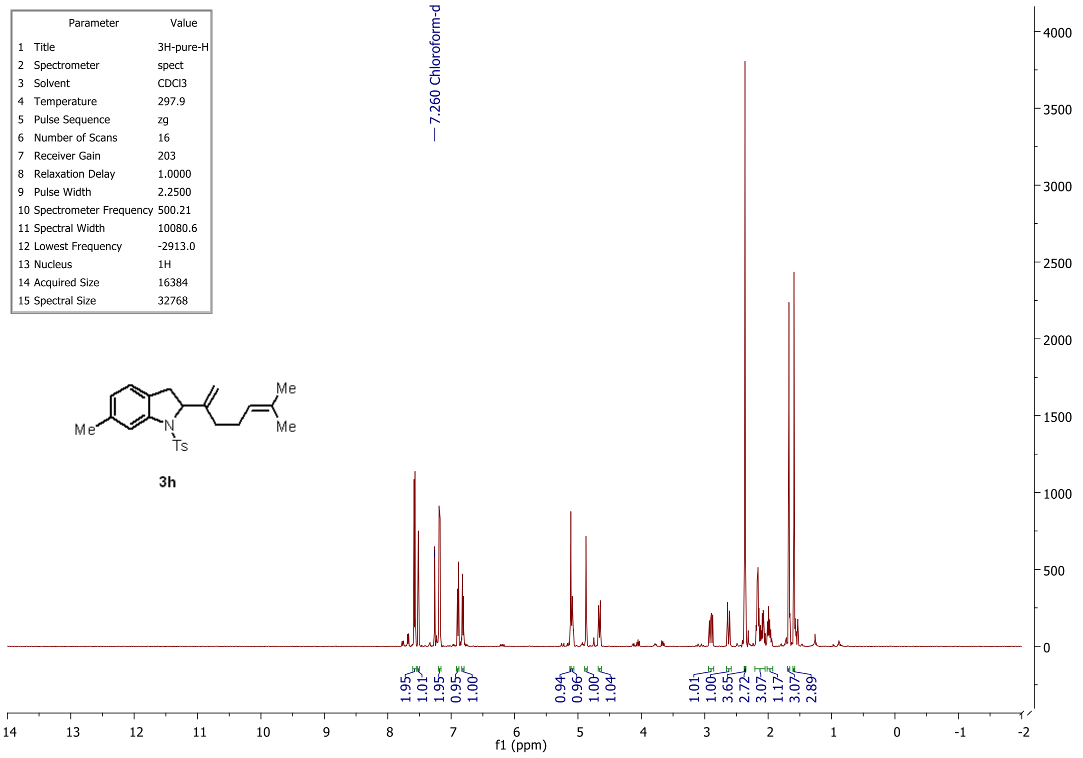

Parameter		Value
1	Title	3H-pure-H
2	Spectrometer	spect
3	Solvent	CDCl3
4	Temperature	297.9
5	Pulse Sequence	zg
6	Number of Scans	16
7	Receiver Gain	203
8	Relaxation Delay	1.0000
9	Pulse Width	2.2500
10	Spectrometer Frequency	500.21
11	Spectral Width	10080.6
12	Lowest Frequency	-2913.0
13	Nucleus	1H
14	Acquired Size	16384
15	Spectral Size	32768

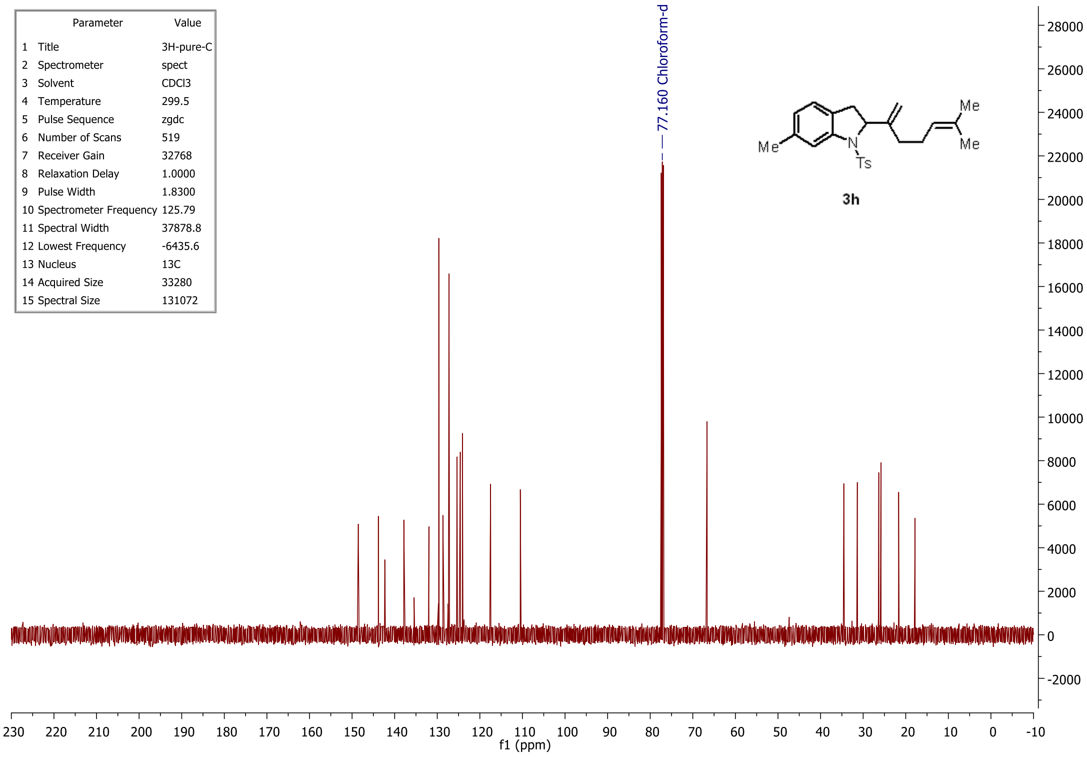

Parameter		Value
1	Title	3H-pure-C
2	Spectrometer	spect
3	Solvent	CDCl3
4	Temperature	299.5
5	Pulse Sequence	zgdc
6	Number of Scans	519
7	Receiver Gain	32768
8	Relaxation Delay	1.0000
9	Pulse Width	1.8300
10	Spectrometer Frequency	125.79
11	Spectral Width	37878.8
12	Lowest Frequency	-6435.6
13	Nucleus	13C
14	Acquired Size	33280
15	Spectral Size	131072

6-Chloro-2-(6-methylhepta-1,5-dien-2-yl)-*N*-tosylindoline (3i)

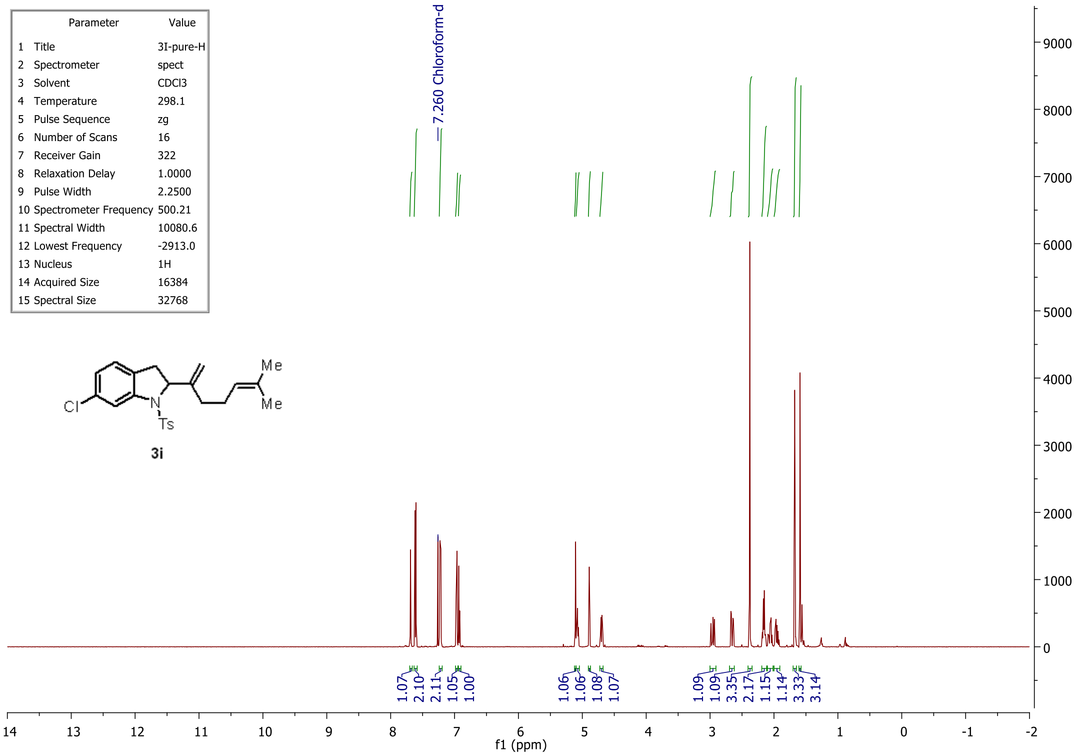

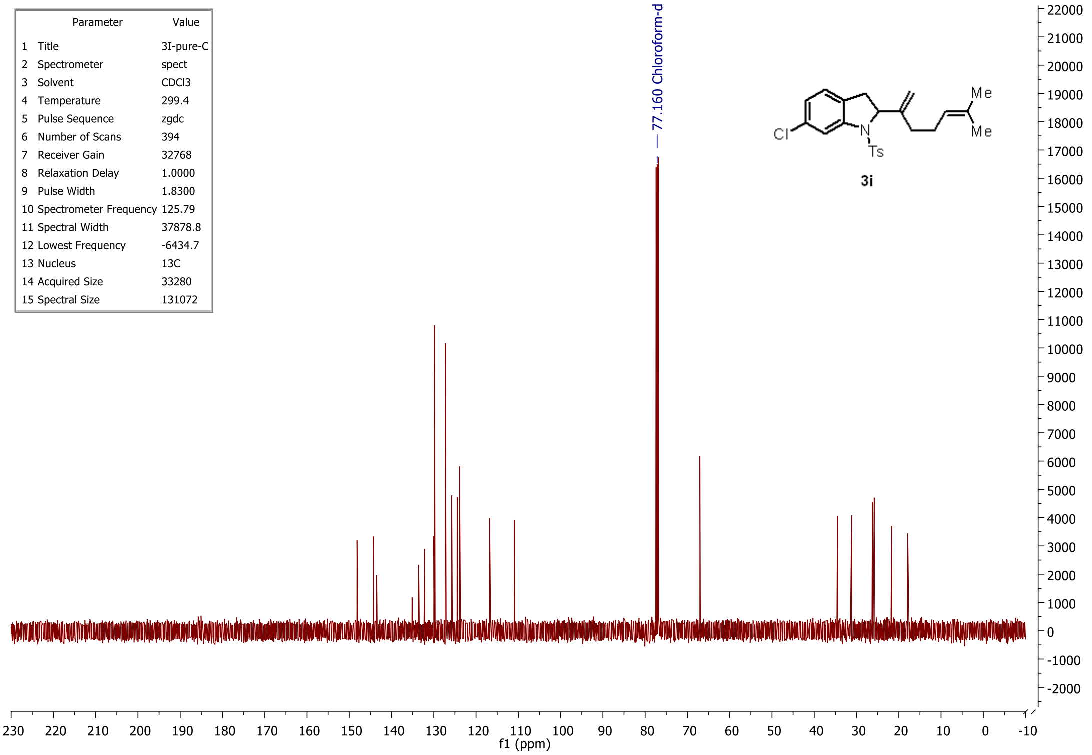

2-(6-Methylhepta-1,5-dien-2-yl)-*N*-tosyl-6-(trifluoromethyl)indoline (3j)

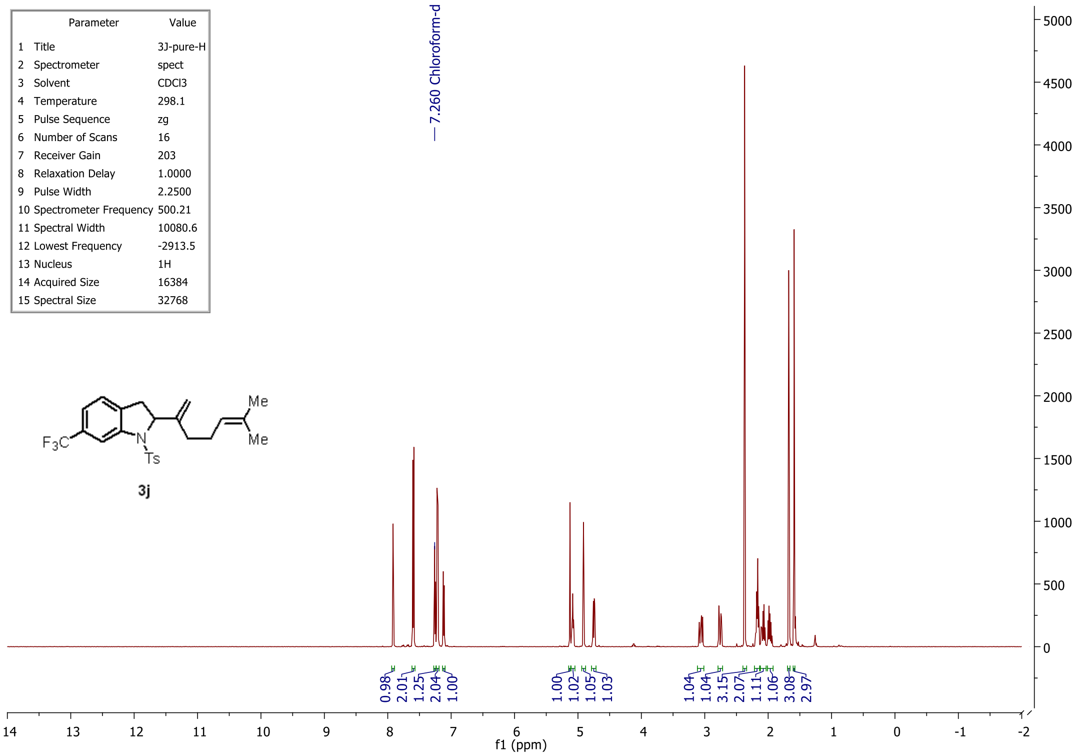

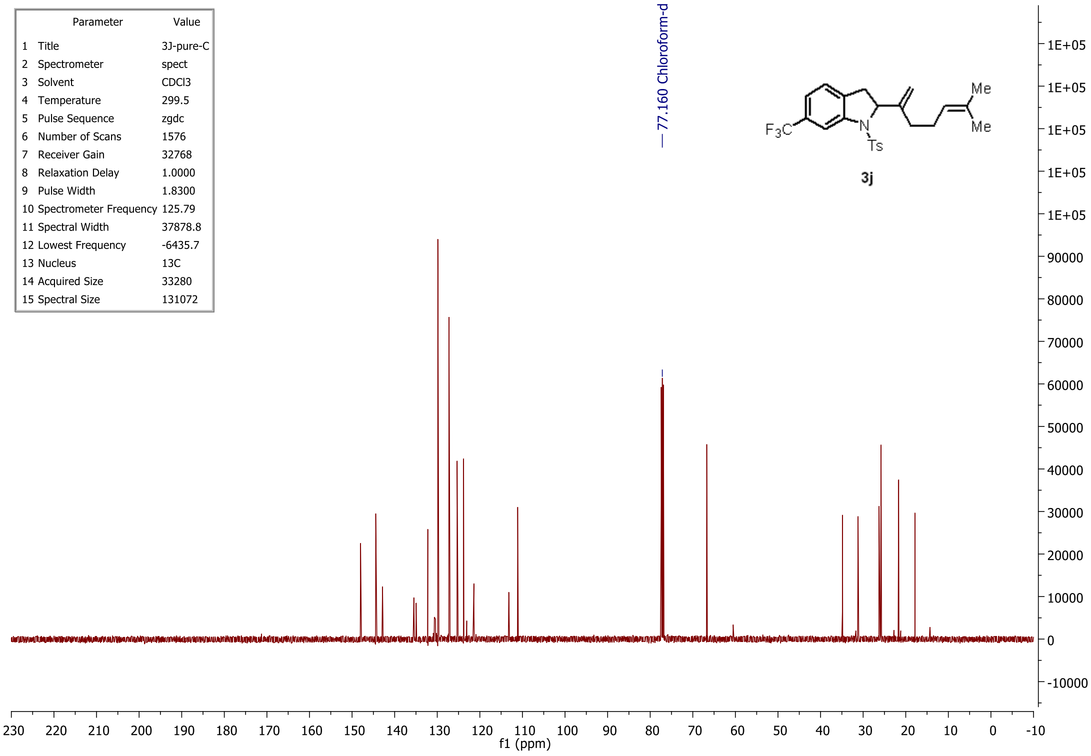

4-Methyl-2-(6-methylhepta-1,5-dien-2-yl)-*N*-tosylindoline (3k)

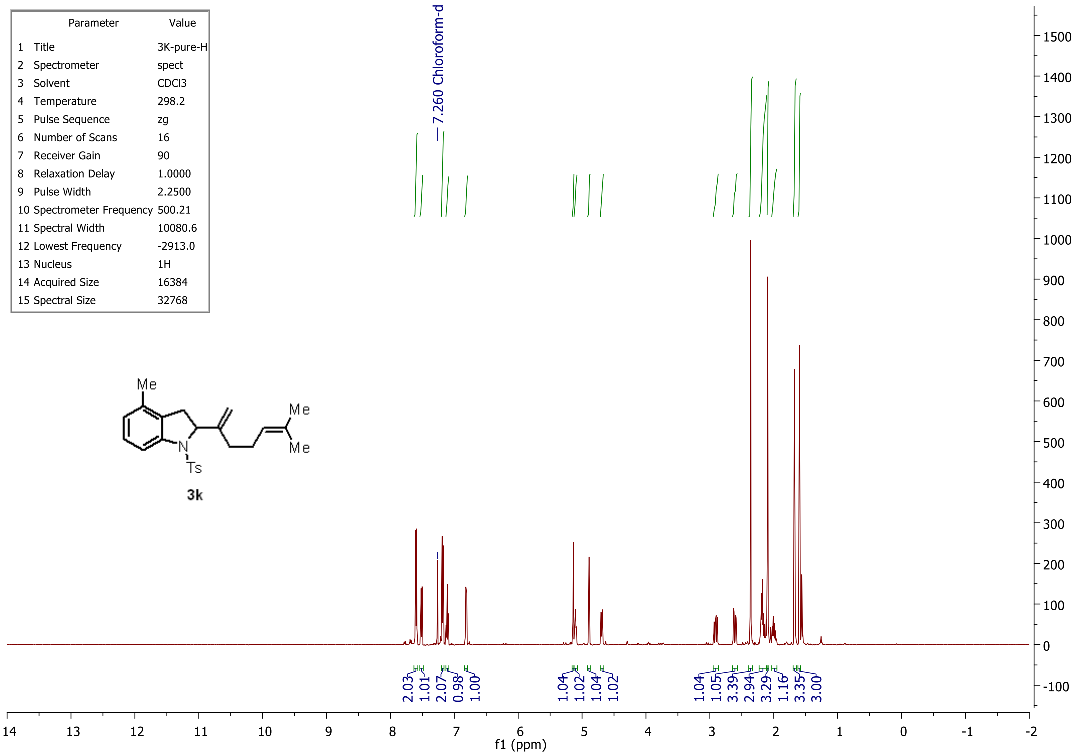

Parameter		Value
1	Title	3K-pure-H
2	Spectrometer	spect
3	Solvent	CDCl3
4	Temperature	298.2
5	Pulse Sequence	zg
6	Number of Scans	16
7	Receiver Gain	90
8	Relaxation Delay	1.0000
9	Pulse Width	2.2500
10	Spectrometer Frequency	500.21
11	Spectral Width	10080.6
12	Lowest Frequency	-2913.0
13	Nucleus	1H
14	Acquired Size	16384
15	Spectral Size	32768

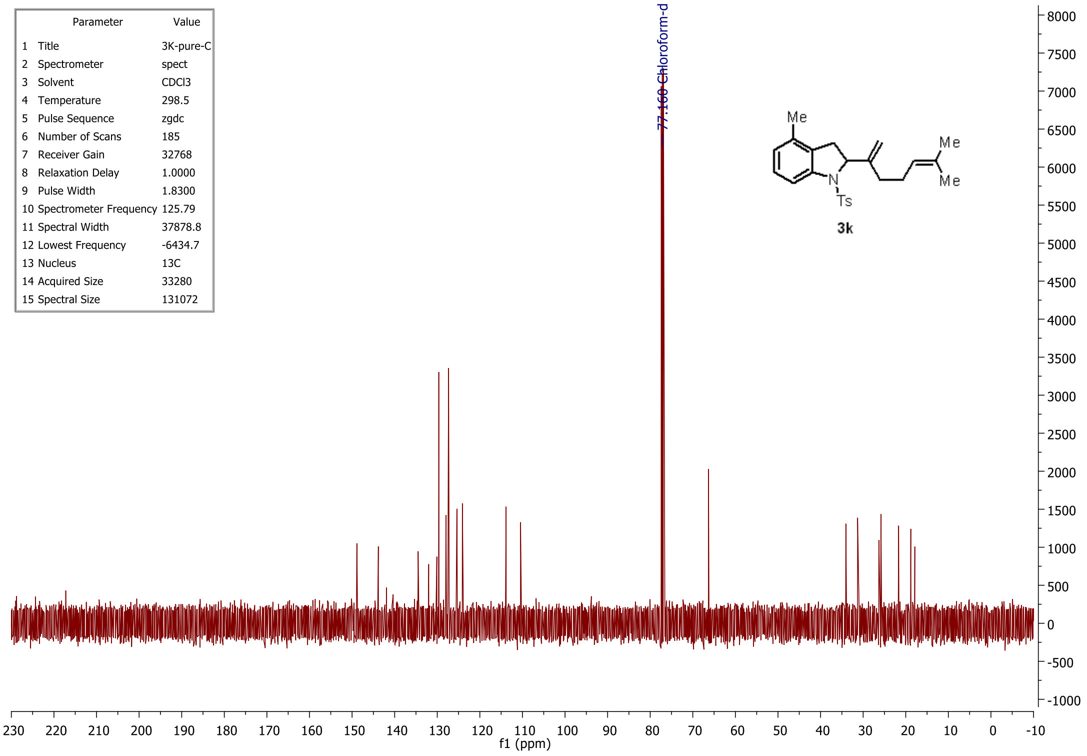

Parameter		Value
1	Title	3K-pure-C
2	Spectrometer	spect
3	Solvent	CDCl3
4	Temperature	298.5
5	Pulse Sequence	zgdc
6	Number of Scans	185
7	Receiver Gain	32768
8	Relaxation Delay	1.0000
9	Pulse Width	1.8300
10	Spectrometer Frequency	125.79
11	Spectral Width	37878.8
12	Lowest Frequency	-6434.7
13	Nucleus	13C
14	Acquired Size	33280
15	Spectral Size	131072

5,7-Dimethyl-2-(6-methylhepta-1,5-dien-2-yl)-1-tosylindoline (3l)

Parameter	Value
1 Title	3L-pure2-H
2 Spectrometer	spect
3 Solvent	CDCl3
4 Temperature	296.6
5 Pulse Sequence	zg
6 Number of Scans	16
7 Receiver Gain	406
8 Relaxation Delay	1.0000
9 Pulse Width	2.2500
10 Spectrometer Frequency	500.21
11 Spectral Width	10080.6
12 Lowest Frequency	-2913.5
13 Nucleus	1H
14 Acquired Size	16384
15 Spectral Size	32768

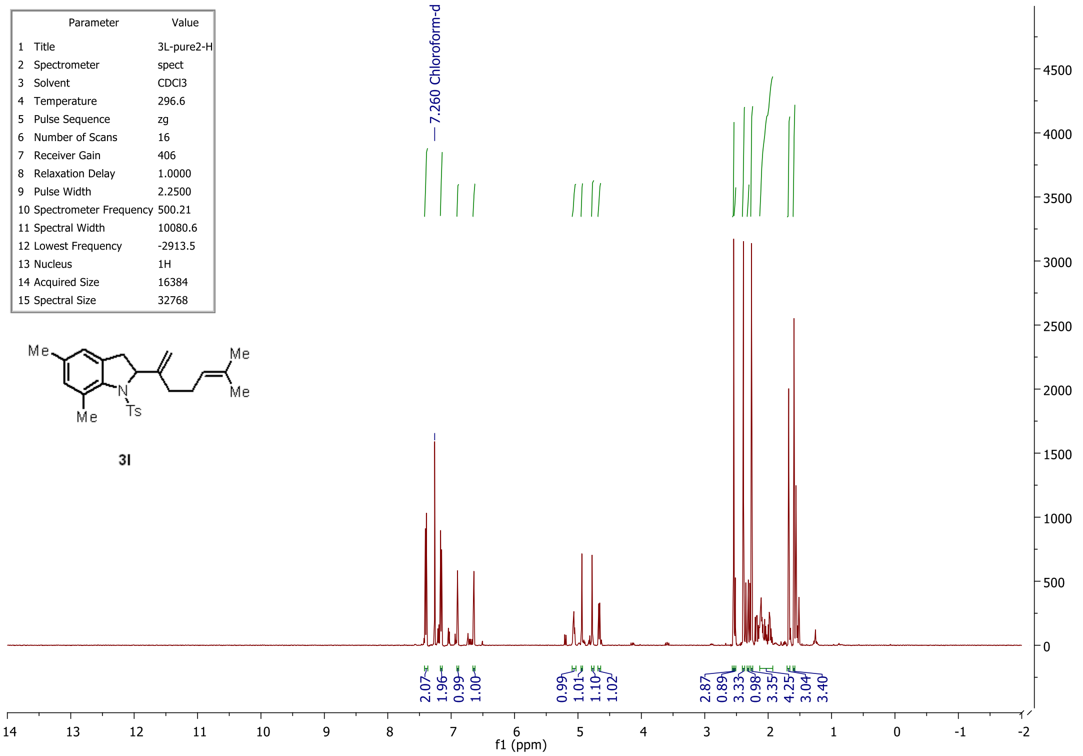

Parameter	Value
1 Title	3L-pure-C
2 Spectrometer	spect
3 Solvent	CDCl3
4 Temperature	299.3
5 Pulse Sequence	zgdc
6 Number of Scans	334
7 Receiver Gain	32768
8 Relaxation Delay	1.0000
9 Pulse Width	1.8300
10 Spectrometer Frequency	125.79
11 Spectral Width	37878.8
12 Lowest Frequency	-6435.2
13 Nucleus	13C
14 Acquired Size	33280
15 Spectral Size	131072

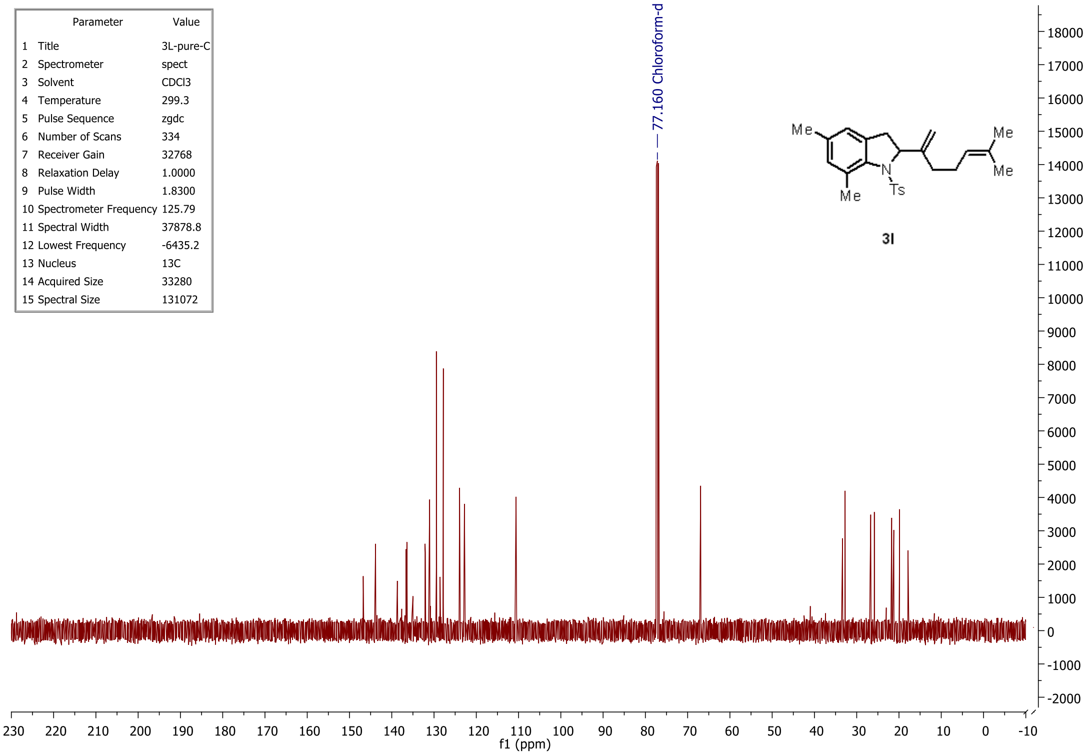

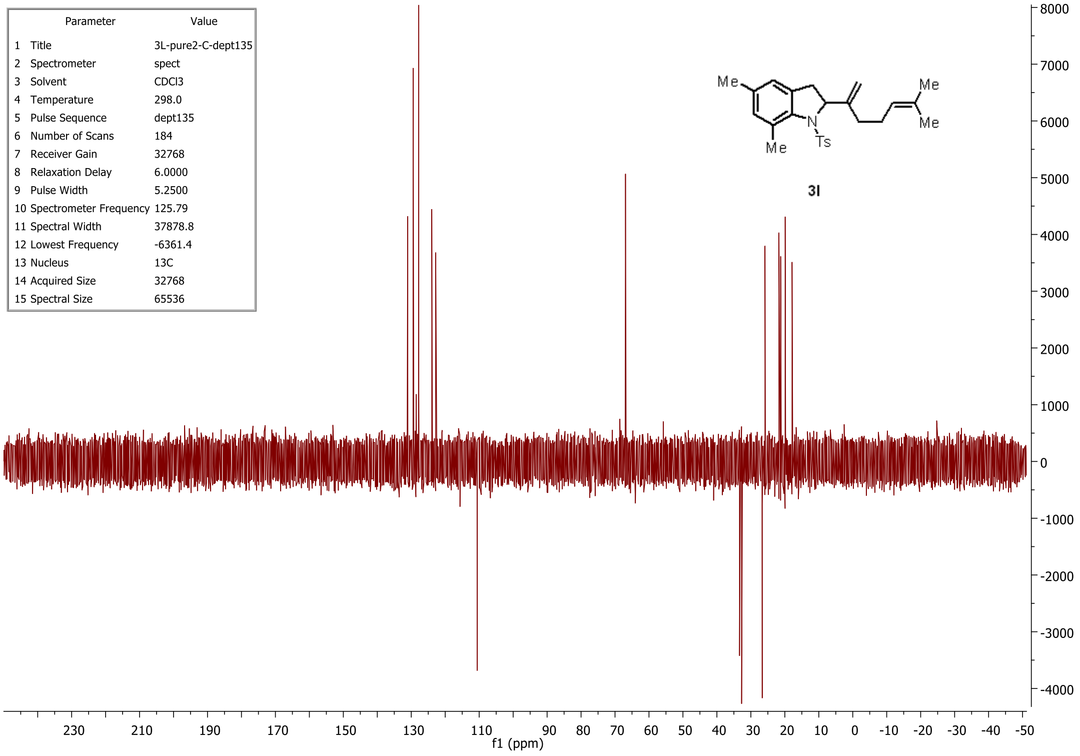

	Parameter	Value
1	Title	3L-pure2-C-dept135
2	Spectrometer	spect
3	Solvent	CDCl3
4	Temperature	298.0
5	Pulse Sequence	dept135
6	Number of Scans	184
7	Receiver Gain	32768
8	Relaxation Delay	6.0000
9	Pulse Width	5.2500
10	Spectrometer Frequency	125.79
11	Spectral Width	37878.8
12	Lowest Frequency	-6361.4
13	Nucleus	13C
14	Acquired Size	32768
15	Spectral Size	65536

Ethyl 2-(6-methylhepta-1,5-dien-2-yl)-*N*-tosylindoline-5-carboxylate (3m)

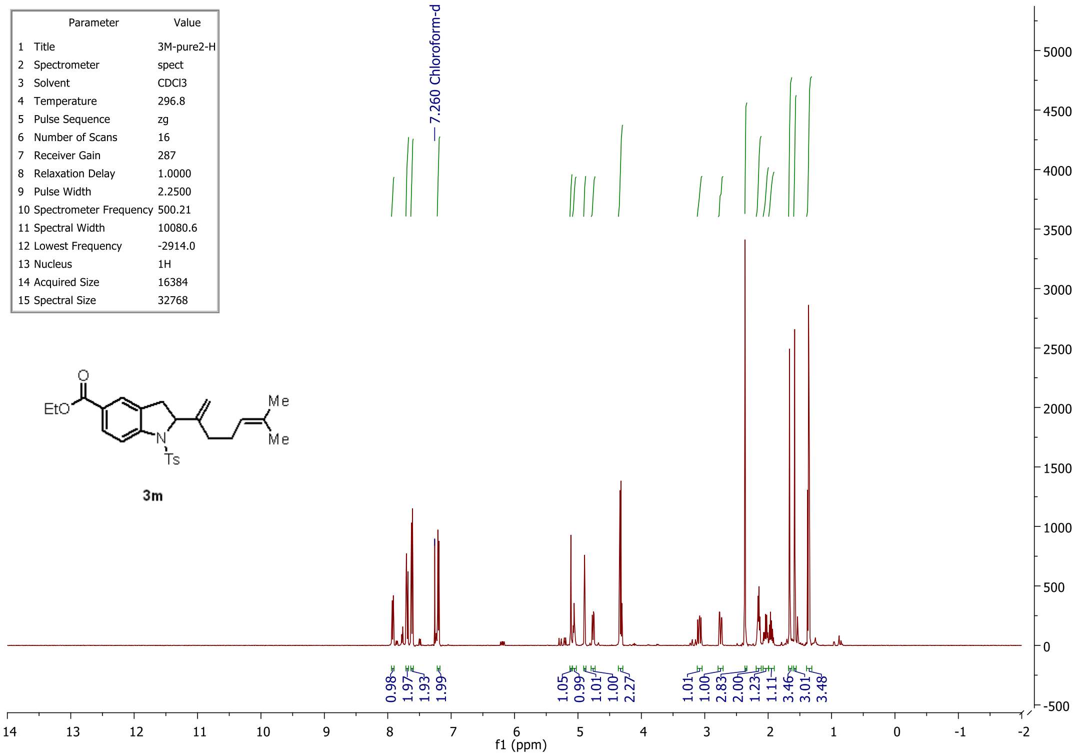

Parameter		Value
1	Title	3M-pure2-H
2	Spectrometer	spect
3	Solvent	CDCl3
4	Temperature	296.8
5	Pulse Sequence	zg
6	Number of Scans	16
7	Receiver Gain	287
8	Relaxation Delay	1.0000
9	Pulse Width	2.2500
10	Spectrometer Frequency	500.21
11	Spectral Width	10080.6
12	Lowest Frequency	-2914.0
13	Nucleus	1H
14	Acquired Size	16384
15	Spectral Size	32768

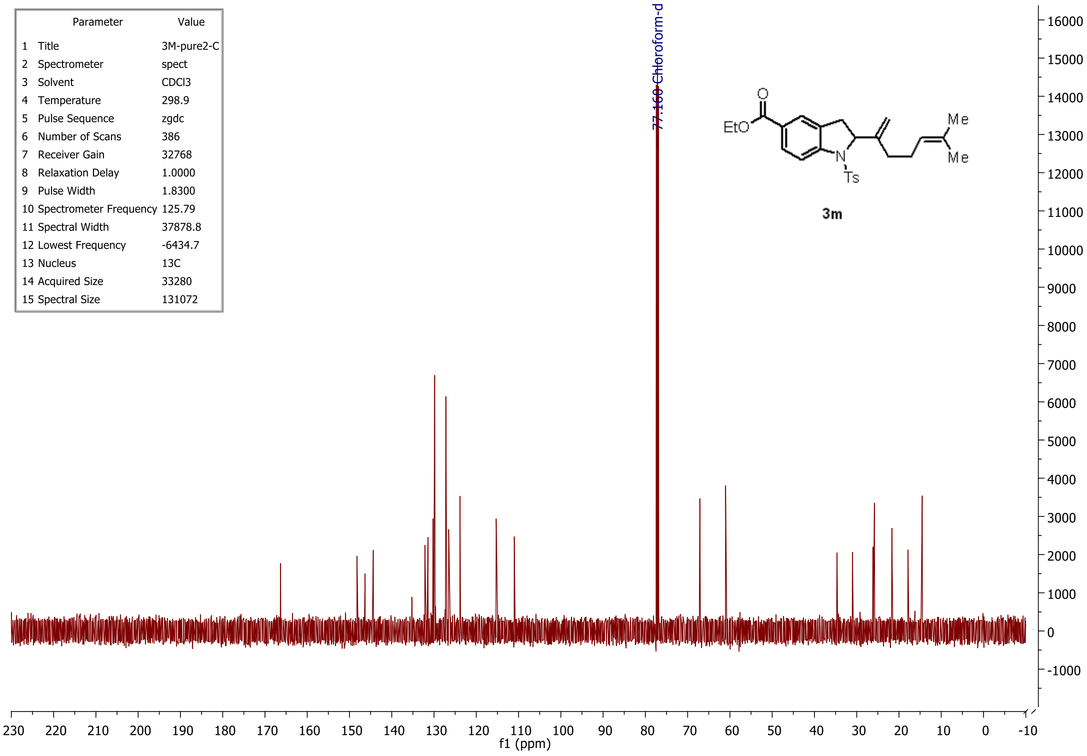

Parameter		Value
1	Title	3M-pure2-C
2	Spectrometer	spect
3	Solvent	CDCl3
4	Temperature	298.9
5	Pulse Sequence	zgdc
6	Number of Scans	386
7	Receiver Gain	32768
8	Relaxation Delay	1.0000
9	Pulse Width	1.8300
10	Spectrometer Frequency	125.79
11	Spectral Width	37878.8
12	Lowest Frequency	-6434.7
13	Nucleus	13C
14	Acquired Size	33280
15	Spectral Size	131072

1-(2-(6-Methylhepta-1,5-dien-2-yl)-*N*-tosylindolin-5-yl)ethan-1-one (3n)

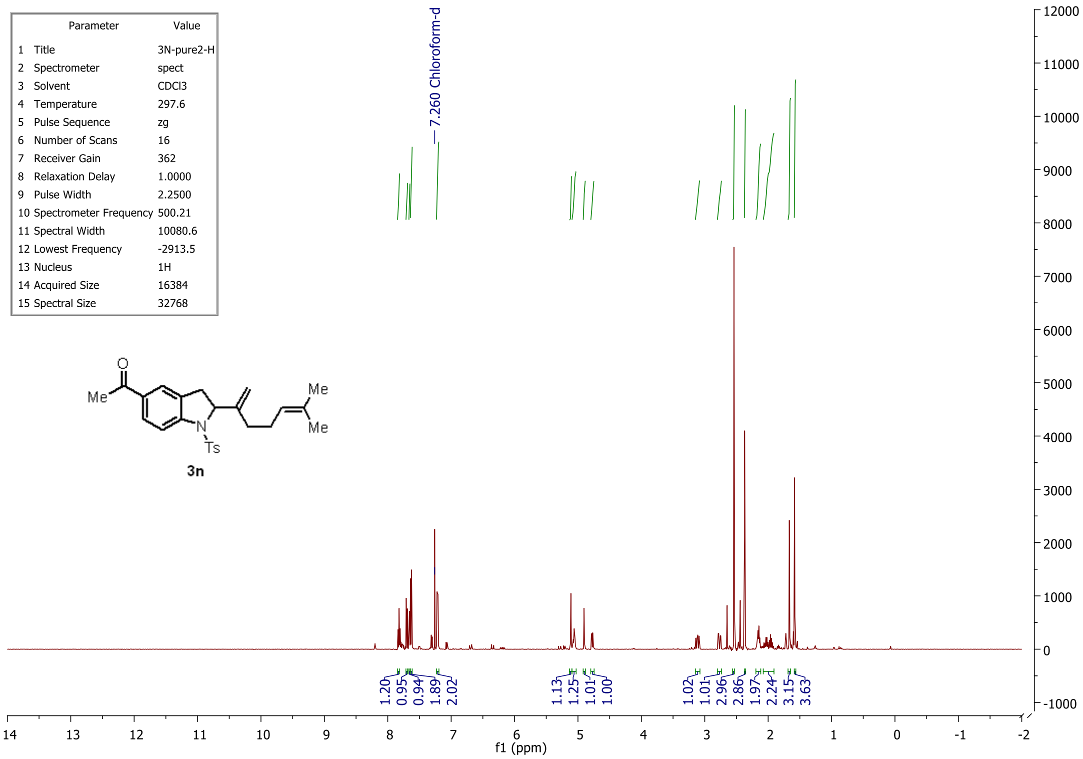

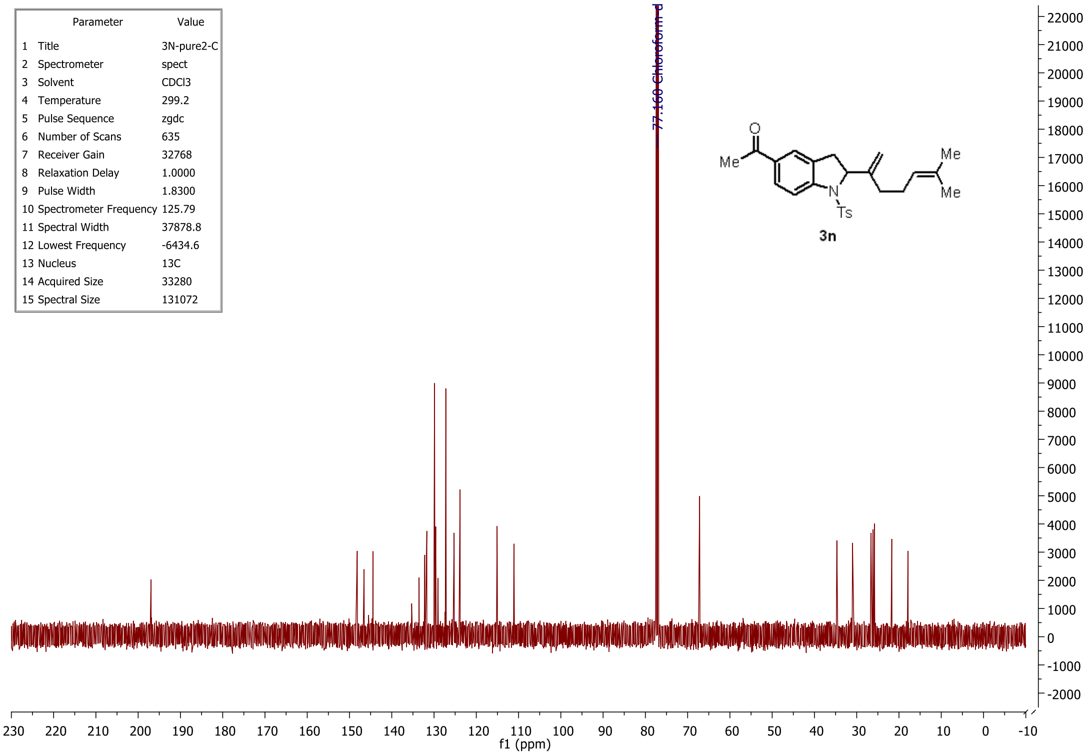

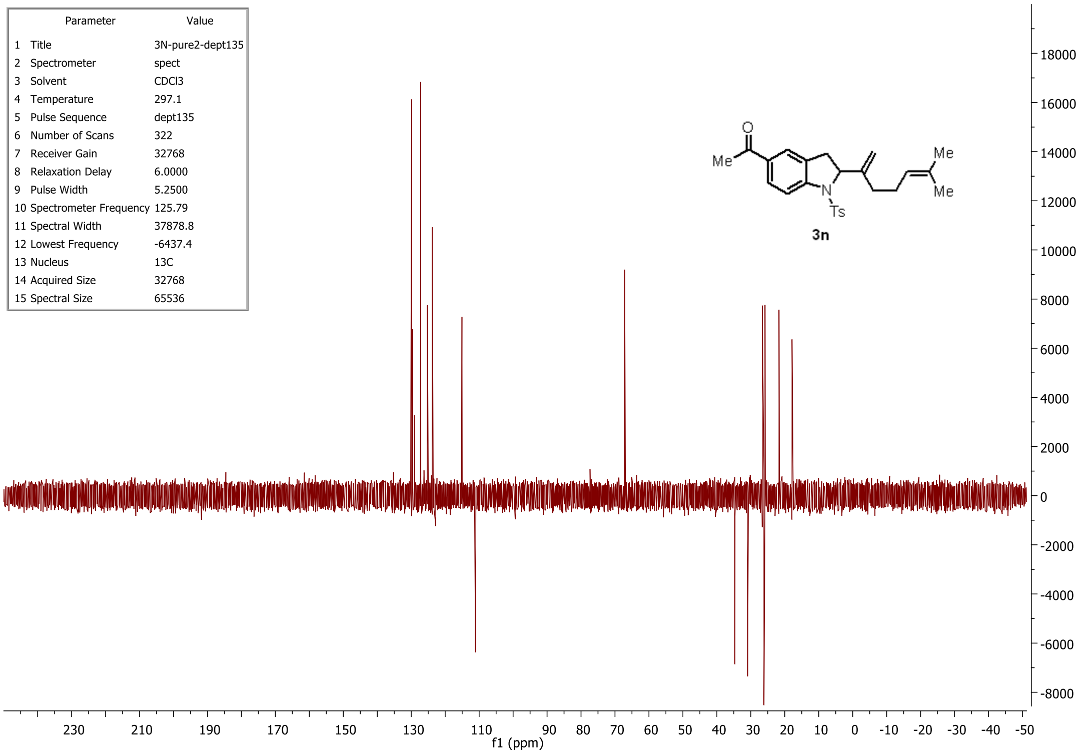
Parameter Value
1 Title 3N-pure2-dept135
2 Spectrometer spect
3 Solvent CDCl3
4 Temperature 297.1
5 Pulse Sequence dept135
6 Number of Scans 322
7 Receiver Gain 32768
8 Relaxation Delay 6.0000
9 Pulse Width 5.2500
10 Spectrometer Frequency 125.79
11 Spectral Width 37878.8
12 Lowest Frequency -6437.4
13 Nucleus 13C
14 Acquired Size 32768
15 Spectral Size 65536
O
Me
Me
N
Ts
Me
3n
18000
16000
14000
12000
10000
8000
6000
4000
2000
0
-2000
-4000
-6000
-8000
230 210 190 170 150 130 110 90 80 70 60 50 40 30 20 10 0 -10 -20 -30 -40 -50
f1 (ppm)

N,N-diisopropyl-2-(6-methylhepta-1,5-dien-2-yl)-*N*-tosylindoline-5-carboxamide (3o)

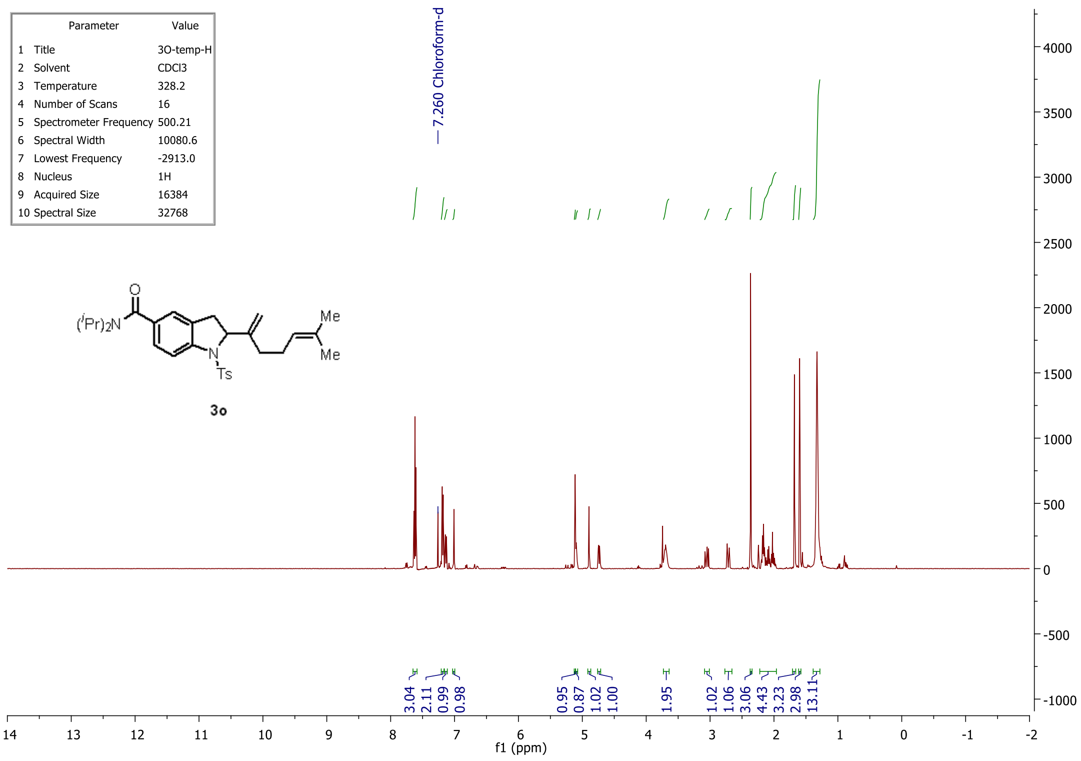

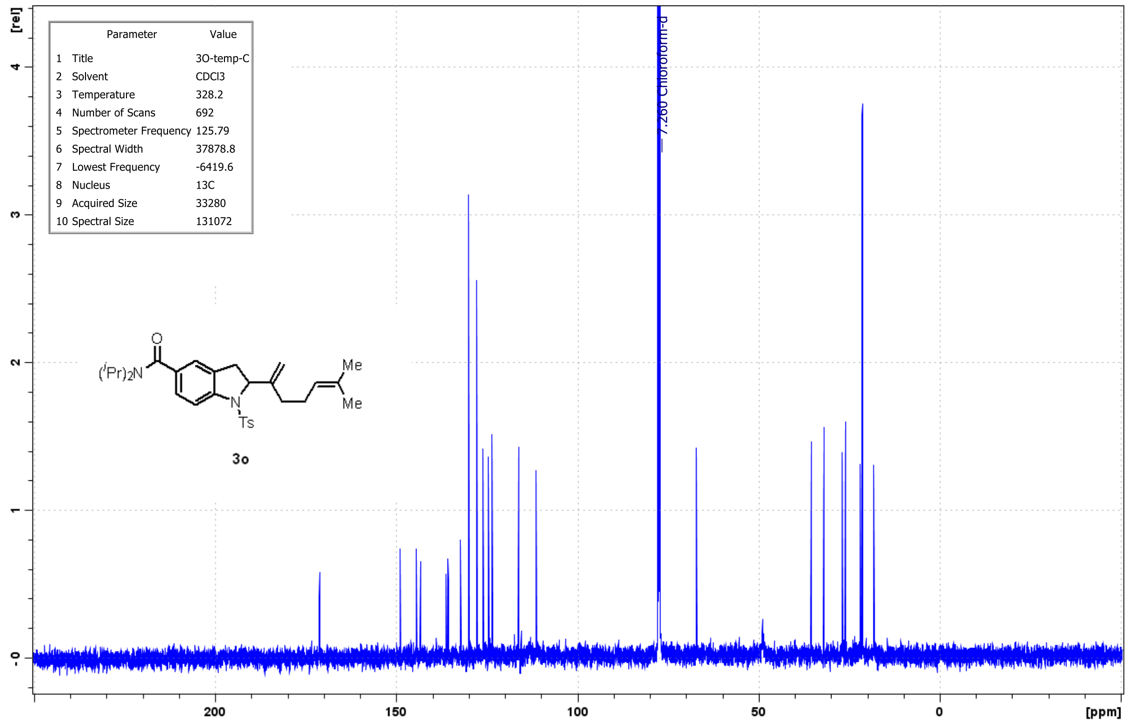

(*E*)-2-Styryl-*N*-tosylindoline (3ab)

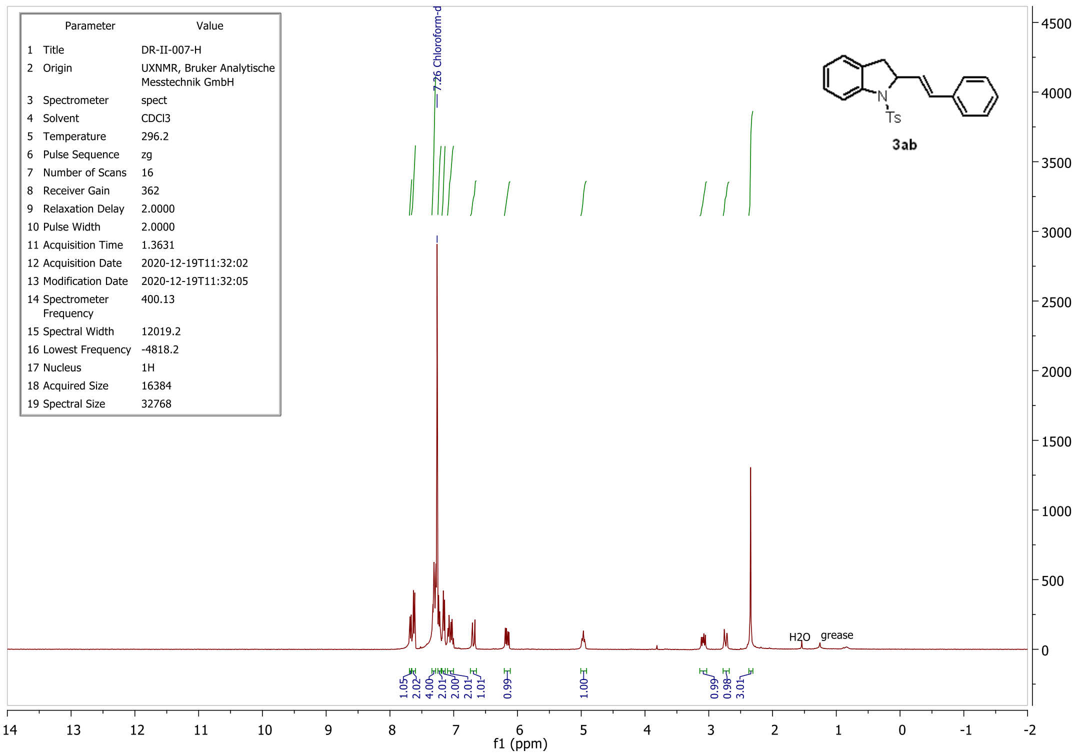

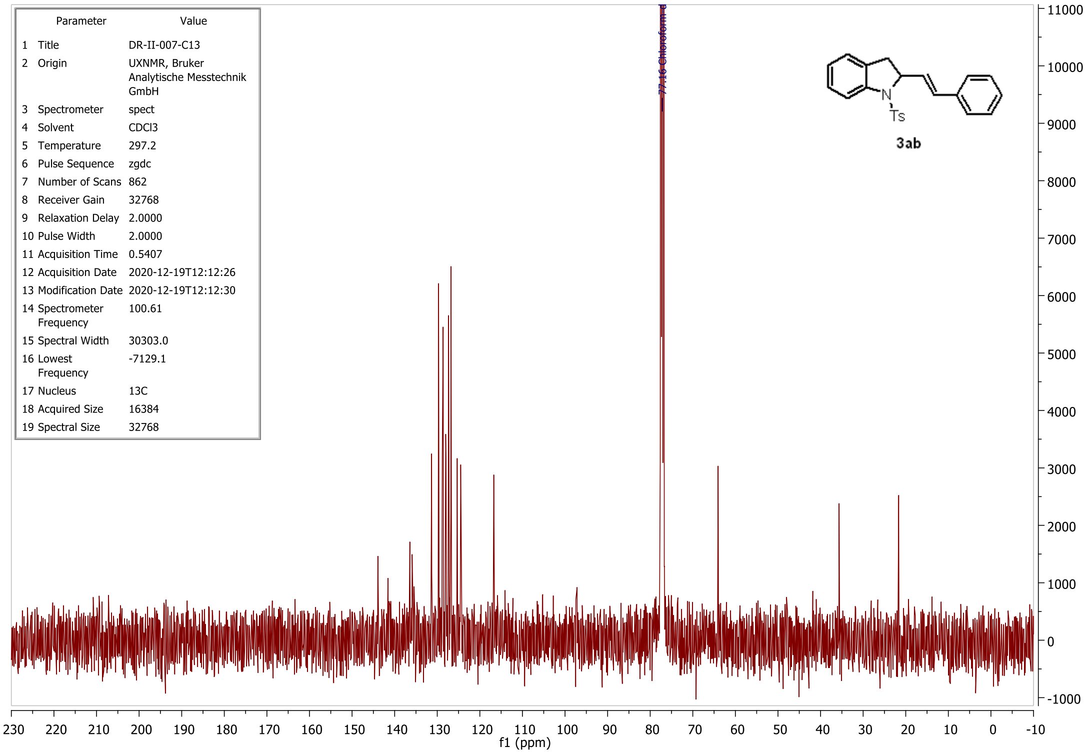

(*E*)-2-(4-Methoxystyryl)-*N*-tosylindoline (3ac)

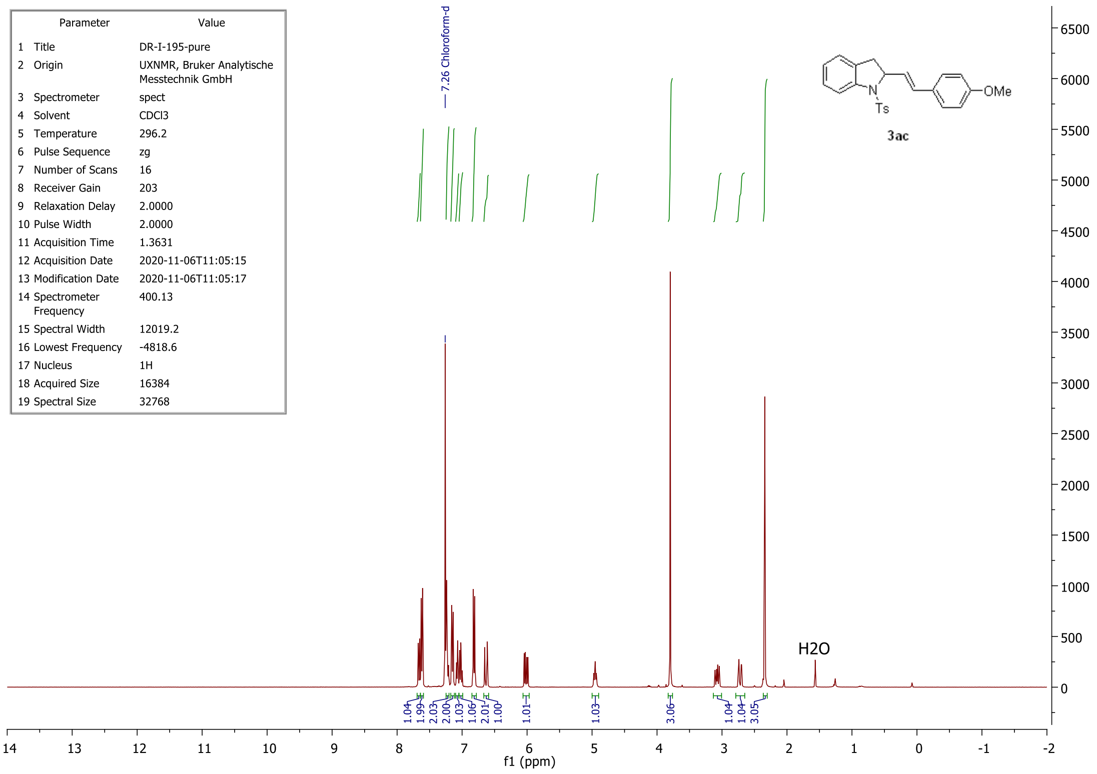

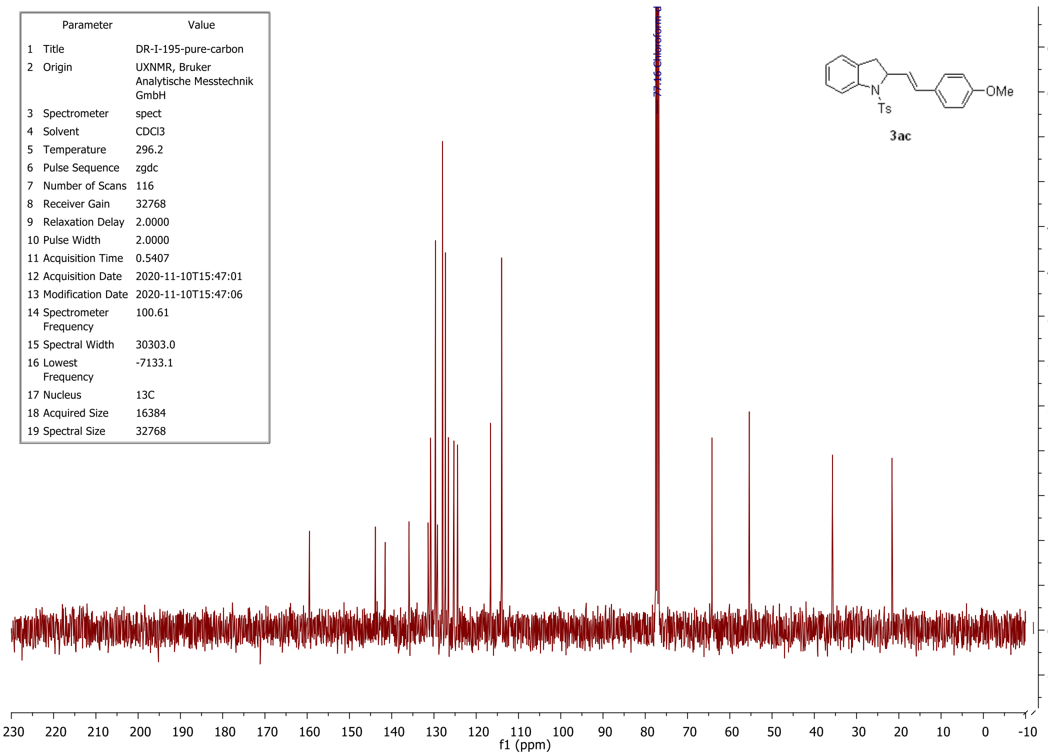

(*E*)-*N*-Tosyl-2-(3,4,5-trimethoxystyryl)indoline (3ad)

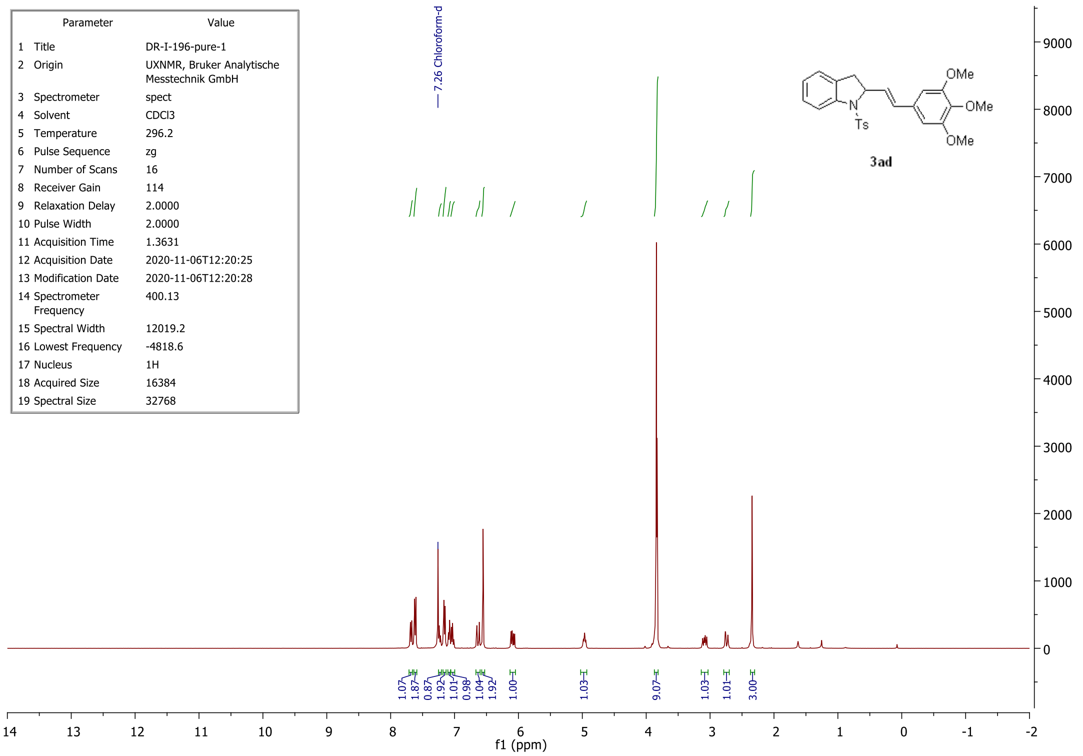

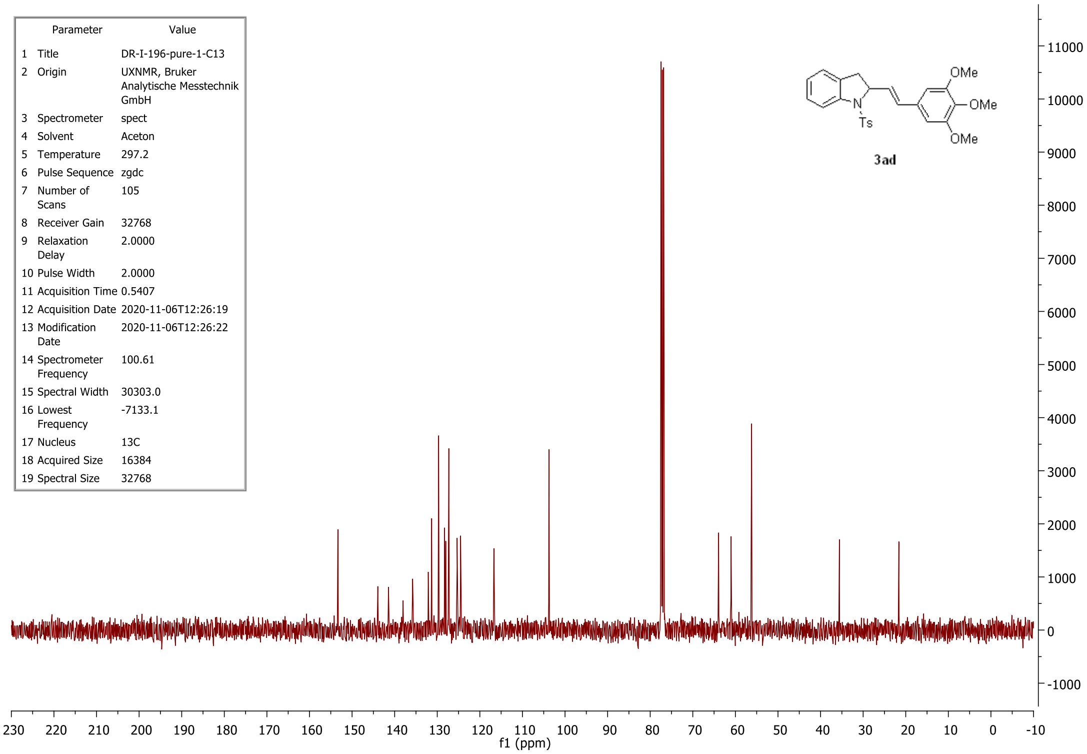

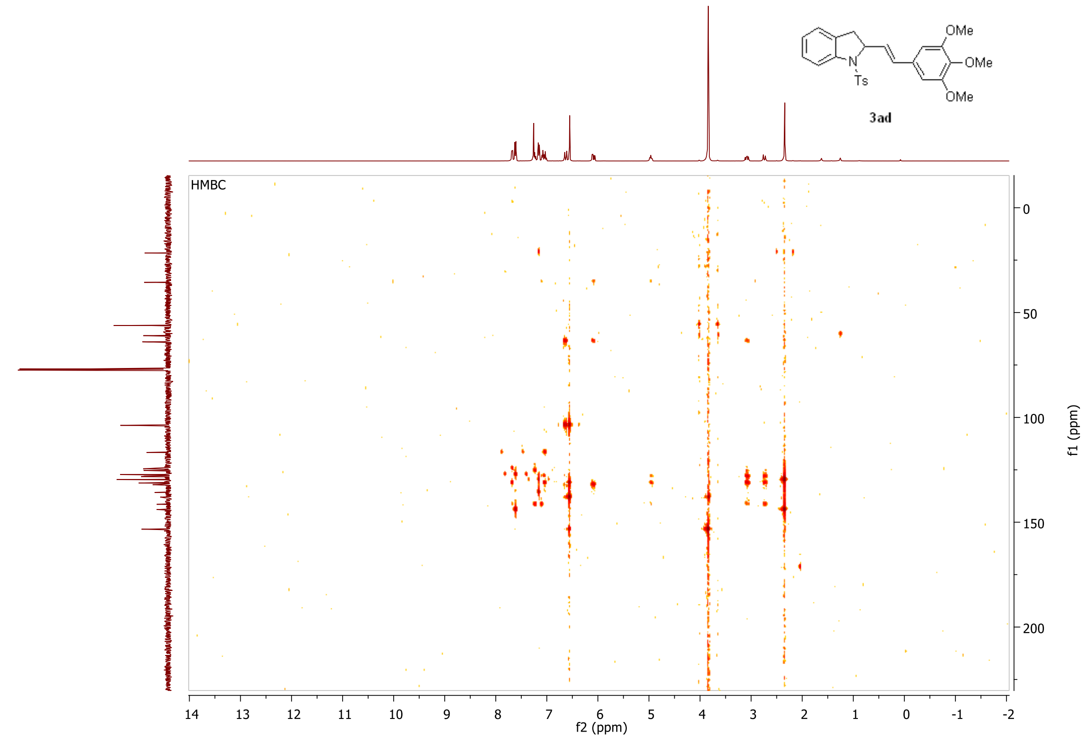
OMe
OMe
OMe
N
Ts
3ad
HMBC
f1 (ppm)
f2 (ppm)

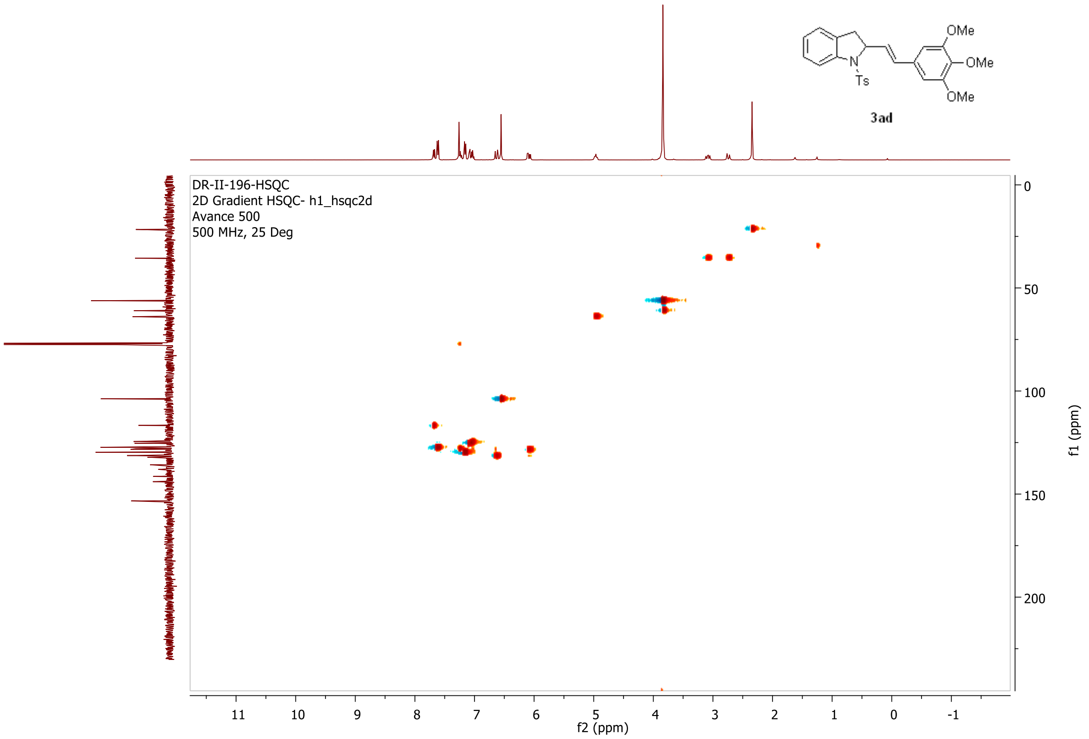
OMe
OMe
OMe
N
Ts
3ad
DR-II-196-HSQC
2D Gradient HSQC- h1_hsqc2d
Avance 500
500 MHz, 25 Deg
f1 (ppm)
f2 (ppm)

(*E*)-*N*-Tosyl-2-(3-(trifluoromethyl)styryl)indoline (3ae)

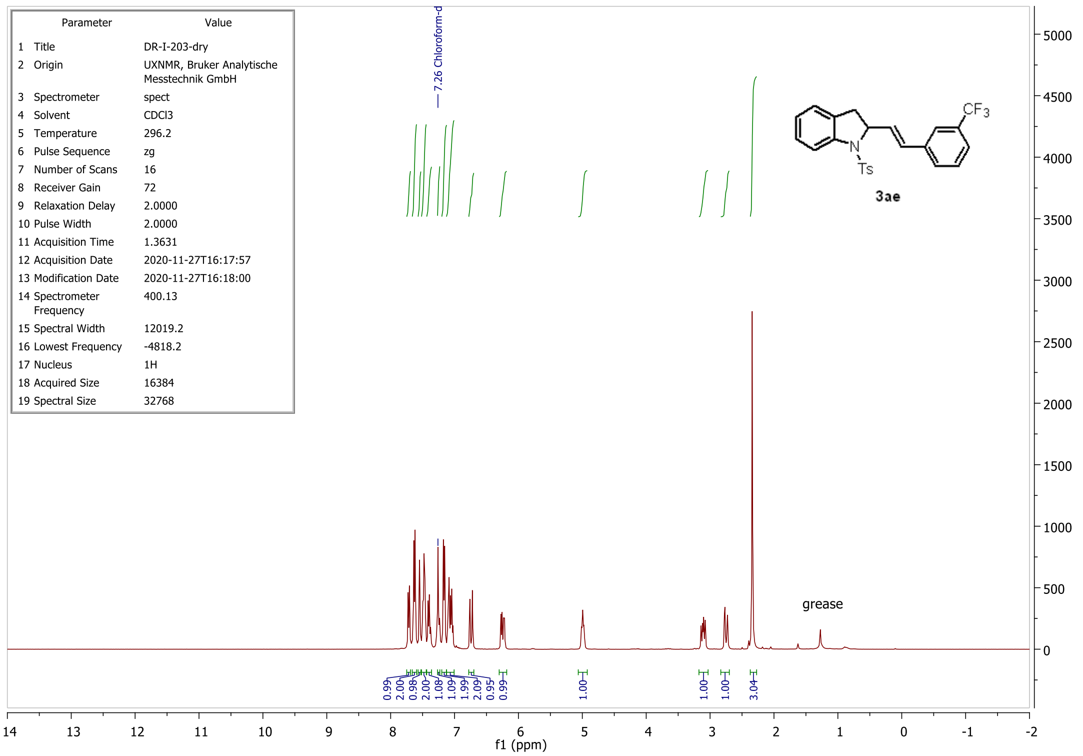

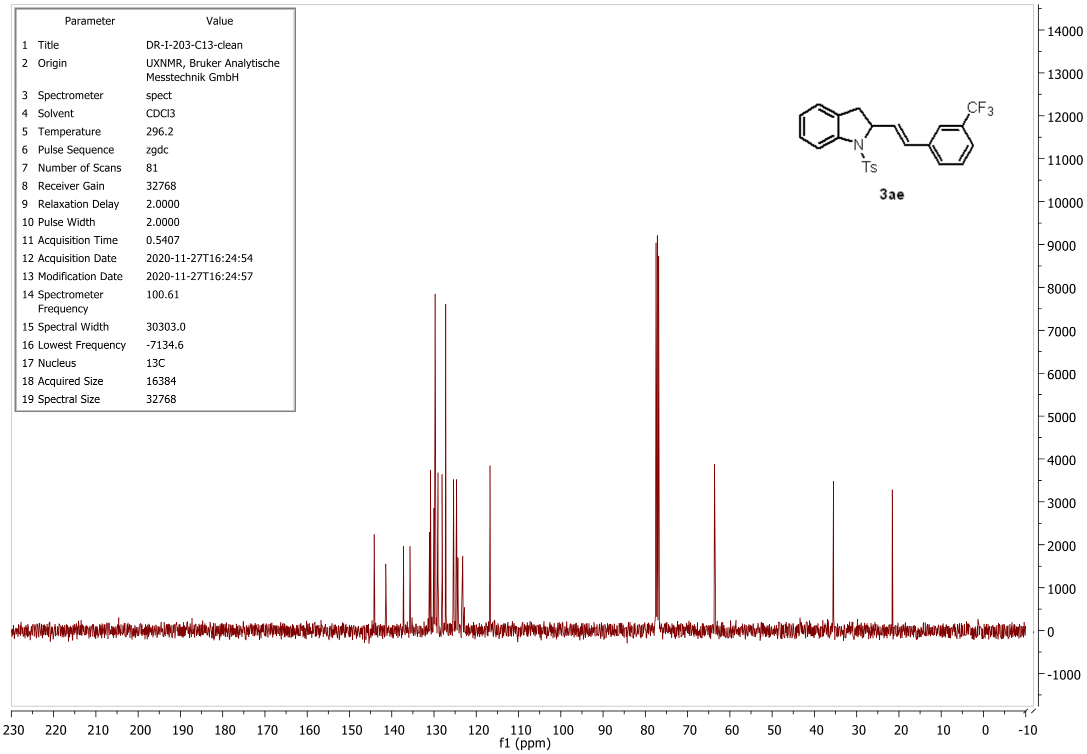

(*E*)-2-(2-(2,3-Dihydrobenzofuran-5-yl)vinyl)-*N*-tosylindoline (3af)

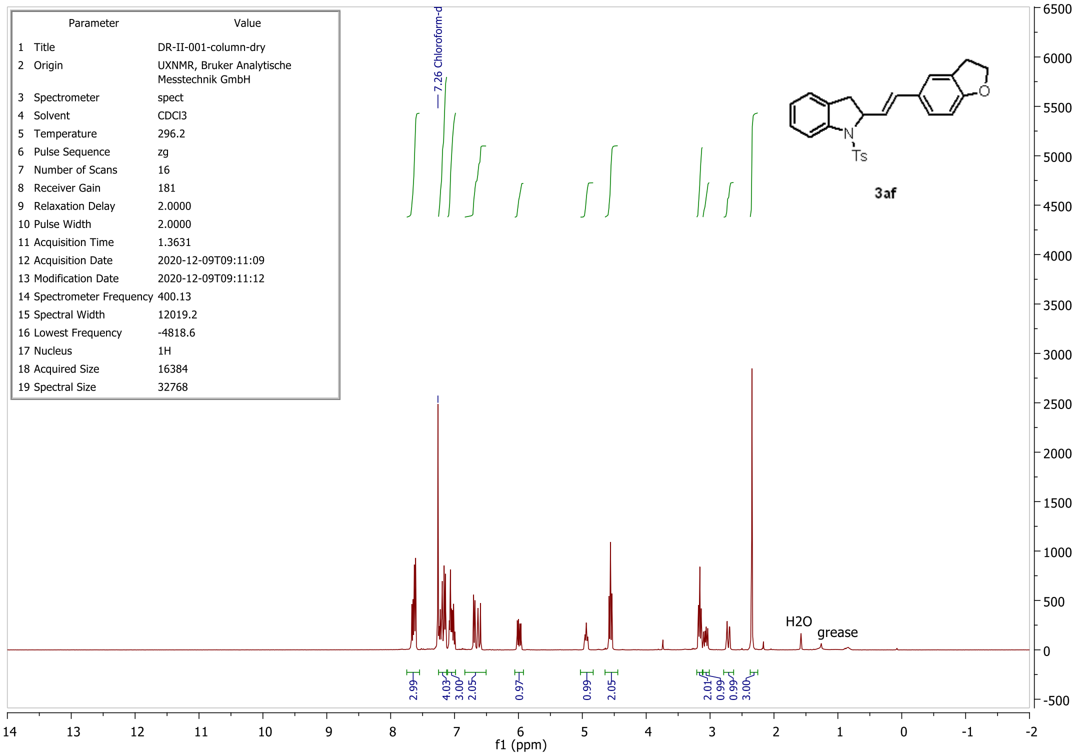

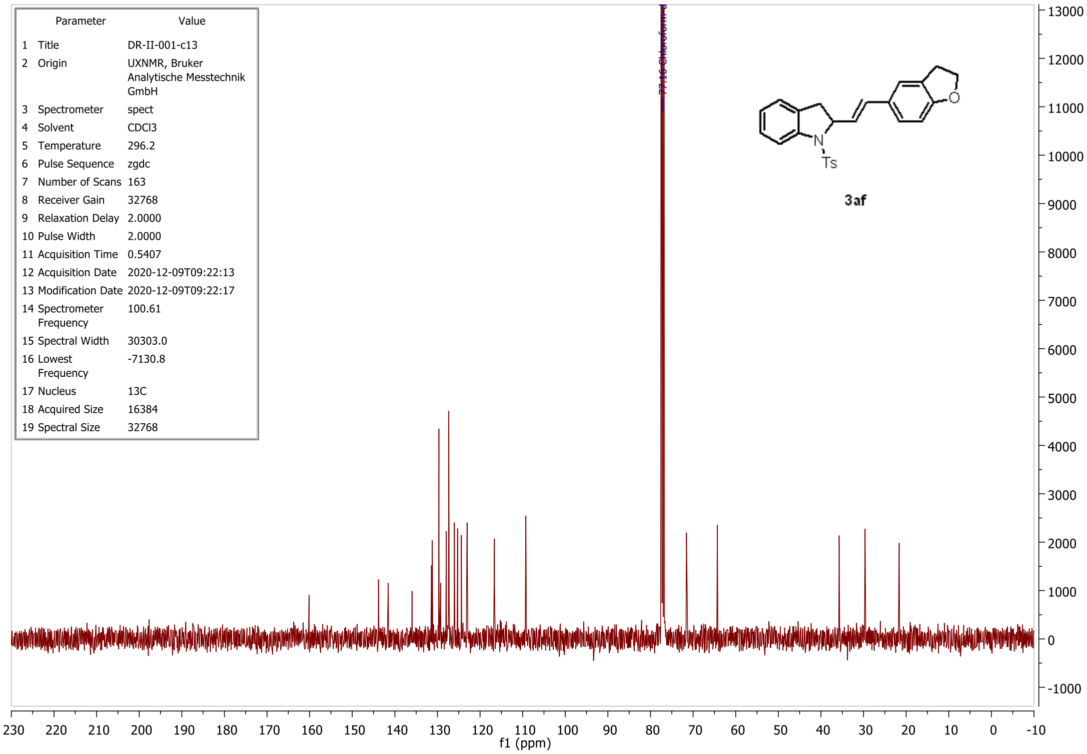

(*E*)-2-(2-(Thiophen-2-yl)vinyl)-*N*-tosylindoline (3ag)

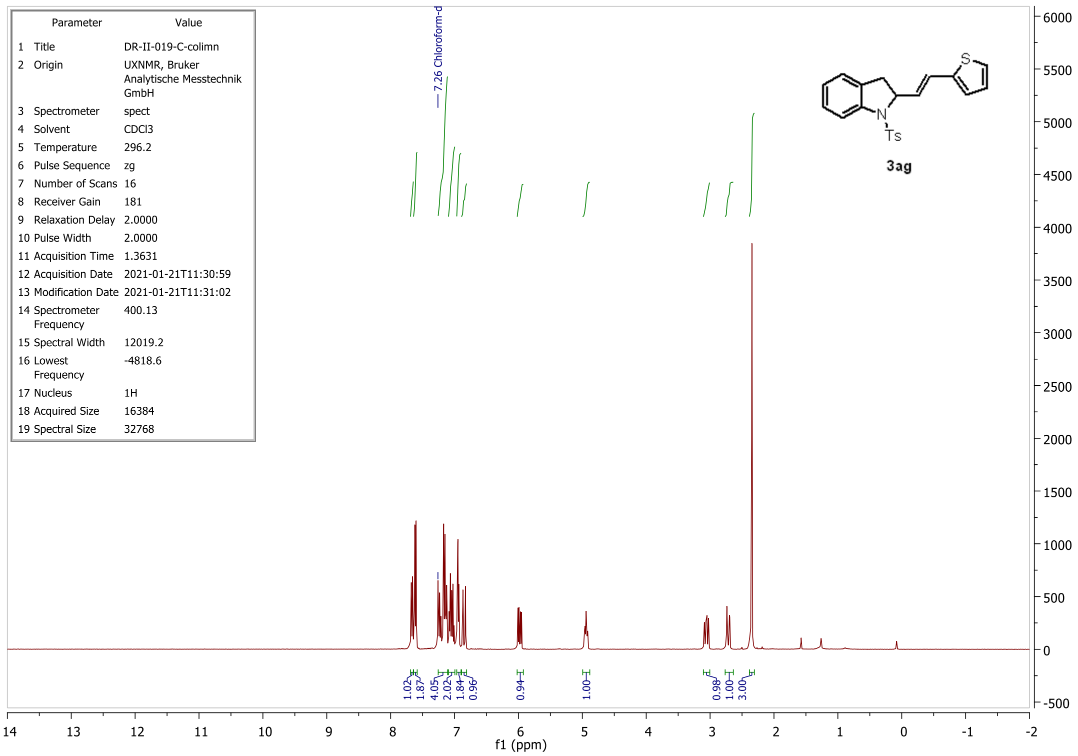

Parameter		Value
1	Title	DR-II-019-C-colimn
2	Origin	UXNMR, Bruker Analytische Messtechnik GmbH
3	Spectrometer	spect
4	Solvent	CDCl3
5	Temperature	296.2
6	Pulse Sequence	zg
7	Number of Scans	16
8	Receiver Gain	181
9	Relaxation Delay	2.0000
10	Pulse Width	2.0000
11	Acquisition Time	1.3631
12	Acquisition Date	2021-01-21T11:30:59
13	Modification Date	2021-01-21T11:31:02
14	Spectrometer Frequency	400.13
15	Spectral Width	12019.2
16	Lowest Frequency	-4818.6
17	Nucleus	1H
18	Acquired Size	16384
19	Spectral Size	32768

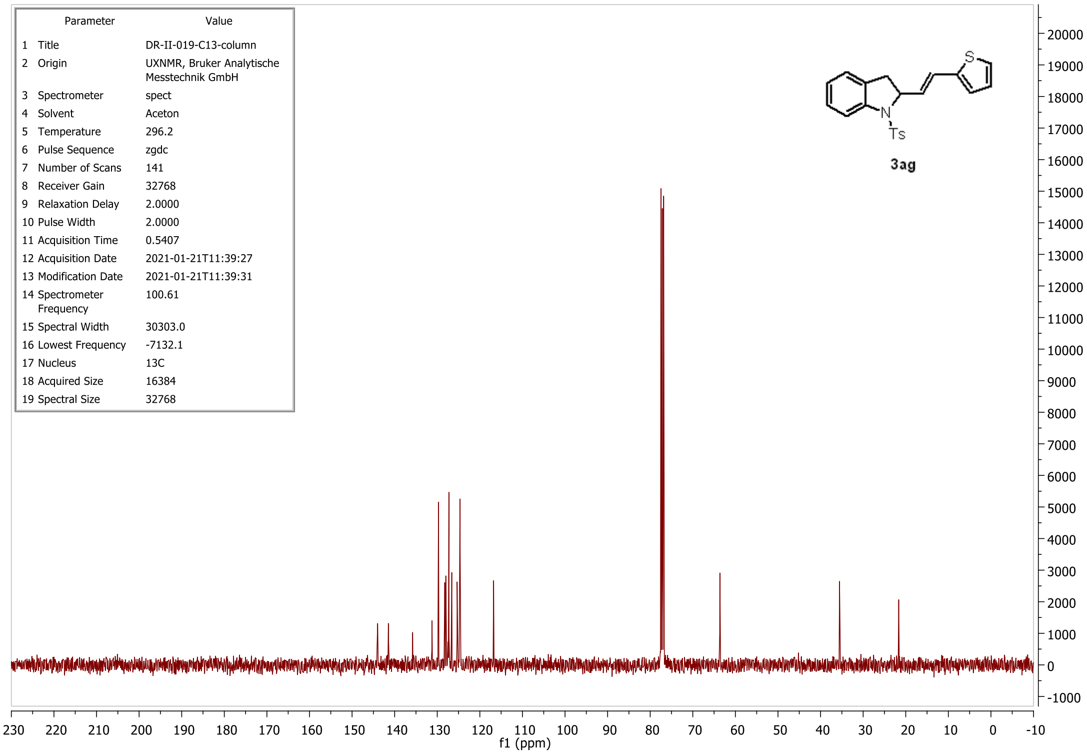

Parameter		Value
1	Title	DR-II-019-C13-column
2	Origin	UXNMR, Bruker Analytische Messtechnik GmbH
3	Spectrometer	spect
4	Solvent	Aceton
5	Temperature	296.2
6	Pulse Sequence	zgdc
7	Number of Scans	141
8	Receiver Gain	32768
9	Relaxation Delay	2.0000
10	Pulse Width	2.0000
11	Acquisition Time	0.5407
12	Acquisition Date	2021-01-21T11:39:27
13	Modification Date	2021-01-21T11:39:31
14	Spectrometer Frequency	100.61
15	Spectral Width	30303.0
16	Lowest Frequency	-7132.1
17	Nucleus	13C
18	Acquired Size	16384
19	Spectral Size	32768

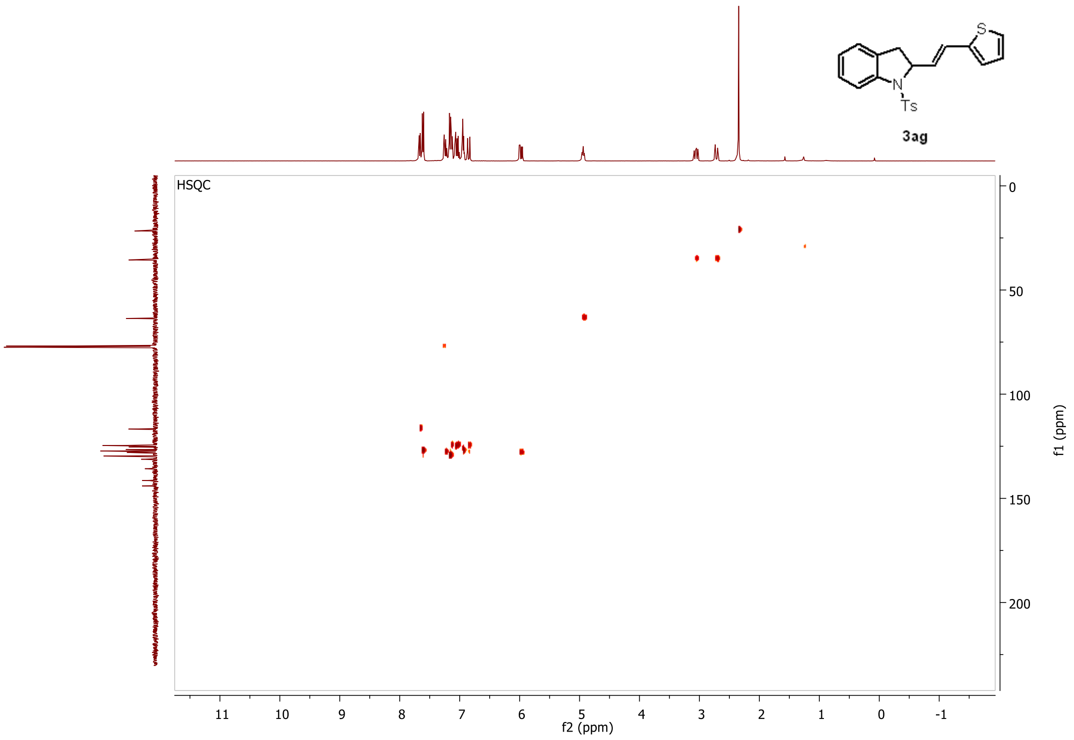

S
N
Ts
3ag
HSQC
0
50
100
150
200
f1 (ppm)
11
10
9
8
7
6
5
4
3
2
1
0
-1
f2 (ppm)

(*E*)-1-Me-3-(2-(*N*-tosylindolin-2-yl)vinyl)-1H-indole (3ah)

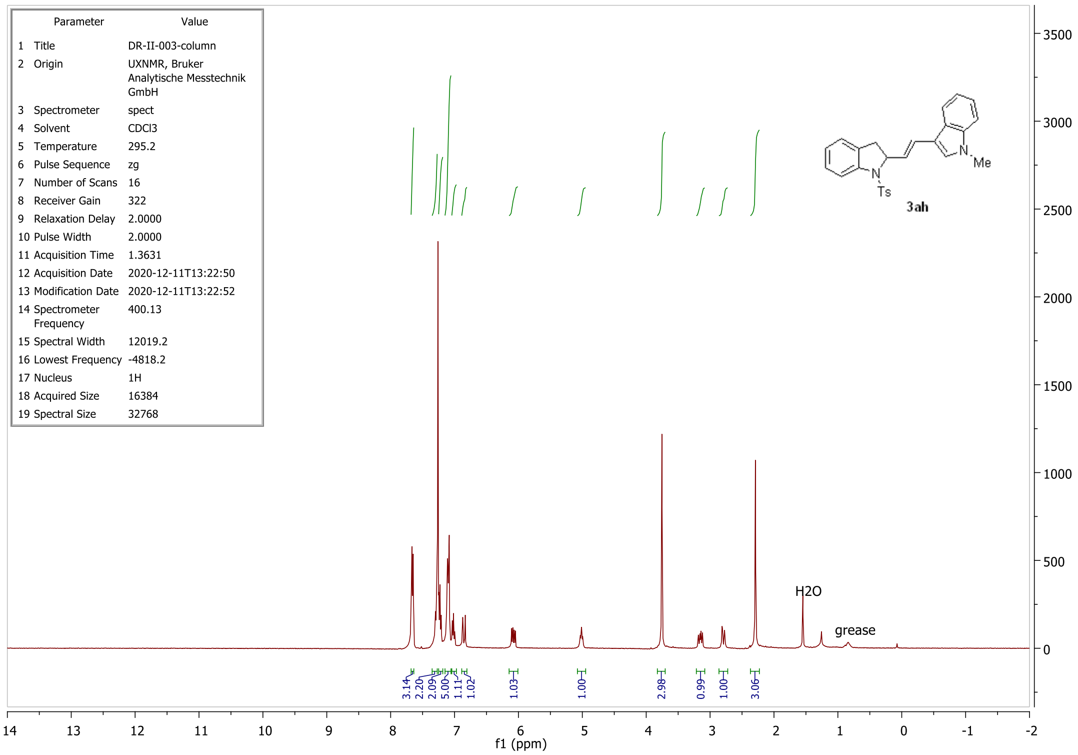

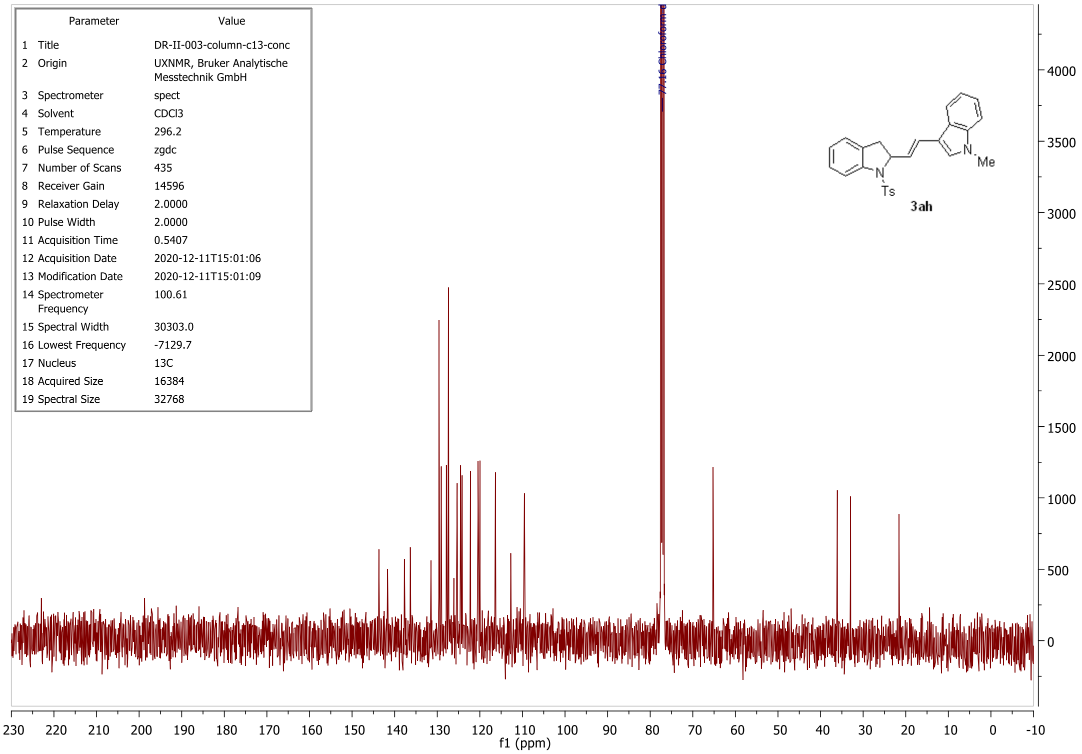

(*E*)-4-(*N*-Tosylindolin-2-yl)but-3-en-1-ol (3ai)

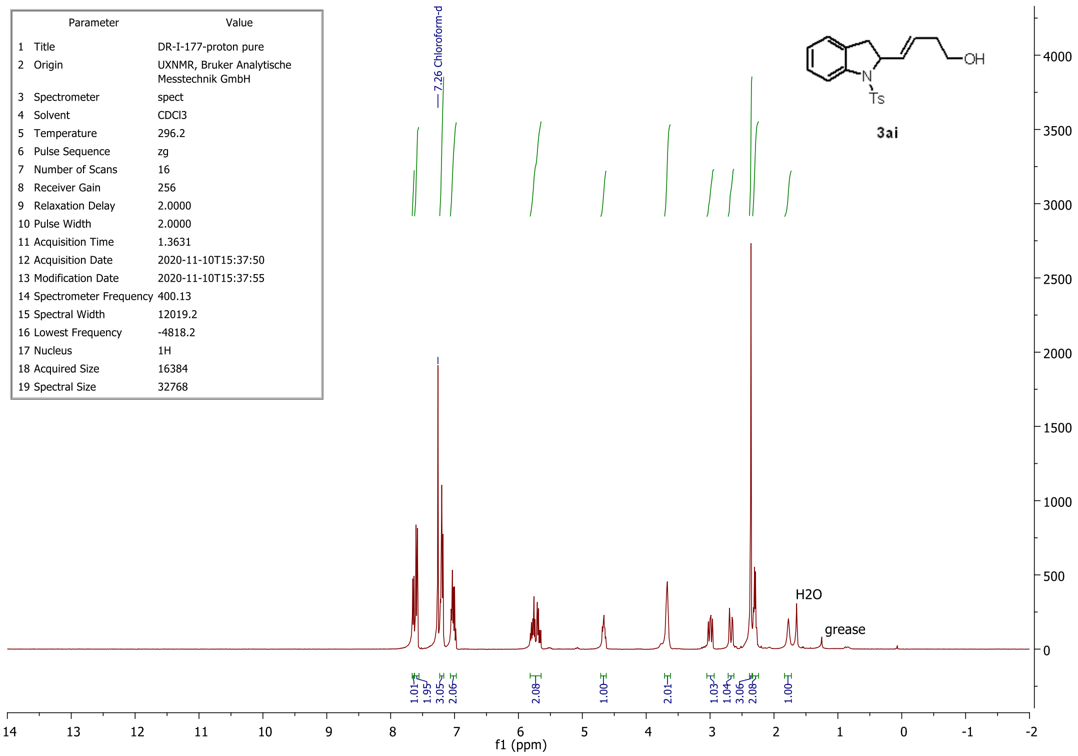

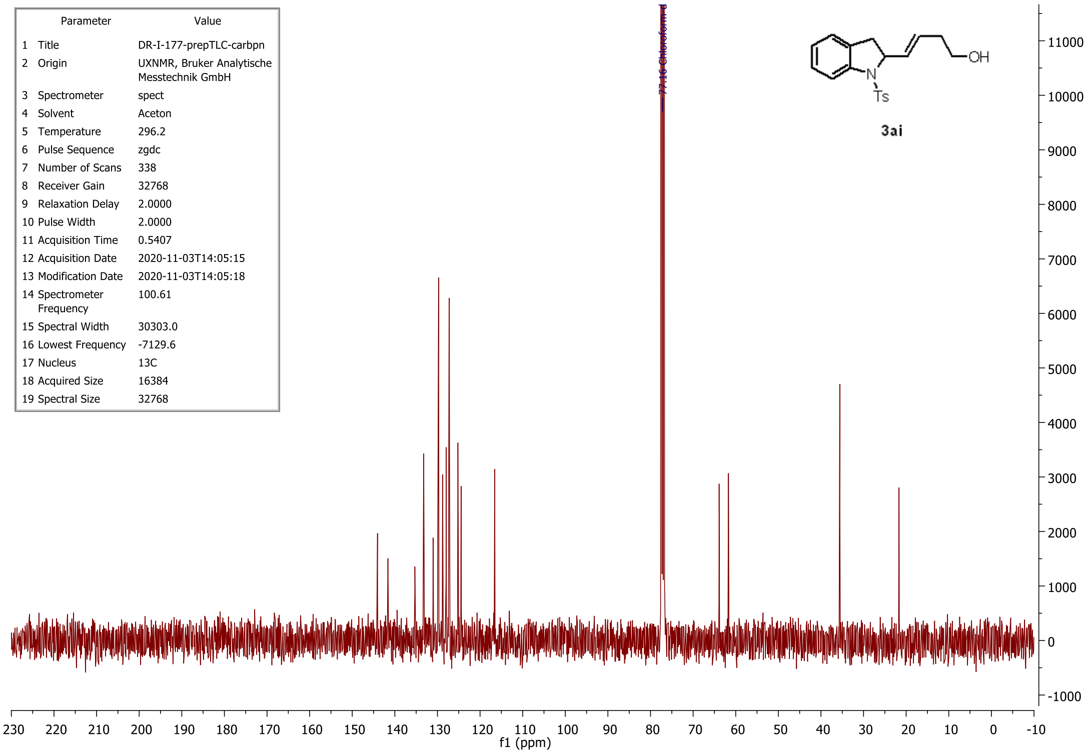

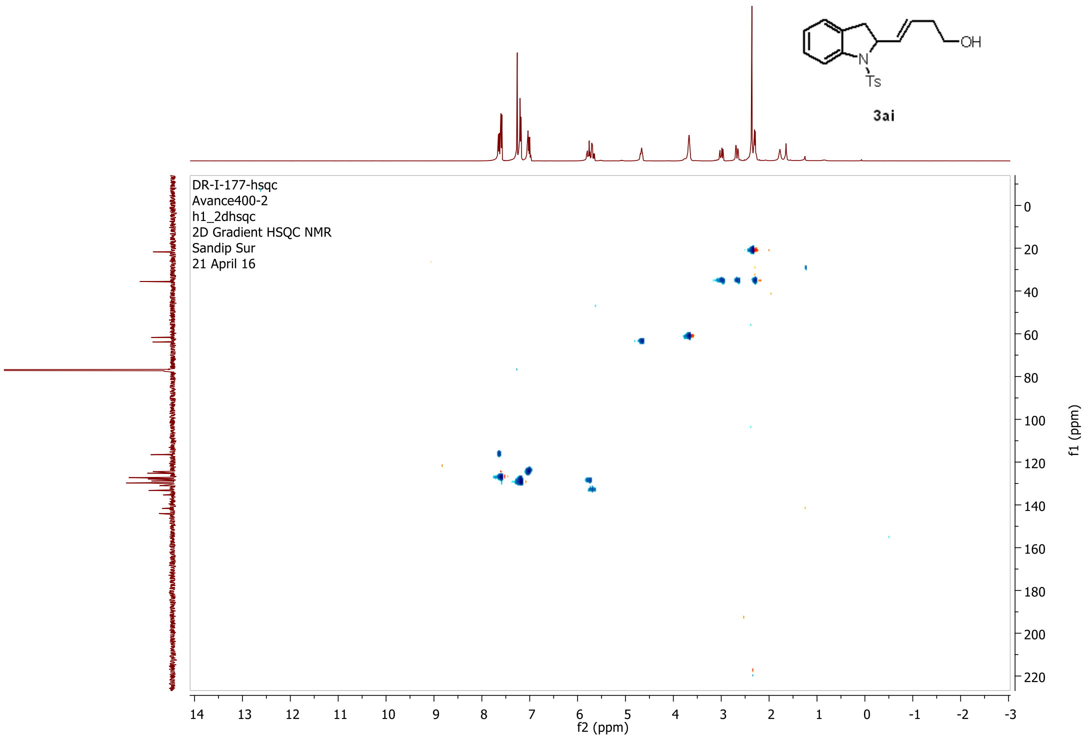
DR-I-177-hsqc
Avance400-2
h1_2dhsqc
2D Gradient HSQC NMR
Sandip Sur
21 April 16
N
Ts
OH
3ai
f2 (ppm)
f1 (ppm)

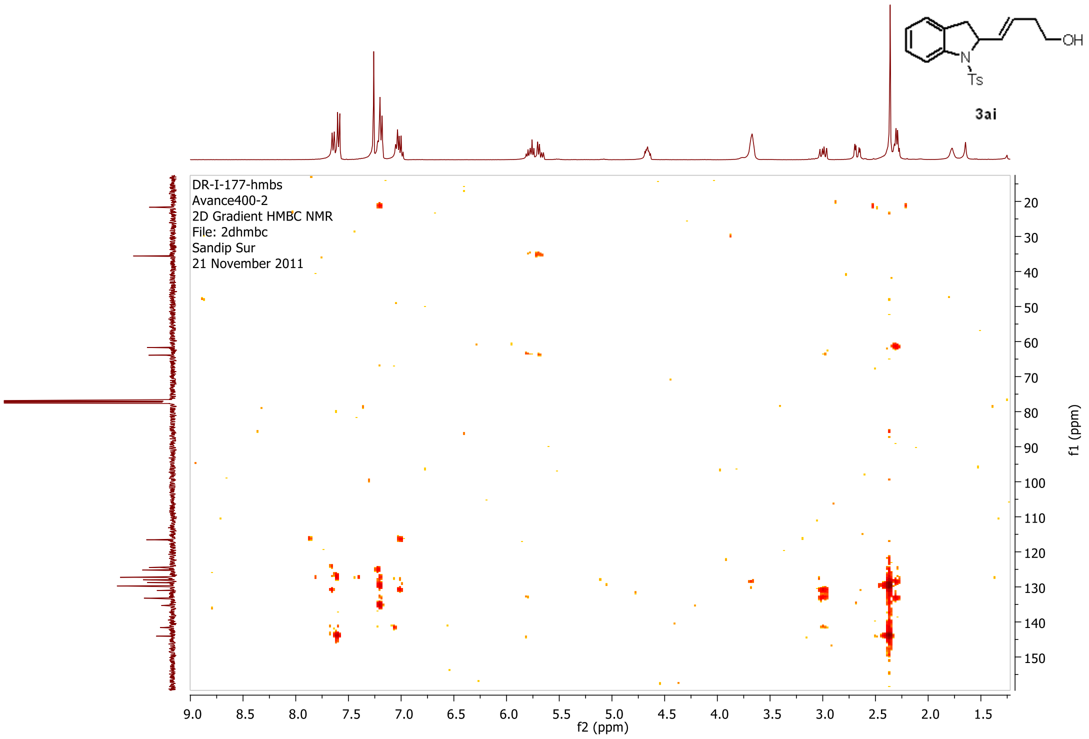
DR-I-177-hmbs
Avance400-2
2D Gradient HMBC NMR
File: 2dhmbc
Sandip Sur
21 November 2011
N
Ts
OH
3ai
f2 (ppm)
f1 (ppm)

(*E*)-2-(4-((*tert*-Butyldimethylsilyl)oxy)but-1-en-1-yl)-*N*-tosylindoline (3aj)

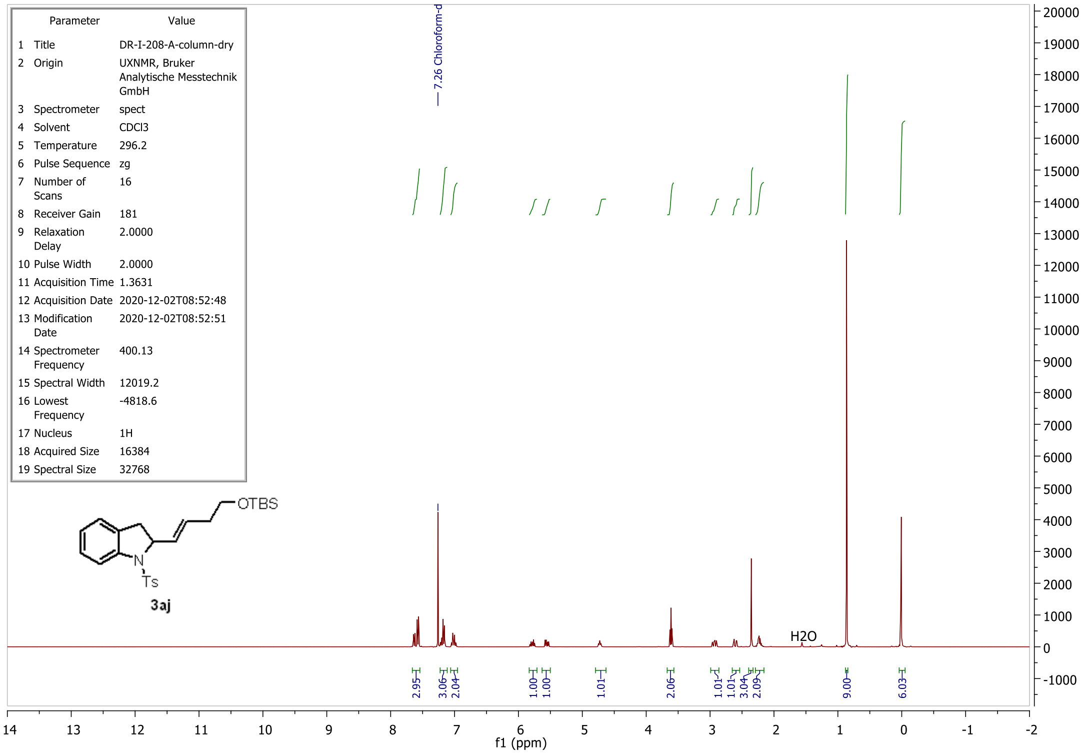

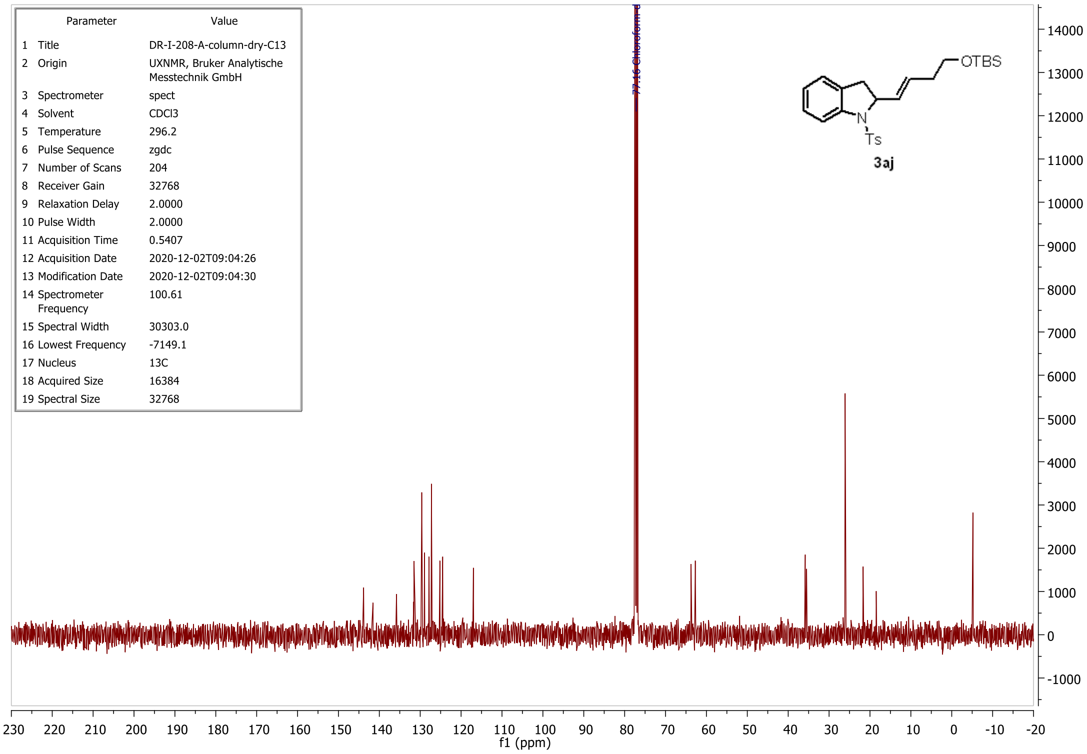

(*E*)-2-(4-(*N*-Tosylindolin-2-yl)but-3-en-1-yl)isoindoline-1,3-dione (3ak)

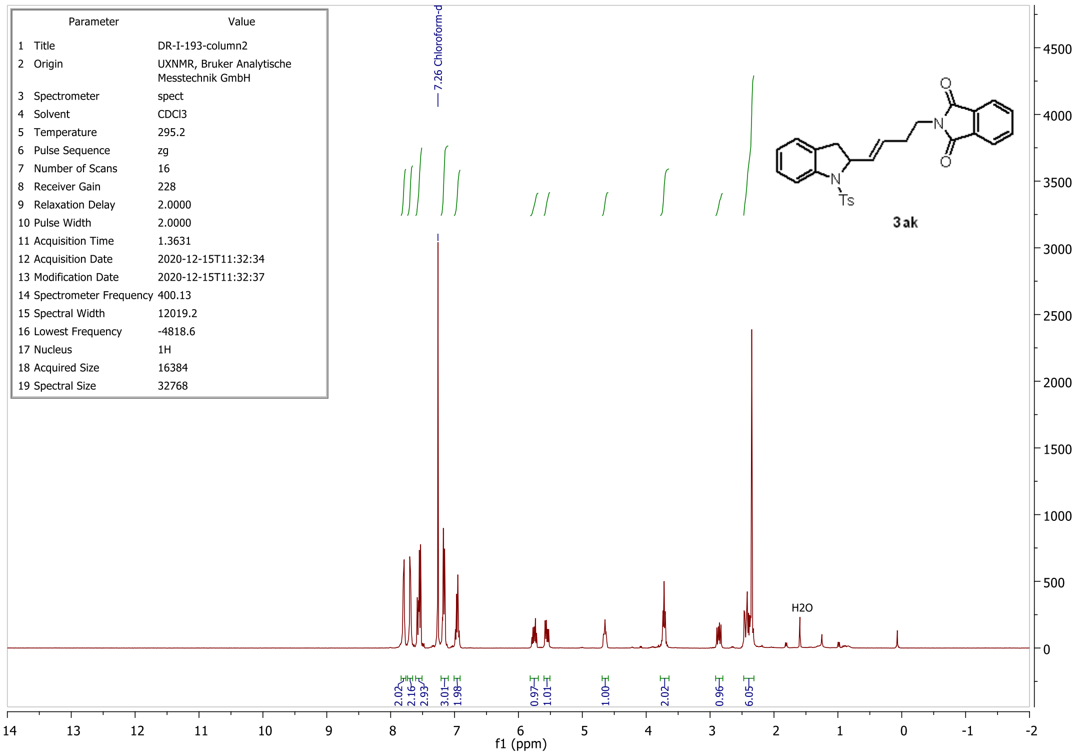

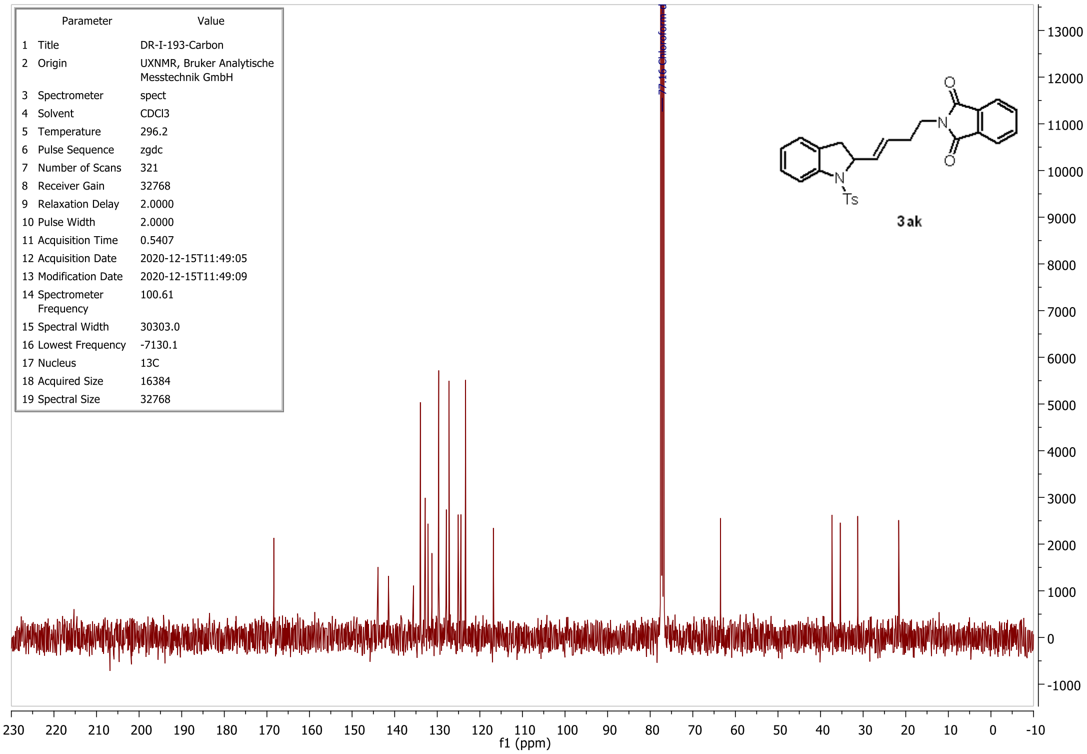

Ethyl (*E*)-4-(*N*-tosylindolin-2-yl)but-2-enoate (3al)

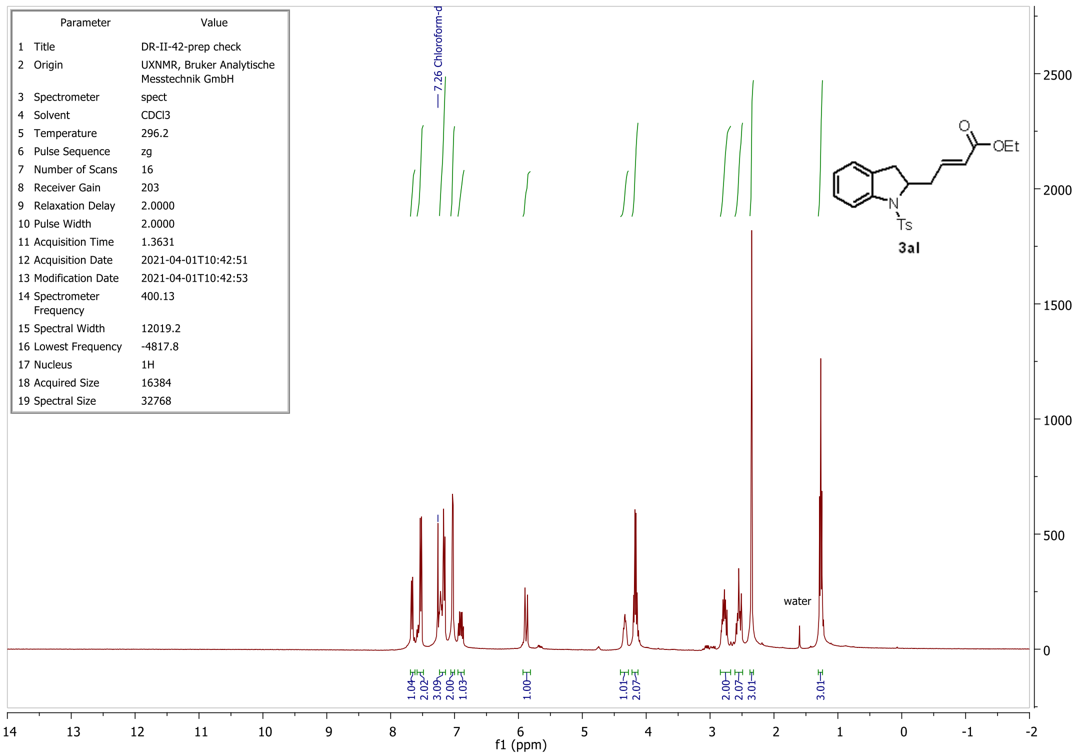

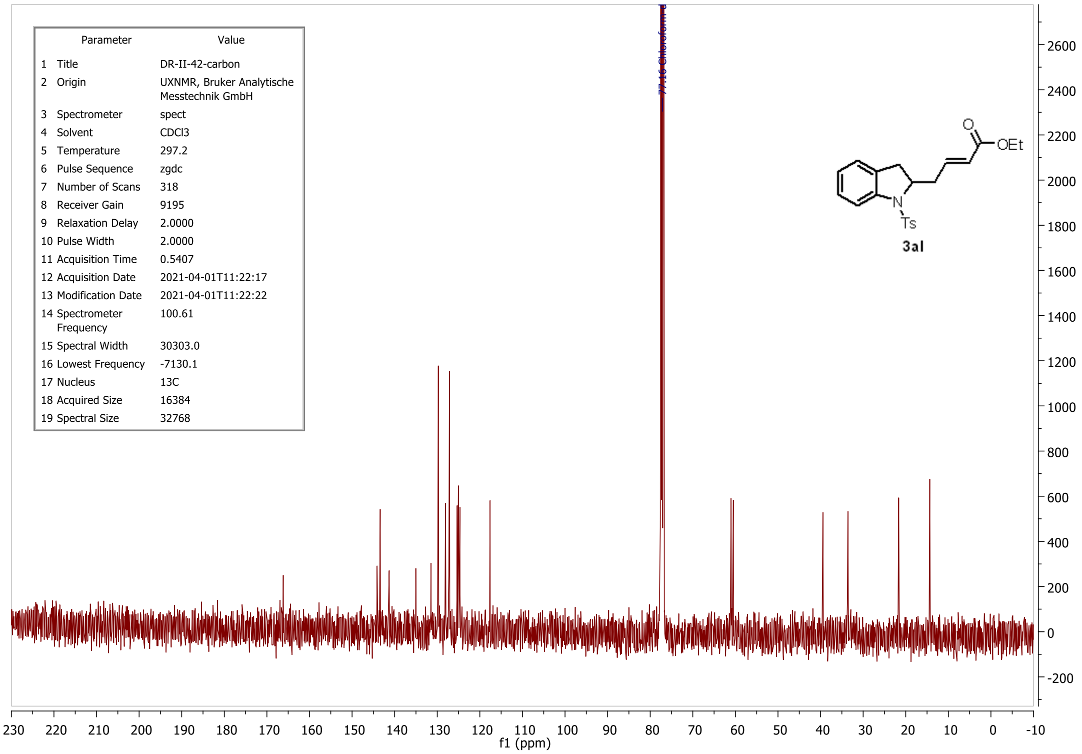

2-(Prop-1-en-2-yl)-*N*-tosylindoline (3am)

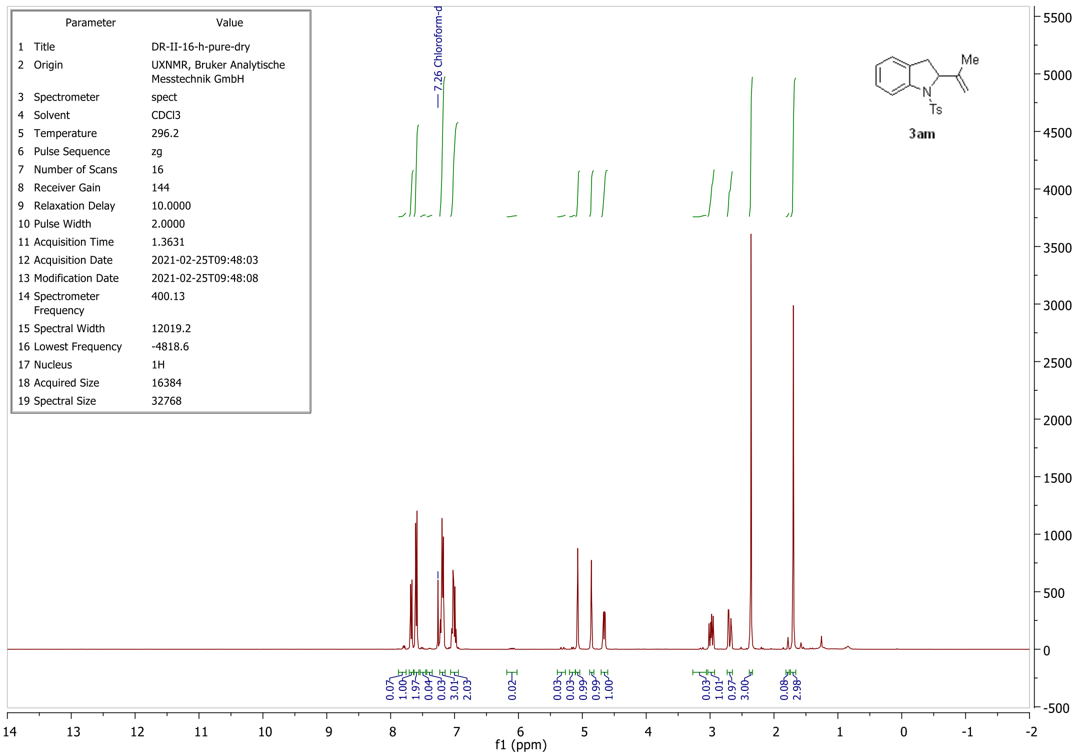

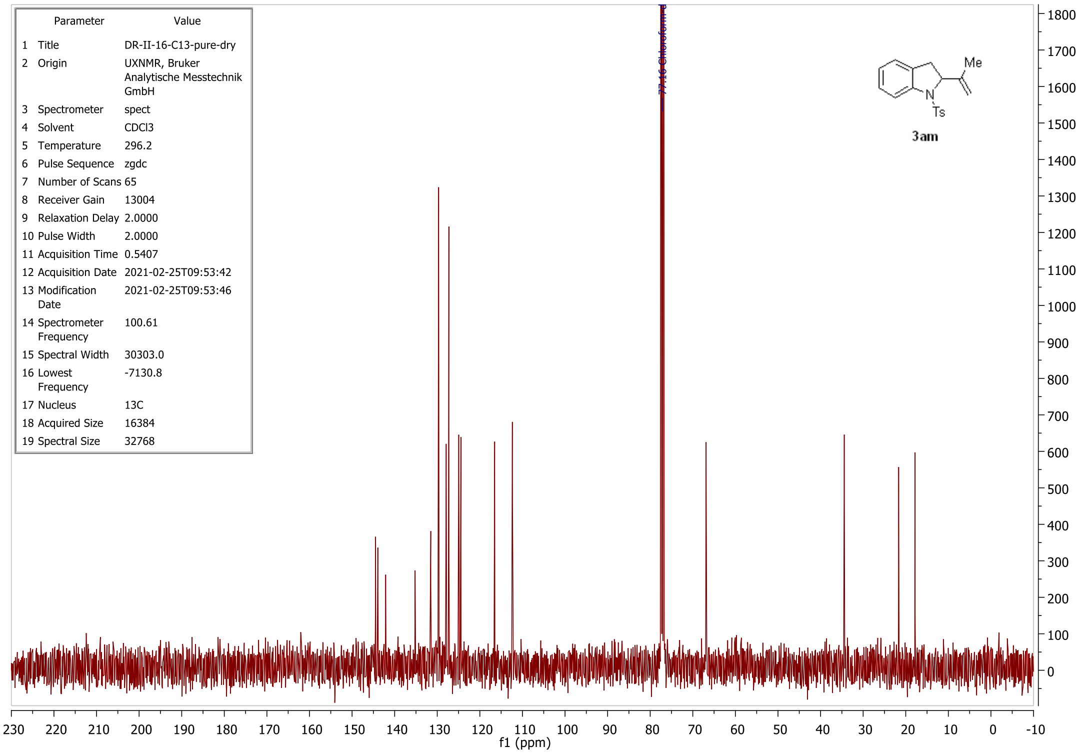

2-(4-(3,3-Dimethyloxiran-2-yl)but-1-en-2-yl)-*N*-tosylindoline (3an)

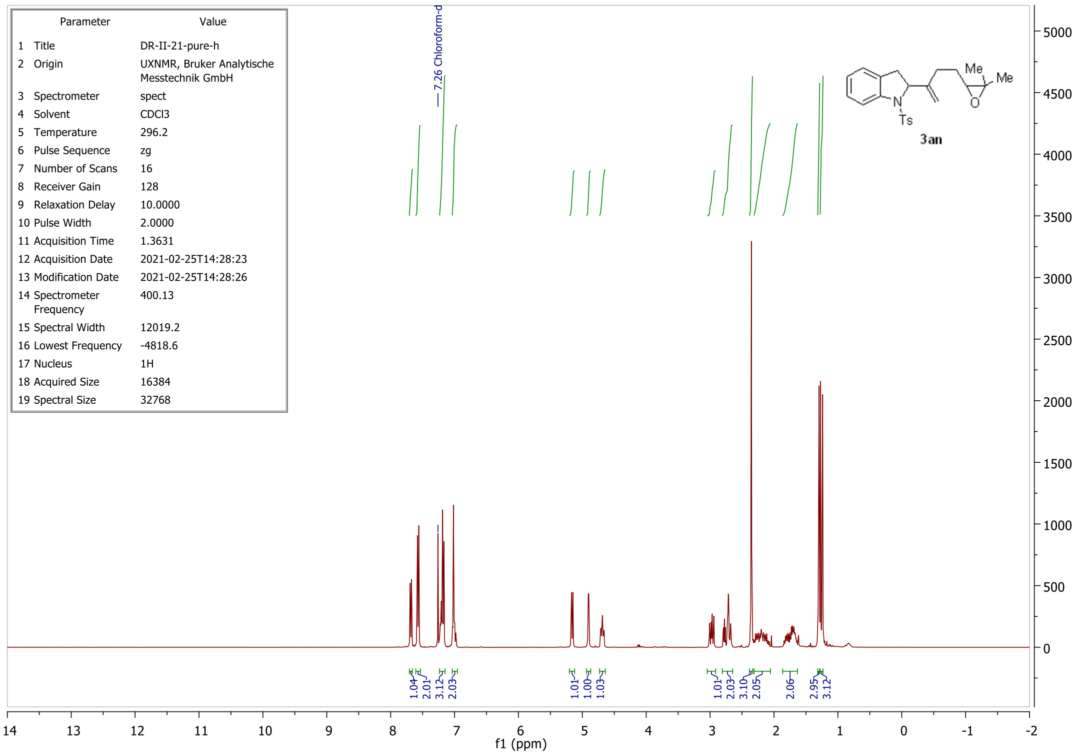

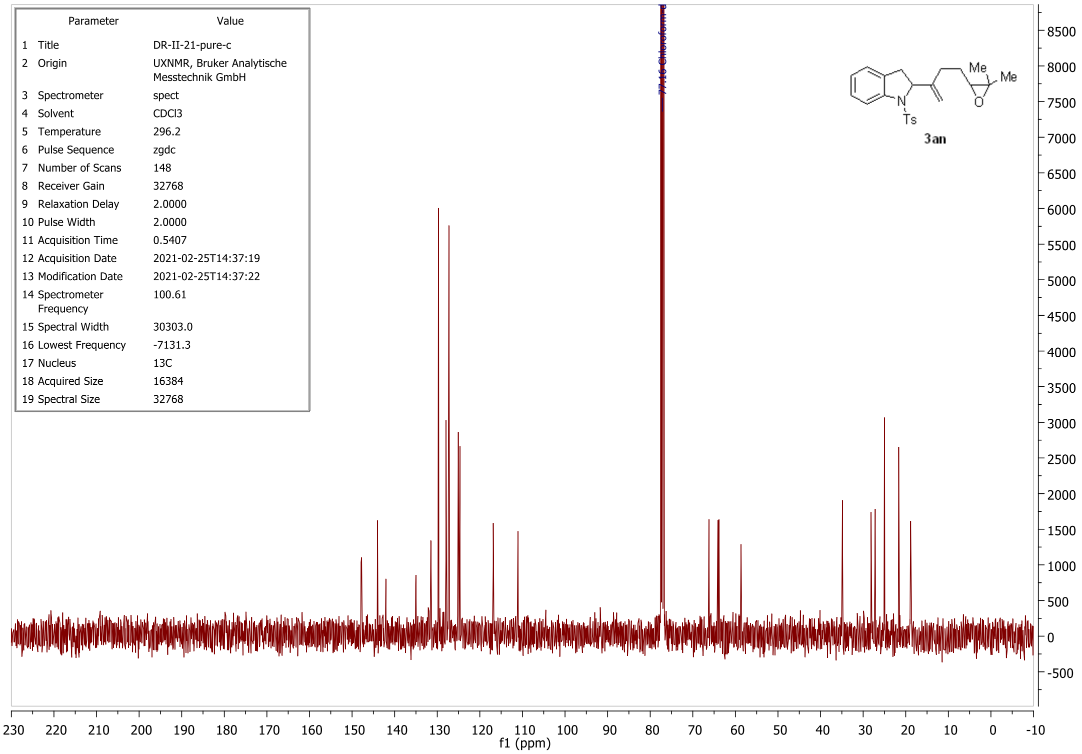

4-(*N*-Tosylindolin-2-yl)pent-4-en-1-ol (3ao)

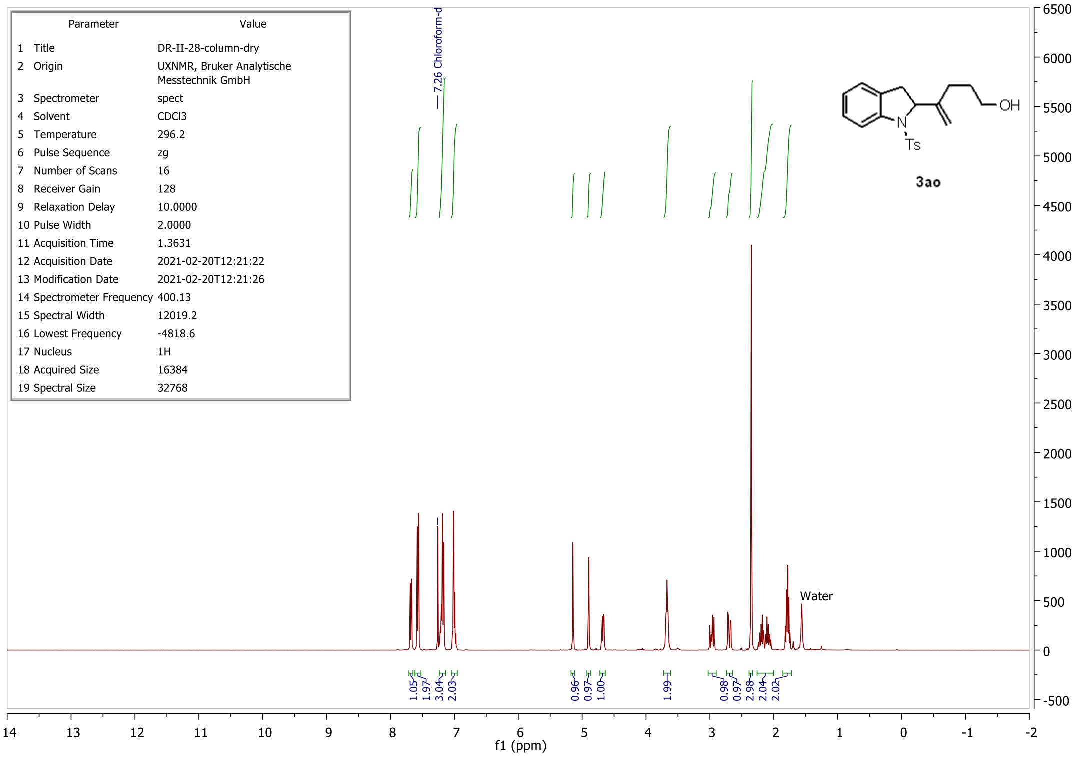

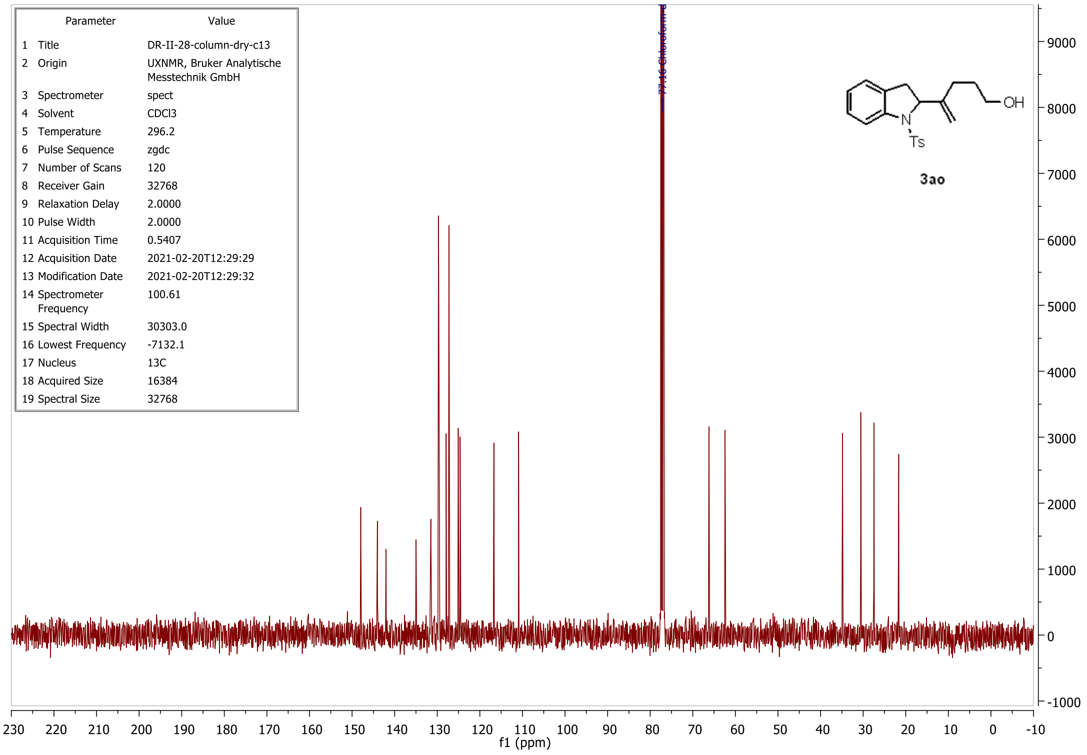

(*E*)-4-(*N*-Tosylindolin-2-yl)pent-3-en-1-ol (3ap)

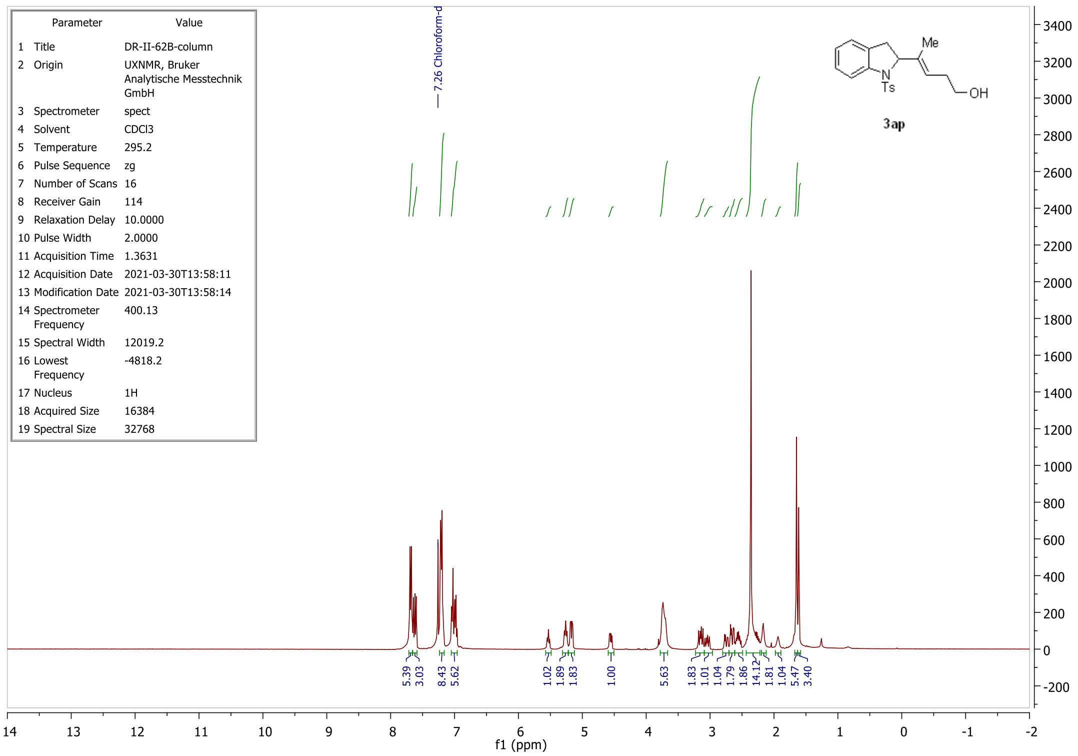

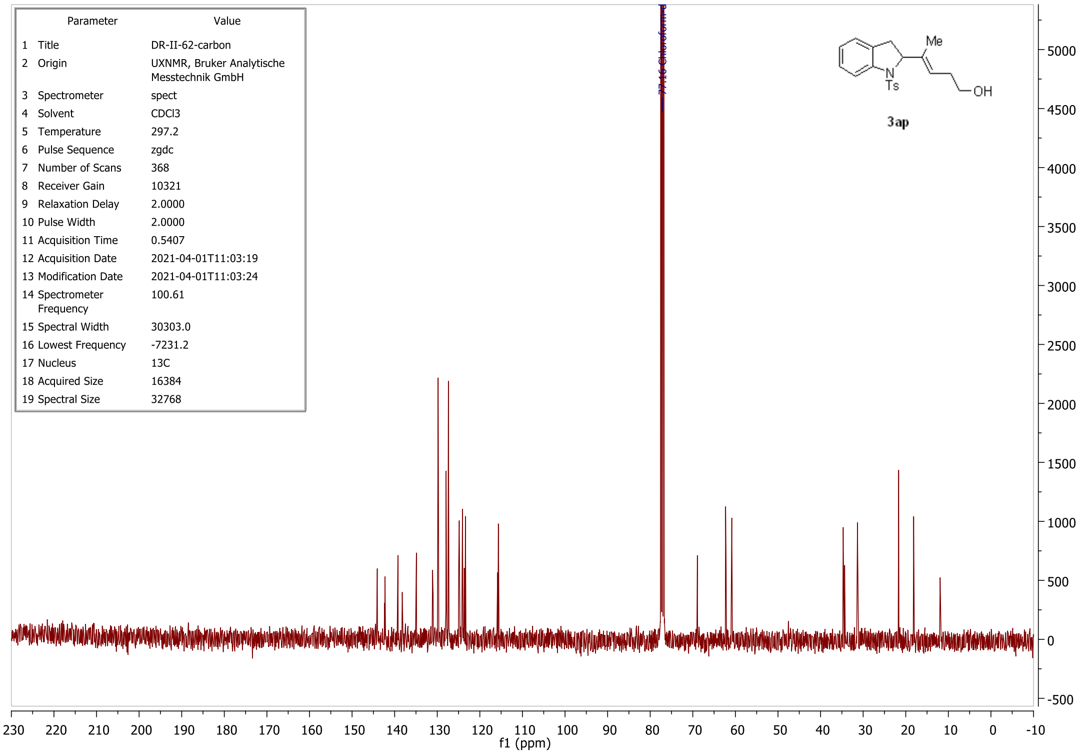

(*E*)-2-(*N*-Phenylprop-1-en-2-yl)-1-tosylindoline (3aq)

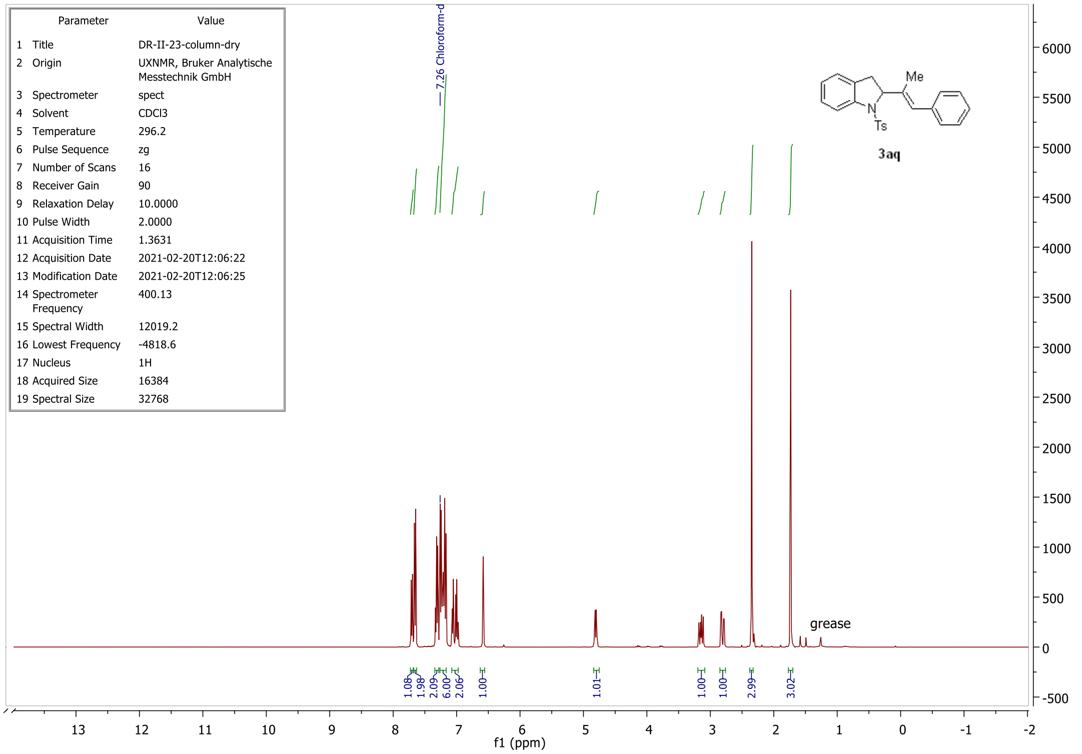

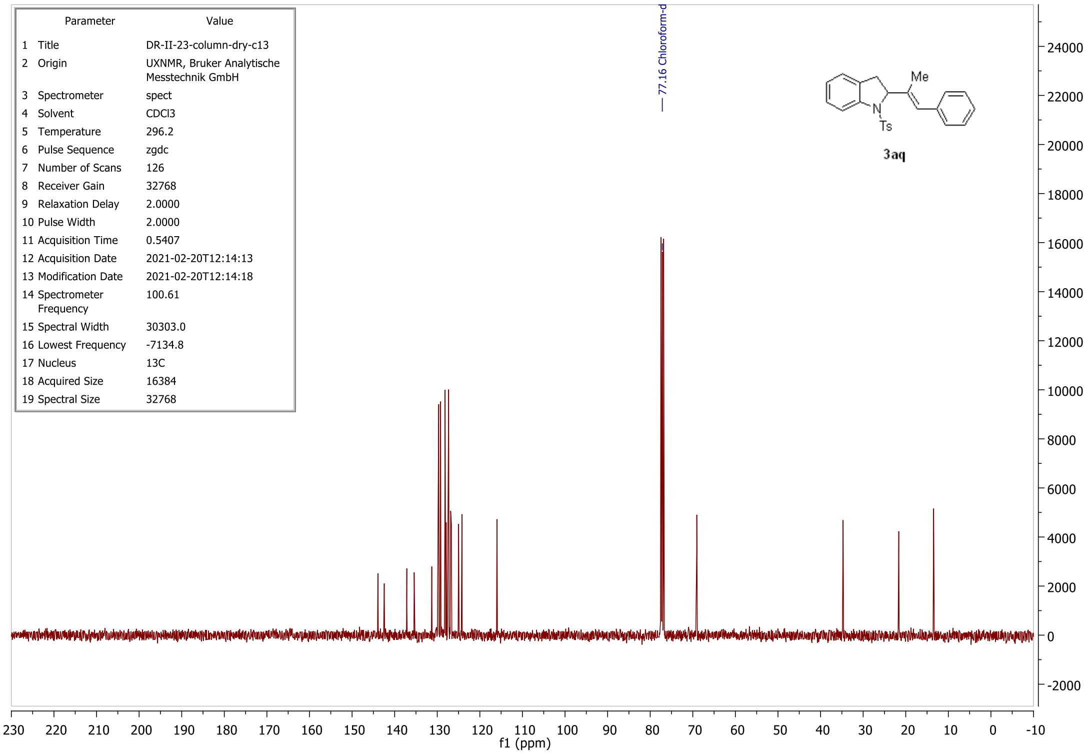

(*E*)-2-(Hex-1-en-1-yl)-2-Me-*N*-tosylindoline (3ar)

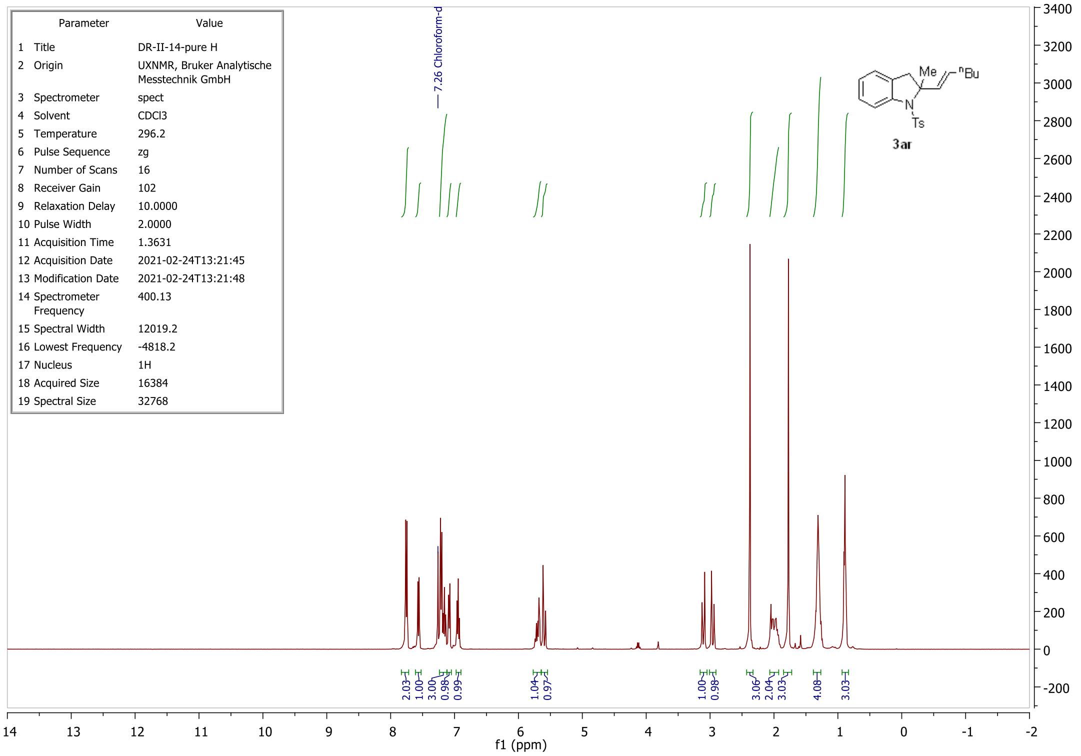

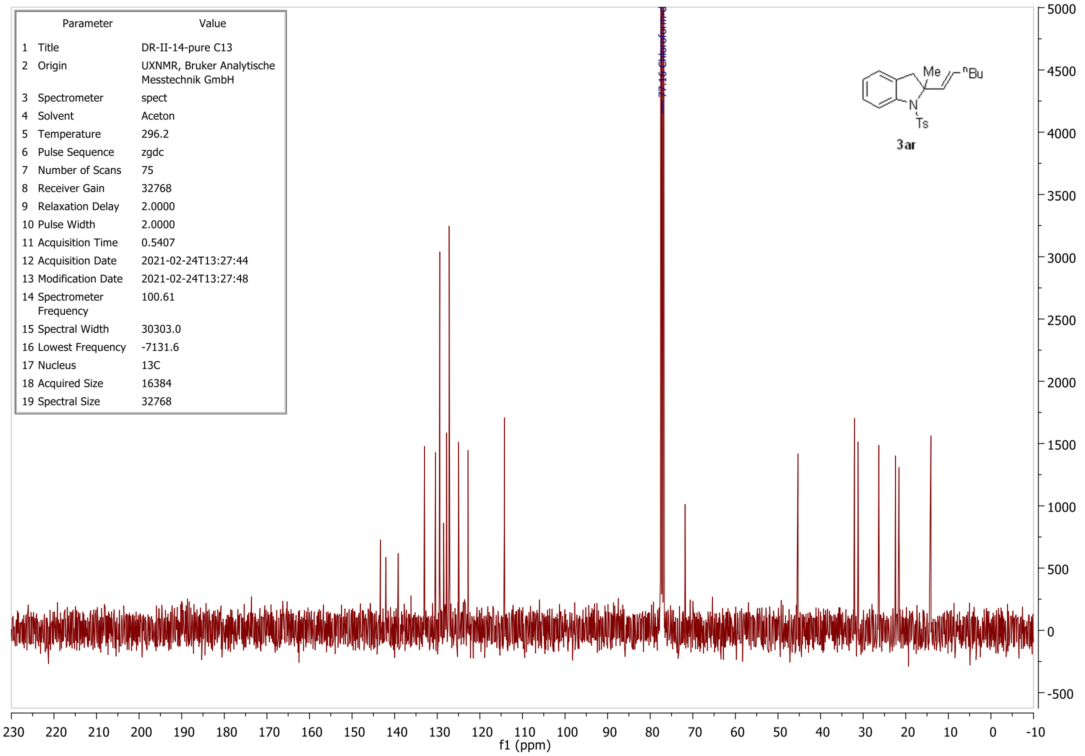

Supplement: Supplementary file 1 — ja2c01019_si_001.pdf [file ja2c01019_si_001.pdf]
